# Eight Indole Alkaloids from the Roots of *Maerua siamensis* and Their Nitric Oxide Inhibitory Effects

**DOI:** 10.3390/molecules27217558

**Published:** 2022-11-04

**Authors:** Sasiwimon Nukulkit, Angkana Jantimaporn, Preeyaporn Poldorn, Mattaka Khongkow, Thanyada Rungrotmongkol, Hsun-Shuo Chang, Rutt Suttisri, Chaisak Chansriniyom

**Affiliations:** 1 Department of Pharmacognosy and Pharmaceutical Botany, Faculty of Pharmaceutical Sciences, Chulalongkorn University, Bangkok 10330, Thailand; sasiwimon.nukulkit@gmail.com (S.N.); rutt.s@chula.ac.th (R.S.); 2 Natural Products and Nanoparticles Research Unit, Chulalongkorn University, Bangkok 10330, Thailand; 3 National Nanotechnology Center (NANOTEC), National Science and Technology Development Agency, Pathum Thani 12120, Thailand; angkana.jan@ncr.nstda.or.th (A.J.); mattaka@nanotec.or.th (M.K.); 4 Center of Excellence in Biocatalyst and Sustainable Biotechnology, Department of Biochemistry, Faculty of Science, Chulalongkorn University, Bangkok 10330, Thailand; preeyaporn.po.59@ubu.ac.th (P.P.); thanyada.r@chula.ac.th (T.R.); 5 Program in Bioinformatics and Computational Biology, Graduate School, Chulalongkorn University, Bangkok 10330, Thailand; 6 School of Pharmacy, College of Pharmacy, Kaohsiung Medical University, Kaohsiung 807, Taiwan; hschang@kmu.edu.tw

**Keywords:** *Maerua siamensis*, Capparaceae, indole alkaloids, nitric oxide inhibition

## Abstract

*Maerua siamensis* (Capparaceae) roots are used for treating pain and inflammation in traditional Thai medicine. Eight new indole alkaloids, named maeruanitriles A and B, maeroximes A–C, and maeruabisindoles A–C, were isolated from them. Spectroscopic methods and computational analysis were applied to determine the structure of the isolated compounds. Maeroximes A–C possesses an unusual O-methyloxime moiety. The bisindole alkaloid maeruabisindoles A and B possess a rare azete ring, whereas maeruabisindole C is the first indolo[3,2-*b*]carbazole derivative found in this plant family. Five compounds [maeruanitriles A and B, maeroxime C, maeruabisindoles B, and C] displayed anti-inflammatory activity by inhibiting nitric oxide (NO) production in the lipopolysaccharide-induced RAW 264.7 cells. Maeruabisindole B was the most active inhibitor of NO production, with an IC_50_ of 31.1 ± 1.8 μM compared to indomethacin (IC_50_ = 150.0 ± 16.0 μM) as the positive control.

## 1. Introduction

*Maerua* is a genus in the family Capparaceae distributed in Africa, Arabia, South Asia, and Indo-China. Up to now, 69 accepted species have been discovered [1]. However, there are only a few reports on the phytochemicals of *Maerua* plants. For example, aminoguanidine derivatives were isolated from the leaves of *Maerua edulis* from southern Africa [2], whereas lupane triterpenoids were obtained from the aerial parts of *M. oblongifolia* collected in Saudi Arabia [3] and ionol and flavonoid glycosides were found in the aerial parts of *M. crassifolia* grown in Egypt [4]. The methanolic leaf extract of *M. crassifolia* has been reported to exhibit analgesic and anti-inflammatory activities in rodent models [5]. Moreover, extracts from *M. subcordata* were able to inhibit nitric oxide (NO) production in murine macrophage and human osteosarcoma cells [6]. In traditional Thai medicine, the roots of *Maerua siamensis* (Kurz) Pax are used in several anti-inflammatory and pain-relieving preparations [7]. The plant is the only recorded species of *Maerua* in Thailand [8]. Recently, the anti-inflammatory activity of its ethanolic root extract has been demonstrated in a protein denaturation assay [9]. Previous phytochemical studies on the leaves and twigs of *M. siamensis* revealed the presence of larvicidal 1*H*-indole-3 acetonitrile glycosides [10]. These indole derivatives are similar to the ones found in plants of the Brassicaceae family, which are closely related taxonomically to Capparaceae [11] and are a source of glucosinolate-derived indole alkaloids exhibiting an anti-inflammatory activity [12].

In this paper, we reported eight new indole and oxindole alkaloids isolated from the roots of *M. siamensis*. Their structures were elucidated by spectroscopic techniques and were confirmed by molecular computational analysis. In addition, their anti-inflammatory activity, as measured by the inhibition of nitric oxide production in lipopolysaccharide (LPS)-induced RAW 264.7 macrophage cells, is presented.

## 2. Results and Discussion

### 2.1. Structure Determination of Isolated Compounds

The ethyl acetate (EtOAc) and methanol (MeOH) extracts of *M. siamensis* were preliminarily screened for their inhibition of nitric oxide production in lipopolysaccharide (LPS)-induced macrophages (RAW 267.4 cells). Following pretreatment with 100 μg/mL of EtOAc or MeOH extract, the cells exhibited nitric oxide production at 45.82 ± 11.42% and 7.65 ± 4.31%, respectively, compared to the LPS-treated control (60.82 ± 2.92 %). Eight compounds bearing a 1*H*-indole or 2-oxoindoline nucleus, including three bisindole alkaloids, were isolated from the EtOAc extract and the butanol-soluble fraction of the MeOH extract.

**Figure 1:**
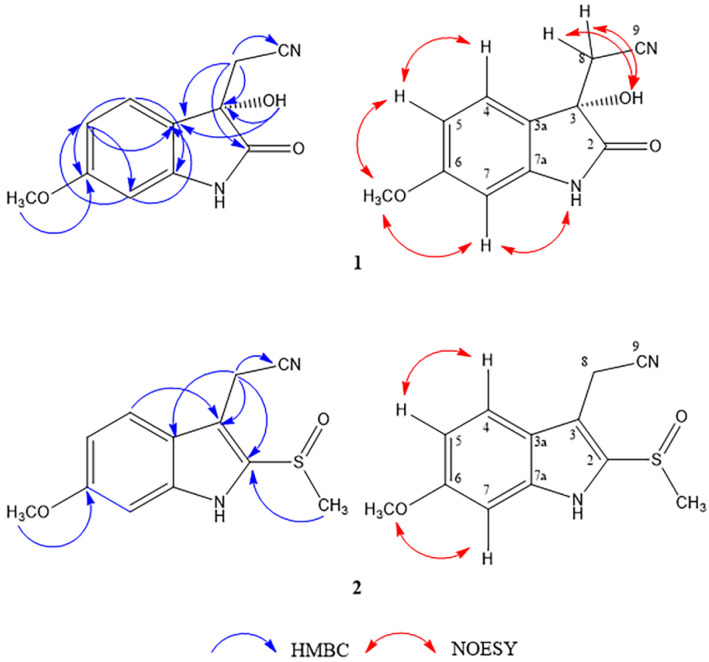
Key HMBC and NOESY correlations of compounds **1**–**2**.

Compound **1** was obtained as a reddish-brown amorphous solid displaying optical activity with [α] $\begin{array}{c} D\\ 25 \end{array}$ +3 (*c* 0.001, MeOH). Its molecular formula of C_11_H_10_N_2_O_3_, corresponding to an index hydrogen deficiency (IHD) of eight, was deduced from the sodium-adduct [M + Na]^+^ molecular ion peak at *m*/*z* 241.0585 (calculated for C_11_H_10_N_2_O_3_Na, 241.0584) in the HR-ESI-MS. Its IR spectrum displayed absorption bands due to the hydroxyl and amide NH (3291 cm^−1^, broad), aromatic ring (1630 and 1463 cm^−1^), γ-lactam carbonyl (1722 cm^−1^), and nitrile (2256 cm^−1^) components. Its UV absorption maxima at 218, 268, and 322 nm indicated oxindole moiety [13], which was supported by a carbonyl signal at δ_c_ 178.2 (C-2) and an indole NH signal at δ_H_ 9.52 (NH-1) (Table 1). The ^1^H-NMR spectrum of **1** showed peaks of an ABX spin system at δ_H_ 7.48 (1H, d, *J* = 8.4 Hz, H-4), 6.63 (1H, dd, *J* = 8.4, 2.4 Hz, H-5), and 6.53 (1H, d, *J* = 2.4 Hz, H-7). Their positions were confirmed by ^1^H-^13^C HMBC three-bond correlations [H-4 to C-6 and C-7a; H-5 to C-3a and C-7; and H-7 to C-3a and C-5]. A methoxy group, at δ_H_ 3.81/ δ_c_ 56.5, was located at C-6 by the observed HMBC correlation between its proton resonance and C-6 (δ_C_ 163.4), and ^1^H-^1^H NOESY cross peaks of 6-OCH_3_ with both H-5 and H-7. The methylene resonances at δ_H_ 3.09 (1H, d, *J* = 16.8 Hz, H-8a), and 2.89 (1H, d, *J* = 16.8 Hz, H-8b), exhibited HMBC correlations to C-2 (δ_C_ 178.2), C-3 (δ_C_ 73.8), C-3a (δ_C_ 122.8) and C-9 (δ_C_ 117.7) signals, indicating the position of an acetonitrile group at C-3 of the oxindole structure. A hydroxy group (δ_H_ 5.44) was also located at C-3, as evidenced by its HMBC correlations to C-3 and C-3a. Selected HMBC and NOESY correlations are shown in Figure 1. The chemical structure of **1** resembled that of plasiaticine C, isolated from *Plantago asiatica* (Plantaginaceae) [14], except for a methoxy instead of a hydroxyl substituent at C-6. The *S*-configuration at C-3 was determined from prominent positive and negative Cotton effects at 240 and 265.5 nm, respectively, in its CD spectrum. In addition, its Rh_2_(OCOCF_3_)_4_-CD spectrum showed a positive Cotton effect at 350 nm (see Supplementary materials A: Figure S4). These findings were in agreement with those of (+)-(*S*)-2-(3-hydroxy-4-methoxy-2-oxindolin-3-yl)acetonitrile isolated from the roots of *Isatis indigotica* (Brassicaceae) [15]. Therefore, the chemical structure of **1** was established as (+)-(*S*)-2-(3-hydroxy-6-methoxy-2-oxoindolin-3-yl)acetonitrile and named maeruanitrile A.

Compound **2** was isolated as a reddish-brown amorphous solid with the molecular formula of C_12_H_12_N_2_O_2_S (IHD 8), based on its [M + Na]^+^ ion peak at **m*/*z** 271.0511 (calcd. for C_12_H_12_N_2_O_2_SNa, 271.0512). The IR spectrum showed absorption bands that were attributable to amine NH (3164 cm^−1^, broad), nitrile (2250 cm^−1^), the aromatic ring (1627, 1451 cm^−1^), and sulfoxide (1022 cm^−1^, strong) functionalities. The ^1^H-NMR spectrum of **2** showed signals due to ABX coupling protons [δ_H_ 7.63 (1H, d, *J* = 9.0 Hz, H-4), 6.98 (1H, d, *J* = 2.4 Hz, H-7), 6.87 (1H, dd, *J* = 9.0, 2.4 Hz, H-5)], one methylene [δ_H_ 4.18 (1H, d, *J* = 18.0 Hz, H-8a) and 4.13 (1H, d, *J* = 18.0 Hz, H-8b)], one methoxy (δ_H_ 3.86, 6-OCH_3_) and one methylsulfinyl group (δ_H_ 2.16, 2-SOCH_3_). Its ^13^C-NMR spectrum showed twelve carbon signals of eight unsaturated carbons (δ_c_ 160.6, 140.3, 132.5, 121.4, 121.4, 113.4, 110.8, 95.3), one aliphatic methylene carbon (δ_c_ 13.0, C-8), one methoxy carbon (δ_c_ 55.9, 6-OCH_3_), one nitrile carbon (δ_c_ 118.9) and one methylsulfinyl carbon (δ_c_ 40.4, 2-SOCH_3_) (Table 1). These NMR data of **2** were similar to those of **1** except for the presence of an additional tetra-substituted double bond and a methylsulfinyl instead of a carbonyl group. The methylene signals (H-8a and H-8b) of the acetonitrile group appeared more downfield due to the anisotropic effect of a double bond between positions 2 and 3. This effect was also observed in indole-3-acetonitrile-2-*S*-β-glucopyranoside, which was isolated from the roots of *I. indigotica* [12]. The HMBC correlation of H-4 to a carbon signal at δ_c_ 110.8 (C-3) helped assign the carbon signal at δ_c_ 118.9 as that of the nitrile C-9. In addition, an HMBC correlations of H-8a and H-8b signals to C-2 (δ_c_ 132.5), C-3, C-3a (δ_c_ 121.4), and C-9 supported the indole-3-acetonitrile structure of **2**, and a HMBC correlation of 2-SOCH_3_ to C-2 signals, which established the position of the methylsulfinyl group (Figure 1). Thus, the structure of compound **2** was determined as 2-(6-methoxy-2-(methylsulfinyl)-1*H*-indol-3-yl)acetonitrile and given the trivial name maeruanitrile B.

**Figure 2:**
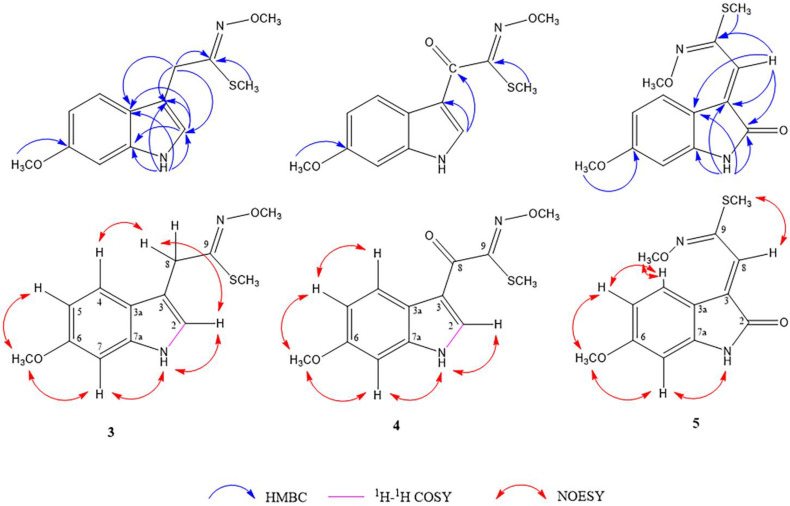
Key HMBC, ^1^H-^1^H COSY, and NOESY correlations of compounds **3**–**5**.

Compound **3** was isolated as a reddish-brown amorphous solid. The molecular formula was deduced to be C_13_H_16_N_2_O_2_S (IHD 7), according to a pseudo-molecular [M + H]^+^ ion peak at *m*/*z* 265.0999 in the HR-ESI-MS (calcd. for C_13_H_17_N_2_O_2_S, 265.1005). The IR spectrum showed absorption bands of amine NH (3370 cm^−1^, broad), O-methyloxime, and the aromatic ring (1628, 1579, 1457 cm^−1^) [16]. The ^1^H- and ^13^C-NMR spectroscopic data (Table 2) showed resonances of an ABX spin system at δ_H_ 7.33 (1H, d, *J* = 8.8 Hz, H-4), 6.84 (1H, d, *J* = 2.4 Hz, H-7), and 6.64 (1H, dd, *J* = 8.8, 2.4 Hz, H-5), an olefinic proton at δ_H_ 7.03 (1H, d, *J* = 2.0 Hz, H-2), one aliphatic methylene at δ_H_ 3.80 (2H, s, H-8), an indole NH at δ_H_ 10.72 (1H, br s, NH-1), two methoxy groups at δ_H_ 3.86 (3H, s, NOCH_3_) and 3.74 (3H, s, 6-OCH_3_) and one methylthio group at δ_H_ 2.17 (3H, s, SCH_3_). The difference in the structure of this compound from compound **2** is the absence of a substituent on C-2, as supported by a ^1^H-^1^H COSY correlation of NH-1/H-2, and the presence of substituted imine, instead of nitrile, in its side chain. A methylthio group was located at C-9 on the imine double bond, based on an HMBC correlation between its methyl signal (δ_H_ 2.17) to C-9 (δ_C_ 157.8), and a methoxy group (δ_H_ 3.86, δ_C_ 61.5) was placed on the nitrogen atom of this imine bond as an O-methyloxime substructure based on the comparison of its chemical shift with that of 2-isopropyl-5-methylcyclohexanone O-methyloxime [16]. The methyl-N-methoxyethanimidothioate-2-yl substitution at C-3 was confirmed by HMBC correlations of H-8 methylene signals (δ_H_ 3.80) to C-2 (δ_C_ 123.1), C-3 (δ_C_ 107.8), C-3a (δ_C_ 121.5), and C-9 (Figure 2). The *cis* orientation between the N-OCH_3_ and SCH_3_ groups was suggested by the lowest relative energy computation based on a DFT calculation at a B3LYP/6-31g (d,p) level (Section 2.2). Therefore, the chemical structure of **3** was proposed to be methyl (*Z*)-*N*-methoxy-2-(6-methoxy-1*H*-indol-3-yl)ethanimidothioate and named maeroxime A. Sulfur-containing indole derivatives from *M. siamensis* could be phytoalexins similar to those found in Brassicaceae plants, and glutathione-*S*-transferase (GST) might be involved in the biosynthesis of these phytochemicals [17].

Compound **4** was obtained as an orange-brown amorphous solid. Its molecular formula of C_13_H_14_N_2_O_3_S (IHD 8), determined from the HR-ESI [M + H]^+^ ion peak at *m*/*z* 279.0782 (calcd. for C_13_H_15_N_2_O_3_S, 279.0803), was 14 mass units higher than compound **3,** and thus, suggested the presence of a carbonyl instead of a methylene group. This was supported by a conjugated keto carbonyl signal (δ_C_ 182.5) in its ^13^C-NMR spectrum. The 3,6-disubstituted 1*H*-indole nucleus of **4** is identical to that of **3**, as evidenced by their ^1^H- and ^13^C-NMR data in Table 2. The difference was the presence of a carbonyl C-8 (δ_C_ 182.5), which displayed an HMBC correlation with an H-2 signal (δ_H_ 7.97) (Figure 2). Similar to compound **3**, the *cis* conformation between the N-OCH_3_ (δ_H_ 3.73, δ_C_ 61.9) and SCH_3_ groups (δ_H_ 2.43, δ_C_ 12.7) on the imine bond was proposed based on computational studies (Section 2.2). Thus, the chemical structure of **4** was established as methyl (*Z*)-*N*-methoxy-2-(6-methoxy-1*H*-indol-3-yl)-2-oxoethanimidothioate and given the trivial name maeroxime B.

Compound **5**, which was obtained as a yellow amorphous solid, gave a protonated molecular ion [M + H]^+^ at *m*/*z* 279.0780 (calcd. for C_13_H_15_N_2_O_3_S, 279.0803), indicating the same molecular formula as **4** (C_13_H_14_N_2_O_3_S). Its IR absorption bands at 3307 and 1729 cm^−1^ and UV maxima at 208, 272, and 316 nm were characteristic of the oxindole feature [12]. The presence of an amide carbonyl signal at δ_C_ 168.6 in its ^13^C-NMR spectrum also supported these observations. The IR spectrum also exhibited O-methyloxime absorption bands at 1618 and 1462 cm^−1^. Based on its ^1^H- and ^13^C-NMR data (Table 2), the difference between compounds **5** and **4** is at positions two and eight. The structure of **5** could be inferred as a 6-methoxyindolin-2-one with an olefinic methine C-8, which was supported by HMBC correlations of NH (δ_H_ 10.63, br s) to C-2 (δ_C_ 168.6), C-3 (δ_C_ 131.1), C-3a (δ_C_ 113.3), and C-7a (δ_C_ 145.6) and H-8 (δ_H_ 6.80, s) to C-2, C-3, C-3a, C-3, and C-9 (δ_C_ 150.8). The signal of SCH_3_ (δ_H_ 2.35, s) exhibited an HMBC correlation to C-9 and a NOESY interaction with H-8 (Figure 2). The orientation of the O-methyloxime group (δ_H_ 4.00, δ_C_ 62.5) was *trans* to the methylthio group, as suggested by its NOESY correlation with H-4 and computational analysis (Section 2.2). Therefore, compound **5** was established as methyl (*E*)-*N*-methoxy-2-[(*E*)-6-methoxy-2-oxindolin-3-ylidene]ethanimidothiolate and named maeroxime C.

**Figure 3:**
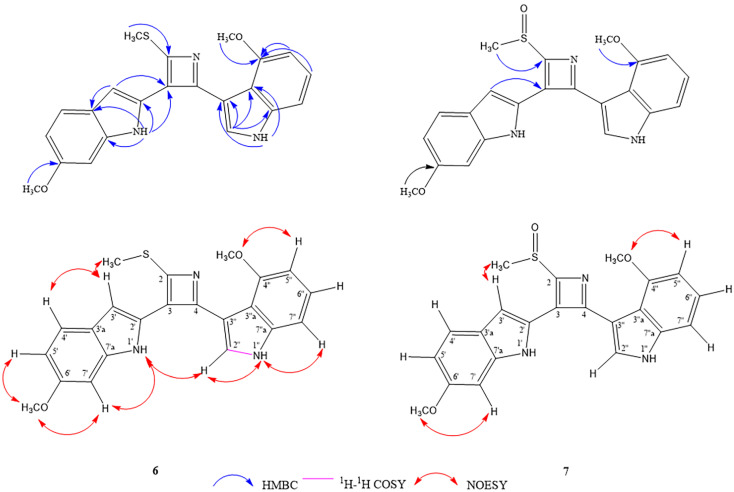
Key HMBC, ^1^H-^1^H COSY, and NOESY correlations of compounds **6**–**7**.

**Figure 4:**
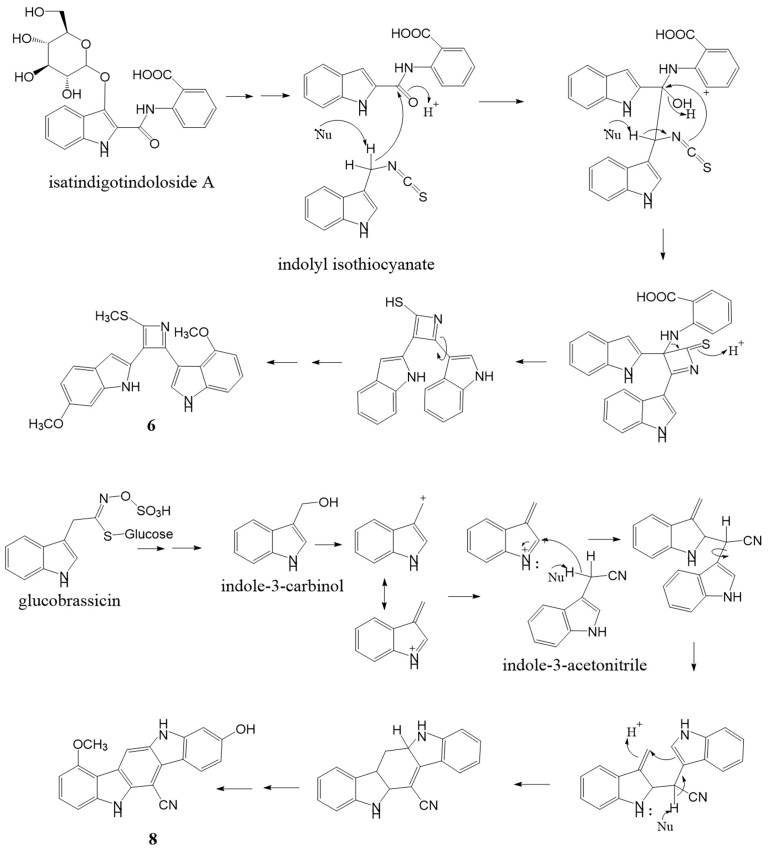
A proposed biosynthesis for **6** and **8**.

Compound **6** was isolated as a pale green amorphous solid. Its molecular formula of C_22_H_19_N_3_O_2_S (IHD 15) was deduced from an [M + H]^+^ ion peak observed in the HR-ESI-MS at *m*/*z* 390.1298 (calcd. for C_22_H_20_N_3_O_2_S, 390.1271). The ^1^H-NMR data (Table 3) displayed signals attributed to one 1,2,4-trisubstituted benzene ring [δ_H_ 8.05 (1H, d, *J* = 8.4 Hz, H-4′), 6.78 (1H, dd, *J* = 8.4, 2.0 Hz, H-5′), and 6.99 (1H, d, *J* = 2.4 Hz, H-7′)], one 1,2,3-trisubstituted benzene ring [δ_H_ 6.56 (1H, dd, *J* = 6.0, 2.4 Hz, H-5′′), 7.10 (1H, overlapped, H-6′′) and 7.12 (1H, overlapped, H-7′′)], two trisubstituted double bonds [δ_H_ 7.79 (1H, br s, H-3′) and 7.51 (1H, d, *J* = 2.0 Hz, H-2′′)], two NH [δ_H_ 10.71 (1H, br s, NH-1′) and 11.52 (1H, br s, NH-1′′)], two methoxys [δ_H_ 3.81 (3H, s, 6′-OCH_3_) and 3.52 (3H, s, 4′′-OCH_3_)] and one methylthio group (δ_H_ 2.59, 3H, s), implying a bisindole alkaloid structure. Its ^13^C-NMR spectrum (Table 3) showed three quaternary carbon signals of an azete moiety [δ_C_ 144.8 (C-2), 139.5 (C-4), 133.4 (C-3)], and an SCH_3_ methyl signal (δ_C_ 14.0). The 6′-methoxy-1′*H*-indole-2′-yl substructure was confirmed by key HMBC correlations [NH-1′ to C-2′, C-3′a, C-7′a; H-3′ to C-3′a; 6′-OCH_3_ to C-6′] and NOESY correlations [NH-1′/H-7′, 6′-OCH_3_/H-5′, 6′-OCH_3_/H-7′]. The 4″-methoxy-1″*H*-indole-3″-yl substructure was confirmed by HMBC correlations [NH-1′′ to C-3′′, C-3”a; H-2′′ to C-3′′, C-3′′a, C-7′′a; H-5′′, H-6′′ and 4′′-OCH_3_ to C-4′′], ^1^H-^1^H COSY cross peak between NH-1″/H-2″ and NOESY correlations [NH-1′′/H-2′′, NH-1′′/H-7′′, 4′′-OCH_3_/H-5] (Figure 3). Both indole subunits were connected through a 2,3,4-trisubstituted azete ring similar to the sulfur-containing bisindole alkaloids isatindigosides F and G from *Isatis tinctoria* roots [18]. The methylthio group was placed at position 2 on the azete ring based on an HMBC correlation of its methyl singlet to C-2 (δ_C_ 144.8), while the 6′-methoxy-1′*H*-indole-2′-yl moiety was connected to C-3 (δ_C_ 133.4), as indicated by HMBC correlations from both H-3′ and NH-1′ signals to this position on the azete ring. Therefore, the 4′′-methoxy-1′′*H*-indole-3′′-yl moiety could be located at position 4 of the same ring and furnished the chemical structure of **6** as 4-methoxy-3-[3-(6-methoxy-1*H*-indol-2-yl)-2-(methylthio)azet-4-yl]-1*H*-indole. Compound **6** was named maeruabisindole A, and its biosynthetic pathway was proposed, as shown in Figure 4.

Compound **7** was obtained as a pale green amorphous solid. Its molecular formula of C_22_H_19_N_3_O_3_S was confirmed by the pseudo-molecular [M + H]^+^ ion at *m*/*z* 406.1224 (calcd. for C_22_H_20_N_3_O_3_S, 406.1220) in the HR-ESI mass spectrum. Its IR spectrum was similar to **6** except for the presence of a sulfoxide absorption peak (1025 cm^−1^). In its ^1^H- and ^13^C-NMR spectra, resonances of a methylsulfinyl group were observed at δ_H_ 2.98 and δ_C_ 42.1, respectively, replacing the methylthio ones of compound **6**. Otherwise, the NMR data representing both indole subunits of this bisindole alkaloid were similar to those of the previous compound (Table 3). Three sp^2^ carbons of an azete ring resonated at δ_C_ 151.7 (C-2), δ_C_ 142.7 (C-4), and δ_C_ 137.8 (C-3) in its ^13^C-NMR spectrum. A methylsulfinyl substitution at C-2 and the linkage of the 6′-methoxy-1′*H*-indole-2′-yl moiety to C-3 of the azete ring were confirmed by HMBC correlations from the 2-SOCH_3_ signal to C-2 and from the H-3′ signal (δ_H_ 8.43) to C-3, respectively. The more downfield chemical shift of H-3′, compared to that of **6**, could be due to the anisotropic effect of the S=O bond on the nearby methylsulfinyl group. NOESY correlations of 2-SOCH_3_/H-3′, 6′-OCH_3_/H-7′ and 4”-OCH_3_/H-5” supported the proposed structure of **7** as 4-methoxy-3-[3-(6-methoxy-1*H*-indol-2-yl)-2-(methylsulfinyl)azet-4-yl]-1*H*-indole (Figure 3). Compound **7** was named maeruabisindole B.

**Figure 5:**
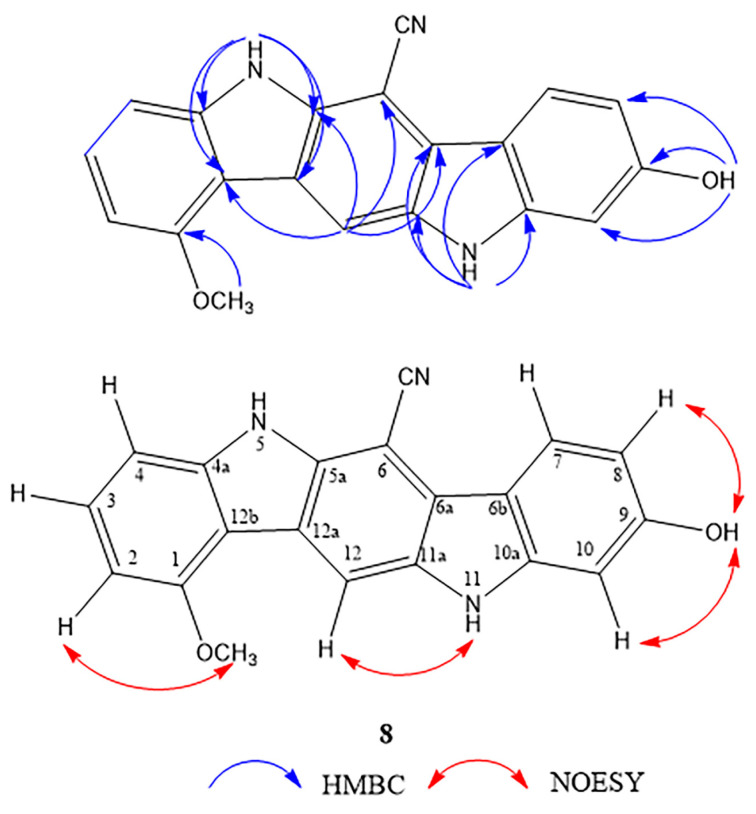
Key HMBC and NOESY correlations of compound **8**.

Compound **8** was isolated as a dark green amorphous solid. Its molecular formula was established as C_20_H_13_N_3_O_2_ (IHD 16)_,_ based on the HR-ESI [M − H]^−^ ion at *m*/*z* 326.0968 (calcd. for C_20_H_12_N_3_O_2_, 326.0935). The IR spectrum showed absorption bands due to hydroxyl and amine NH (3359 cm^−1^), nitrile (2212 cm^−1^), and the aromatic ring (1632 and 1468 cm^−1^). The ^1^H-NMR data (Table 4) show signals of one 1,2,3-trisubstituted benzene ring [δ_H_ 6.80 (1H, d, *J* = 8.0 Hz, H-2), 7.40 (1H, t, *J* = 8.0 Hz, H-3) and 7.24 (1H, d, *J* = 8.0 Hz, H-4)], one 1,2,4-trisubstituted benzene ring [δ_H_ 8.33 (1H, d, *J* = 8.8 Hz, H-7), 6.86 (1H, dd, *J* = 8.8, 2.4 Hz, H-8) and 7.02 (1H, d, *J* = 2.4 Hz, H-10)], an aromatic proton at δ_H_ 8.53 (1H, s, H-12), two NH protons at δ_H_ 10.86 (1H, br s, NH-5) and 10.39 (1H, br s, NH-11), a methoxy group at δ_H_ 4.12 (3H, s, 1-OCH_3_) and a hydroxyl group at δ_H_ 8.70 (1H, br s, 9-OH). The ^13^C NMR spectra exhibited eleven quaternary and seven methine carbon resonances, a methoxy signal at δ_C_ 56.0 (1-OCH_3_), and a nitrile carbon signal at δ_C_ 118.3 (6-CN). These data indicate that the structure of **8** comprised two indole rings connected into the core structure of indolo[3,2-*b*]carbazole [19]. HMBC cross peaks from NH-5 (δ_H_ 10.86) to C-5a (δ_C_ 138.0), C-4a (δ_C_ 143.4), C-12a (δ_C_ 122.0), and C-12b (δ_C_ 113.0), from NH-11 (δ_H_ 10.39) to C-6a (δ_C_ 123.0), C-6b (δ_C_ 115.4), and C-10a (δ_C_ 144.6) and from H-12 (δ_H_ 8.53) to C-5a, C-6a, C-6 (δ_C_ 82.5), and C-12b confirmed this skeleton. A methoxy group was located at C-1, as evidenced by an HMBC correlation of its proton signal (δ_H_ 4.12) with C-1 (δ_C_ 157.1) and a NOESY correlation of 1-OCH_3_/H-2. The hydroxy substituent on C-9 was proven by HMBC correlations of its signal (δ_H_ 8.70) to C-8 (δ_C_ 110.0), C-9 (δ_C_ 158.8), and C-10 (δ_C_ 97.5), as well as NOESY correlations of 9-OH/H-8 and 9-OH/H-10. NOESY cross peak was also observed between H-12/NH-11 (Figure 5). Finally, the nitrile group could be placed at position 6 of the indolo[3,2-*b*]carbazole nucleus. The downfield chemical shifts of H-7 and NH-5 signals might be due to the anisotropic effect of this nitrile group. Thus, compound **8** was elucidated as 9-hydroxy-1-methoxy-5,11-dihydroindolo[3,2-*b*]carbazole-6-carbonitrile and trivially named maeruabisindole C. Indolo[3,2-*b*]carbazole compounds are metabolites of indole-3-carbinol, which can occur from the breakdown of a glucobrassinin under acid condition [20]. In the stomach, they can be formed during the digestion of *Brassica* vegetables and are beneficial to gut immune function [21]. The biosynthesis of maeruabisindole C has been proposed and shown in Figure 4.

### 2.2. Computational Analysis

**Figure 6:**
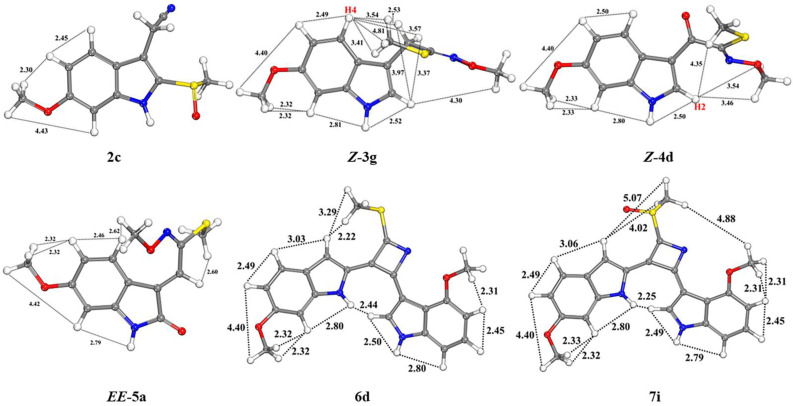
The most stable conformers due to the lowest relative energy of compounds **2**–**7**, based on the DFT calculation at B3LYP/6-31g (d,p) level in the DMSO for compounds **3**–**6**, and MeOH for compounds **2** and **7** (see Supplementary materials B: Computational studies).

Configuration of compound **1** and conformational analysis of compounds **2**–**7** were optimized by the density of the functional theory (DFT) at a B3LYP/6-31G(d,p) level. Theoretical ECD spectra of maeruanitrile A (**1**) were computed using a time-dependent–density functional theory (TD–DFT) method at a B3LYP/6-311++G(d,p) level. The ECD spectrum of (*S*)-**1** agreed with the experimental CD spectrum of **1** and supported the assignment of the *S* configuration on C-3 (see Supplementary materials A: Figure S4). Based on the lowest relative energy, the proposed conformational structures of maeruanitrile B (**2**) and maeruabisindoles A and B (**6**–**7**), and geometrical conformations of maeroximes A–C (**3**–**5**) were consistent with their NOESY interactions (Figure 6).

### 2.3. Inhibition of Nitric Oxide Production of Isolated Compounds

Nitric oxide (NO) is an effector mediator in the immune system synthesized by nitric oxide syntheses (NOS). When infection or tissue injury occurs, macrophages are stimulated by pro-inflammatory cytokines [tumor necrosis factor α- (TNF-α), interleukin-1 (IL-1)], and/or lipopolysaccharide (LPS). The inducible NOS gene is subsequently expressed, and NO is produced [22,23]. In chronic inflammation, NO can stimulate cyclooxygenase-2 (COX-2) activity, resulting in increased prostaglandin production and the pathogenesis of inflammation [24]. Thus, screening for inhibitors of nitric oxide production is an important step to determine the potential anti-inflammatory activity of the test compounds.

All isolates were evaluated for anti-inflammatory activities in the LPS-induced RAW 267.4 cells. Maeroxime C (**5**) and maeruabisindoles B (**7**) and C (**8**) showed a nitric oxide inhibition activity stronger than indomethacin (*p* < 0.005, *p* < 0.0001 and *p* < 0.0001, respectively (see Table 5 and Supplementary materials A: Figures S97 and S98), while maeruanitriles A (**1**) and B (**2**) were as strong as indomethacin against nitric oxide production. Having an acetonitrile substituent at C-3, maeruanitriles A (**1**) and B (**2**) demonstrated an NO inhibitory activity similar to indole-3-acetonitrile compounds isolated from *Isatis indigotica* roots [12]. Among the 1*H*-indole isolates, maeruanitrile B (**2**), which possesses acetonitrile and methylsulfinyl substitution at C-2 and C-3, respectively, exhibited a higher inhibitory activity on NO production than maeroxime B (**4**) (*p* < 0.05). In the oxindole series, maeroxime B (**5**) showed higher NO inhibitory activity than maeruanitrile A (**1**) (*p* < 0.0001). This is also the first report on the NO inhibitory activity of (*E*)-*N*-methoxy-2-[(*E*)-6-methoxy-2-oxindolin-3-ylidene]ethanimidothiolate. For bisindole alkaloids, maeruabisindole B (**7**) showed strong NO inhibitory activity at IC_50_ of 31.1 ± 1.8 μM, whereas maeruabisindole A (**6**) was too cytotoxic. Similar bisindoles with an azete ring, i.e., isatindigosides F and G, also exhibited NO inhibitory activity with IC_50_ of 70.3 ± 6.9 and 67.3 ± 5.5 μM, respectively [18]. Maeruabisindole C (**8**) inhibited NO production with IC_50_ of 56.7 ± 3.8 μM. This is the first report on the NO inhibitory activity of the indolo[3,2-*b*]carbazole derivative. Bisindole compounds from *Isatis tinctoria* roots (synonym *Isatis indigotica* [25]) have been reported to exhibit this activity [26,27].

## 3. Materials and Methods

### 3.1. General Experimental Procedures

UV spectra were obtained by a Milton Roy Spectronic 3000 Array spectrophotometer (Rochester, NY, USA). Optical rotations were measured on a JASCO P-2000 polarimeter (Kyoto, Japan). CD spectra were recorded using a Jasco J-815 CD spectrophotometer (Kyoto, Japan). IR spectra were recorded on a Nicolet^TM^ iS50 FT-IR spectrometer (Thermo Fisher Scientific, Waltham, MA, USA) and Perkin Elmer FT-IR 1760X spectrometer (Boston, MA, USA). HR-ESI spectra were measured on a Bruker APEX II mass spectrometer (Karlsruche, Germany) and Agilent 6540 UHD Accurate-Mass Q-TOF mass spectrometer (CA, USA). NMR was recorded on a Bruker Advance NEO 400 MHz NMR spectrometer (Karlsruche, Germany) and a Varian VNMRS-600 spectrometer (Lexington, MA, USA). Medium-performance liquid chromatography (MPLC) and flash column chromatography (Flash CC) were performed by using a PuriFlash^®^ XS 420 (Advion Inc., NY, USA), Sepacore^®^ purification system (Buchi AG, Flawill, Switzerland), or a ceramic pump (VSP-3050; EYELA, Kyoto, Japan). Silica gel 60 (70–230 or 230–400 mesh ASTM, Merck, Darmstadt, Germany), LiChroprep^®^ RP-18 (25–40 μm, Merck, Darmstadt, Germany), and Sephadex^TM^ LH-20 (GE Healthcare, Amersham, UK) were used as a stationary phase material for column chromatography (CC). Organic solvents (commercial grade) were redistilled prior to their use as a mobile phase composition.

### 3.2. Plant Material

The roots of *Maerua siamensis* (Kurz) Pax. were collected in the Sikhio district, Nakhon Ratchasima province, and identified by one of the authors (C.C.) according to the Botanical Garden Organization (BGO) plant database, Thailand. A voucher specimen (CC-MS-0419) has been deposited at the herbarium of the Department of Pharmacognosy and Pharmaceutical Botany, Faculty of Pharmaceutical Sciences, Chulalongkorn University, Thailand.

### 3.3. Extraction and Isolation

Dried roots were cut into small pieces and extracted with EtOAc (3 × 30 L) to yield the EtOAc extract (29.3 g). The marc was further extracted with MeOH (3 × 30 L) to obtain the MeOH extract (350 g). The MeOH extract was mixed with distilled water and partitioned with n-butanol (3 × 5 L) to give an n-butanol extract (50.8 g).

The EtOAc extract was separated by FCC using silica gel (Si) as the stationary phase, and the mobile phase comprised *n*-hexane (C_6_H_12_)/acetone (Me_2_CO) from 15:1 to 6:1 to gain nine fractions (Fr.1-9). Fr.6 (274.50 mg) was loaded on MPLC [Si, C_6_H_12_/Me_2_CO (3:1)] to yield ten fractions (Fr. 6-1 to 6-10). Fr. 6-5 (38.2 mg) was further separated by MPLC [C18-reverse phase silica gel (RP-18), deionized (DI) water/MeCN (1:2)] to obtain ten fractions (Fr. 6-5-1 to 6-5-10). Fr. 6-5-2 (8 mg) was further purified by preparative RP-18 TLC, which was developed with DI water/MeCN (1:4) to afford compound **3** (2.4 mg). Fr. 8 (2.14 g) was subjected to MPLC [Si, dichloromethane (CH_2_Cl_2_)/Me_2_CO (120:1 to 20:1)] to yield twelve fractions (Fr. 8-1 to 8-12). Fr. 8-2 (66.6 mg) before being separated by MPLC [Si, C_6_H_12_/CH_2_Cl_2_/Me_2_CO (8:1:1 to 4:1:1)] to gain twelve fractions (Fr. 8-2-1 to 8-2-12), and then thrice separated by the mobile phase ratio of 4:1:1 to 2:1:1 to give compound **1** (2.4 mg) and compound **2** (1.4 mg). Fr. 8-6 (31.9 mg) was subjected to MPLC [Si, C_6_H_12_/Me_2_CO (10:1 to 6:1)] to yield sixteen fractions (Fr. 8-6-1 to 8-6-16). Fr. 8-6-13 was purified by MPLC [Si, C_6_H_12_/Me_2_CO (6:1)] to gain five fractions (Fr. 8-6-13-1 to 8-6-13-5). Fr. 8-6-13-3 was separated by CC [Si, C_6_H_12_/Me_2_CO (3:1)] to gain compound **4** (1.0 mg). Fr. 8-9 (158.00 mg) was purified by MPLC [Si, C_6_H_12_/CH_2_Cl_2_/Me_2_CO (8:1:1) and C_6_H_12_/CH_2_Cl_2_/Me_2_CO (6:1:1)], and Sephadex LH-20 (MeOH) to give compound **5** (3.5 mg).

Butanol extract (50.8 g) was separated by Sephadex LH-20 (MeOH) to obtain five fractions (Fr. A-E). Fr. C (5.7 g) was subjected to MPLC [Si, CH_2_Cl_2_/Me_2_CO (10:1 to 1:1)] to obtain sixteen fractions (Fr. C-1 to C-16). Fr. C1-7 (1.56 g), before it was separated by MPLC [Si, CH_2_Cl_2_/Me_2_CO (10:1)] to obtain 10 fractions (Fr. C-1-7-1 to C-1-7-10). Fr. C-1-7-5 (283.5 mg) was purified repeatedly by MPLC and Sephadex LH-20 (MeOH) to obtain compound **7** (1.1 mg). Fr. C-15 (3.58 g) was separated by MPLC [Si, CH_2_Cl_2_/Me_2_CO (6:1)] into five fractions (Fr. C15-1 to C15-5). Fr. C15-4 (8.6 mg) was purified by Sephadex LH-20 (MeOH) to obtain compound **6** (2.8 mg). Fr. E3 (2.31g) was loaded on MPLC [Si, CH_2_Cl_2_/Me_2_CO (10:1)] to yield compound **8** (3.5 mg).

Compound **1** (maeruanitrile A): reddish brown amorphous; [α] $\begin{array}{c} D\\ 25 \end{array}$ + 3.0 (c 0.001, MeOH); UV λ_max_ (MeOH) nm (log ε): 218 (5.49), 268 (4.66), 322 (4.04); CD (c 4.5 × 10^−5^, MeOH) nm (mdeg): 240 (+8.20), 265.5 (−9.64), 283.0 (0.07); Rh_2_(OCOCF_3_)_4_-induced CD (CH_2_Cl_2_) nm (Mol CD): 350.0 (+1.51); IR (ATR) ν_max_: 3291, 2256, 1789, 1629, 1462, 1342, 1722 cm^−1^; ^1^H and ^13^C-NMR data (acetone-*d*_6_): see Table 1; HR-ESI-MS *m*/*z* 241.0585 (calcd. for C_11_H_10_N_2_O_3_Na, 241.0584).

Compound **2** (maeruanitrile B): reddish brown amorphous; UV λ_max_ (MeOH) nm (log ε): 228 (4.85), 300 (4.39), 342 (3.77); IR (ATR) ν_max_: 3163, 2924, 2850, 2360, 2249, 1626, 1451, 1298, 1208, 1160, 1022 cm^−1^; ^1^H and ^13^C-NMR data (CD_3_OD): see Table 1; HR-ESI-MS *m*/*z* 271.05112 (calcd. for C_12_H_12_N_2_O_2_SNa, 271.05172).

Compound **3** (maeroxime A): reddish brown amorphous; UV λ_max_ (MeOH) nm (log ε): 212 (5.12), 217 (5.26), 225 (4.80), 269 (4.15); IR (ATR) ν_max_: 3369, 2923, 2852, 1714, 1627,1501, 1457, 1337, 1198, 1093 cm^−1^; ^1^H and ^13^C-NMR data (DMSO-*d*_6_): see Table 2; HR-ESI-MS *m*/*z* 265.0999 (calcd. for C_13_H_16_N_2_O_2_S, 265.1010).

Compound **4** (maeroxime B): orange-brown amorphous; UV λ_max_ (MeOH) nm (log ε): 212 (4.69), 280 (3.25), 314 (4.18); IR (ATR) ν_max_: 3283, 2924, 2851, 1718, 1617, 1521, 1421, 1241, 1197, 1074, 1032 cm^−1^; ^1^H and ^13^C-NMR data (DMSO-*d*_6_): see Table 2; HR-ESI-MS *m*/*z* 279.0780 (calcd. for C_13_H_14_N_2_O_3_S, 279.0803).

Compound **5** (maeroxime C): yellow amorphous; UV λ_max_ (MeOH) nm (log ε): 208 (4.80), 272 (4.47), 316 (4.15); IR (ATR) ν_max_: 3306, 2956, 2924, 2854, 1729, 1618, 1461, 1378, 1283, 1074, 1037 cm^−1^; ^1^H and ^13^C-NMR data (DMSO-*d*_6_,): see Table 2; HR-ESI-MS *m*/*z* 279.0782 (calcd. for C_13_H_14_N_2_O_3_S, 279.0803).

Compound **6** (maeruabisindole A): pale green amorphous; UV λ_max_ (MeOH) nm (log ε): 210 (3.72), 270 (4.03), 315 (3.95), 355 (3.49), 365 (3.44); IR (ATR) ν_max_: 3384, 2919, 2850, 1625, 1559, 1508, 1458, 1420, 1325, 1286, 1246, 1228, 1196, 1162, 1089, 1029 cm^−1^; ^1^H and ^13^C-NMR data (DMSO-*d*_6_): see Table 3; HR-ESI-MS *m*/*z* 390.1298 (calcd. for C_22_H_20_N_3_O_2_S, 390.1271).

Compound **7** (maeruabisindole B): pale green amorphous; UV λ_max_ (MeOH) nm (log ε): 210 (4.47), 230 (4.15), 310 (3.93), 340 (3.31), 355 (3.31); IR (ATR) ν_max_: 3396, 2921, 2851, 1602, 1465, 1377, 1258, 1172, 1117, 1025 cm^−1^; ^1^H and ^13^C-NMR data (CD_3_OD): see Table 3; HR-ESI-MS *m*/*z* 406.1224 (calcd. for C_22_H_20_N_3_O_3_S, 406.1220).

Compound **8** (maeruabisindole C): dark green amorphous; UV λ_max_ (MeOH) nm (log ε): 210 (4.07), 285(2.93), 355(2.21), 365(2.36); IR (ATR) ν_max_: 3359, 3192, 2921, 2851, 2212, 1658, 1632, 1468, 1412, 1279, 1135, 702, 632 cm^−1^; ^1^H and ^13^C-NMR data (acetone-*d*_6_): see Table 4; HR-ESI-MS *m*/*z* 326.0968 (calcd. for C_20_H_12_N_3_O_2_, 326.0935).

### 3.4. Computational Detail

For the theoretical ECD spectra of maeruanitrile A (**1**), the possible configurations were computed at a B3LYP/6-31G(d,p) level. The ECD spectra were calculated using the time-dependent density functional theory (TD-DFT) method with a B3LYP functional and 6-311++G(d,p) basis set. The geometry optimization and TD-DFT calculations were both performed with a polarizable continuum model (PCM) solvation model using methanol (MeOH). The rotary strengths of 70 excited states were calculated. All calculations were performed using the Gaussian16 program package [28]. The ECD spectra were simulated with overlapping Gaussian functions with a σ = 0.20 eV fitting parameter using the SpecDis1.64 program [29]. The more reliable length gauge representation was used for the ECD spectra.

In addition, conformational structures of compounds **2**–**7** were calculated at a B3LYP/6-31G(d,p) level of theory to identify the lowest energy conformation of these compounds. The polarizable continuum model (PCM) solvent models, with dimethyl sulfoxide (DMSO) for maeroximes A-C (**3–5**), and maeruabisindole A (**6**) and MeOH for maeruanitrile B (**2**), and maeruabisindole B (**7**) were performed.

### 3.5. Inhibitory Activity of NO Production

#### 3.5.1. Materials

Dimethyl sulfoxide (DMSO), dexamethasone, and lipopolysaccharides (LPS, *Escherichia coli* O26:B6) were purchased from Sigma-Aldrich (St. Louis, MO, USA). The material 3-(4,5-Dimethylthiazol-2-yl)-2,5-diphenyltetrazolium bromide (MTT) was purchased from Invitrogen, Thermo Fisher Scientific (Waltham, MA, USA). Dulbecco’s modified Eagle’s media (DMEM), fetal bovine serum (FBS), antibiotic-antimycotic, and trypsin-EDTA were obtained from Gibco, Thermo Fisher Scientific (Waltham, MA, USA).

#### 3.5.2. Cell Culture

Raw264.7 cells were purchased from ATCC (TIB-71). Cells were maintained in Dulbecco’s modified Eagle’s media (DMEM) supplemented with a 10% heat-inactivated fetal bovine serum (FBS) and 1% antibiotic-antimycotic. Cells were incubated at 37 °C in a humidified atmosphere containing 5% CO_2_.

#### 3.5.3. Preparation of Test Solutions

The compounds were dissolved in DMSO to prepare their stock solutions at a concentration of 50 mM. Then, they were pipetted to mix in the culture medium to make the maximum tested concentration of 200 µM containing 0.4% DMSO. Then, the 200 µM tested solution was further diluted with a culture medium, giving the concentration series of 200, 100, 50, and 25 μM (2-fold dilution). The working concentrations of the test compounds [25, 50, 100, and 200 μM] were used to treat cells.

#### 3.5.4. Stimulation of Inflammation in Raw264.7 Cells

Raw264.7 cells were seeded at a density of 5 × 10^4^ cells/well in a 96-well plate. The cells were pre-treated with various concentrations of samples for 24 h. Cells were induced with 100 ng/mL LPS for 24 h. The culture supernatant was collected for NO production analysis, and cells were further examined for their viability.

The cell viability was measured by the MTT assay [30]. An MTT solution (1 mg/mL) was added to each well and incubated for 4 h at 37 °C. Then, the MTT solution was removed, and the formazan production was dissolved with DMSO. The absorbance was measured at 570 nm using a microplate reader (BioTeK, Santa Clara, CA, USA).

#### 3.5.5. Measurement of NO Production

The NO production was measured using a Griess reagent kit (Invitrogen, Thermo Fisher Scientific, Waltham, MA, USA) [31]. Ninety µL of culture media were mixed with 10 µL of Griess reagent and incubated at room temperature for 30 min, and then the NO concentration was measured at 540 nm using the microplate reader. The percentage of NO production was calculated as Equation (1).

(1) $$\%\mathrm{NO}\;\mathrm{production}\;=\;\frac{A}{B}\;\times \;100$$

A: The concentration of the nitric oxide in the cells induced by LPS and with sample pre-treatment [LPS (+), sample (+)].

B: The concentration of nitric oxide in the cells induced by LPS without sample pre-treatment [LPS (+), sample (−)].

The NO inhibitory activity was expressed as half the maximum (IC_50_) of the inhibitory concentration calculated using GraphPad Prism 9.

#### 3.5.6. Statistical Analysis

The IC_50_ values were expressed as the mean ± standard deviation (SD) from at least three independent experiments. The mean differences of the compounds vs. indomethacin (the positive control) were evaluated by one-way analysis of variance (ANOVA) with Tukey’s multiple comparison test (GraphPad Prism 9.3.1 software, San Diego, CA, USA). Statistical significance was defined as *p* < 0.05, *p* < 0.005, *p* < 0.001, and *p* < 0.0001.

## 4. Conclusions

Eight new indole alkaloids were isolated from the roots of *Maerua siamensis* (Capparaceae). Their structures were elucidated based on spectroscopic methods and computational analysis. Among them, maeruanitriles A (**1**) and B (**2**), maeroxime C (**5**), and maeruabisindoles B (**7**) and C (**8**) inhibited nitric oxide production in LPS-induced RAW 264.7 cells. This finding supports the traditional use of *M. siamensis* roots for analgesic and anti-inflammatory purposes in traditional Thai medicine.

## Figures and Tables

**Table 1:** ^1^H- and ^13^C-NMR data for compounds **1**–**2**.

Position	1		2	
	δ_H_, Multiplicity (J in Hz) ^a^	δ_C_	δ_H_, Multiplicity (J in Hz) ^b^	δ_C_
NH-1	9.52, br s			
2		178.2		132.5
3		73.8		110.8
3a		122.8		121.4
4	7.48, d (8.4)	126.9	7.63, d (9.0)	121.4
5	6.63, dd (8.4, 2.4)	108.4	6.87, dd (9.0, 2.4)	113.4
6		163.4		160.6
7	6.53, d (2.4)	98.8	6.98, d (2.4)	95.3
7a		144.7		140.3
8a 8b	3.09, d (16.8) 2.89, d (16.8)	28.0	4.18, d (18.0) 4.13, d (18.0)	13.0
9		117.7		118.9
2-SOCH_3_			2.16, s	40.4
3-OH	5.44, s			
6-OCH_3_	3.81, s	56.5	3.86, s	55.9

^a 1^H- (600 MHz) and ^13^C-NMR (150 MHz) in acetone-*d*_6_; ppm. ^b 1^H- (600 MHz) and ^13^C-NMR (150 MHz) in CD_3_OD; ppm.

**Table 2:** ^1^H- and ^13^C-NMR data for compounds **3**–**5**.

Position	3		4		5	
	δ_H_, Multiplicity (J in Hz) ^a^	δ_C_	δ_H_, Multiplicity (J in Hz) ^a^	δ_C_	δ_H_, Multiplicity (J in Hz) ^a^	δ_C_
NH-1	10.72, br s		12.10, br s		10.63, br s	
2	7.03, d (2.0)	123.1	7.97, s	136.7		168.6
3		107.8		113.6		131.1
3a		121.5		118.5		113.3
4	7.33, d (8.8)	118.9	7.92, d (8.4)	121.4	7.87, d (8.4)	126.5
5	6.64, dd (8.8, 2.4)	108.8	6.89, dd (8.4)	112.3	6.52, dd (8.4, 2.4)	106.8
6		155.6		156.9		162.2
7	6.84, d (2.4)	94.5	7.00, s	95.8	6.41, d (2.4)	96.6
7a		136.8		138.0		145.6
8	3.80, s	25.9		182.5	6.80, s	119.9
9		157.8		155.9		150.8
6-OCH_3_	3.74, s	55.2	3.79, s	55.3	3.78, s	55.5
SCH_3_	2.17, s	12.5	2.43, s	12.7	2.35, s	12.6
N-OCH_3_	3.86, s	61.5	3.73, s	61.9	4.00, s	62.5

^a 1^H- (400 MHz) and ^13^C-NMR (100 MHz) in DMSO-*d*_6_; ppm.

**Table 3:** ^1^H- and ^13^C-NMR data for compounds **6**–**7.**

Position	6		7	
	δ_H_, Multiplicity (J in Hz) ^a^	δ_C_	δ_H_, Multiplicity (J in Hz) ^b^	δ_C_
2-azete		144.8		151.7
3-azete		133.4		137.8
4-azete		139.5		142.7
NH-1′	10.71, br s			
2′		129.3		130.8
3′	7.79, br s	108.5	8.43, s	109.3
3′a		114.1		116.3
4′	8.05, d (8.4)	122.6	8.12, d (8.4)	123.7
5′	6.78, dd (8.4, 2.0)	108.7	6.93, dd (8.4, 2.0)	111.8
6′		160.0		163.0
7′	6.99, d (2.4)	94.8	7.05, d (2.0)	95.8
7′a		142.9		144.9
NH-1″	11.52, br s			
2″	7.51, d (2.0)	125.0	7.54, s	126.1
3″		113.0		113.2
3″a		116.4		118.2
4″		153.9		155.6
5″	6.56, dd (6.0, 2.4)	100.4	6.60, d (6.8)	101.7
6″	7.10, overlapped	122.5	7.16, dd (7.6, 6.8)	124.4
7″	7.12, overlapped	105.0	7.14, d (6.8)	106.3
7″a		137.9		140.0
2-SCH_3_	2.59, s	14.0		
2-SOCH_3_			2.98, s	42.1
6′-OCH_3_	3.81, s	55.2	3.89, s	56.1
4″-OCH_3_	3.52, s	54.9	3.55, s	55.7

^a 1^H- (400 MHz) and ^13^C-NMR (100 MHz) in DMSO-*d*_6_; ppm. ^b 1^H- (400 MHz) and ^13^C-NMR (100 MHz) in CD_3_OD; ppm.

**Table 4:** ^1^H- and ^13^C-NMR data for compound 8.

8					
Position	δ_H_, Multiplicity (J in Hz) ^a^	δ_C_	Position	δ_H_, Multiplicity (J in Hz) ^a^	δ_C_
1		157.1	9		158.8
2	6.80, d (8.0)	101.5	10	7.02, d (2.4)	97.5
3	7.40, t (8.0)	128.2	10a		144.6
4	7.24, d (8.0)	105.1	NH-11	10.39, br s	
4a		143.4	11a		135.8
NH-5	10.86, br s		12	8.53, s	110.2
5a		138.0	12a		122.0
6		82.5	12b		113.0
6a		123.0	1-OCH_3_	4.12, s	56.0
6b		115.4	6-CN		118.3
7	8.33, d (8.8)	122.6	9-OH	8.70, br s	
8	6.86, dd (8.8, 2.4)	110.0			

^a 1^H- (400 MHz) and ^13^C-NMR (100 MHz) in acetone-*d*_6_; ppm.

**Table 5:** Inhibition concentration of isolated compounds on nitric oxide (NO) production and cytotoxicity in LPS-induced RAW 264.7 cells.

Compound	IC_50_ of NO Inhibition (μM) ^a^	Cytotoxicity (μM) ^c^
**1**	186.4 ± 22.5	>200
**2**	186.8 ± 23.1	>200
**3**	n.d. ^b^	toxicity at 100
**4**	>200 (231.2 ± 20.1 ***)	>200
**5**	92.2 ± 8.9 **	>200
**6**	n.d. ^b^	toxicity at 100
**7**	31.1 ± 1.8 ****	toxicity at 100
**8**	56.7 ± 3.8 ****	>200
indomethacin	150.0 ± 16.0	>200

** *p* < 0.005, *** *p* < 0.001 and **** *p* < 0.0001 versus indomethacin (positive control). ^a^ The IC_50_ of NO inhibition was expressed as Mean ± SD from three independent experiments. ^b^ n.d. refers to “not determined”. The compound could not be determined for IC_50_ value due to its cytotoxicity. ^c^ The maximum concentration of test compounds was 200 μM. Cytotoxicity was indicated by the concentration given that cell viability was lower than 80%. The tested concentration that exhibited cell viability below 80% was noted as “toxicity at that concentration”.

## Data Availability

All data are present in the article and supplementary data.

## Supplementary Materials

The following supplementary information can be downloaded at: https://www.mdpi.com/1420-3049/27/21/7558/s1, Supplementary materials A: Structure elucidation and NO inhibition (Figures S1–S98); Supplementary materials B: Computational studies (pp. 1–9).

## Sample Availability

Samples of the compounds are not available from the authors.
