# Correction: Nukulkit et al. Eight Indole Alkaloids from the Roots of *Maerua siamensis* and Their Nitric Oxide Inhibitory Effects. *Molecules* 2022, *27*, 7558

**DOI:** 10.3390/molecules29071526

**Published:** 2024-03-29

**Authors:** Sasiwimon Nukulkit, Angkana Jantimaporn, Preeyaporn Poldorn, Mattaka Khongkow, Thanyada Rungrotmongkol, Hsun-Shuo Chang, Rutt Suttisri, Chaisak Chansriniyom

**Affiliations:** 1 Department of Pharmacognosy and Pharmaceutical Botany, Faculty of Pharmaceutical Sciences, Chulalongkorn University, Bangkok 10330, Thailand; 2 Natural Products and Nanoparticles Research Unit, Chulalongkorn University, Bangkok 10330, Thailand; 3 National Nanotechnology Center (NANOTEC), National Science and Technology Development Agency, Pathum Thani 12120, Thailand; 4 Center of Excellence in Biocatalyst and Sustainable Biotechnology, Department of Biochemistry, Faculty of Science, Chulalongkorn University, Bangkok 10330, Thailand; 5 Program in Bioinformatics and Computational Biology, Graduate School, Chulalongkorn University, Bangkok 10330, Thailand; 6 School of Pharmacy, College of Pharmacy, Kaohsiung Medical University, Kaohsiung 807, Taiwan

## Error in Table

After a proofreading check, some experimental data were inconsistent with the supplementary information in the original publication [1]. Firstly, the carbon at position 9 of maeruabisindole C (compound **8**) was a quaternary carbon, as evidenced by Figures S90 and S91 (the HSQC and HMBC spectra, respectively, of compound **8**). Thus, the δ_H_ regarding position 9 in Table 4 should be deleted. The correct version of Table 4 is given below.

## Text Correction

Secondly, in Section 3.3, the IR absorption peaks should be corrected to 3359, 3192, 2921, 2851, 2212, 1658, 1632, 1468, 1412, 1279, 1135, 702, and 632 cm^−1^, consistent with the IR spectrum of compound **8** (Figure S85), and the theoretical *m*/*z* of [M-H]^−^ ion of compound 8 (calcd. for C_20_H_12_N_3_O_2_) should be corrected to 326.0935.

Compound **8** (maeruabisindole C): dark green amorphous; UV λ_max_ (MeOH) nm (log ε): 210 (4.07), 285(2.93), 355(2.21), 365(2.36); IR (ATR) ν_max_: 3359, 3192, 2921, 2851, 2212, 1658, 1632, 1468, 1412, 1279, 1135, 702, 632 cm^−1^; ^1^H and ^13^C-NMR data (acetone-*d*_6_): see Table 4; HR-ESI-MS m/z 326.0968 (calcd. for C_20_H_12_N_3_O_2_, 326.0935).

The authors apologize for any inconvenience caused and state that the scientific conclusions are unaffected. This correction was approved by the Academic Editor. The original publication has also been updated.

## Figures and Tables

**Table 4:** ^1^H- and ^13^C-NMR data for compound 8.

8					
Position	δ_H_, Multiplicity (J in Hz) ^a^	δ_C_	Position	δ_H_, Multiplicity (J in Hz) ^a^	δ_C_
1		157.1	9		158.8
2	6.80, d (8.0)	101.5	10	7.02, d (2.4)	97.5
3	7.40, t (8.0)	128.2	10a		144.6
4	7.24, d (8.0)	105.1	NH-11	10.39, br s	
4a		143.4	11a		135.8
NH-5	10.86, br s		12	8.53, s	110.2
5a		138.0	12a		122.0
6		82.5	12b		113.0
6a		123.0	1-OCH_3_	4.12, s	56.0
6b		115.4	6-CN		118.3
7	8.33, d (8.8)	122.6	9-OH	8.70, br s	
8	6.86, dd (8.8, 2.4)	110.0			

^a 1^H- (400 MHz) and ^13^C-NMR (100 MHz) in acetone-*d*_6_; ppm.
