# Supplementary material for: Eight Indole Alkaloids from the Roots of Maerua siamensis and Their Nitric Oxide Inhibitory Effects
Source: Molecules. 2022 Nov 4;27(21):7558. doi: 10.3390/molecules27217558 (PMC9656735; doi:10.3390/molecules27217558)
Supplement: Supplementary file 1 [file molecules-27-07558-s001.zip › molecules-1966267-supplementary materials.pdf]

Supplementary materials A: Structure elucidation and NO inhibition

Eight indole alkaloids from the roots of *Maerua siamensis* and their nitric oxide inhibitory effects

Sasiwimon Nukulkit [1, 2], Angkana Jantimaporn [3], Preeyaporn Poldorn [4], Mattaka Khongkow [3], Thanyada Rungrotmongkol [4, 5]

Hsun-Shuo Chang [6], Rutt Suttisri [1], Chaisak Chansriniyom [1, 2,*]

[1] Department of Pharmacognosy and Pharmaceutical Botany, Faculty of Pharmaceutical Sciences, Chulalongkorn University, Bangkok 10330, Thailand
[2] Natural Products and Nanoparticles Research Unit, Chulalongkorn University, Bangkok 10330, Thailand.
[3] National Nanotechnology Center (NANOTEC), National Science and Technology Development Agency, Pathum Thani 12120, Thailand.
[4] Center of Excellence in Biocatalyst and Sustainable Biotechnology, Department of Biochemistry, Faculty of Science, Chulalongkorn University, Bangkok 10330, Thailand.
[5] Program in Bioinformatics and Computational Biology, Graduate School, Chulalongkorn University, Bangkok 10330, Thailand.
[6] School of Pharmacy, College of Pharmacy, Kaohsiung Medical University, Kaohsiung 807, Taiwan.

***** Correspondence: chaisak.ch@chula.ac.th; Tel.: +66-2218-8347

List of Contents

Contents **Page**

Compound **1**

Figure S1. The HR-ESI-MS spectrum of compound **1** 1

Figure S2. The UV spectrum of compound **1** 2

Figure S3. The IR spectrum of compound **1** (ATR) 3

Figure S4. The CD spectrum in MeOH of compound **1** 4

Figure S5. The ^{13}C-NMR, DEPT 135 and DEPT 90 (150 MHz, acetone-d_6) spectra of compound **1** 5

Figure S6. The ^{1}H NMR (600 MHz, acetone-d_6) spectrum of compound **1** 6

Figure S7. The ^{13}C NMR (150 MHz, acetone-d_6) spectrum of compound **1** 7

Figure S8. The HSQC spectrum of compound **1** 8

Figure S9. The HMBC spectrum of compound **1** 9

Figure S10. The ^{1}H-^{1}H COSY spectrum of compound **1** 10

Figure S11. The NOESY spectrum of compound **1** 11

Compound **2**

Figure S12. The HR-ESI-MS spectrum of compound **2** 12

Figure S13. The UV spectrum of compound **2** 13

Figure S14. The IR spectrum of compound **2** (ATR) 14

Figure S15. The ^{13}C-NMR, DEPT 135 and DEPT 90 (150 MHz, CD_3OD) spectra of compound **2** 15

Figure S16. The ^{1}H NMR (600 MHz, CD_3OD) spectrum of compound **2** 16

Figure S17. The ^{13}C NMR (150 MHz, CD_3OD) spectrum of compound **2** 17

Figure S18. The ^{13}C NMR (150 MHz, CD_3OD) spectrum of compound **2 (continued)** 18

Figure S19. The HSQC spectrum of compound **2** 19

Figure S20. The HMBC spectrum of compound **2** 20

Figure S21. The HMBC spectrum of compound **2 (continued)** 21

Figure S22. The ^{1}H-^{1}H COSY spectrum of compound **2** 22

Figure S23. The NOESY spectrum of compound **2** 23

Compound **3**

Figure S24. The HR-ESI-MS spectrum of compound **3** 24

Figure S25. The UV spectrum of compound **3** 25

Figure S26. The IR spectrum of compound **3** (ATR) 26

Figure S27. The ^{13}C-NMR, DEPT 135 and DEPT 90 (100 MHz, DMSO-d_6) spectra of compound **3** 27

Figure S28. The ^{1}H NMR (400 MHz, DMSO-d_6) spectrum of compound **3** 28

Figure S29. The ^{13}C NMR (100 MH, DMSO-d_6) spectrum of compound **3** 29

Figure S30. The HSQC spectrum of compound **3** 30

Figure S31. The HMBC spectrum of compound **3** 31

Figure S32. The HMBC spectrum of compound **3** (**continued**) 32

Figure S33. The ^{1}H-^{1}H COSY spectrum of compound **3** 33

Figure S34. The NOESY spectrum of compound **3** 34

Figure S35. The NOESY spectrum of compound **3 (continued)** 35

Compound **4**

Figure S36. The HR-ESI-MS spectrum of compound **4** 36

Figure S37. The UV spectrum of compound **4** 37

Figure S38. The IR spectrum of compound **4** (ATR) 38

Figure S39. The ^{1}H NMR (400 MHz, DMSO-d_6) spectrum of compound **4** 39

Figure S40. The ^{13}C NMR (100 MHz, DMSO-d_6) spectrum of compound **4** 40

Figure S41. The HSQC spectrum of compound **4** 41

Figure S42. The HMBC spectrum of compound **4** 42

Figure S43. The HMBC spectrum of compound **4 (continued)** 43

Figure S44. The ^{1}H-^{1}H COSY spectrum of compound **4** 44

Figure S45. The NOESY spectrum of compound **4** 45

Figure S46. The NOESY spectrum of compound **4** (**continued**) 46

Compound **5**

Figure S47. The HR-ESI-MS spectrum of compound **5** 47

Figure S48. The UV spectrum of compound **5** 48

Figure S49. The IR spectrum of compound **5** (ATR) 49

Figure S50. The ^{1}H NMR (400 MHz, DMSO-d_6) spectrum of compound **5** 50

Figure S51. The ^{13}C NMR (100 MHz, DMSO-d_6) spectrum of compound **5** 51

Figure S52. The HSQC spectrum of compound **5** 52

Figure S53. The HMBC spectrum of compound **5** 53

Figure S54. The ^{1}H-^{1}H COSY spectrum of compound **5** 54

Figure S55. The NOESY spectrum of compound **5** 55

Compound **6**

Figure S56. The HR-ESI-MS spectrum of compound **6** 56

Figure S57. The UV spectrum of compound **6** 57

Figure S58. The IR spectrum of compound **6** (ATR) 58

Figure S59. The ^{1}H NMR (400 MHz, DMSO-d_6) spectrum of compound **6** 59

Figure S60. The ^{1}H NMR (400 MHz, DMSO-d_6) spectrum of compound **6** (**continued**) 60

Figure S61. The ^{13}C NMR (100 MHz, DMSO-d_6) spectrum of compound **6** 61

Figure S62. The ^{13}C NMR (100 MHz, DMSO-d_6) spectrum of compound **6** (**continued**) 62

Figure S63. The HSQC spectrum of compound **6** 63

Figure S64. The HMBC spectrum of compound **6** 64

Figure S65. The HMBC spectrum of compound **6 (continued)** 65

Figure S66. The HMBC spectrum of compound **6 (continued)** 66

Figure S67. The HMBC spectrum of compound **6 (continued)** 67

Figure S68. The ^{1}H-^{1}H COSY spectrum of compound **6** 68

Figure S69. The NOESY spectrum of compound **6** 69

Figure S70. The NOESY spectrum of compound **6 (continued)** 70

Compound 7

Figure S71. The HR-ESI-MS spectrum of compound **7** 71

Figure S72. The UV spectrum of compound **7** 72

Figure S73. The IR spectrum of compound **7** (ATR) 73

Figure S74. The ^{1}H NMR (400 MHz, CD_3OD) spectrum of compound **7** 74

Figure S75. The ^{1}H NMR (400 MHz, CD_3OD) spectrum of compound **7 (continued)** 75

Figure S76. The ^{13}C NMR (100 MHz, CD_3OD) spectrum of compound **7** 76

Figure S77. The HSQC spectrum of compound **7** 77

Figure S78. The HSQC spectrum of compound **7 (continued)** 78

Figure S79. The HMBC spectrum of compound **7** 79

Figure S80. The HMBC spectrum of compound **7 (continued)** 80

Figure S81. The ^{1}H-^{1}H COSY spectrum of compound **7** 81

Figure S82. The NOESY spectrum of compound **7** 82

Compound 8

Figure S83. The HR-ESI-MS spectrum of compound **8** 83

Figure S84. The UV spectrum of compound **8** 84

Figure S85. The IR spectrum of compound **8** (ATR) 85

Figure S86. The ^{1}H NMR (400 MHz, acetone-d_6) spectrum of compound **8** 86

Figure S87. The ^{1}H NMR (400 MHz, acetone-d_6) spectrum of compound **8** 87

Figure S88. The ^{13}C NMR (100 MHz, acetone-d_6) spectrum of compound **8** 88

Figure S89. The ^{13}C NMR (100 MHz, acetone-d_6) spectrum of compound **8 (continued)** 89

Figure S90. The HSQC spectrum of compound **8** 90

Figure S91. The HMBC spectrum of compound **8** 91

Figure S92. The HMBC spectrum of compound **8 (continued)** 92

Figure S93. The HMBC spectrum of compound **8 (continued)** 93

Figure S94. The HMBC spectrum of compound **8 (continued)** 94

Figure S95. The ^{1}H-^{1}H COSY spectrum of compound **8** 95

Figure S96. The NOESY spectrum of compound **8** 96

NO inhibition

Figure S97. Effects of compounds isolated from *M. siamensis* roots against LPS-induced nitric oxide production in RAW 264.7 cells. 97

Figure S98. The IC_{50} values of compound isolated from *M. siamensis* roots against LPS-induced nitric oxide production in RAW 264.7 cells. 98

Mass Spectrum SmartFormula Report

Analysis Info

Analysis Name	D:\Data\f7\MSRE824B144_000002.d	11/14/2019 3:43:09 PM
Method	broadband first signal	Operator: YU HSIAO-CHING
Sample Name	MSRE-8-2-4B-14-4	Instrument: BRUKER FT-MS solariX
Comment	ESI Positive	

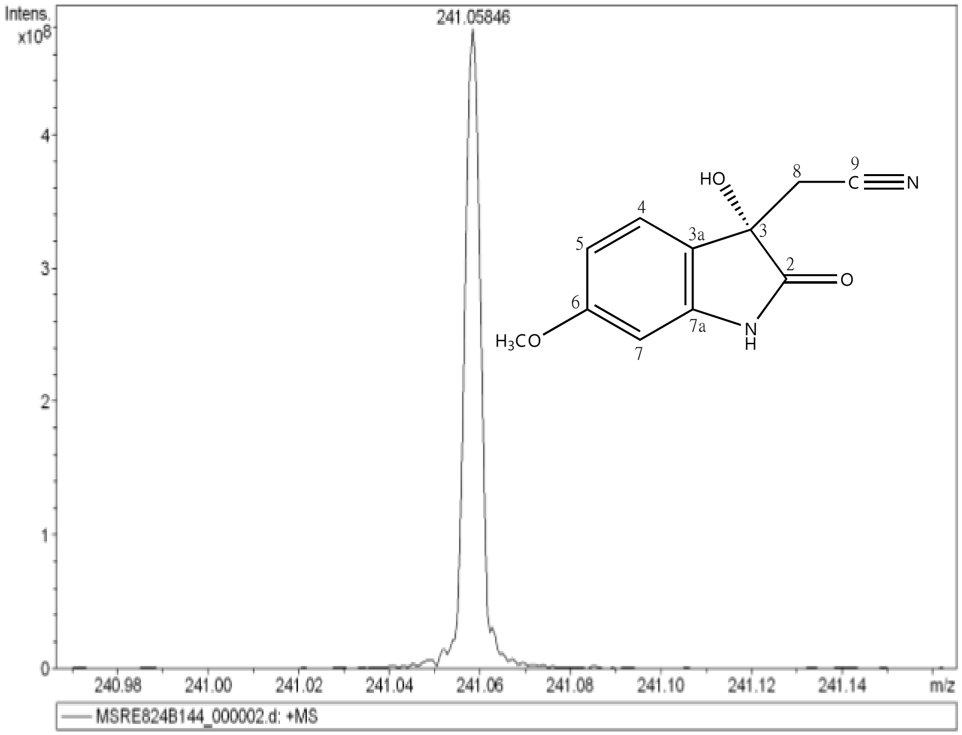

Meas. m/z	#	Formula	Score	m/z	err [mDa]	err [ppm]	mSigma	rdb	e⁻ Conf	N-Rule
241.05846	1	C 11 H 10 N 2 Na O 3	100.00	241.05836	-0.09	-0.39	9.4	7.5	even	ok

Figure S1. The HR-ESI-MS spectrum of compound **1**

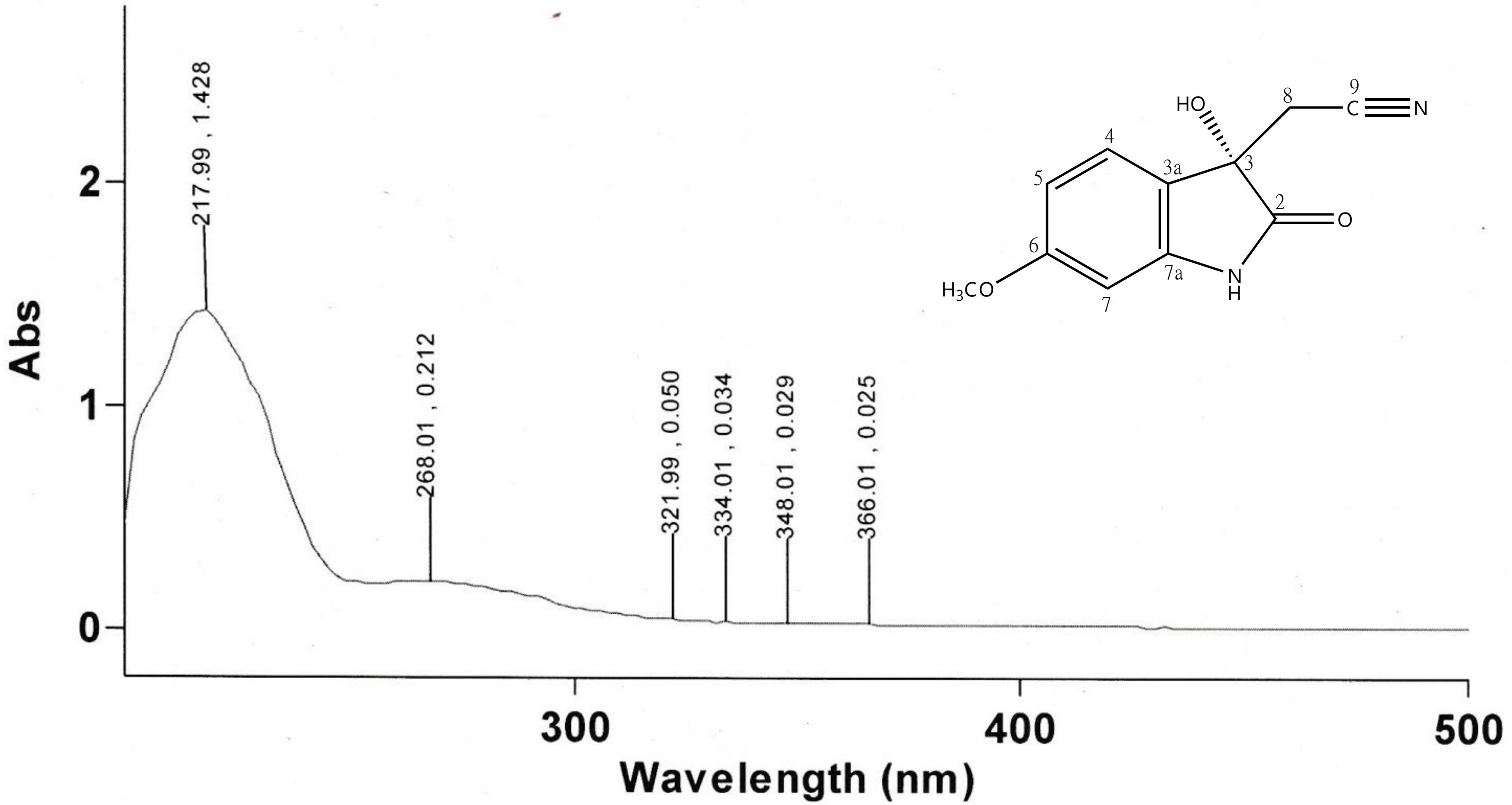

Figure S2. The UV spectrum of compound **1**

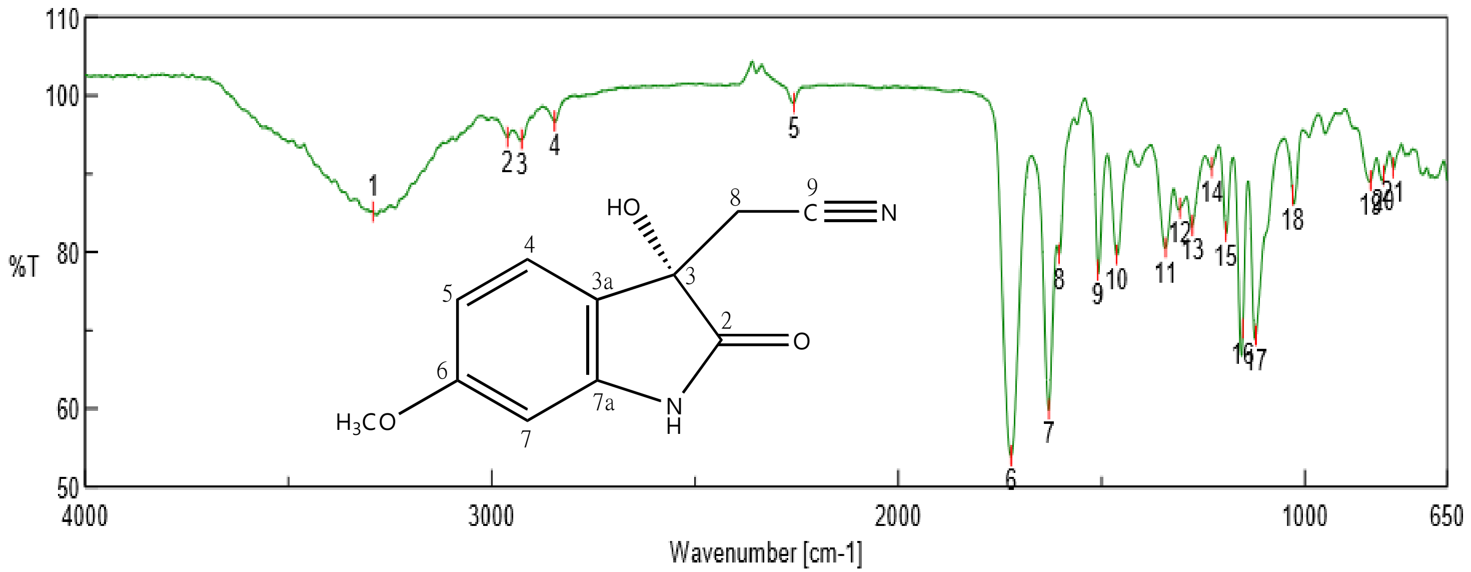

[Result of Peak Picking]

No.	Position	Intensity	No.	Position	Intensity	No.	Position	Intensity
1	3290.93	85.1235	2	2961.16	94.6663	3	2925.48	94.4219
4	2845.45	96.7096	5	2256.31	99.011	6	1722.12	53.9494
7	1629.55	59.8982	8	1604.48	79.7356	9	1509.03	77.612
10	1462.74	79.6136	11	1342.21	80.5442	12	1306.54	85.5827
13	1276.65	83.3796	14	1228.43	90.8438	15	1194.69	82.6032
16	1152.26	70.2385	17	1120.44	69.3525	18	1029.8	87.2652
19	837.919	89.1314	20	806.099	89.7656	21	782.958	90.7427

Figure S3. The IR spectrum of compound **1** (ATR)

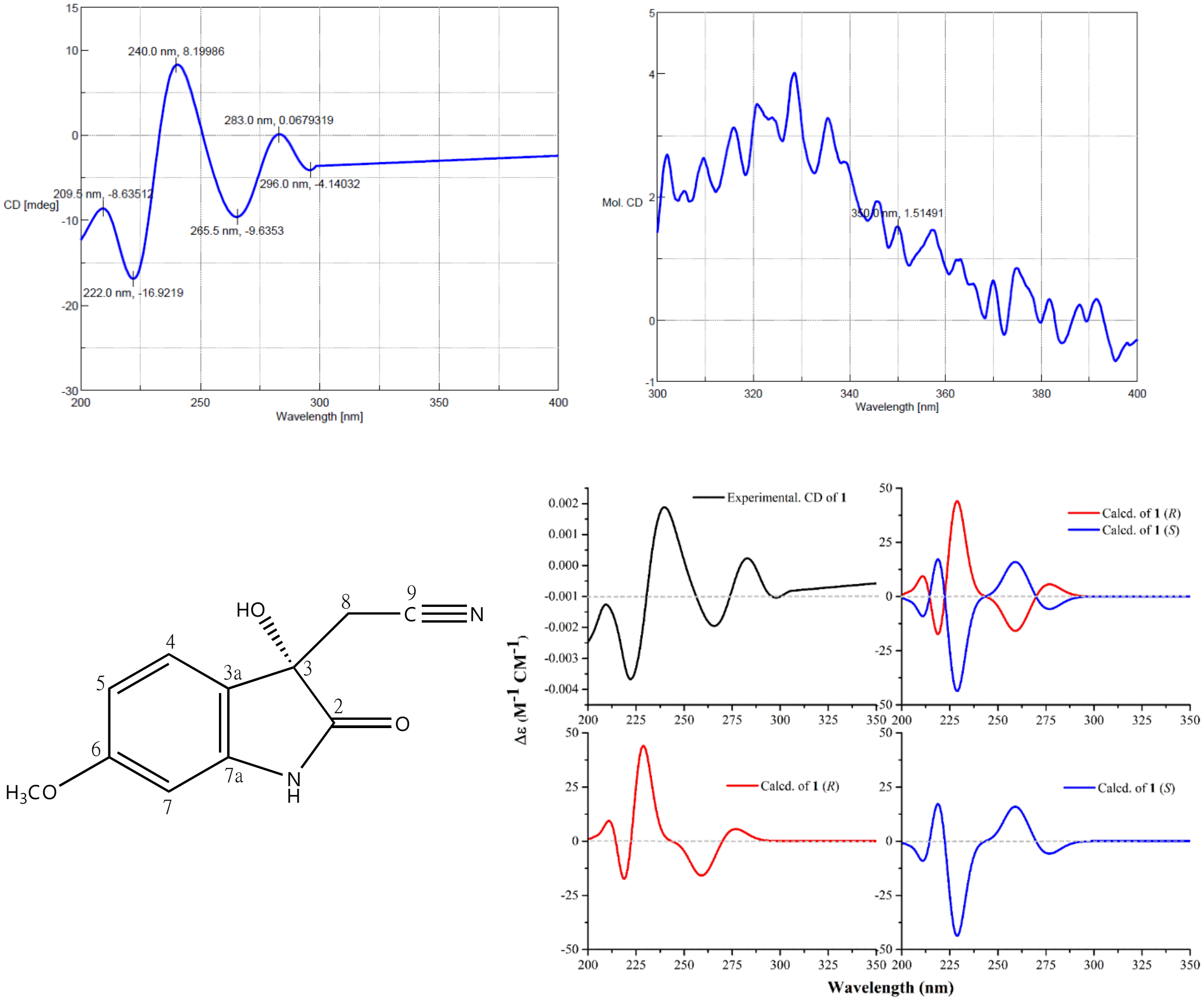

Figure S4. The CD spectrum in MeOH of compound **1** (upper left) and The Rh$_2$ (OCOCF$_3$)$_4$ Induced CD spectrum of **1** CH$_2$Cl$_2$ (upper right). ECD spectrum of **1** (lower right)

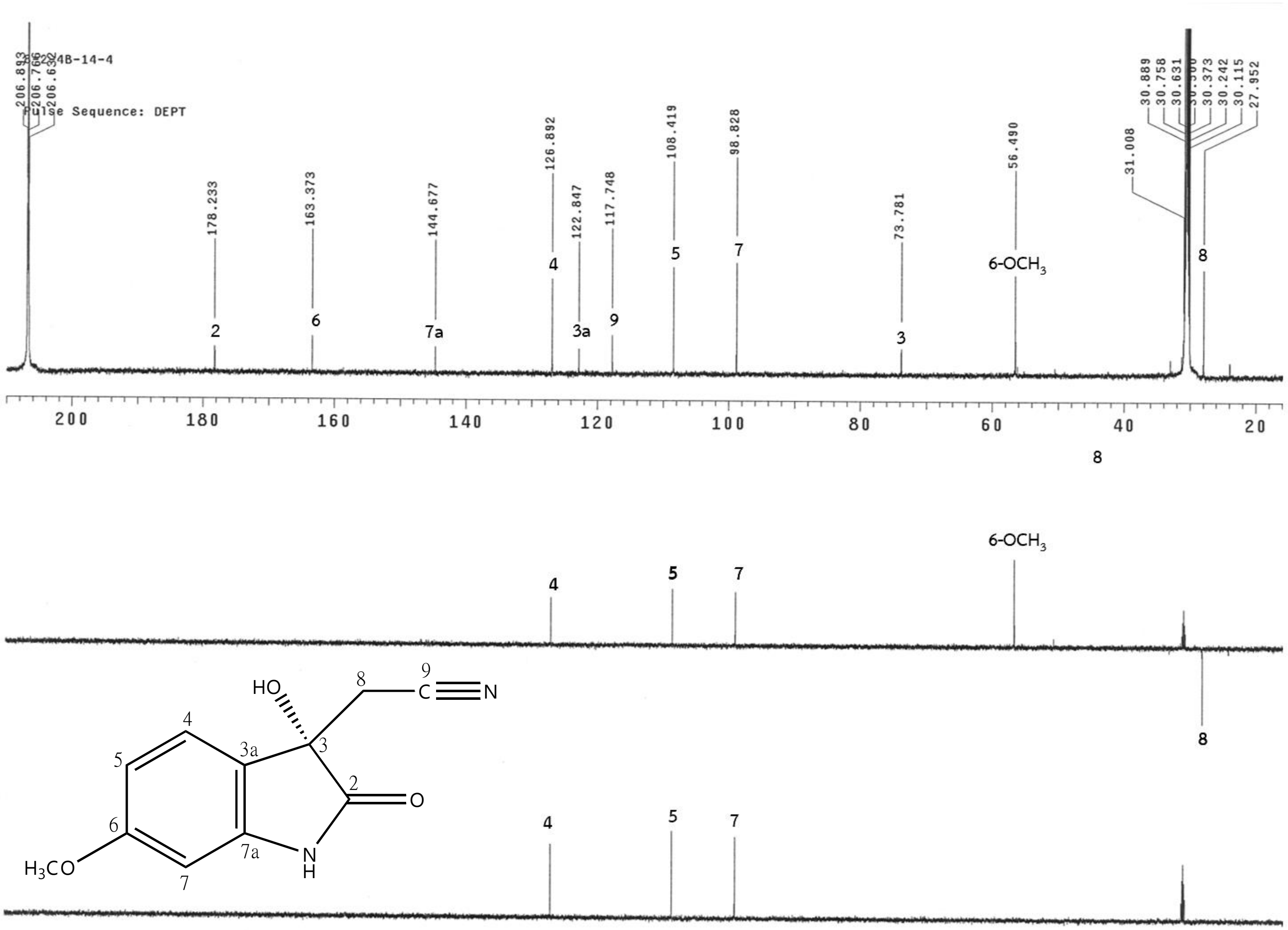

Figure S5. The ^{13}C-NMR, DEPT 135 and DEPT 90 (150 MHz, acetone-*d_6*) spectra of compound **1**

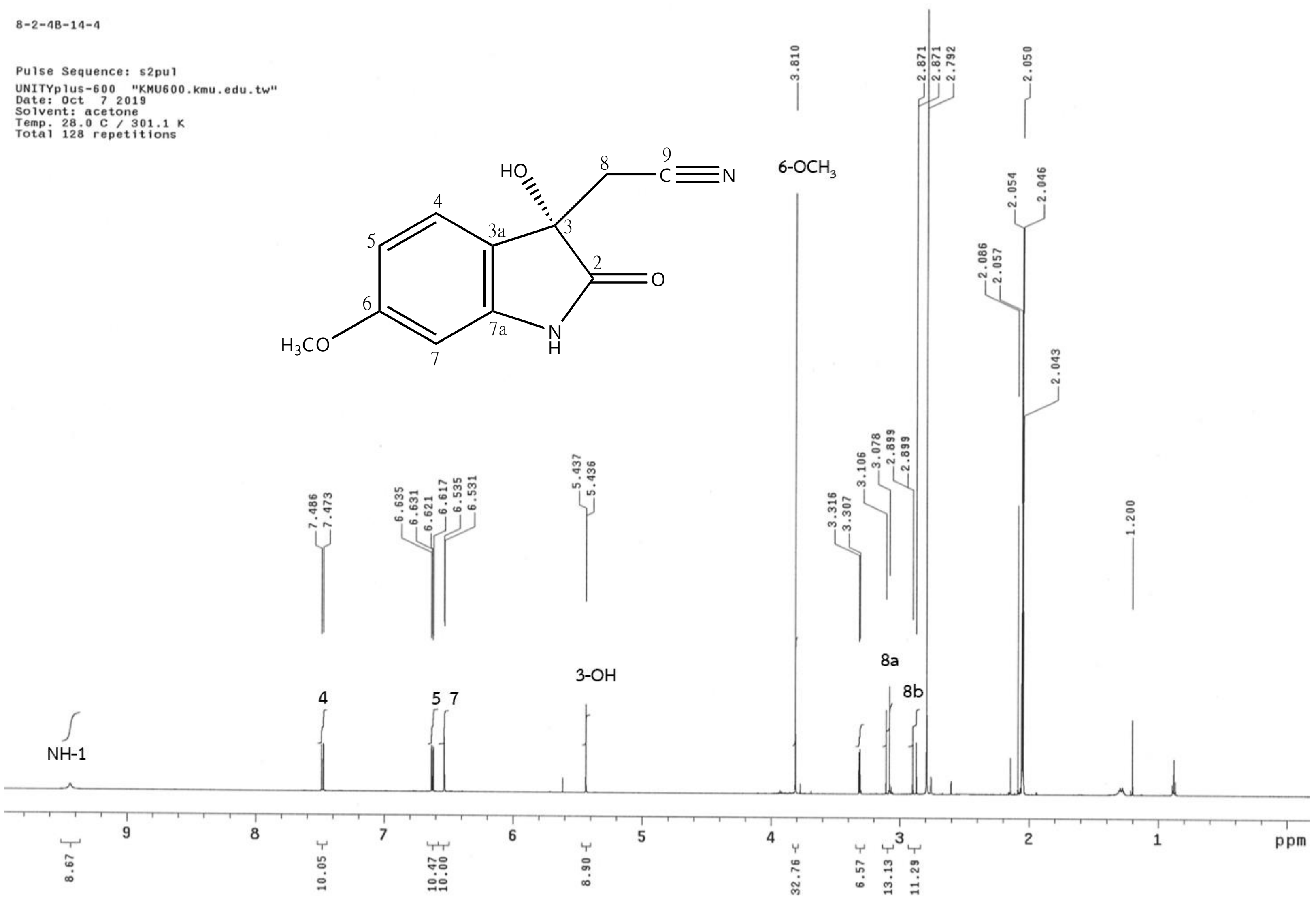

Figure S6. The ^{1}H NMR (600 MHz, acetone-d_6) spectrum of compound **1**

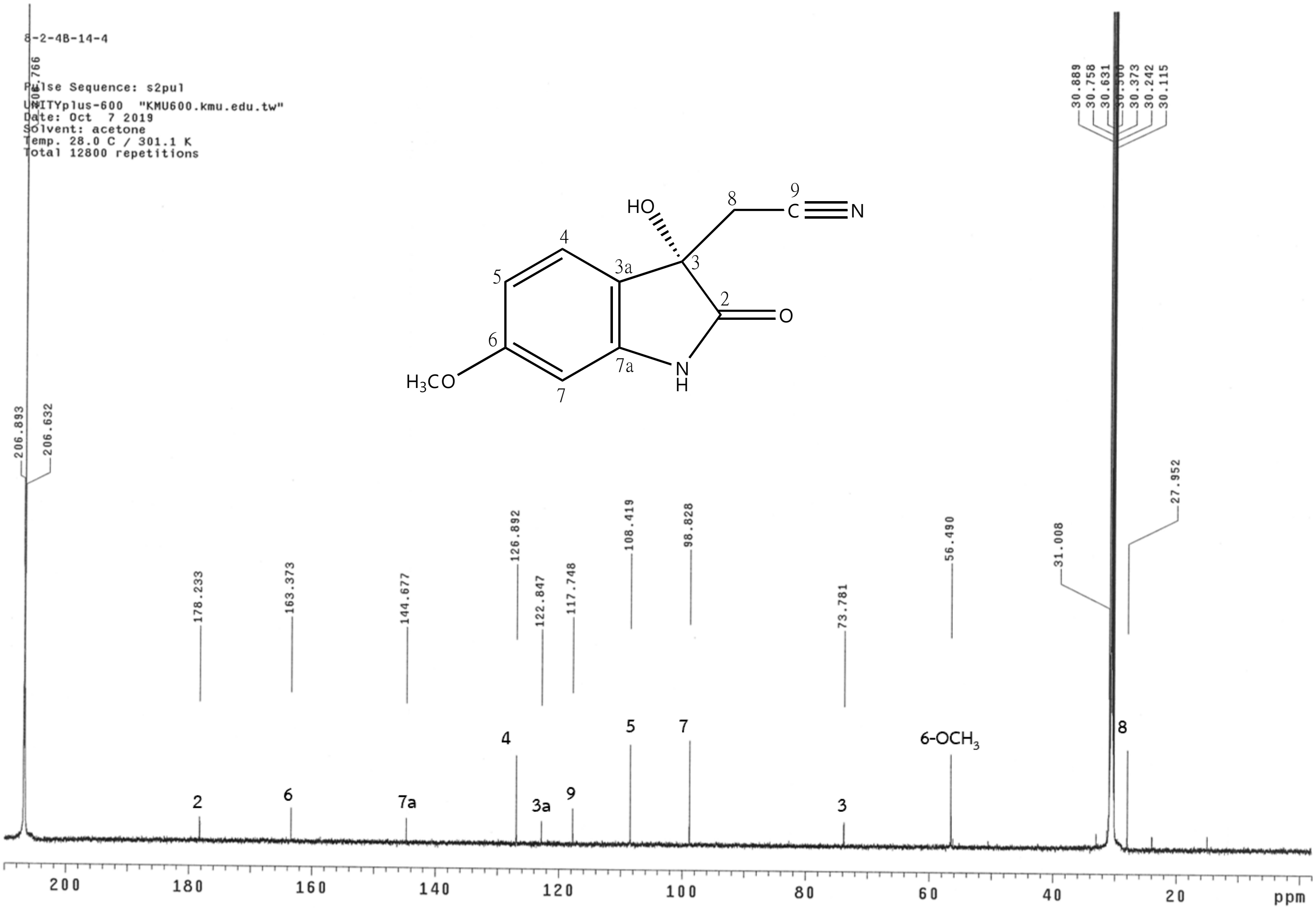

Figure S7. The ^{13}C-NMR (150 MHz, acetone-d_6) spectra of compound **1**

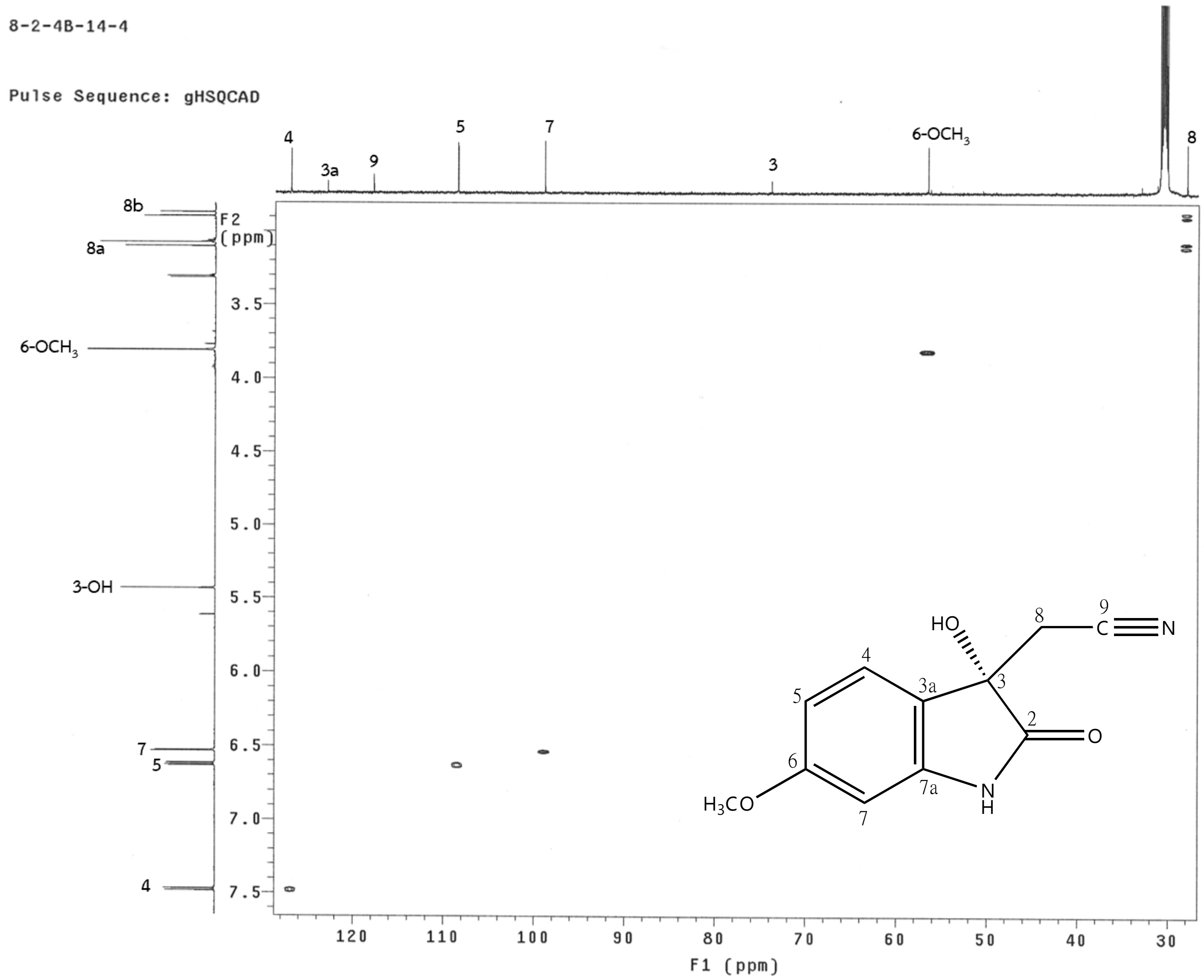

Figure S8. The HSQC spectrum of compound **1**

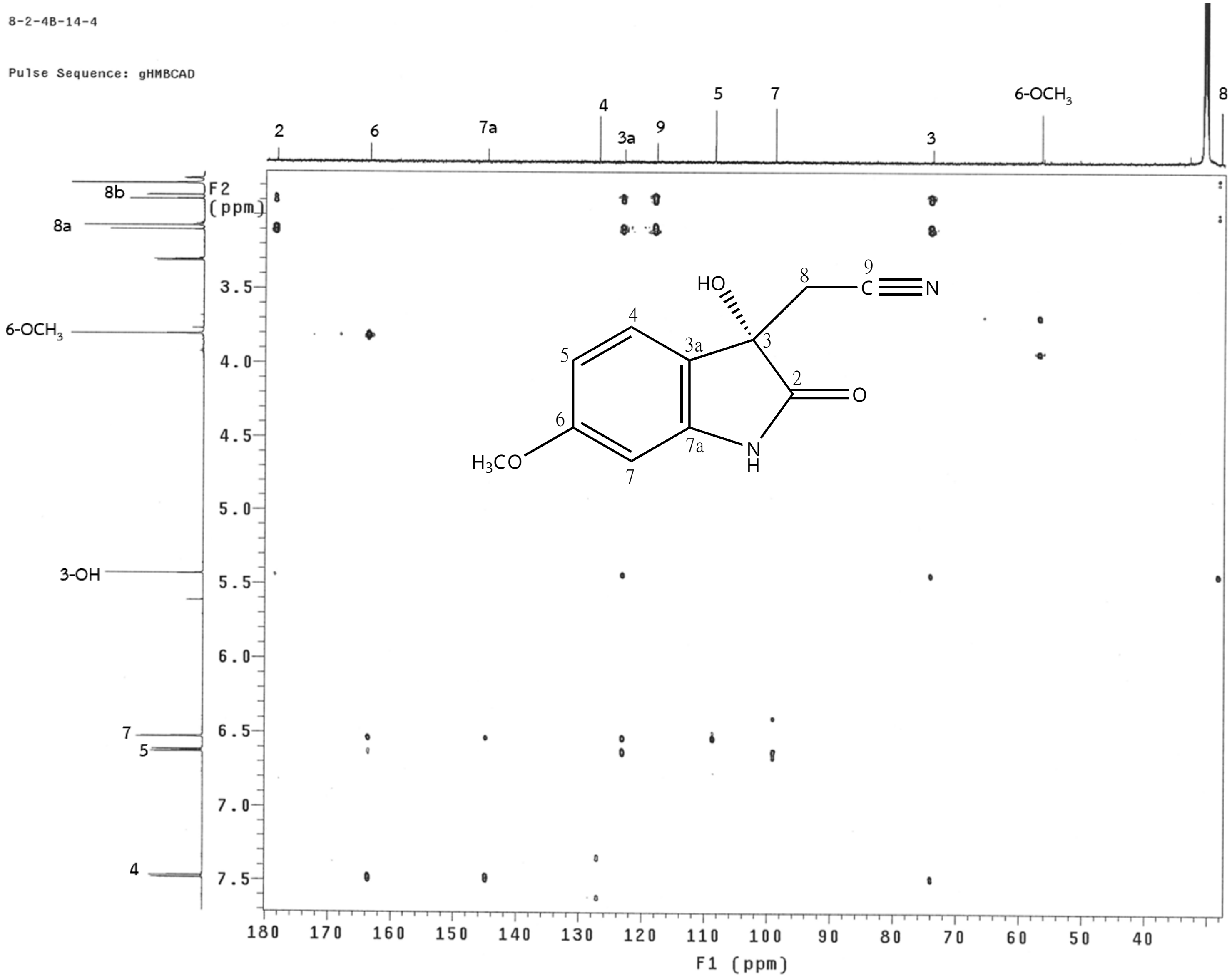

Figure S9. The HMBC spectrum of compound **1**

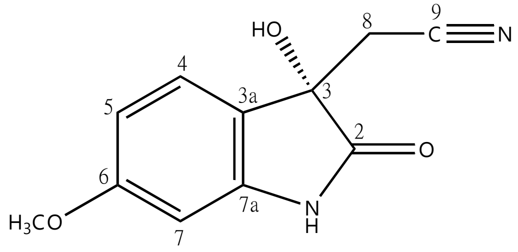

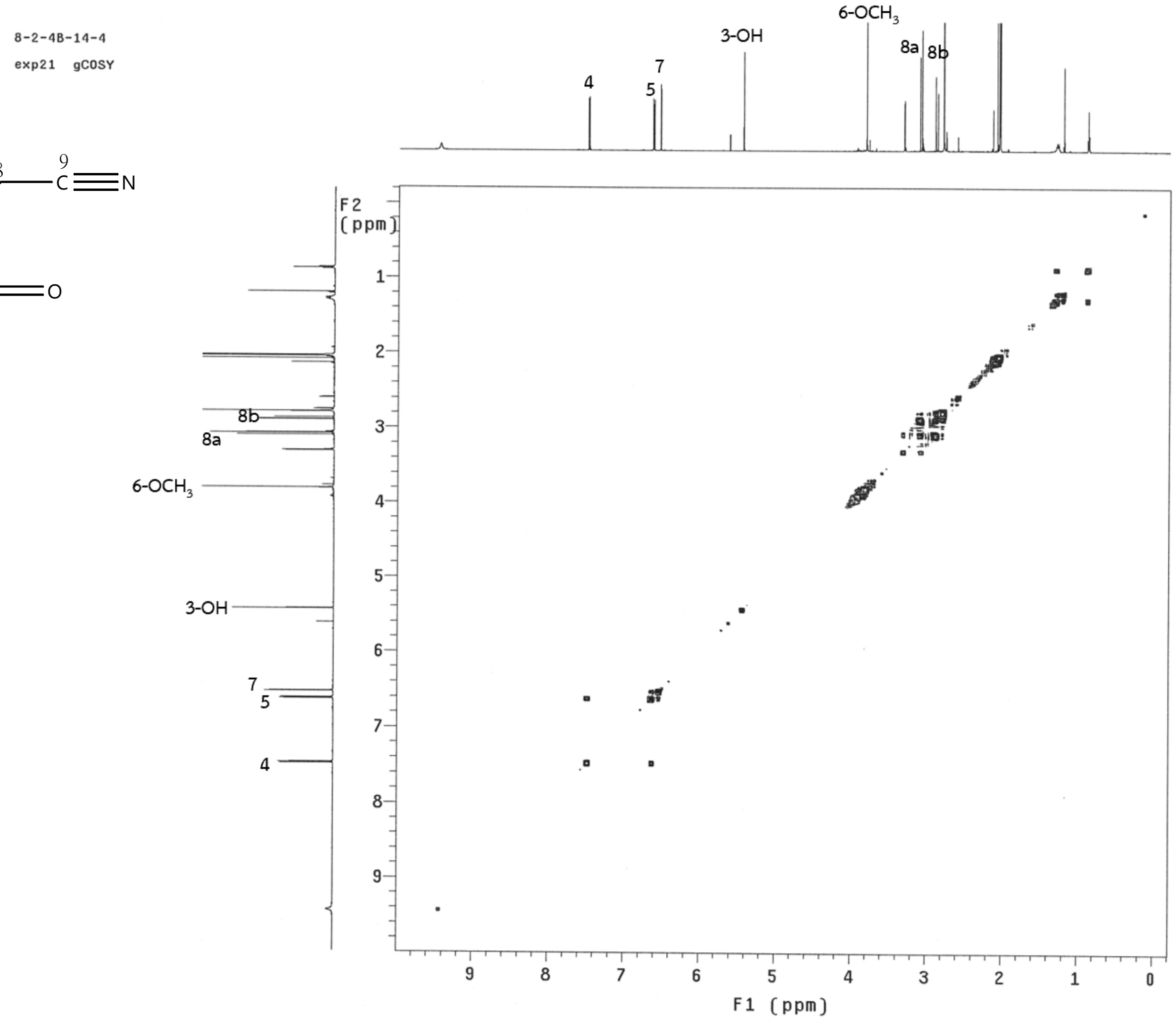

Figure S10. The ^{1}H-^{1}H COSY spectrum of compound **1**

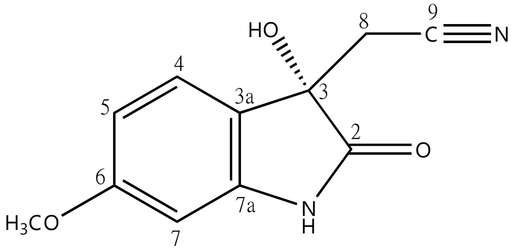

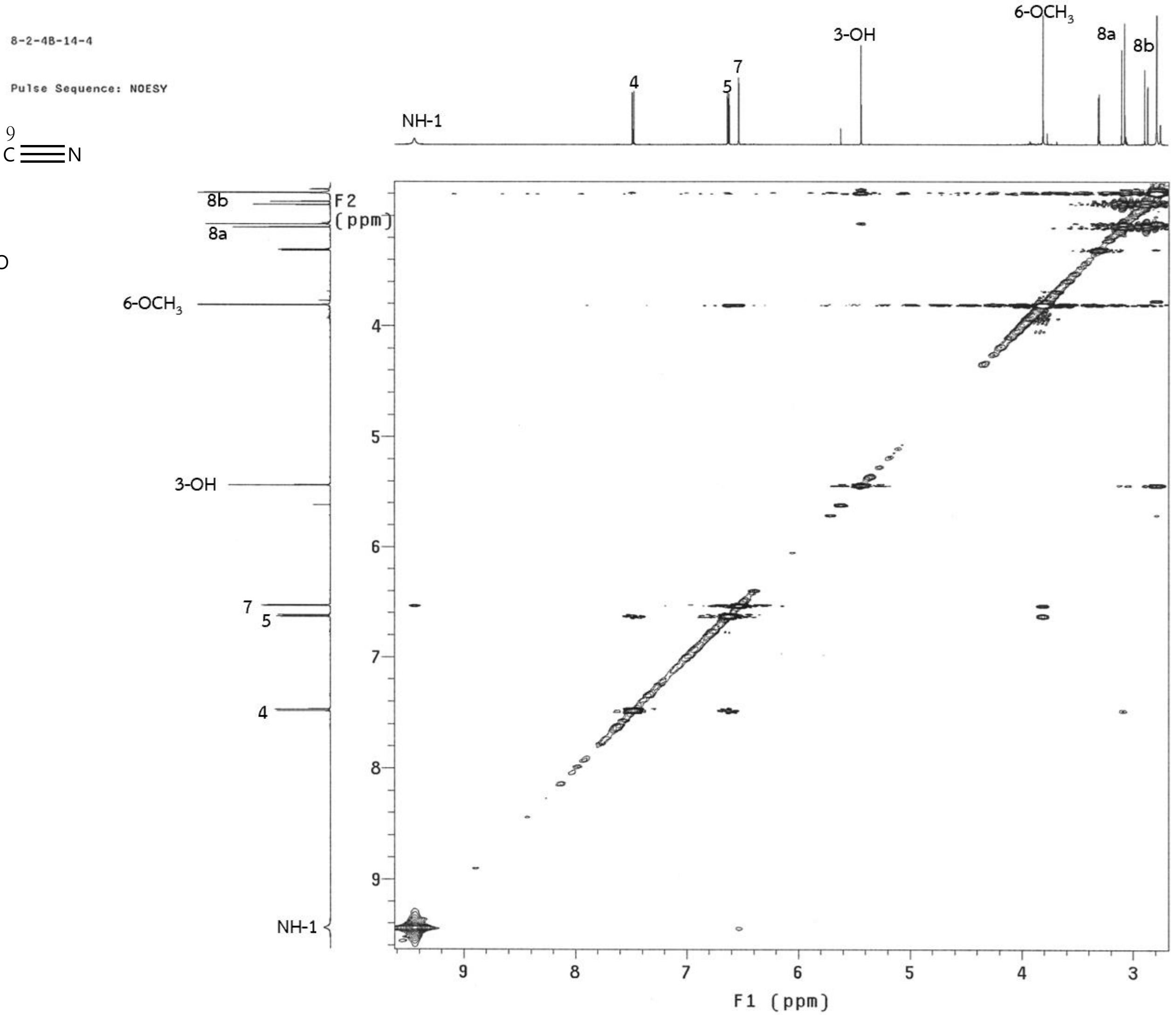

Figure S11. The NOESY spectrum of compound **1**

Mass Spectrum SmartFormula Report

Analysis Info

Analysis Name	D:\Data\f7\MSRE824B148_000002.d	6/30/2020 3:26:08 PM
Method	broadband first signal	Operator: YU HSIAO-CHING
Sample Name	MSRE-8-2-4B	Instrument: BRUKER FT-MS solariX
Comment	ESI Positive	

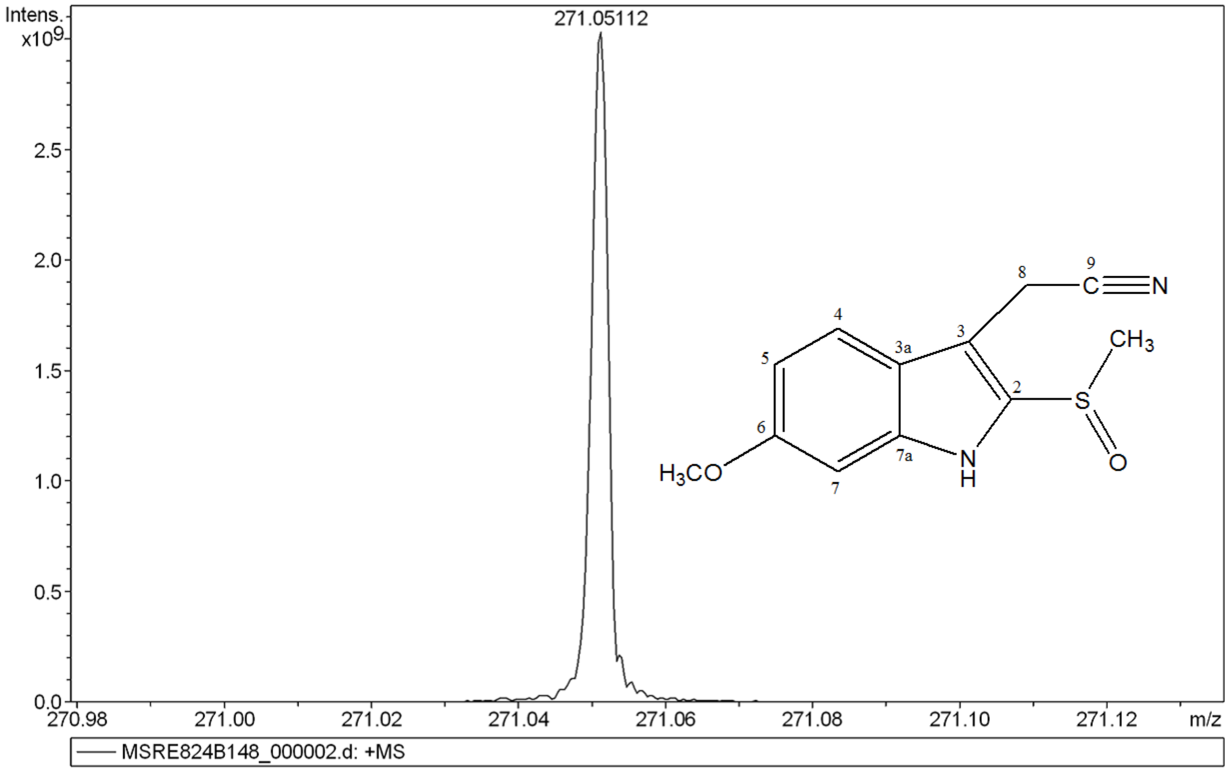

Meas. m/z	#	Formula	Score	m/z	err [mDa]	err [ppm]	mSigma	rdb	e⁻ Conf	N-Rule
271.05112	1	C 12 H 12 N 2 Na O 2 S	100.00	271.05117	0.05	0.19	38.1	7.5	even	ok

Figure S12. The HR-ESI-MS spectrum of compound **2**

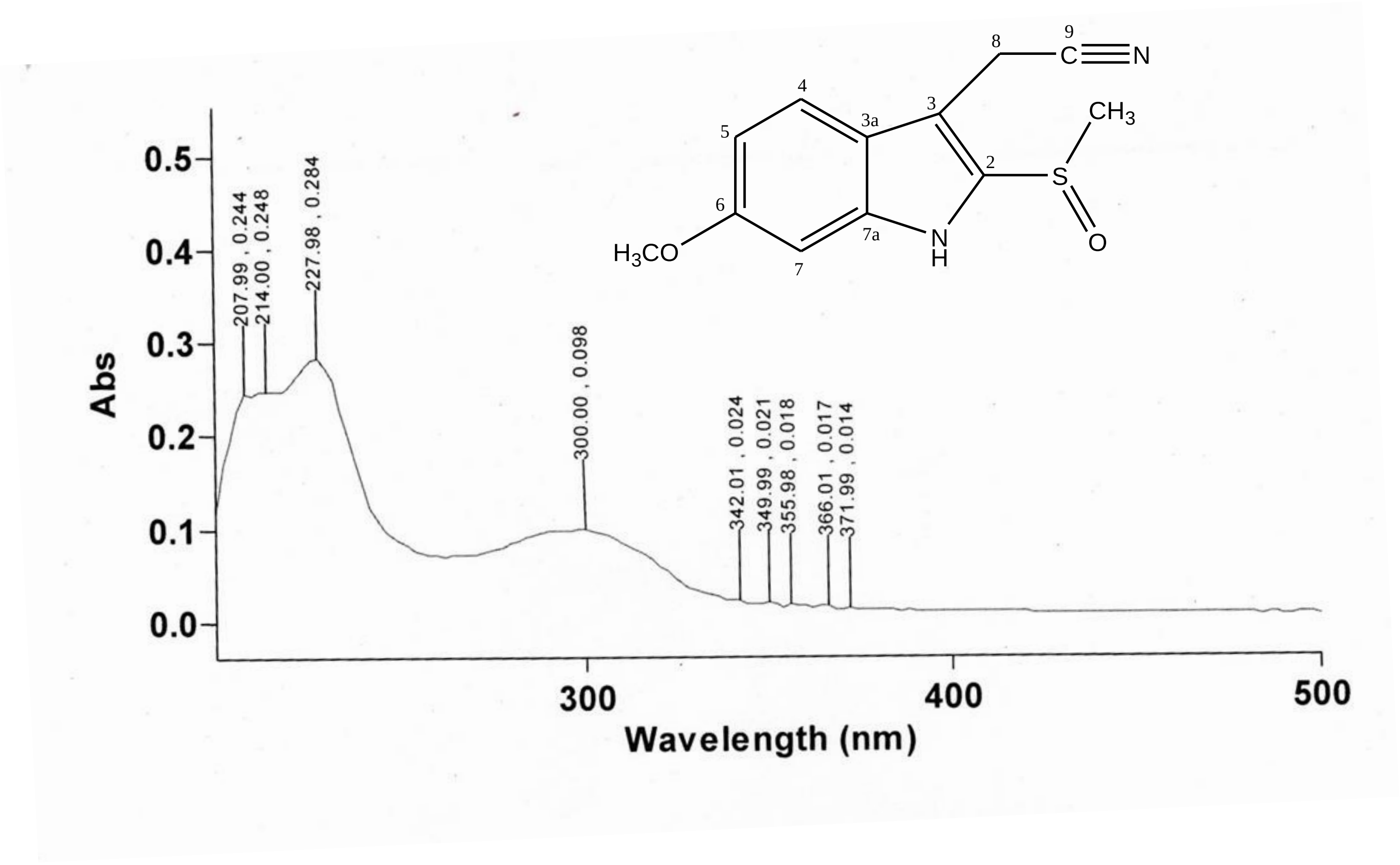

Figure S13. The UV spectrum of compound **2**

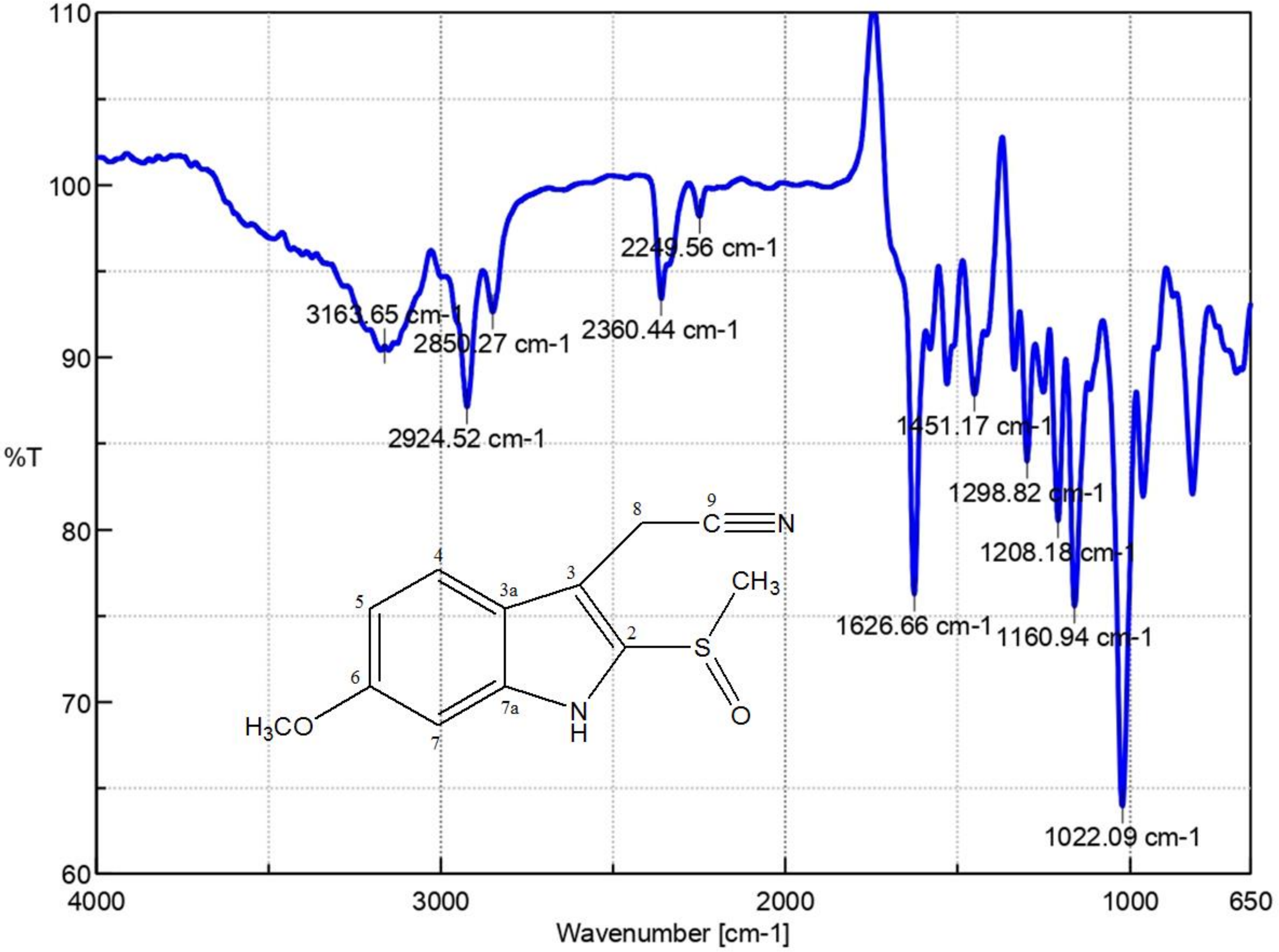

Figure S14. The IR spectrum of compound **2** (ATR)

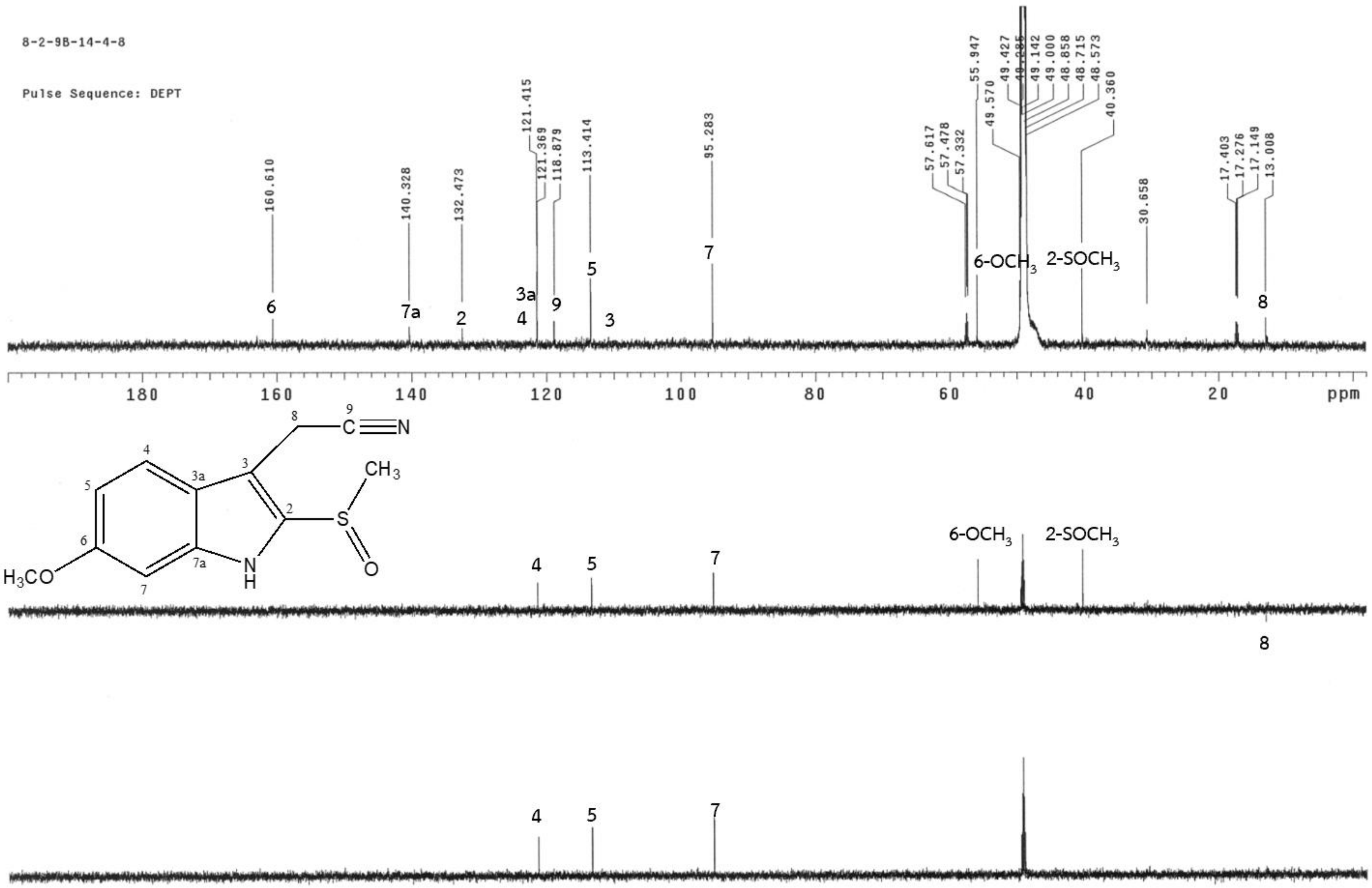

Figure S15. The ^{13}C-NMR, DEPT 135 and DEPT 90 (150 MHz, CD_3OD) spectra of compound **2**

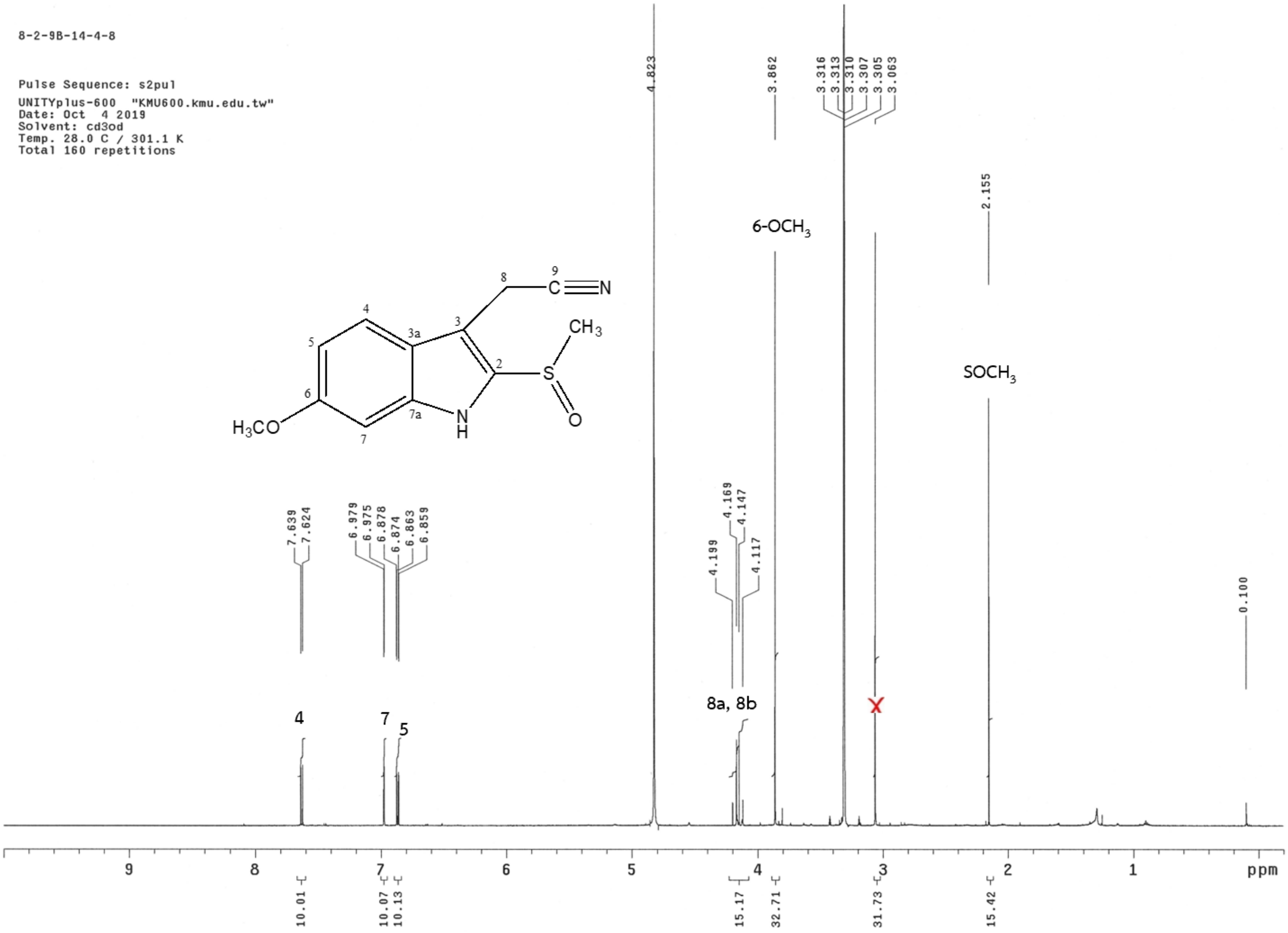

Figure S16. The ^{1}H NMR (600 MHz, CD_3OD) spectrum of compound **2**

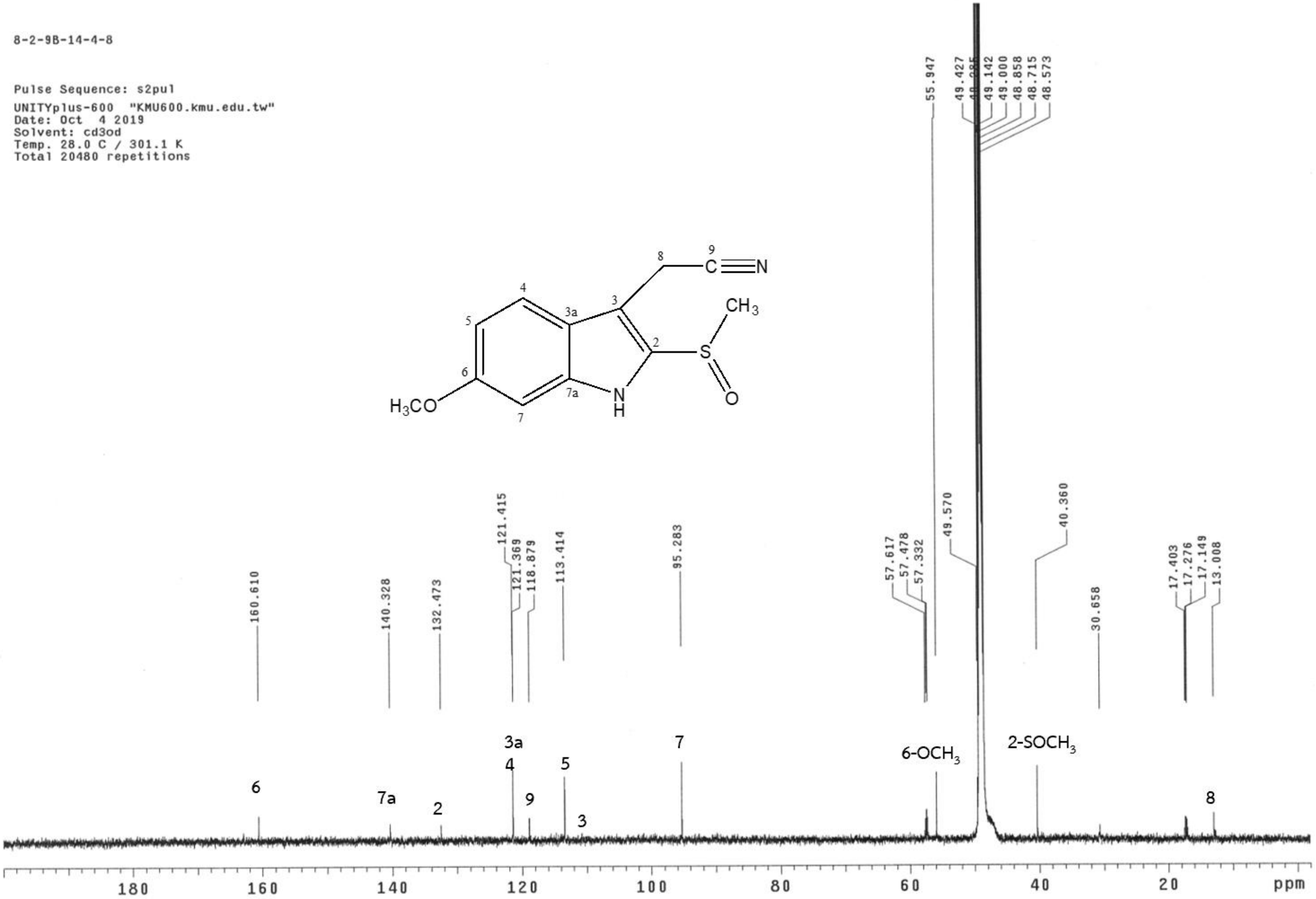

Figure S17. The ^{13}C NMR (150 MHz, CD_3OD) spectrum of compound **2**

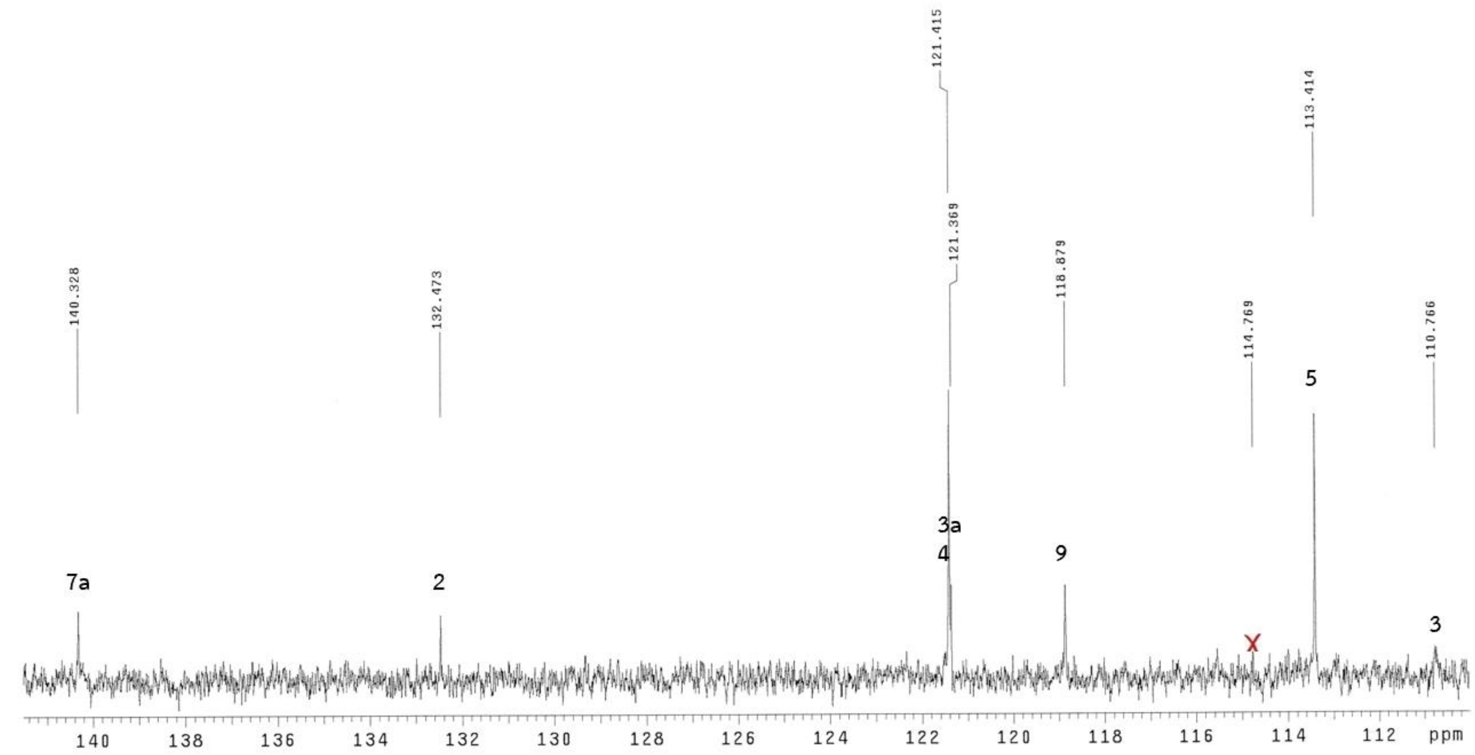

Figure S18. The ^{13}C NMR (150 MHz, CD_3OD) spectrum of compound **2 (continued)**

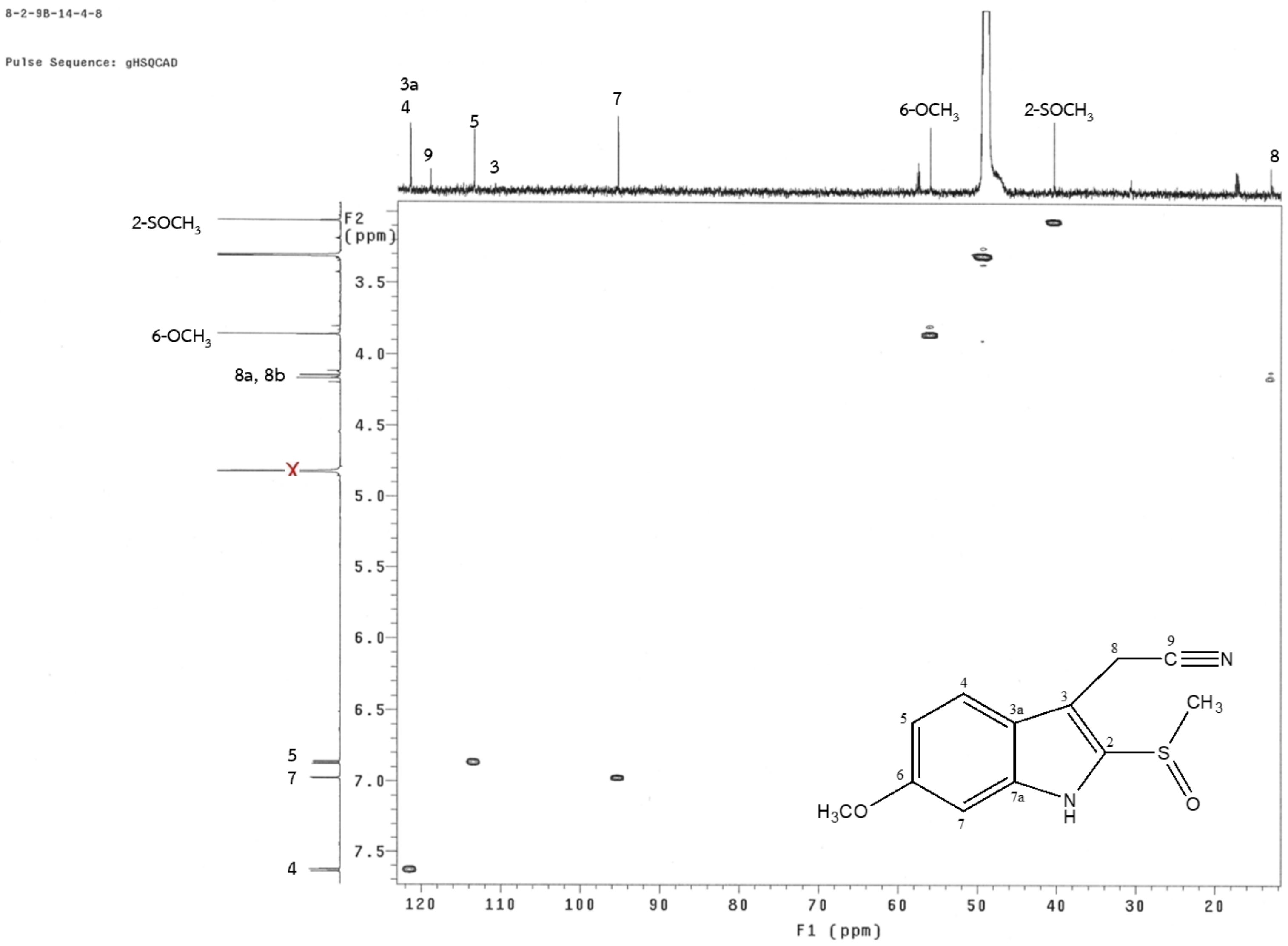

Figure S19. The HSQC spectrum of compound **2**

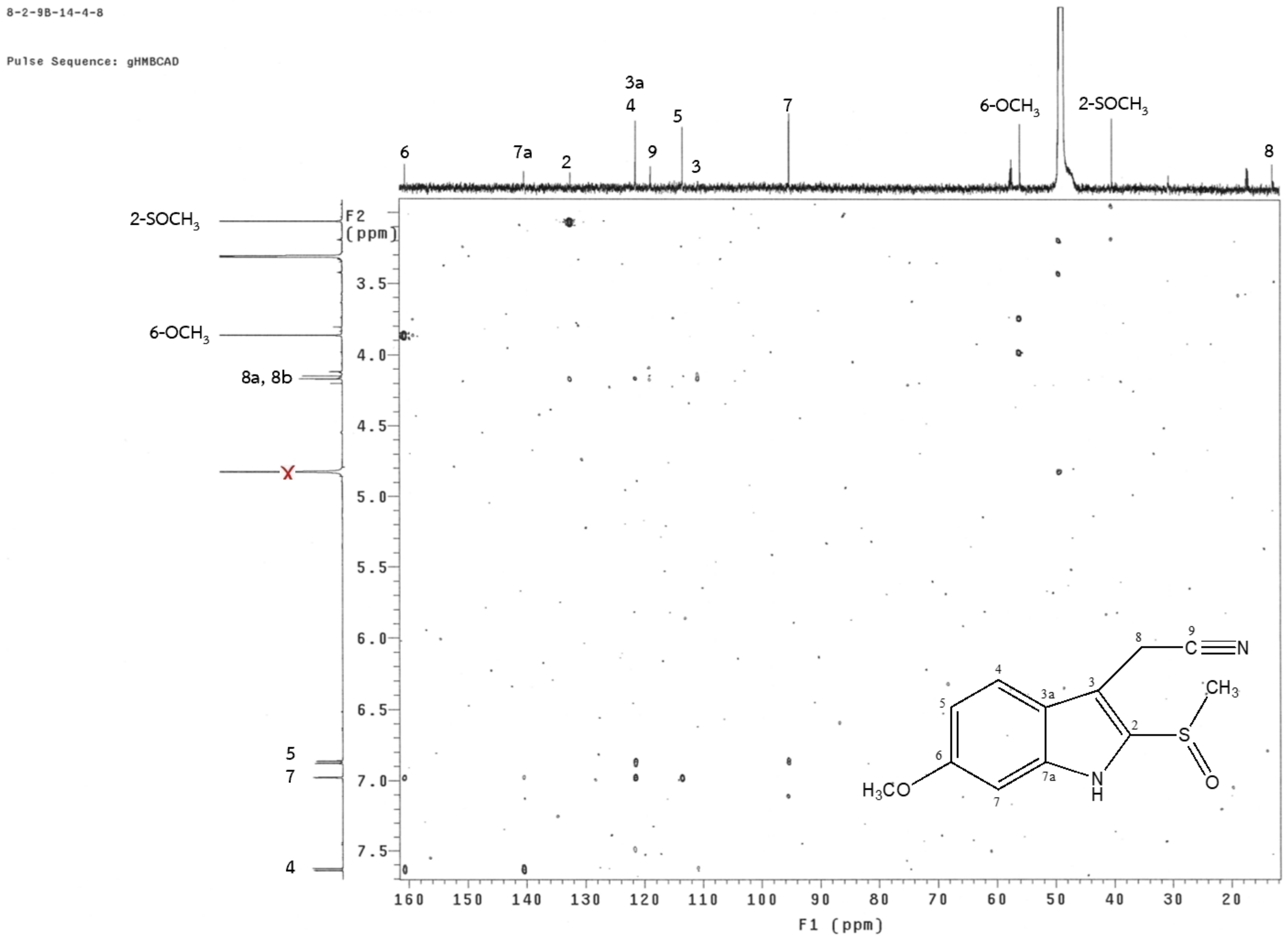

Figure S20. The HMBC spectrum of compound **2**

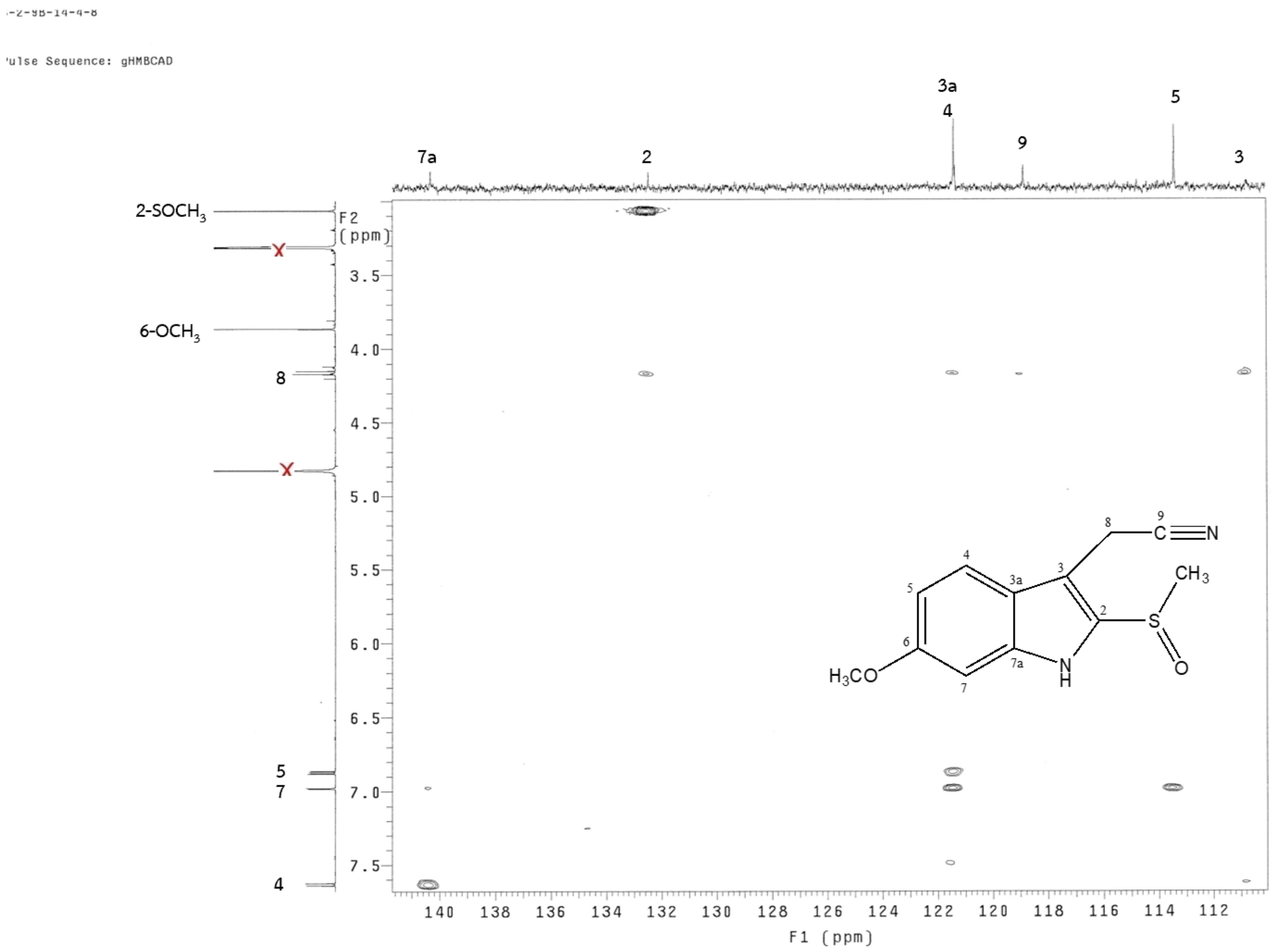

Figure S21. The HMBC spectrum of compound **2 (continued)**

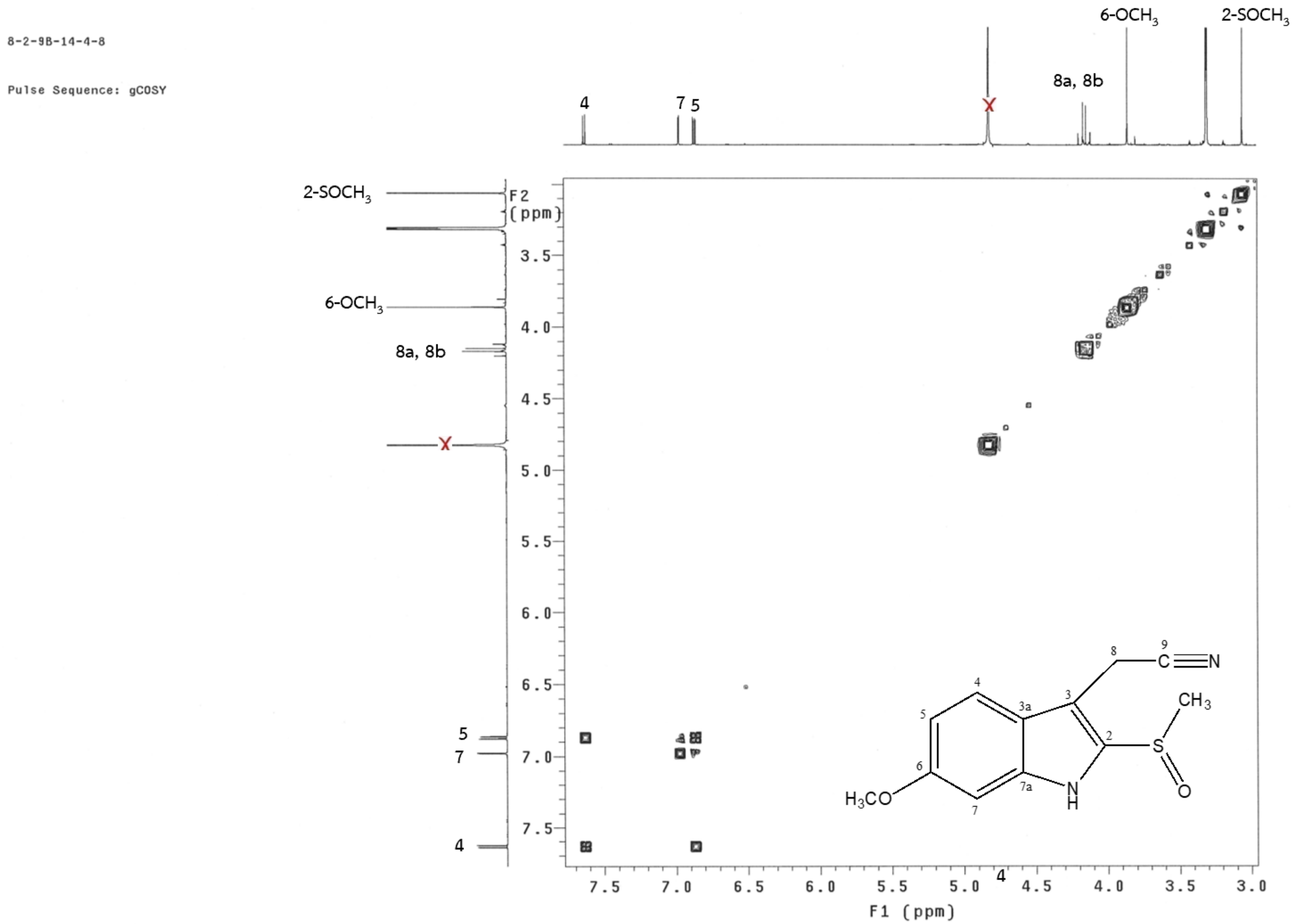

Figure S22. The ^{1}H-^{1}H COSY spectrum of compound **2**

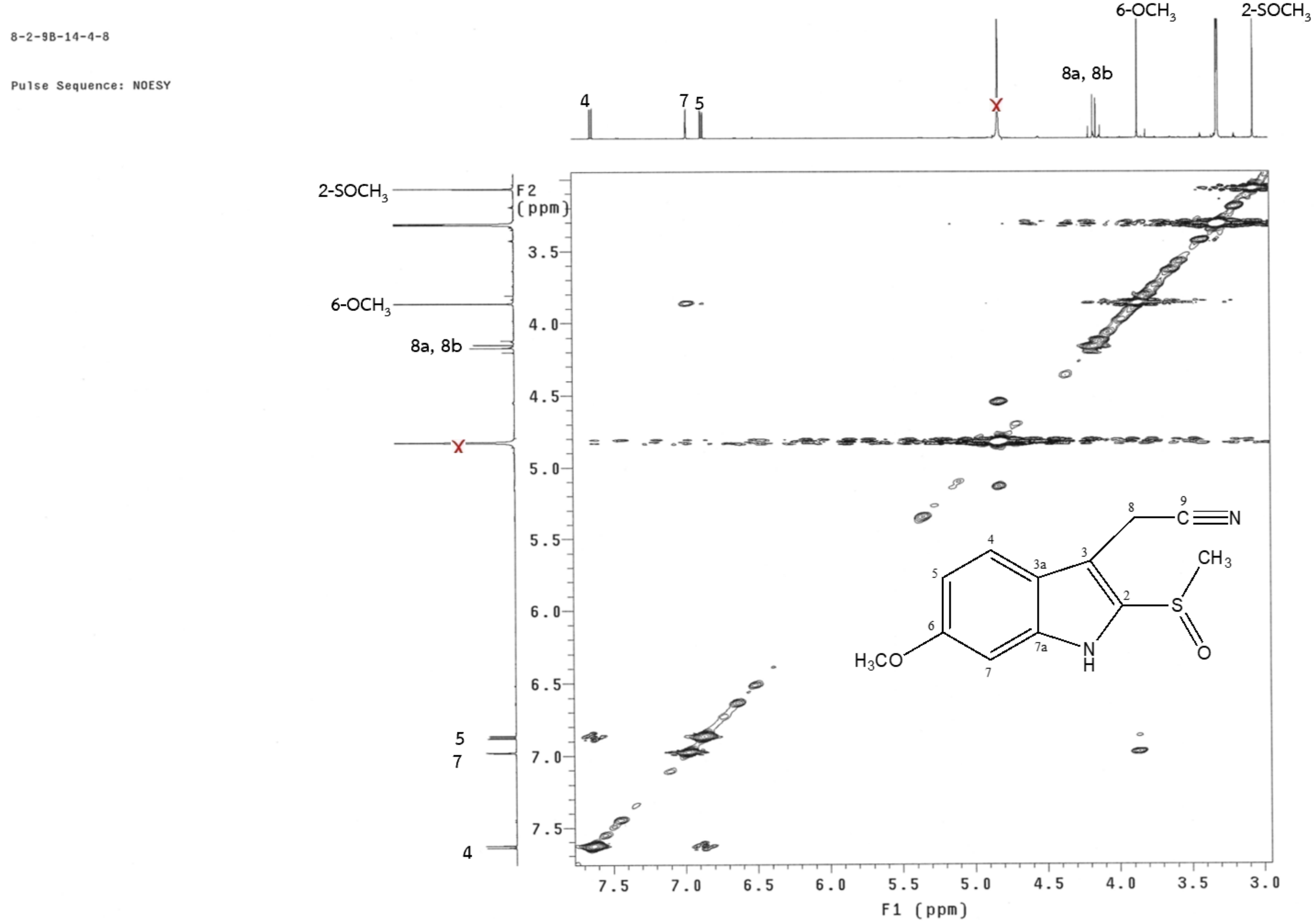

Figure S23. The NOESY spectrum of compound **2**

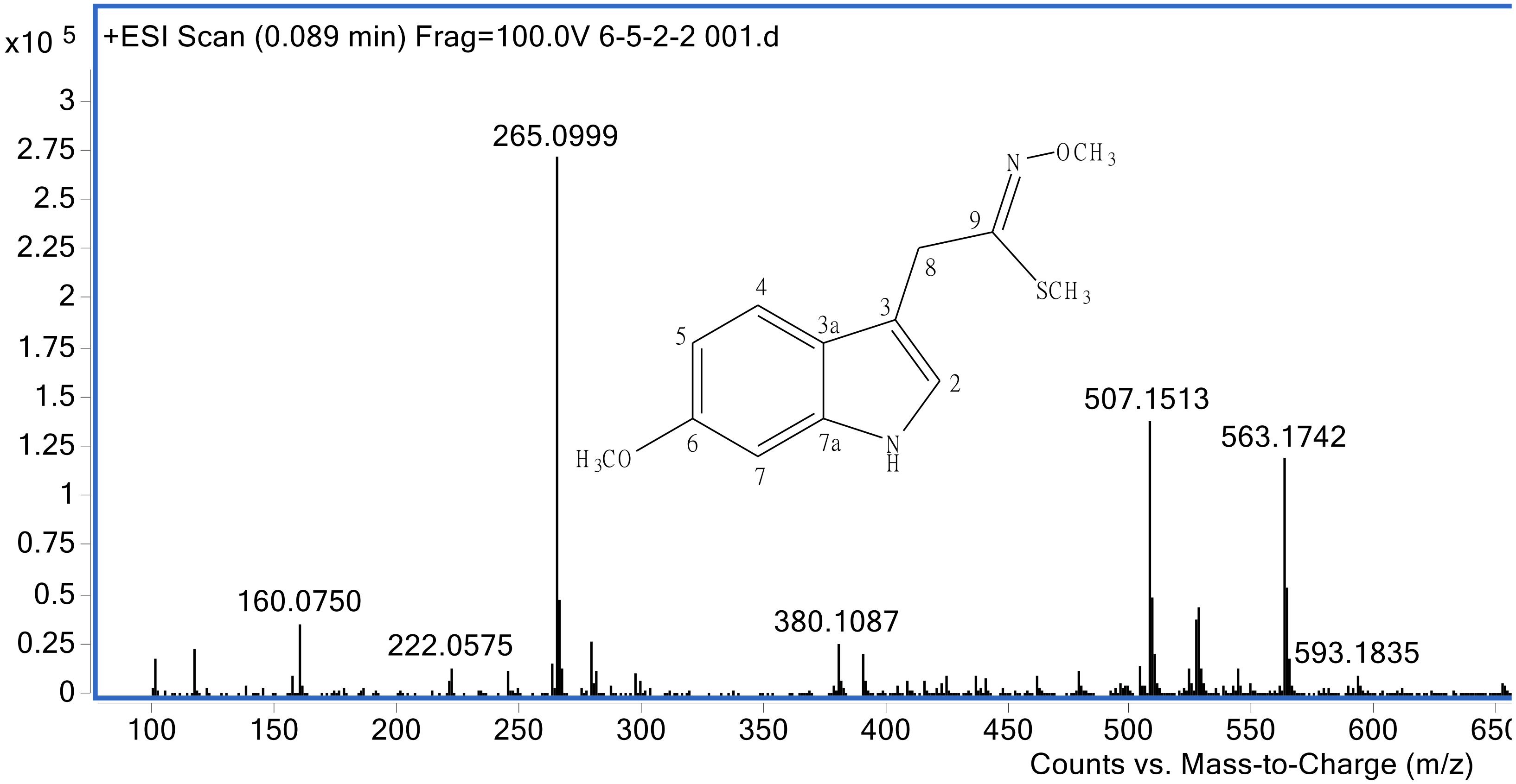

Figure S24. The HR-ESI-MS spectrum of compound **3**

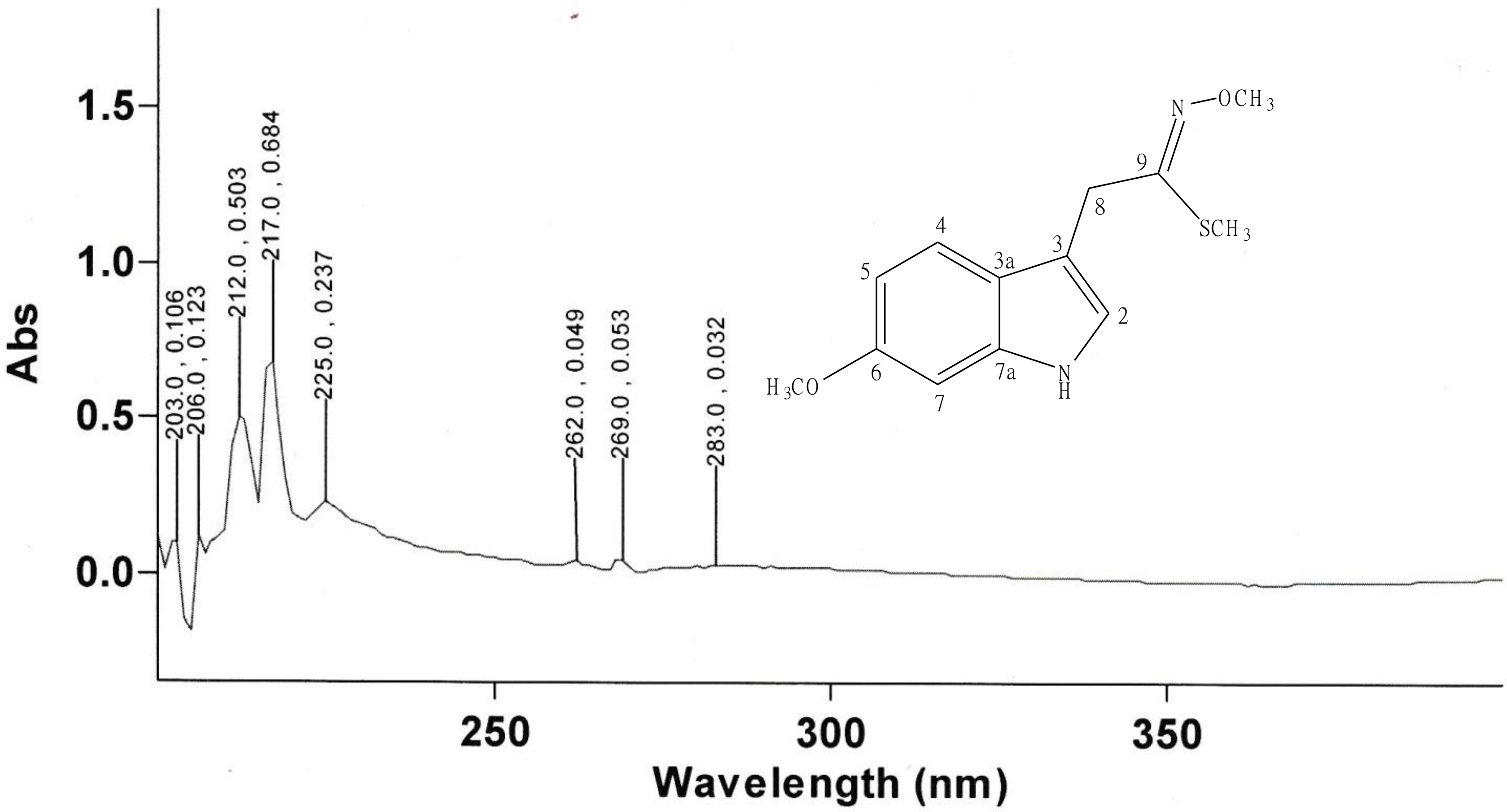

Figure S25. The UV spectrum of compound **3**

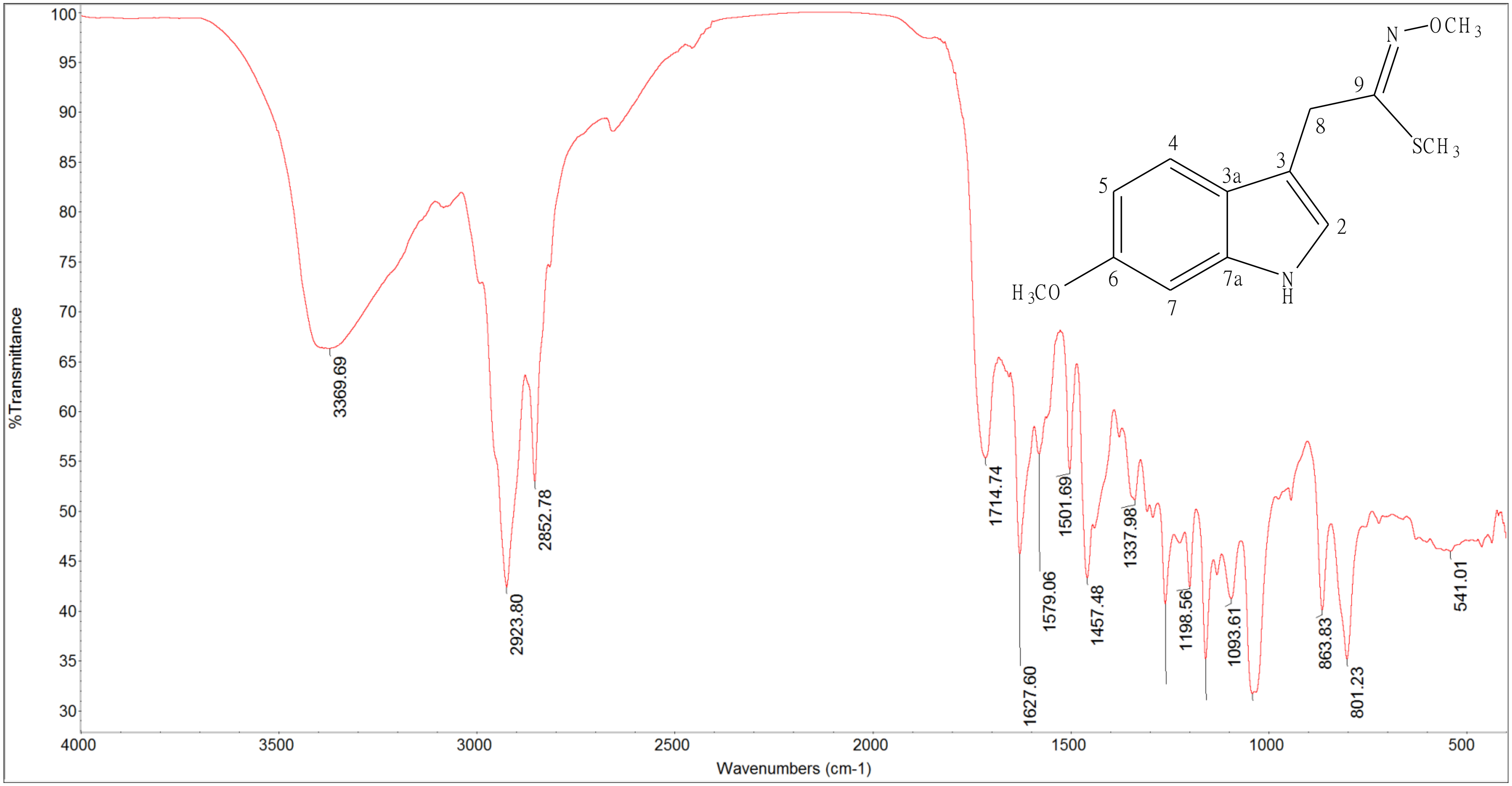

Figure S26. The IR spectrum of compound **3** (ATR)

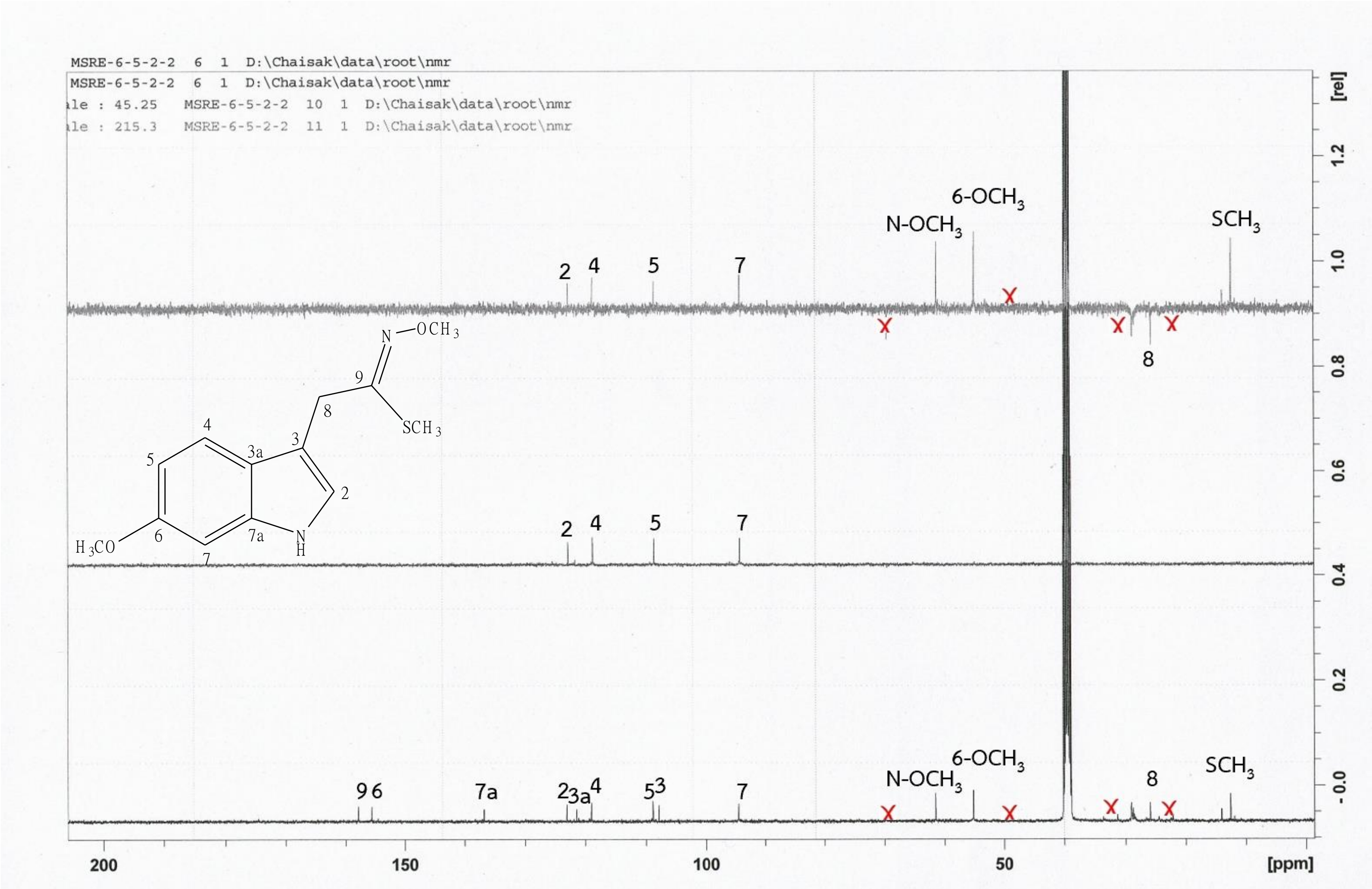

Figure S27. The ^{13}C-NMR, DEPT 135 and DEPT 90 (100 MHz, DMSO-d_6) spectra of compound **3**

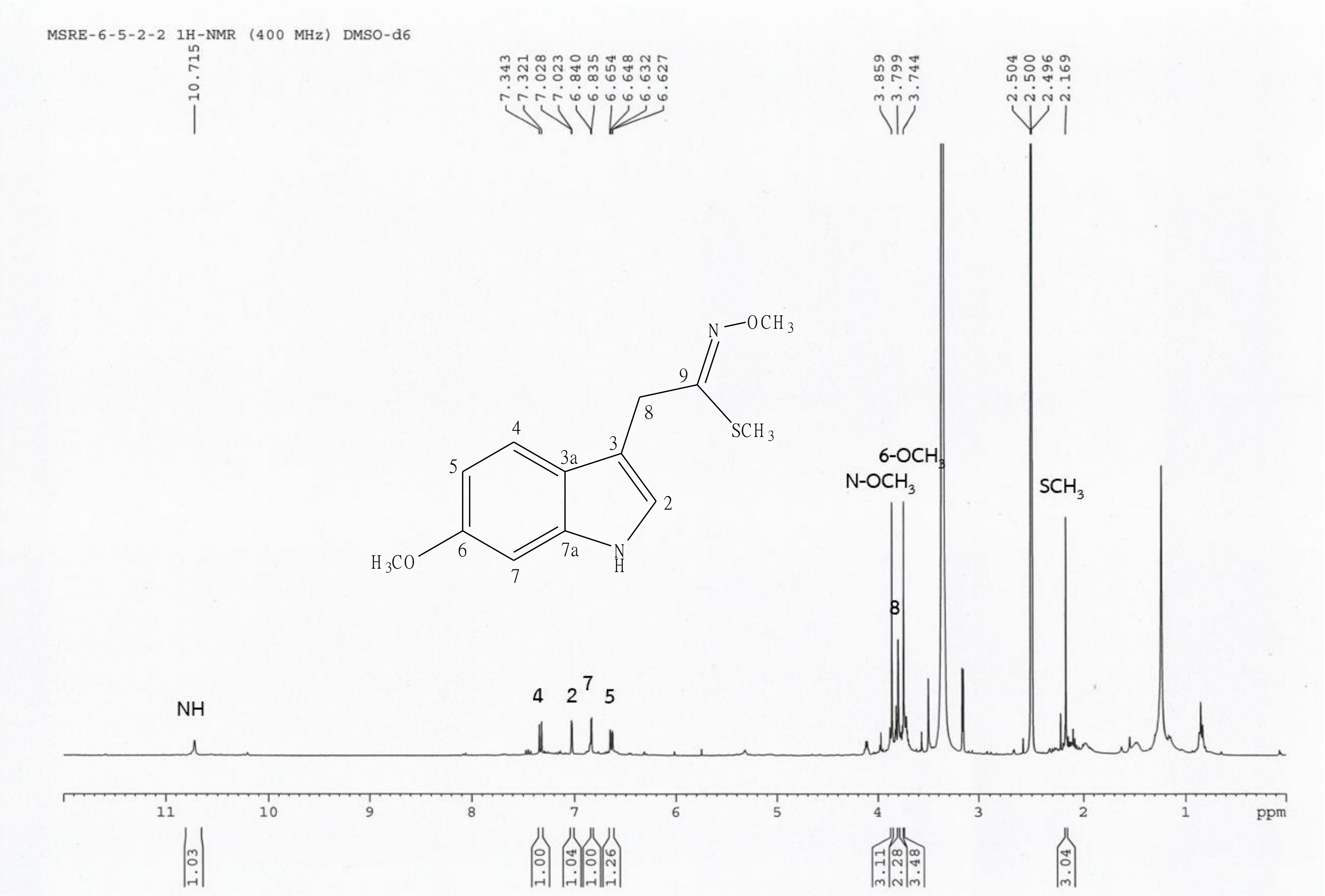

Figure S28. The ^{1}H NMR (400 MHz, DMSO-d_6) spectrum of compound **3**

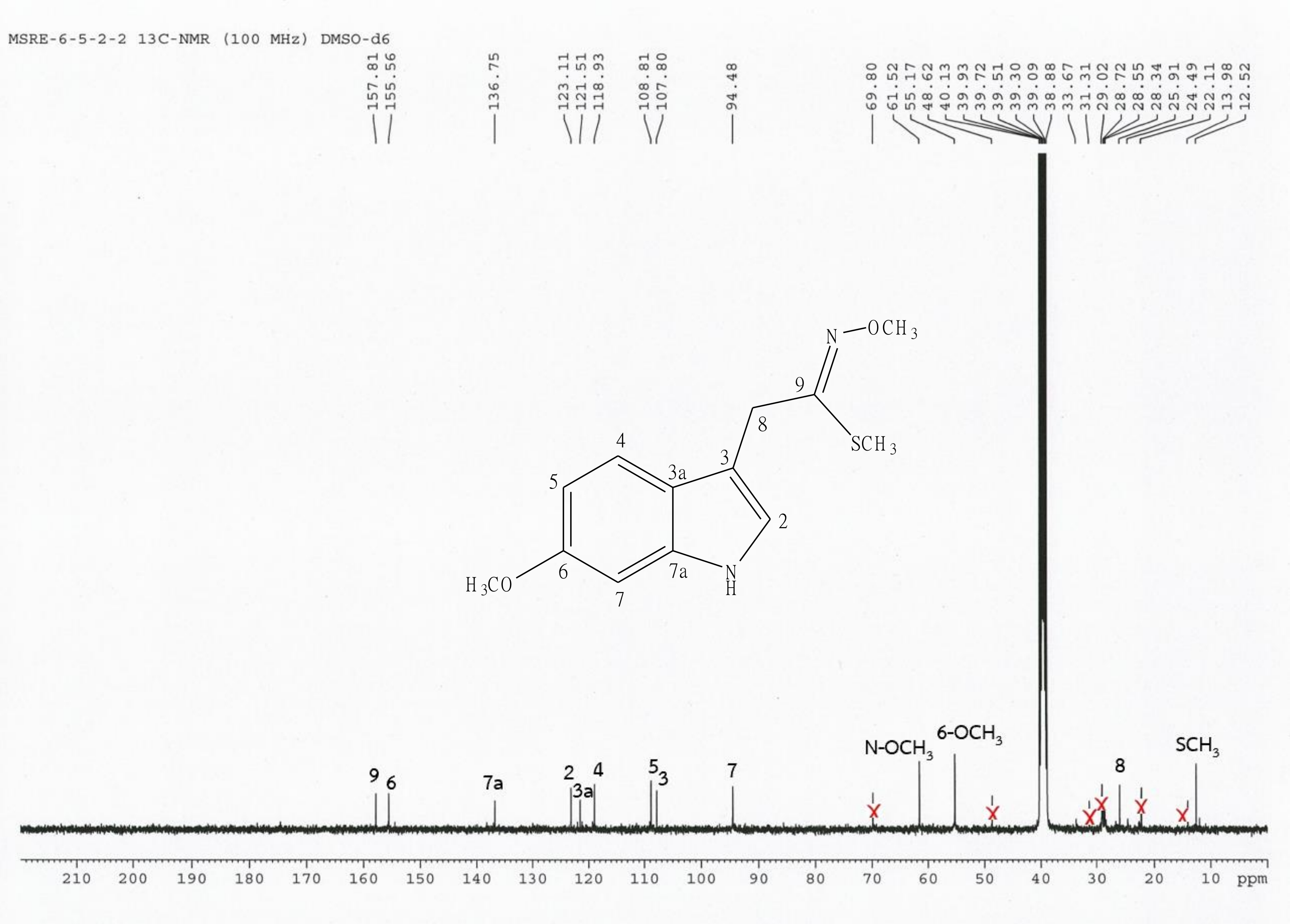

Figure S29. The ^{13}C NMR (100 MHz, DMSO-d_6) spectrum of compound **3**

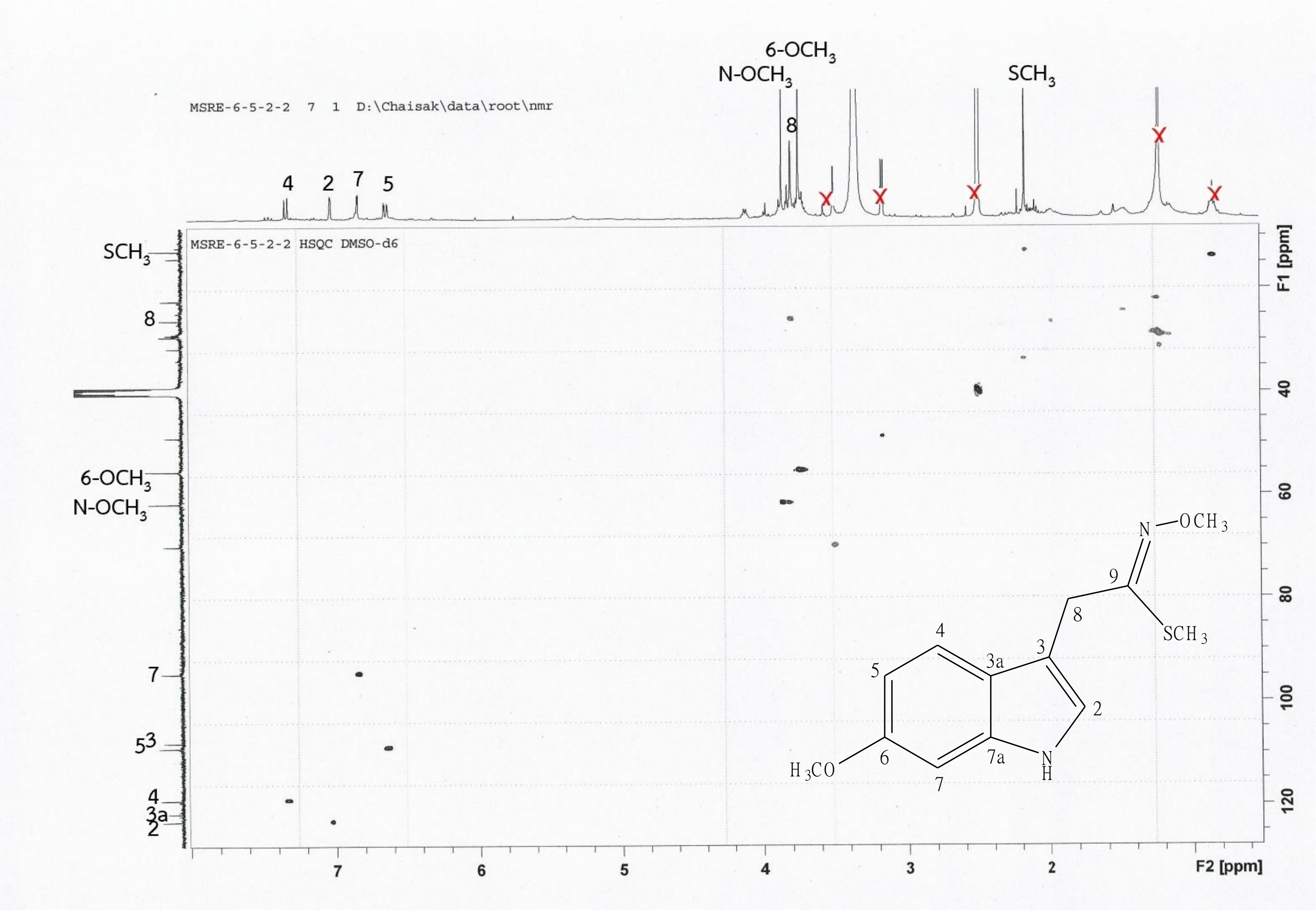

Figure S30. The HSQC spectrum of compound **3**

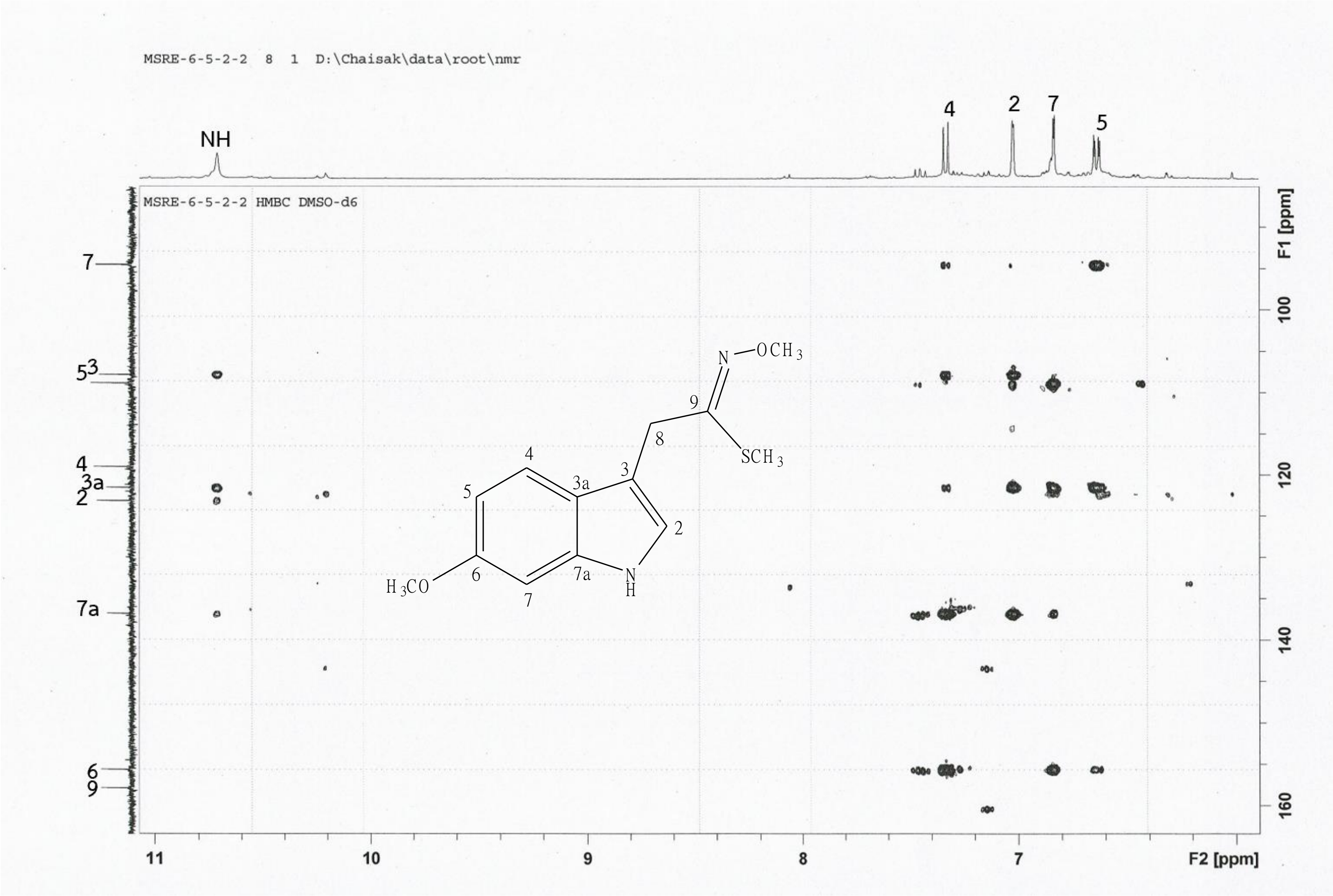

Figure S31. The HMBC spectrum of compound **3**

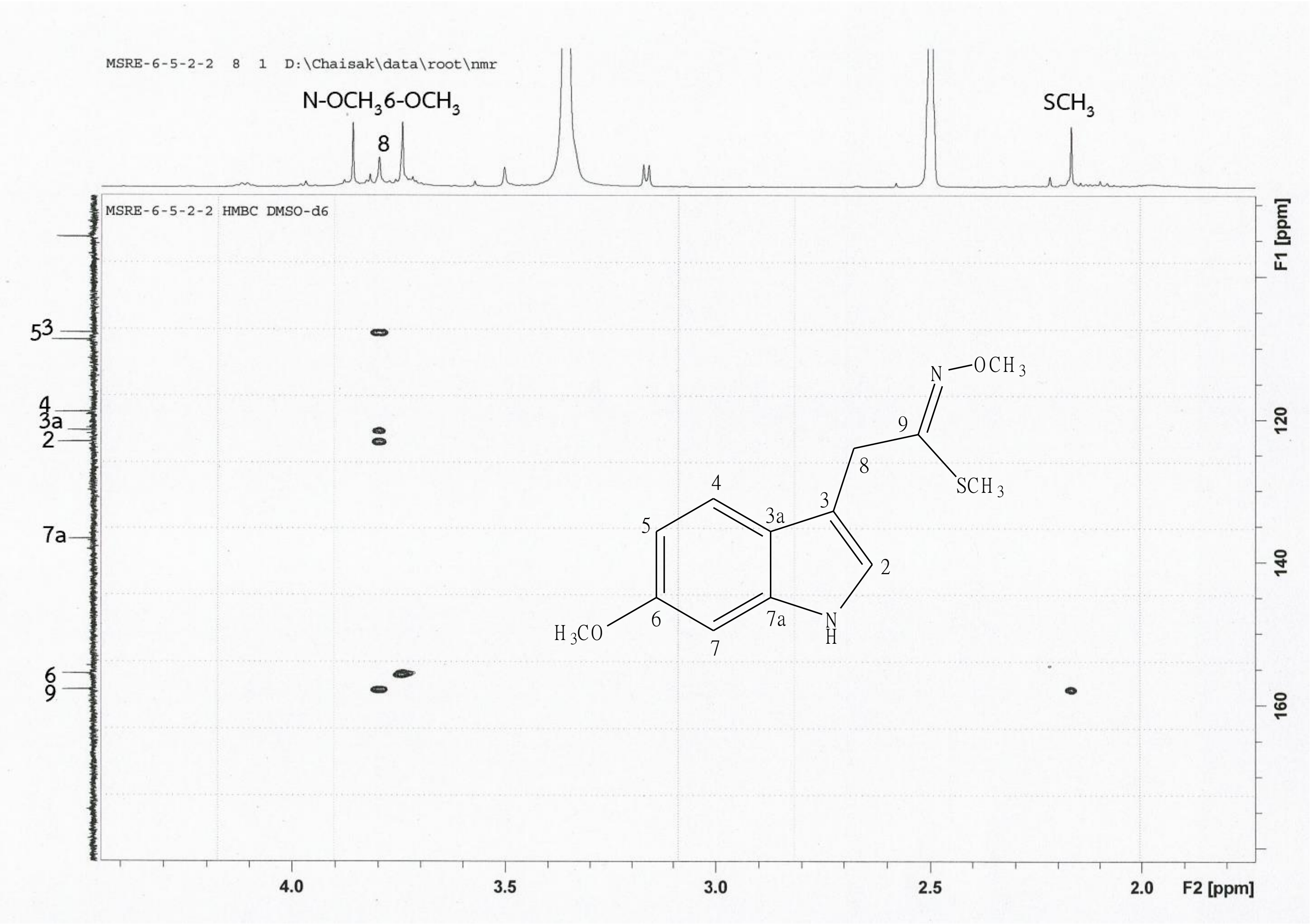

Figure S32. The HMBC spectrum of compound **3 (continued)**

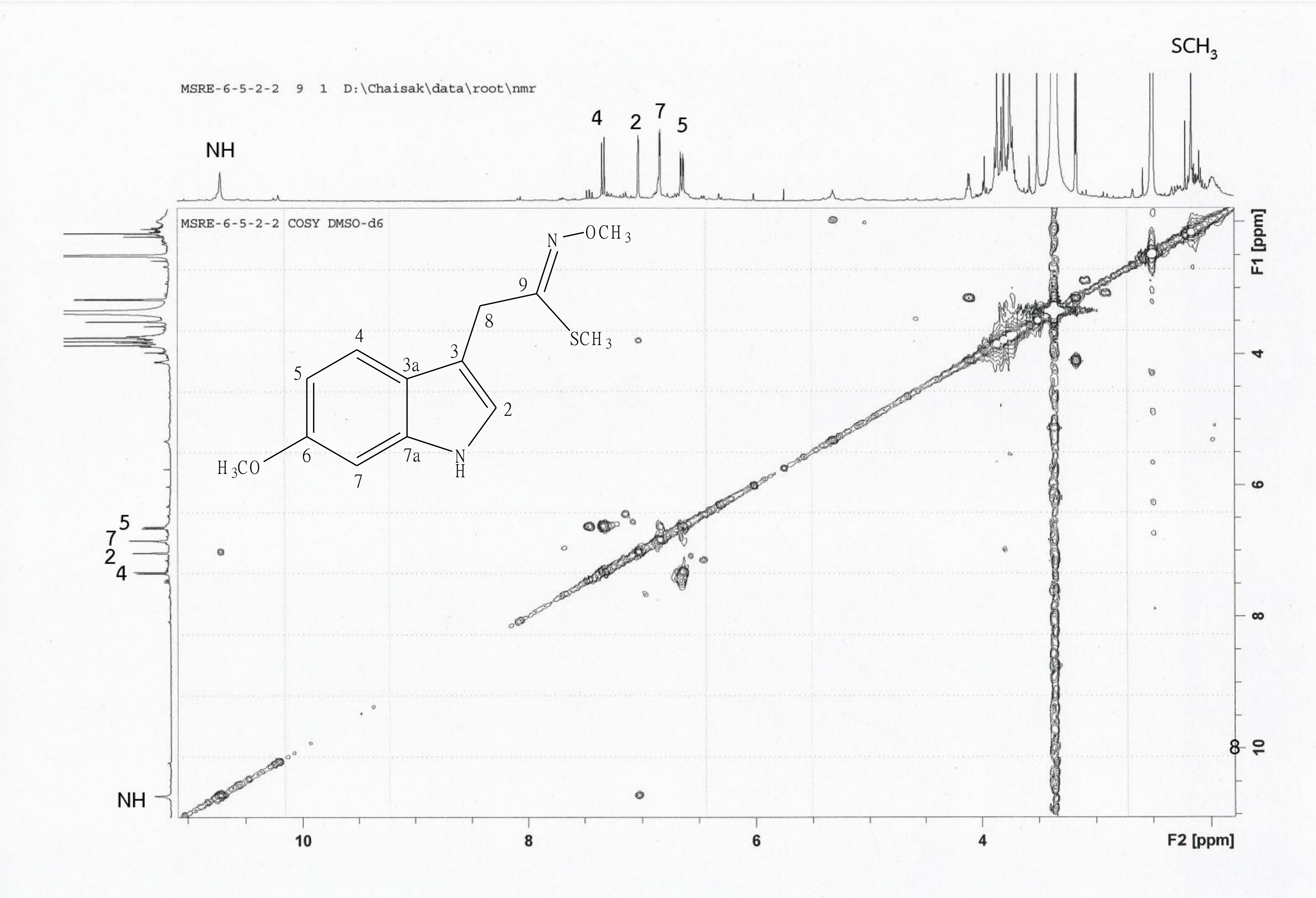

Figure S33. The ^{1}H-^{1}H COSY spectrum of compound **3**

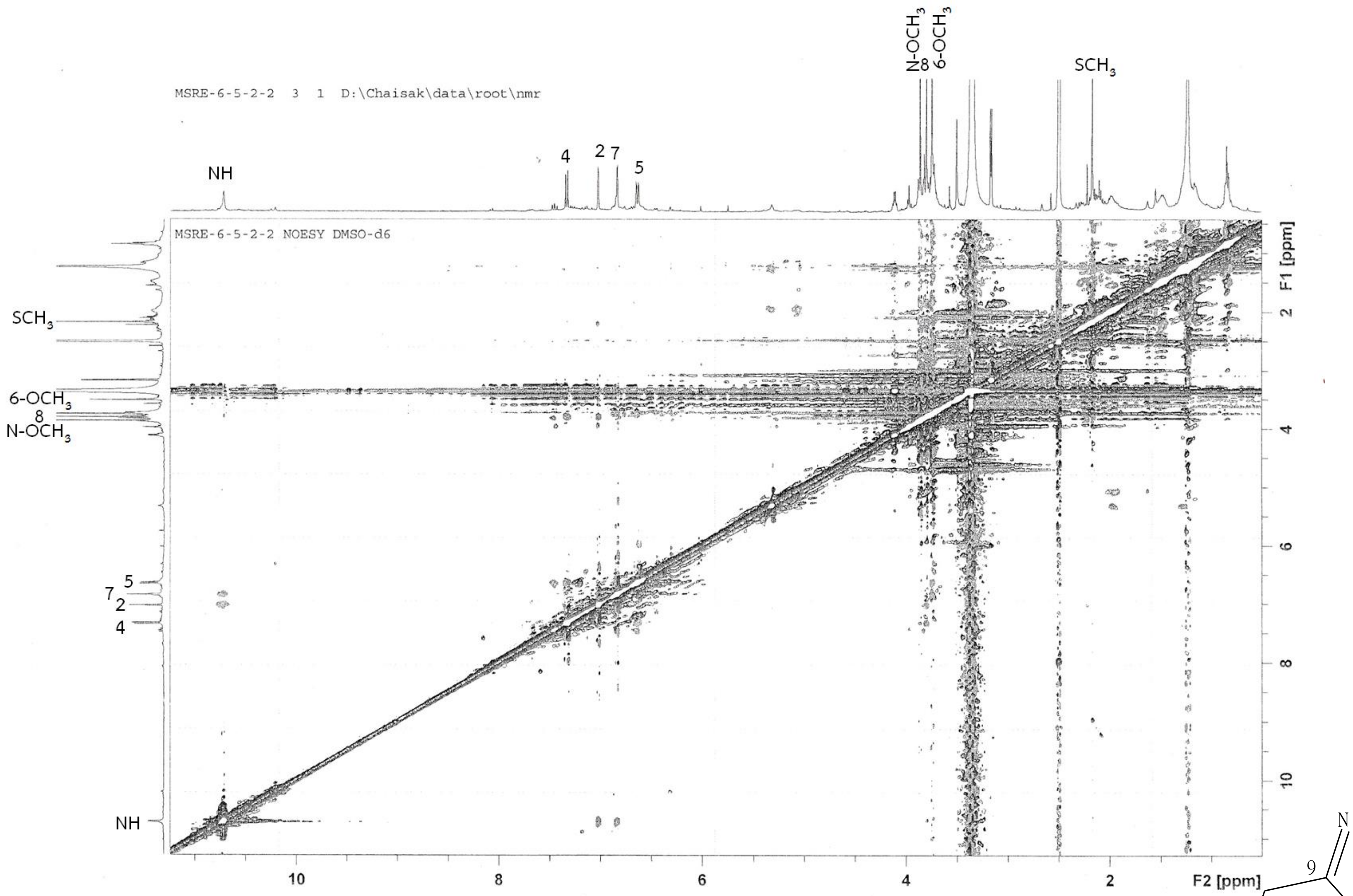

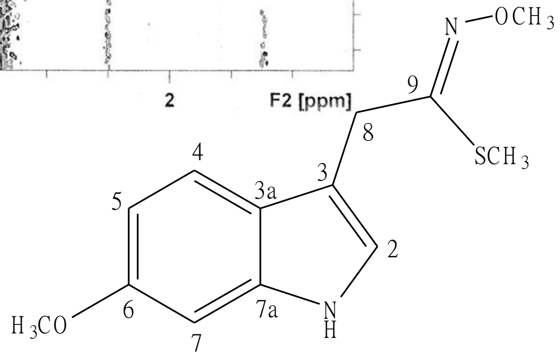

Figure S34. The NOESY spectrum of compound **3**

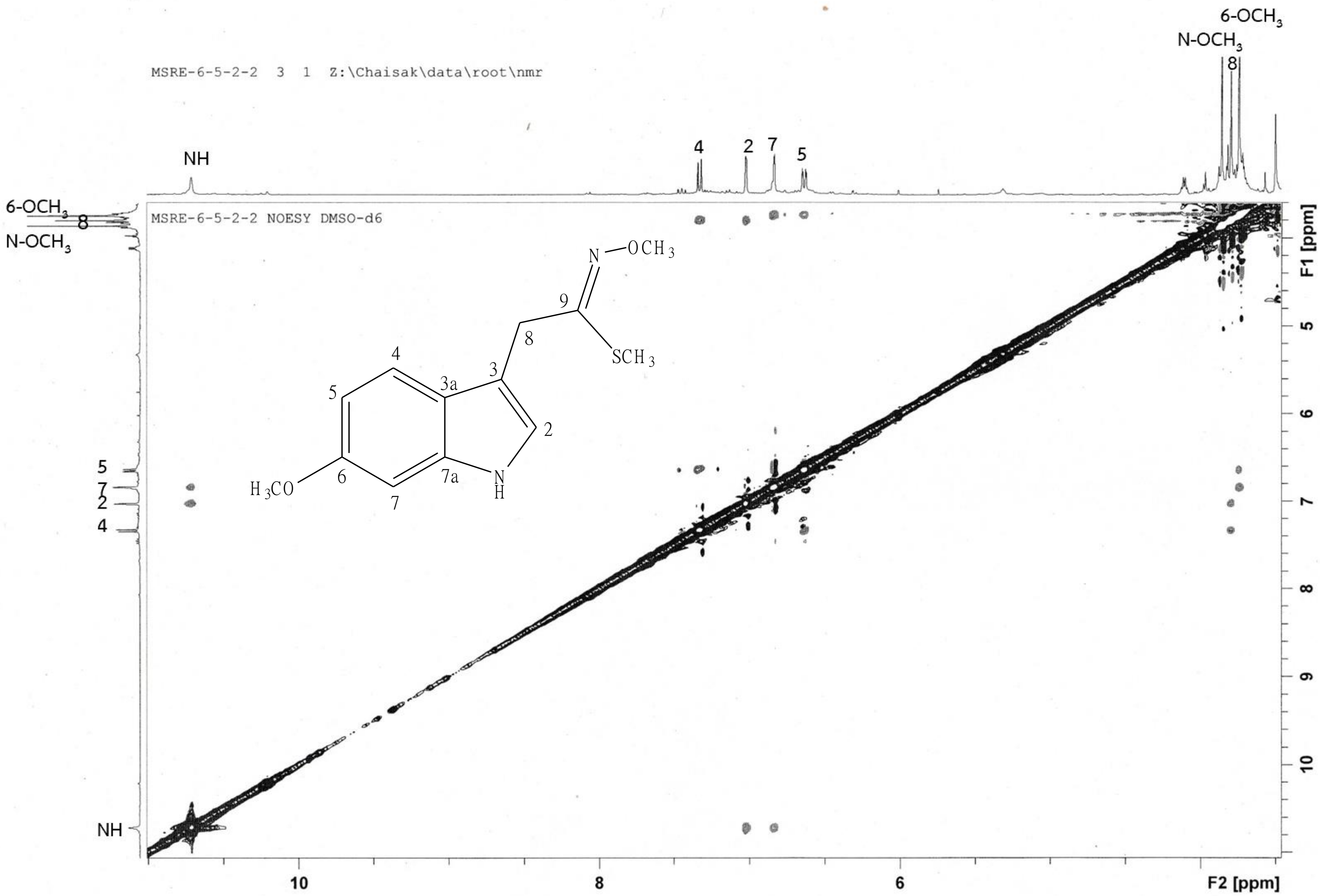

Figure S35. The NOESY spectrum of compound **3 (continued)**

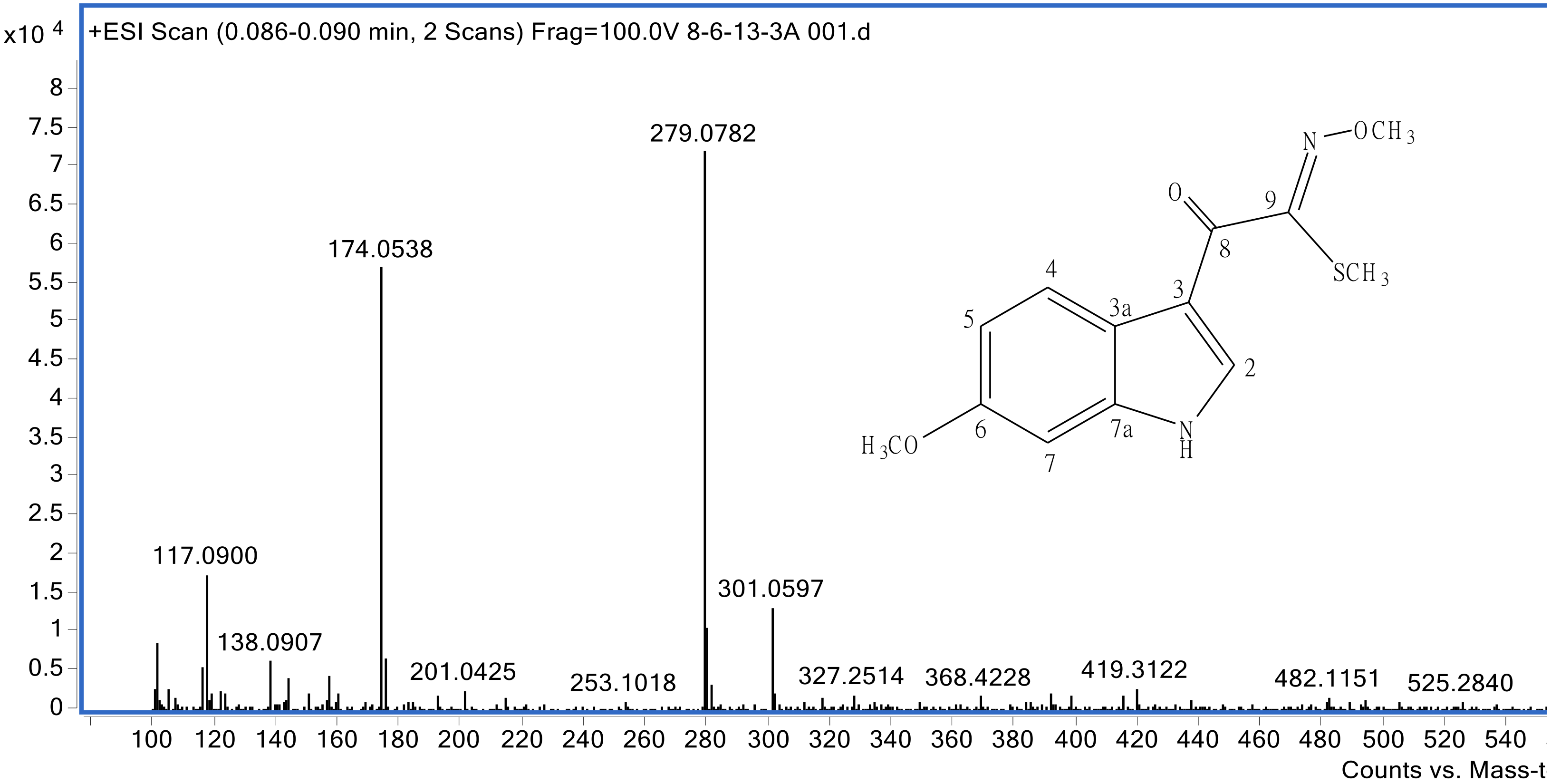

Figure S36. The HR-ESI-MS spectrum of compound **4**

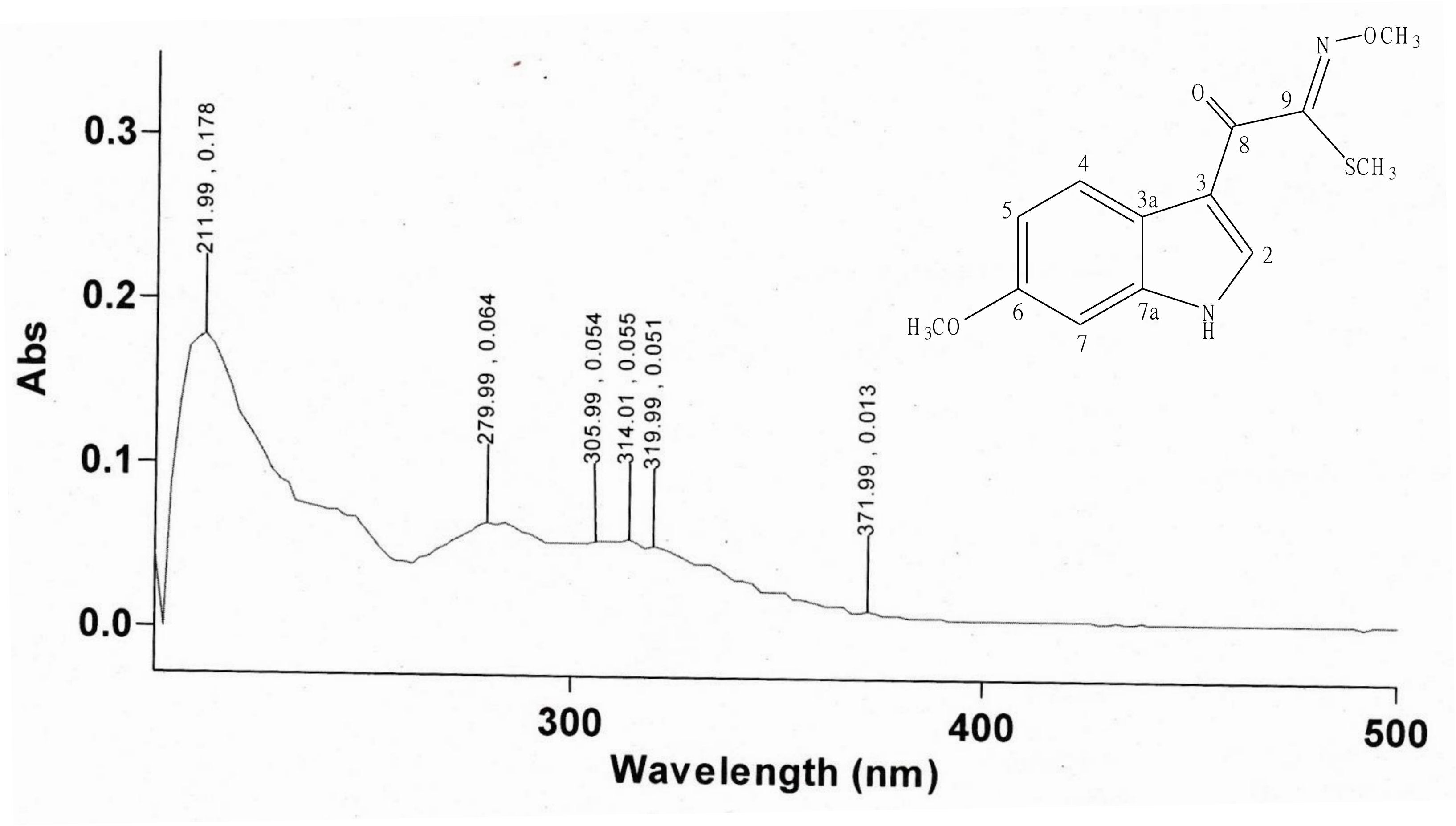

Figure S37. The UV spectrum of compound **4**

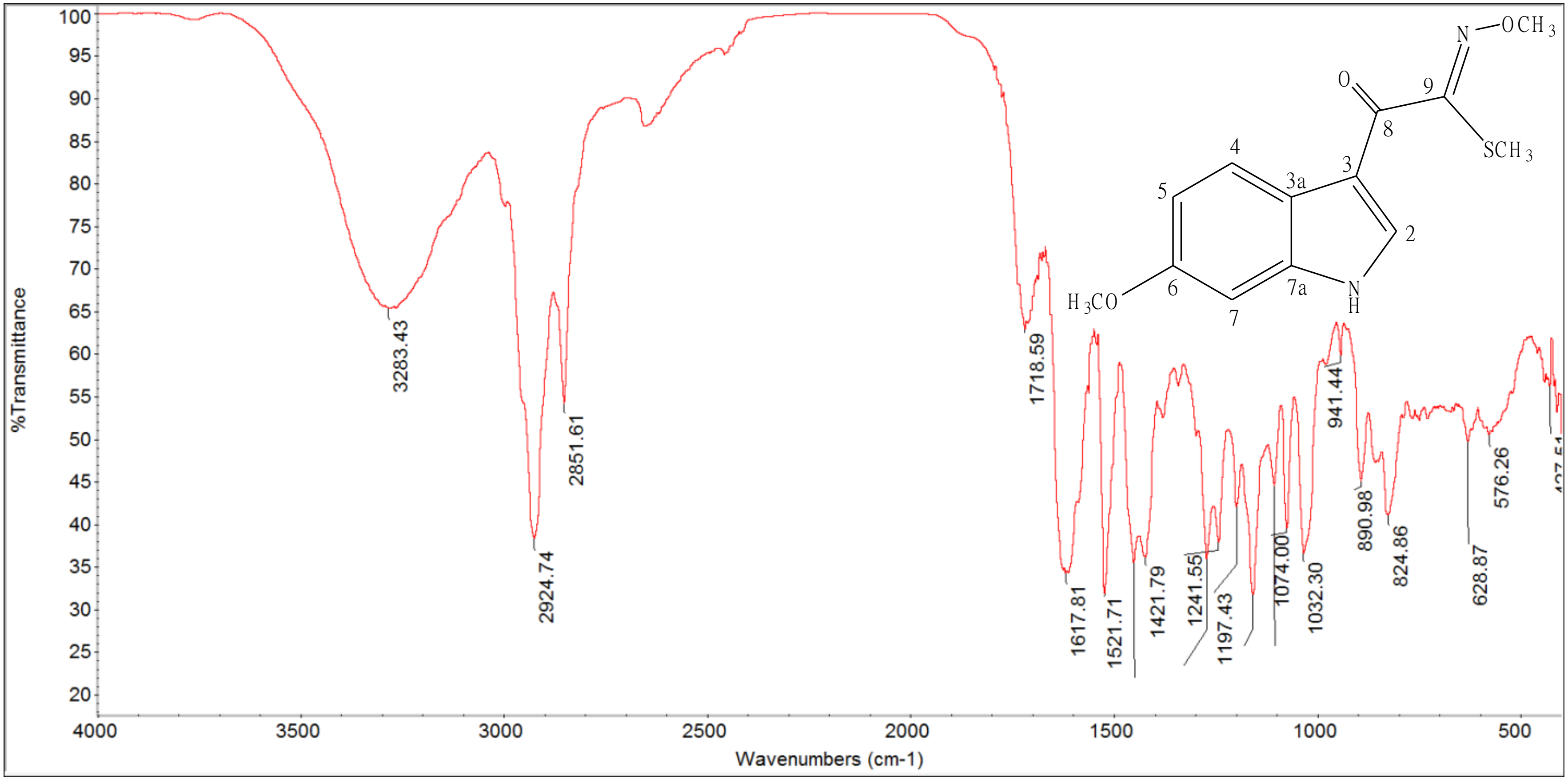

Figure S38. The IR spectrum of compound **4** (ATR)

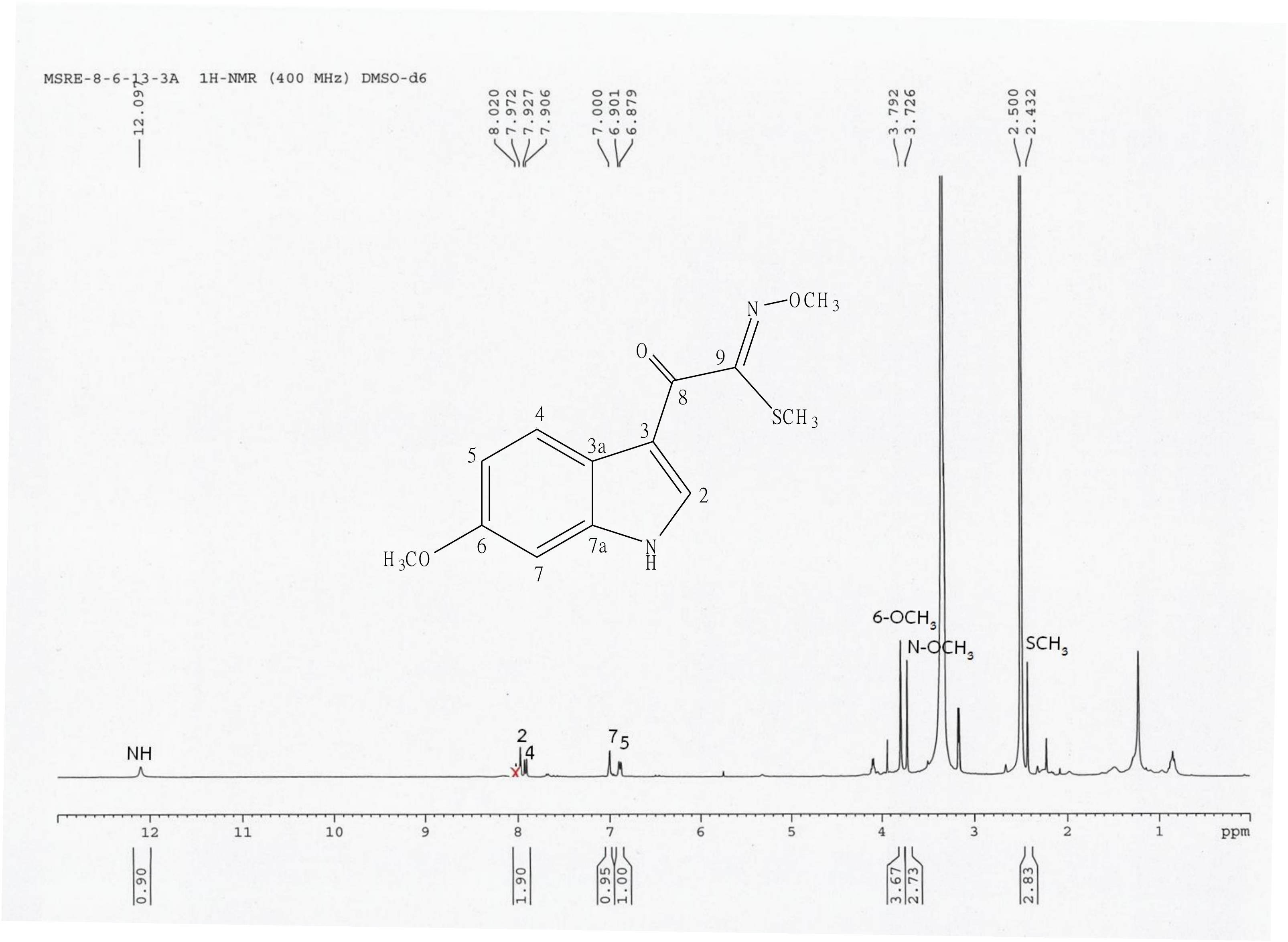

Figure S39. The ^{1}H NMR (400 MHz, DMSO-d_6) spectrum of compound **4**

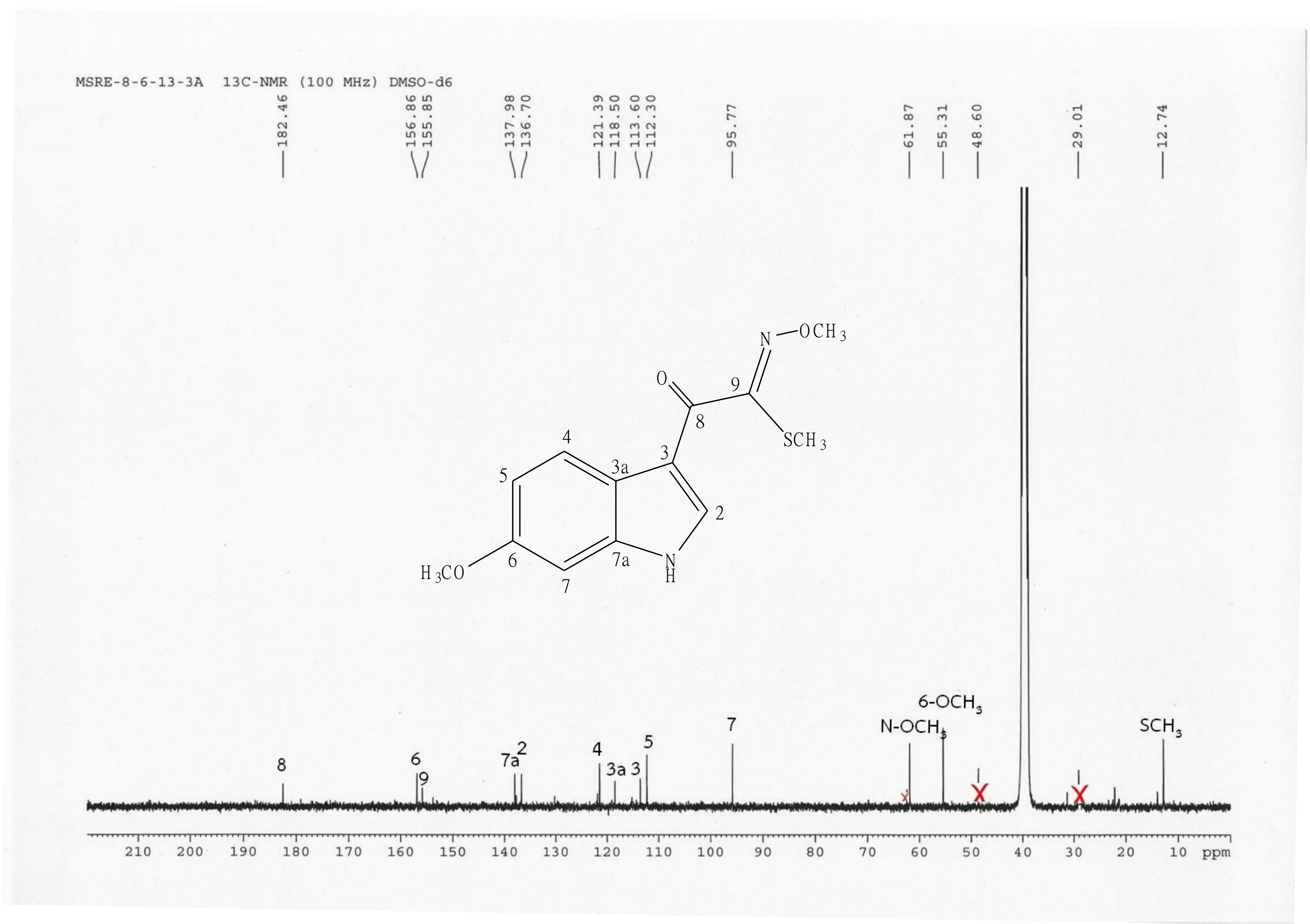

Figure S40. The ^{13}C NMR (100 MHz, DMSO-d_6) spectrum of compound **4**

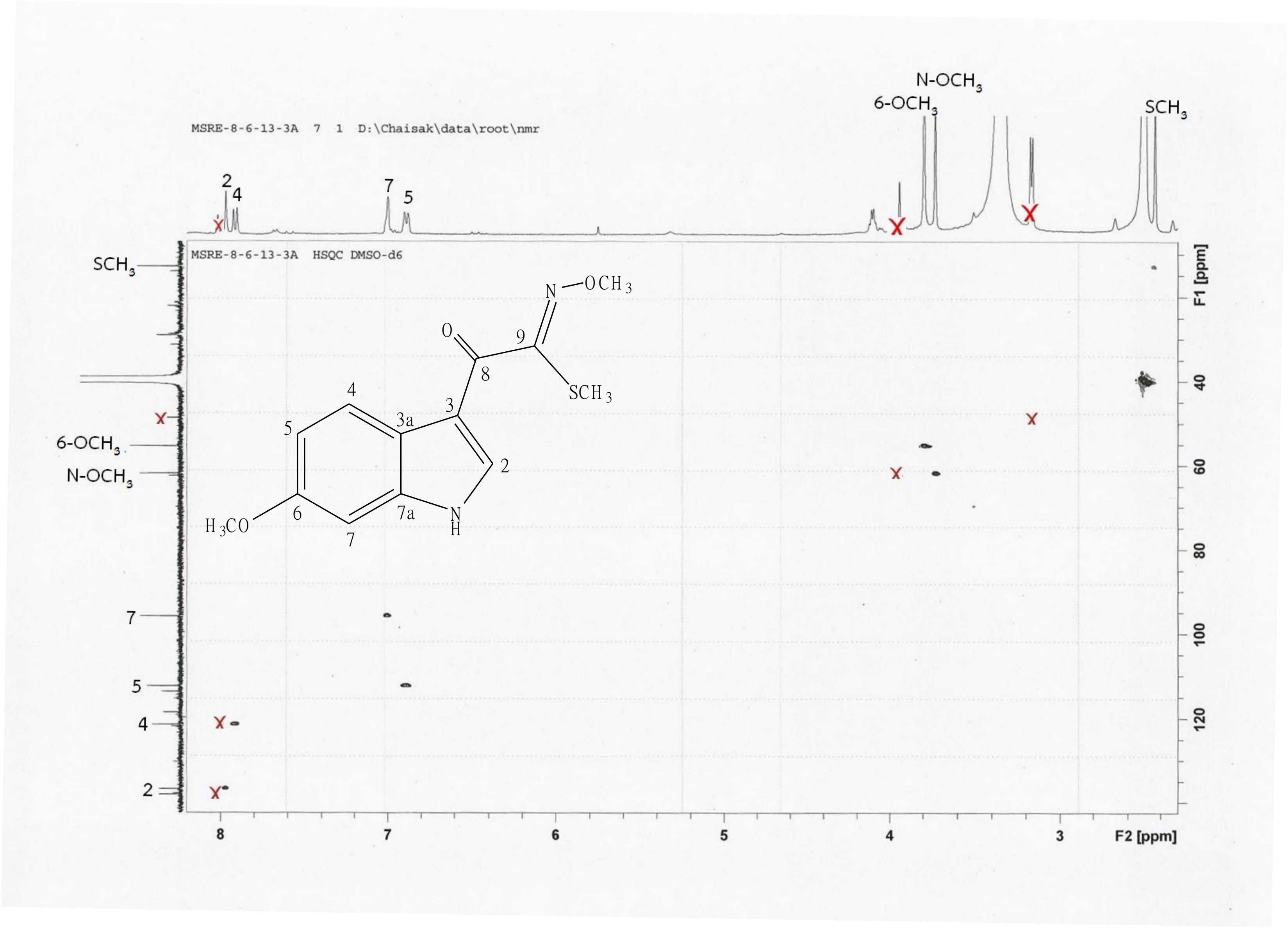

Figure S41. The HSQC spectrum of compound **4**

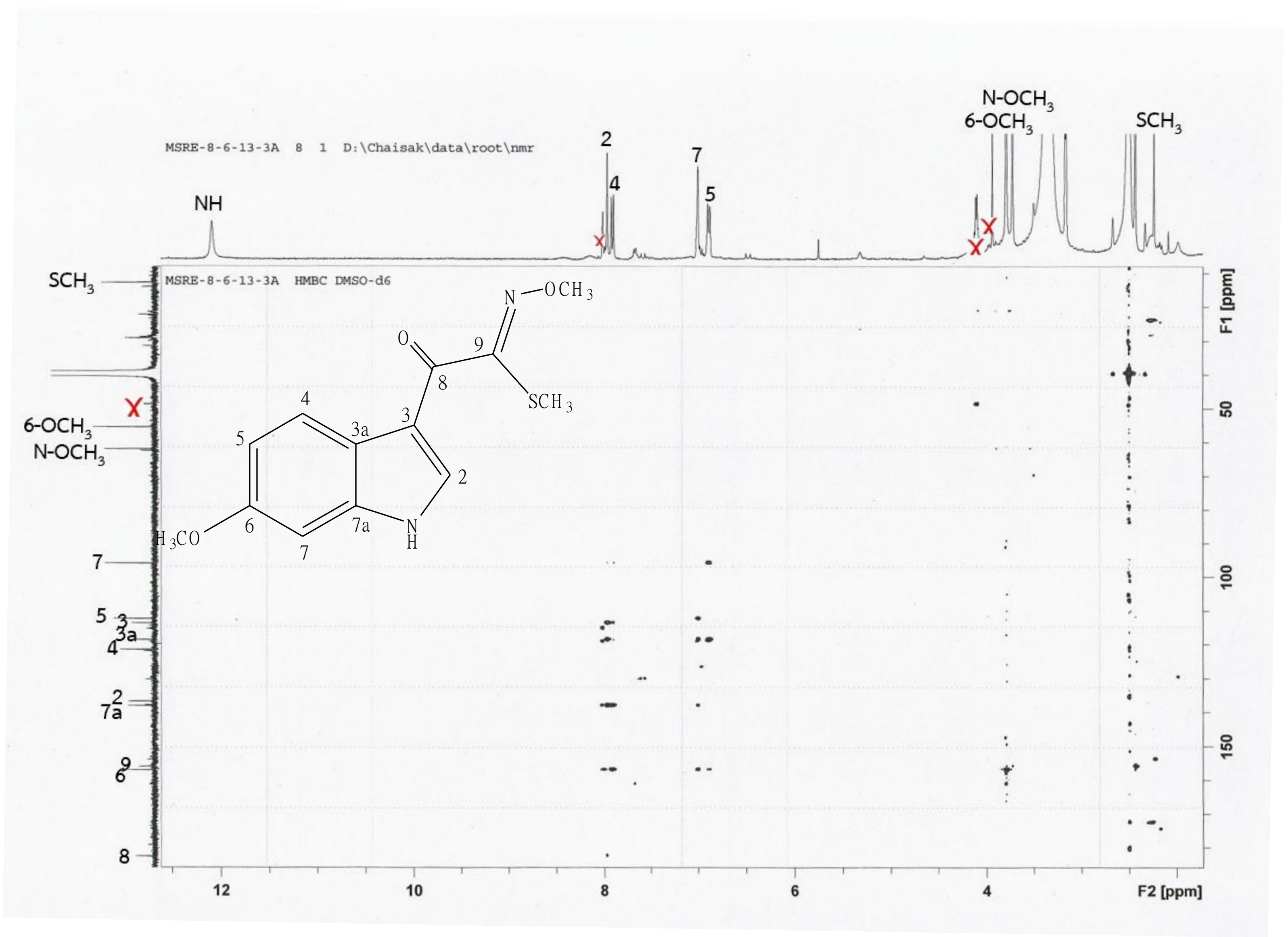

Figure S42. The HMBC spectrum of compound **4**

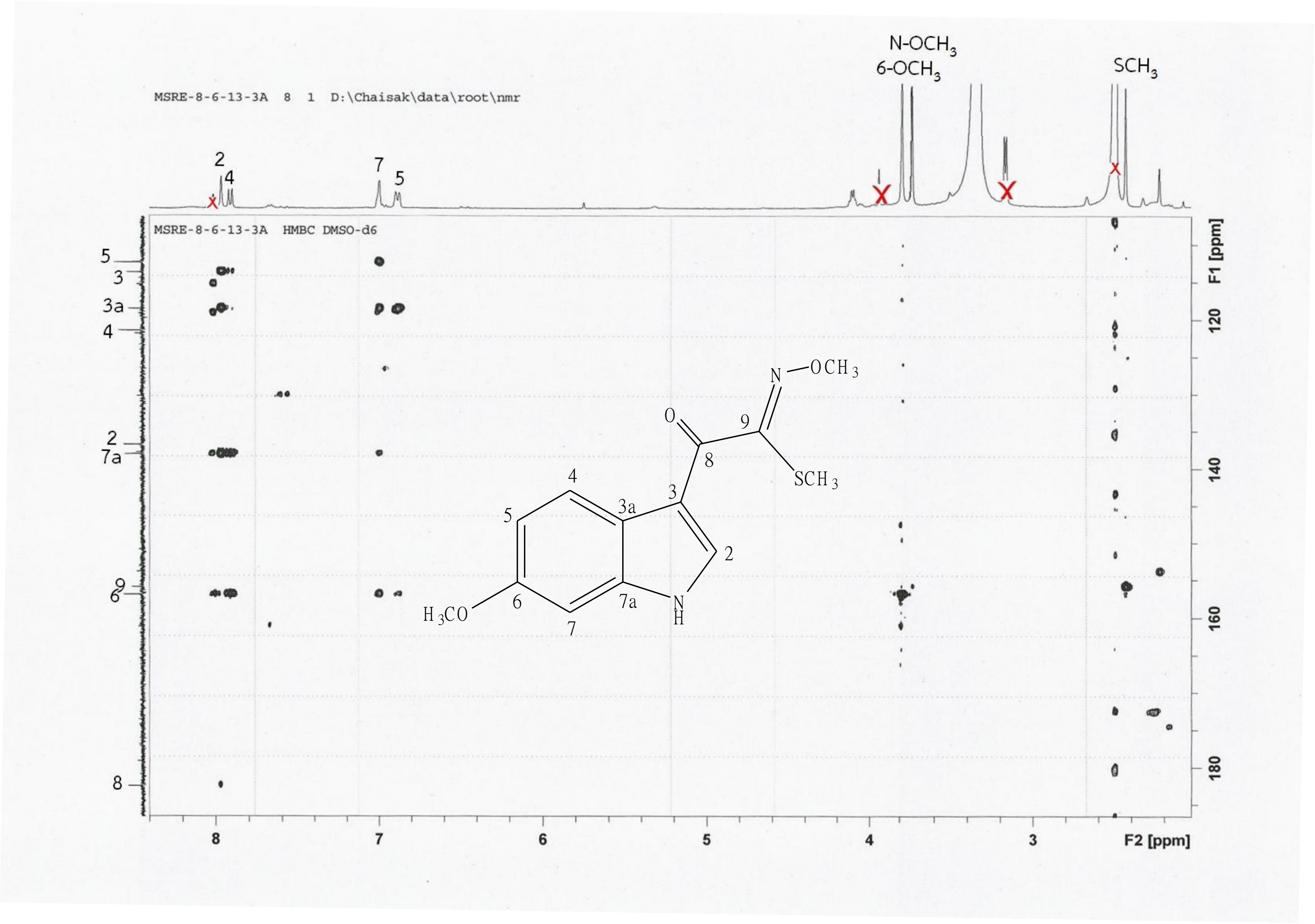

Figure S43. The HMBC spectrum of compound **4 (continued)**

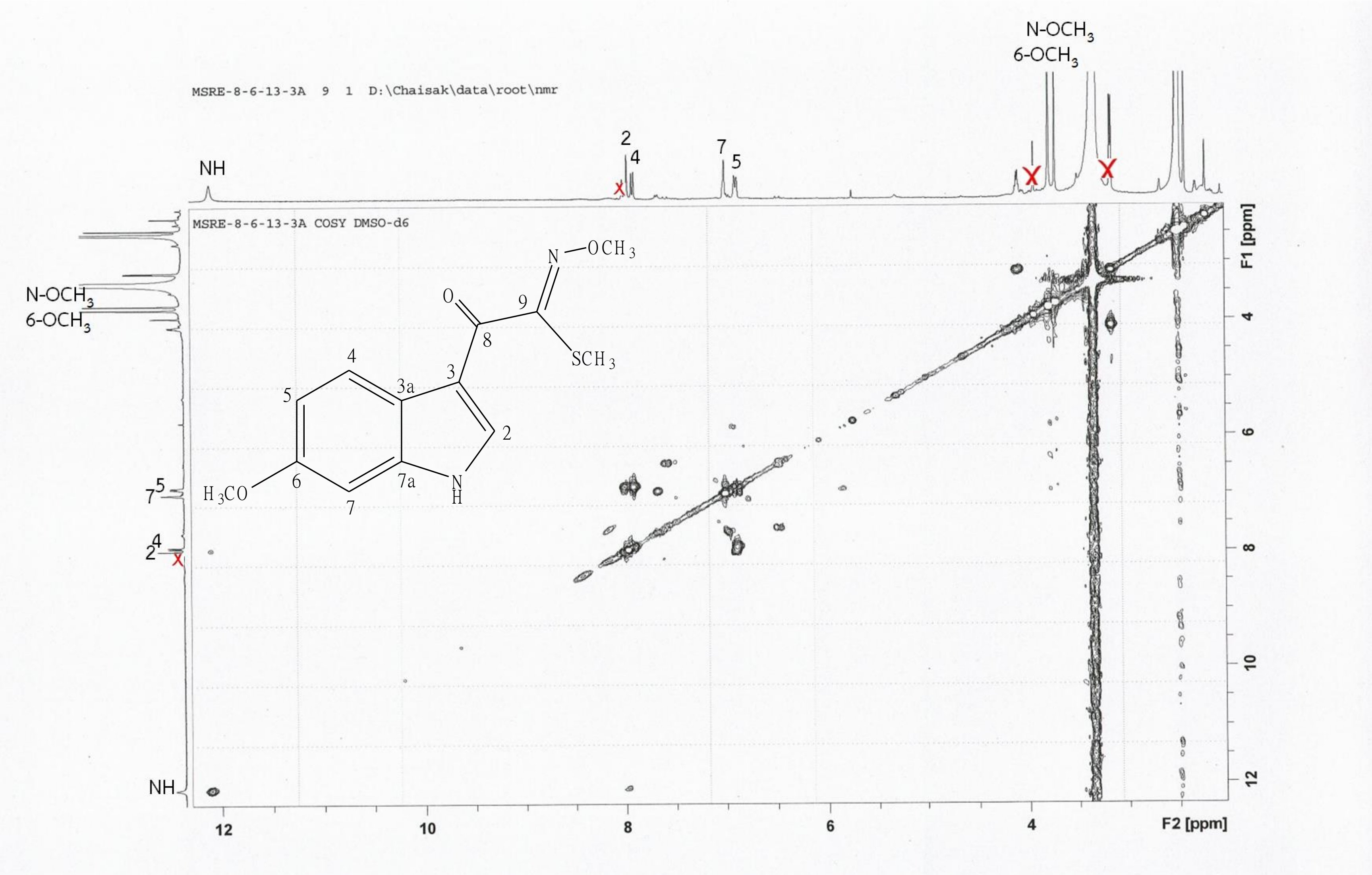

Figure S44. The ^{1}H-^{1}H COSY spectrum of compound **4**

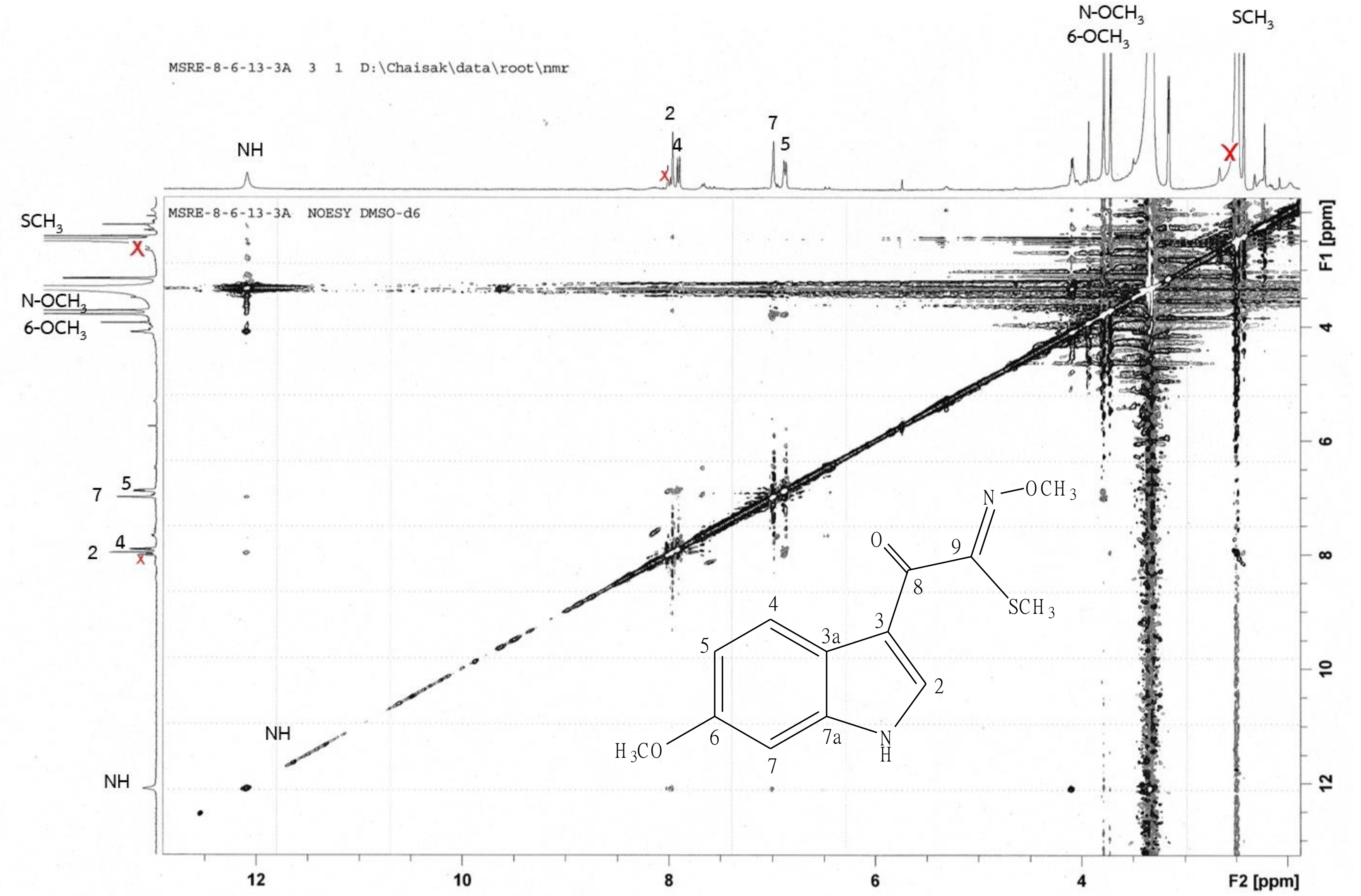

Figure S45. The NOESY spectrum of compound **4**

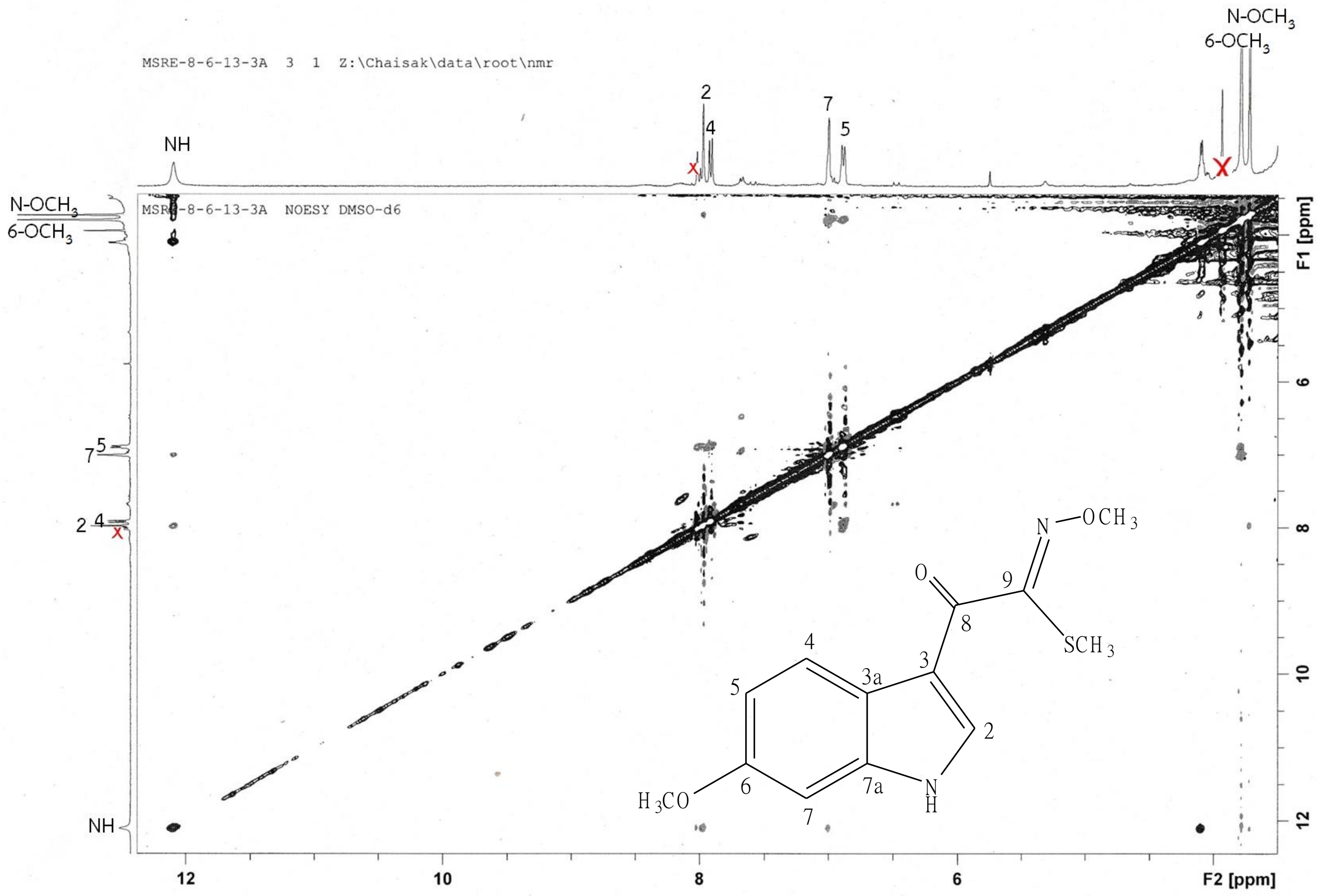

Figure S46. The NOESY spectrum of compound **4 (continued)**

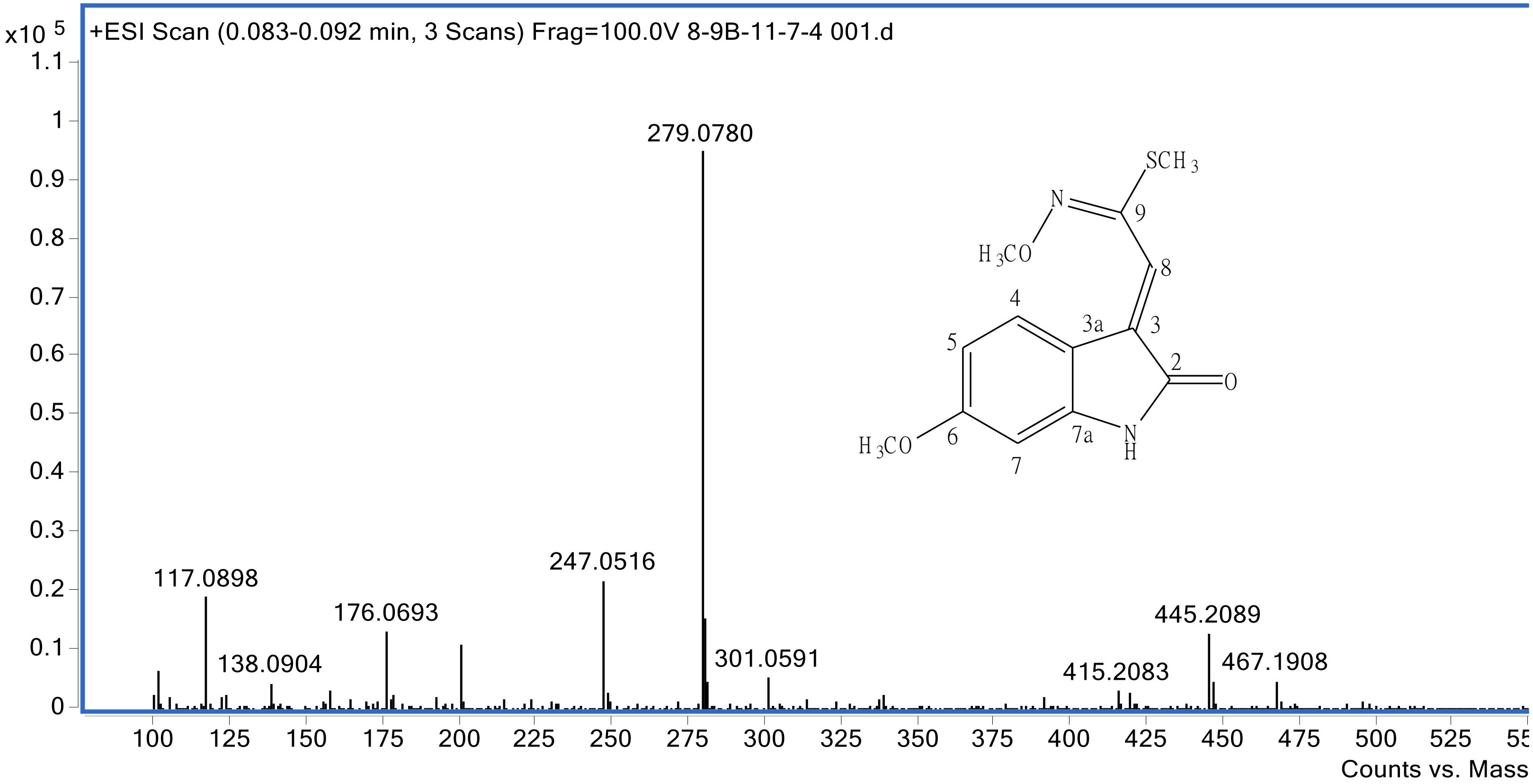

Figure S47. The HR-ESI-MS spectrum of compound **5**

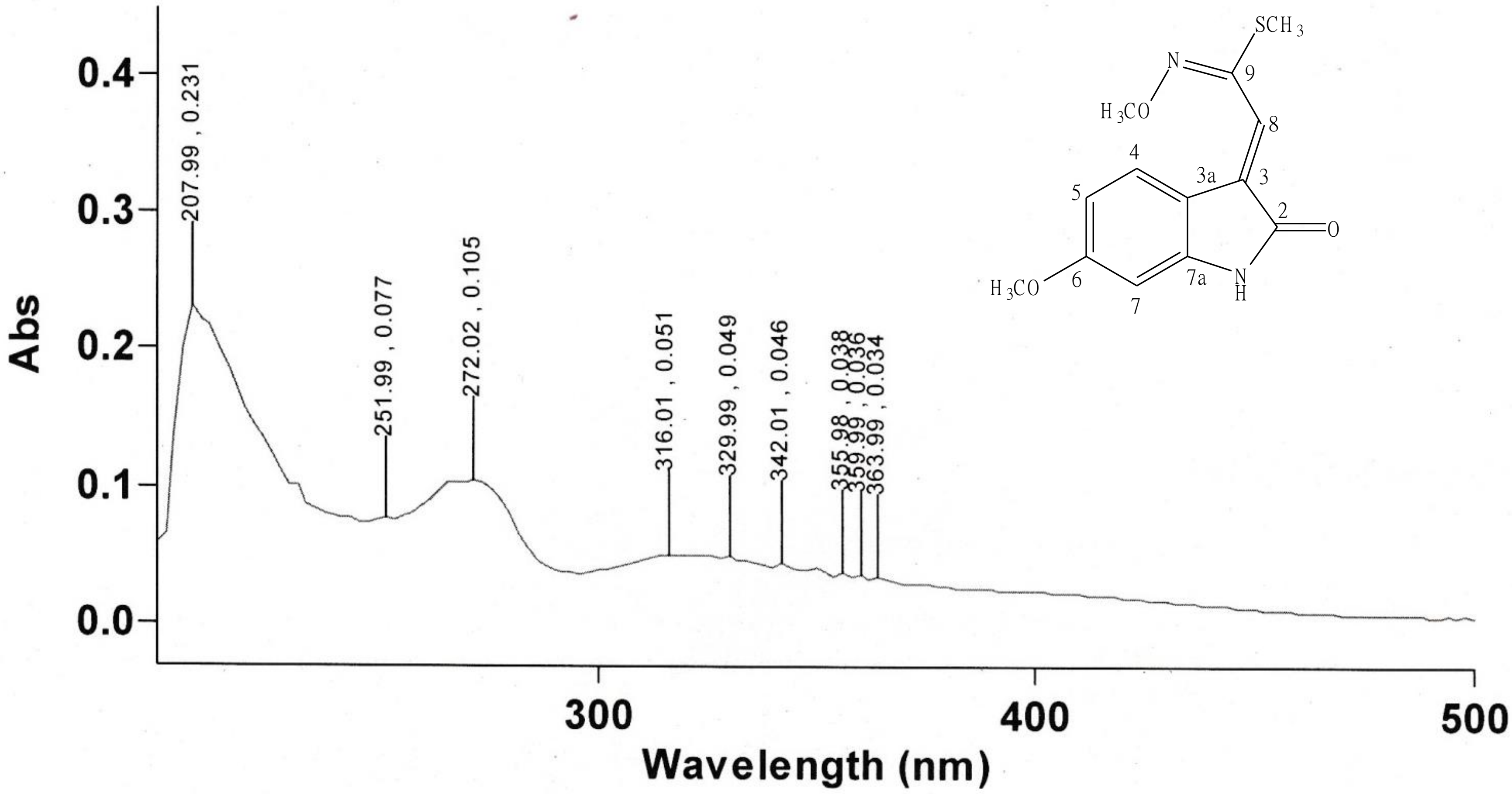

Figure S48. The UV spectrum of compound **5**

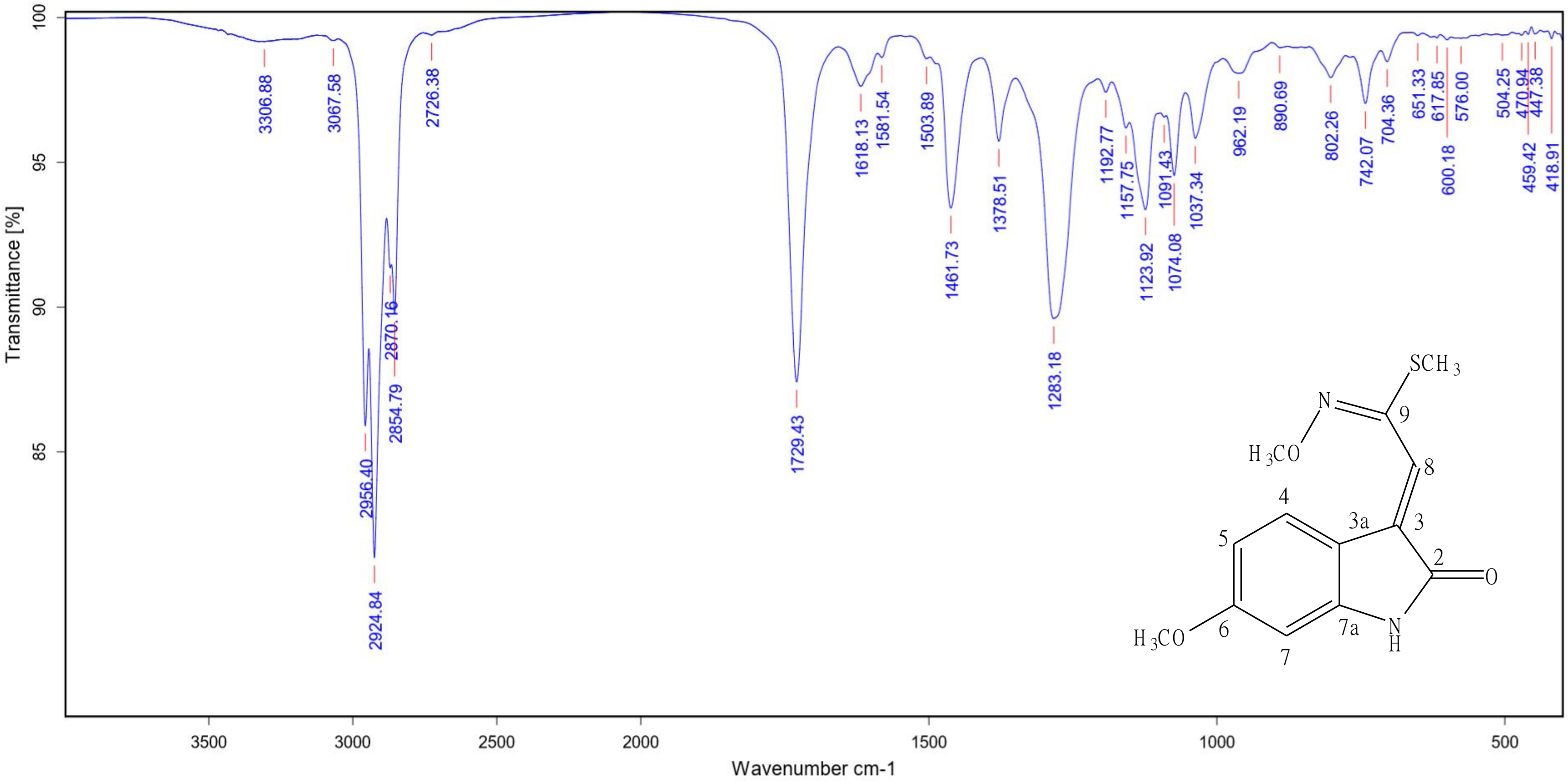

Figure S49. The IR spectrum of compound **5** (ATR)

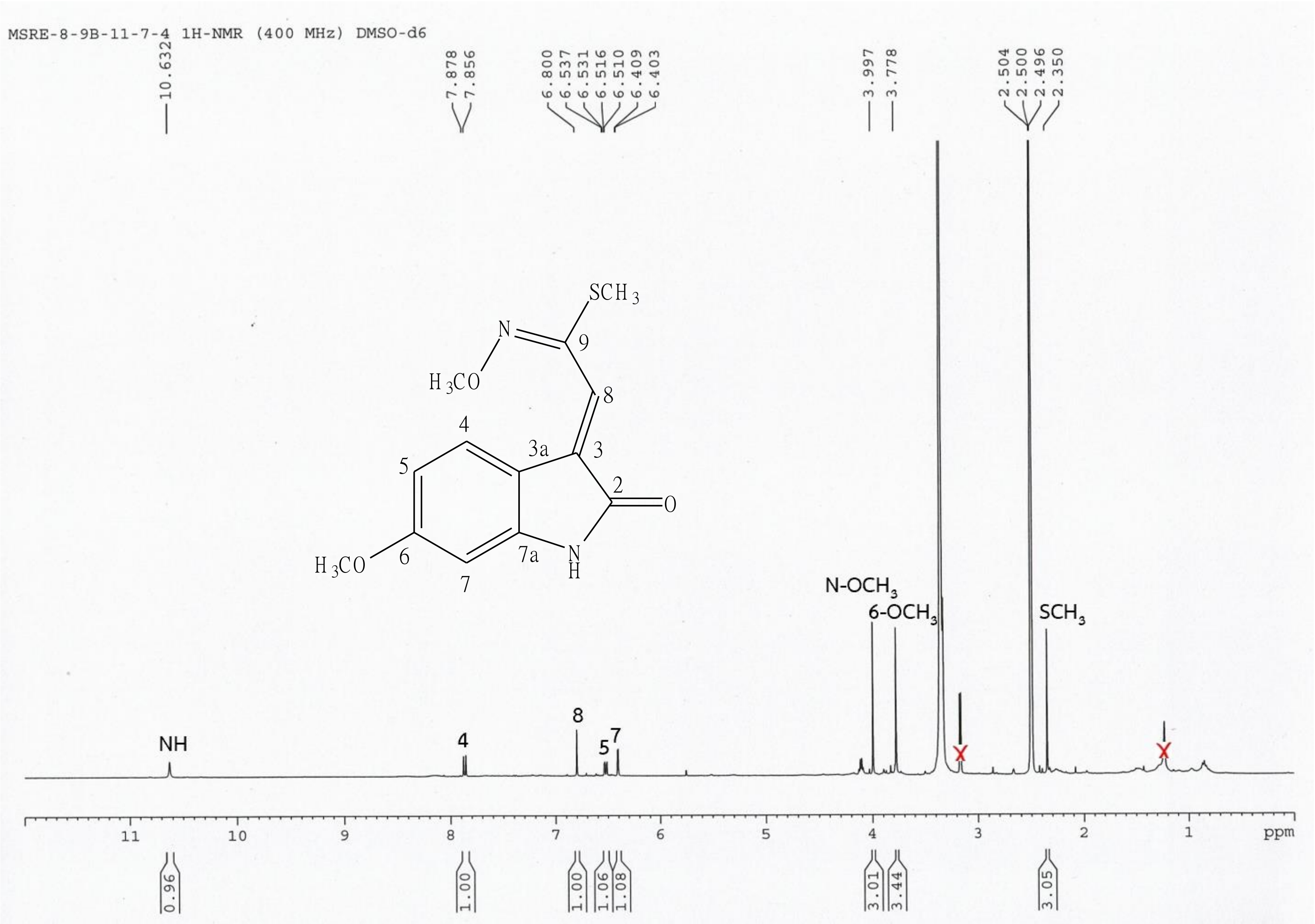

Figure S50. The ^{1}H NMR (400 MHz, DMSO-d_6) spectrum of compound **5**

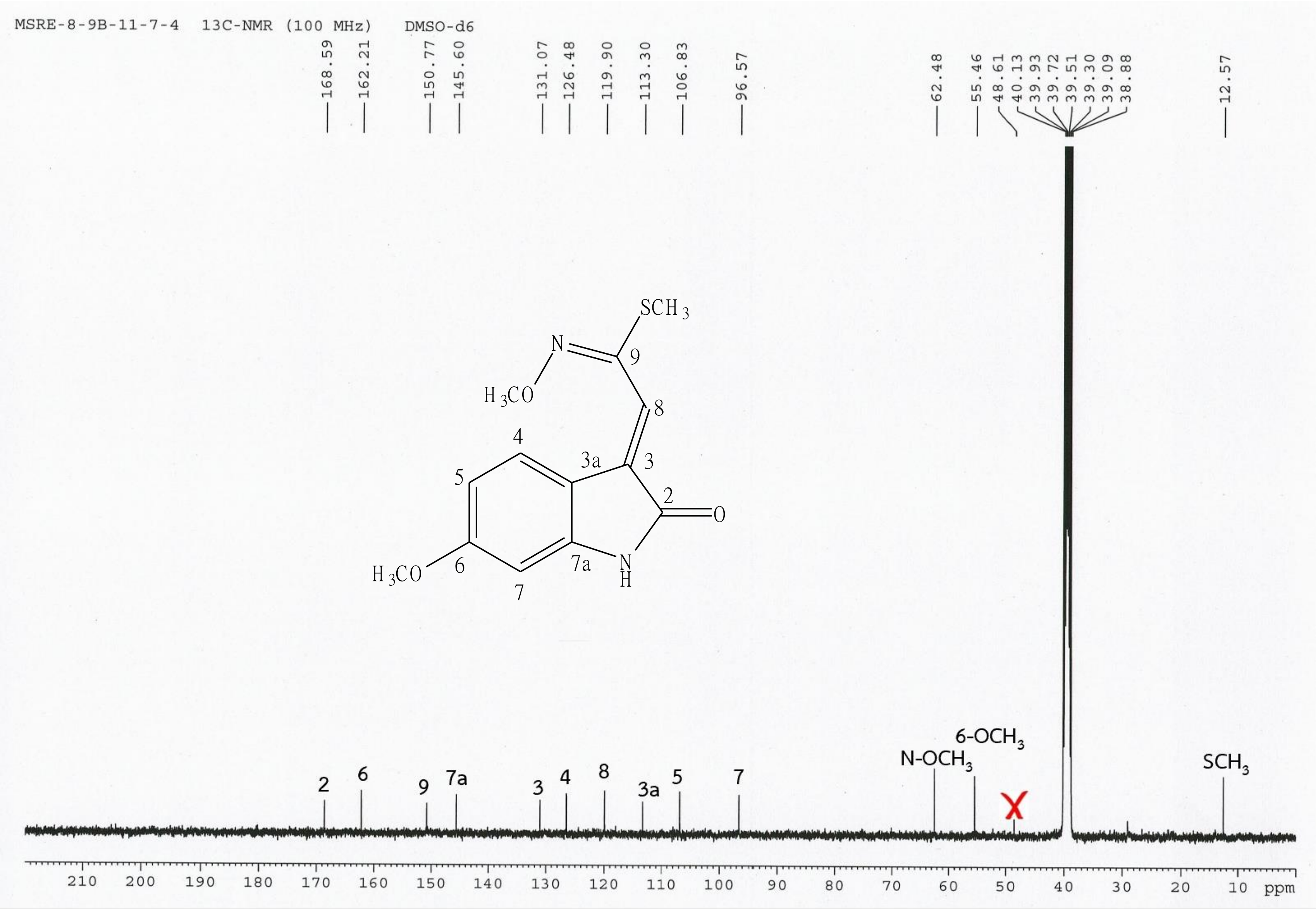

Figure S51. The ^{13}C NMR (100 MHz, DMSO-d_6) spectrum of compound **5**

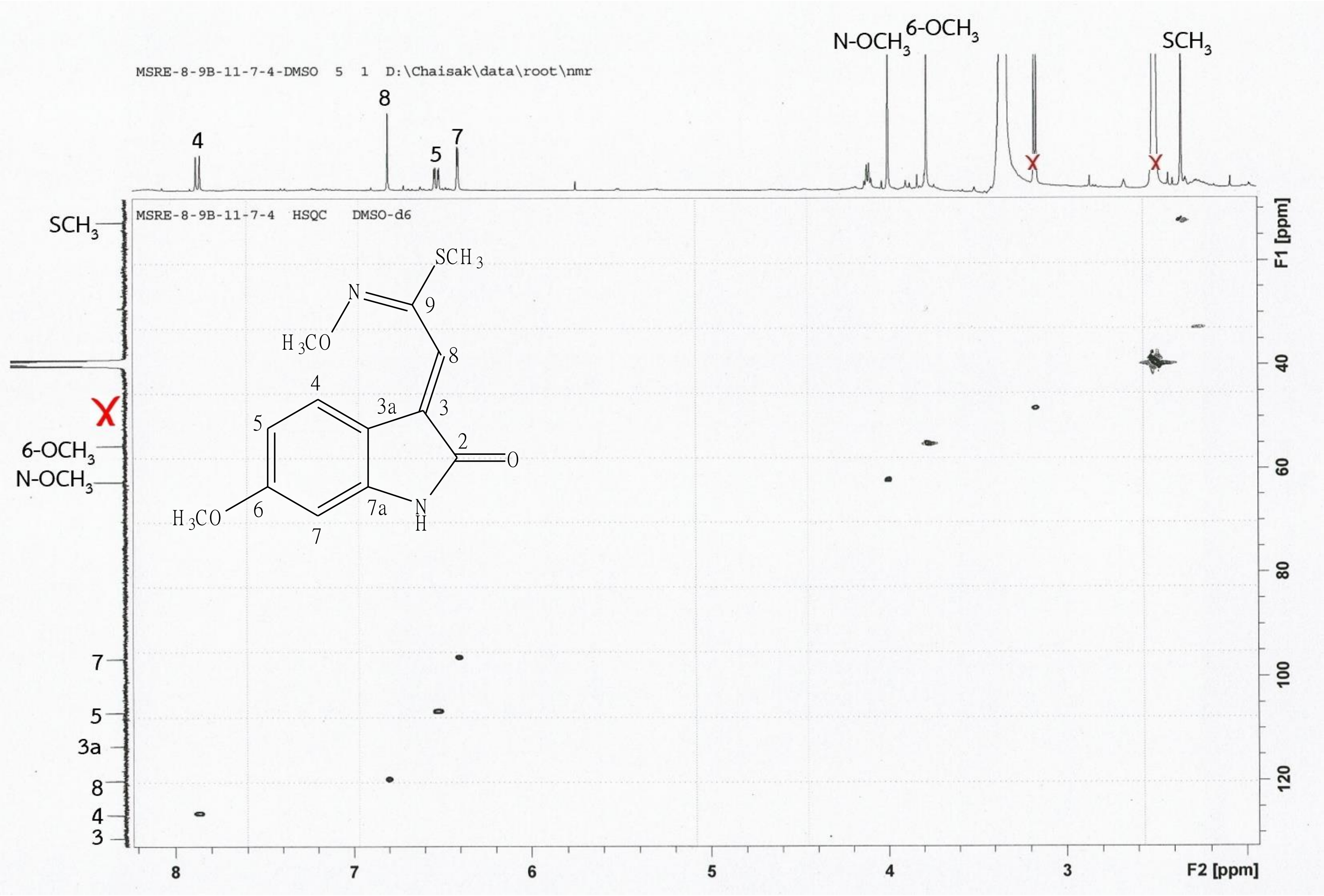

Figure S52. The HSQC spectrum of compound **5**

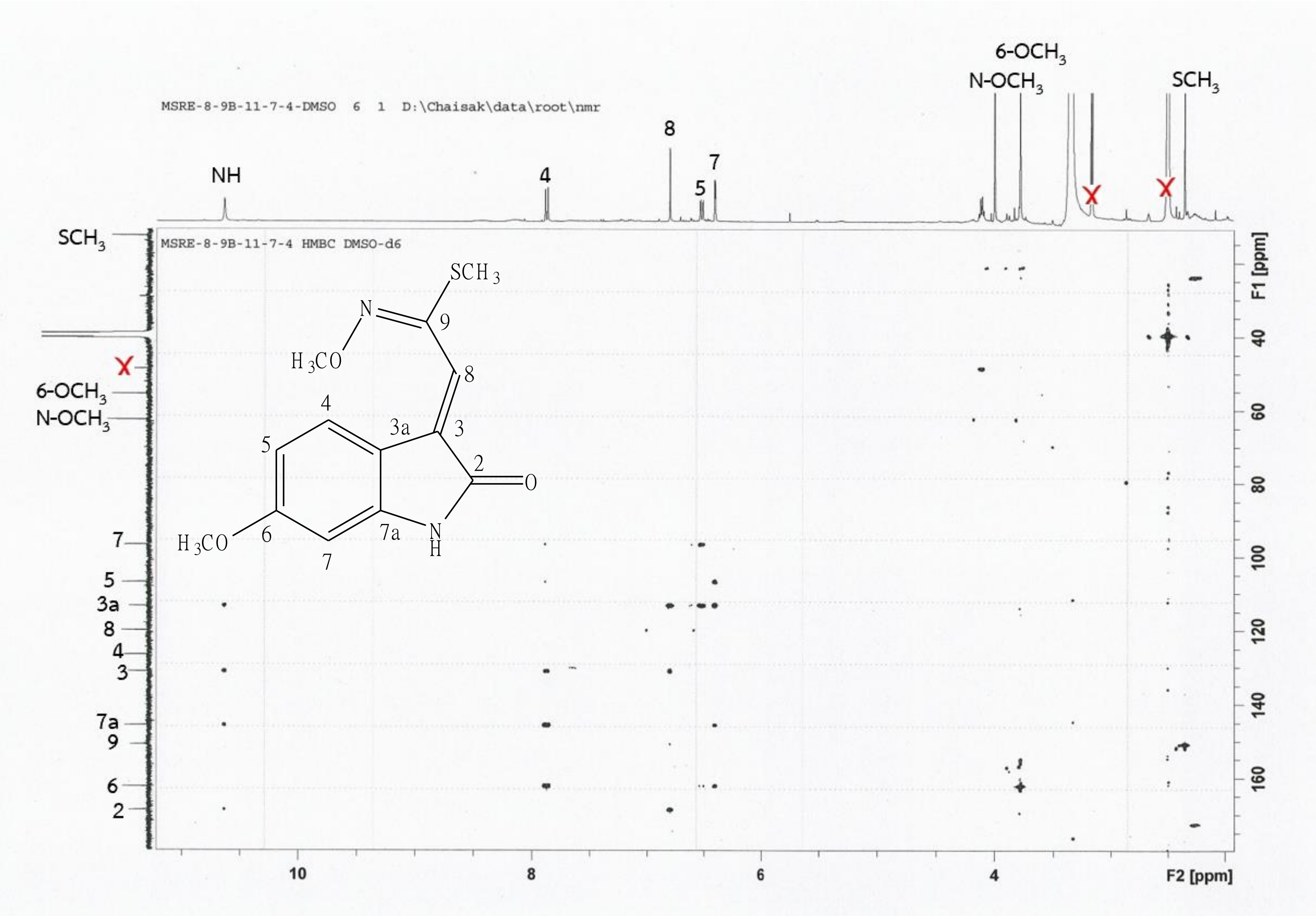

Figure S53. The HMBC spectrum of compound **5**

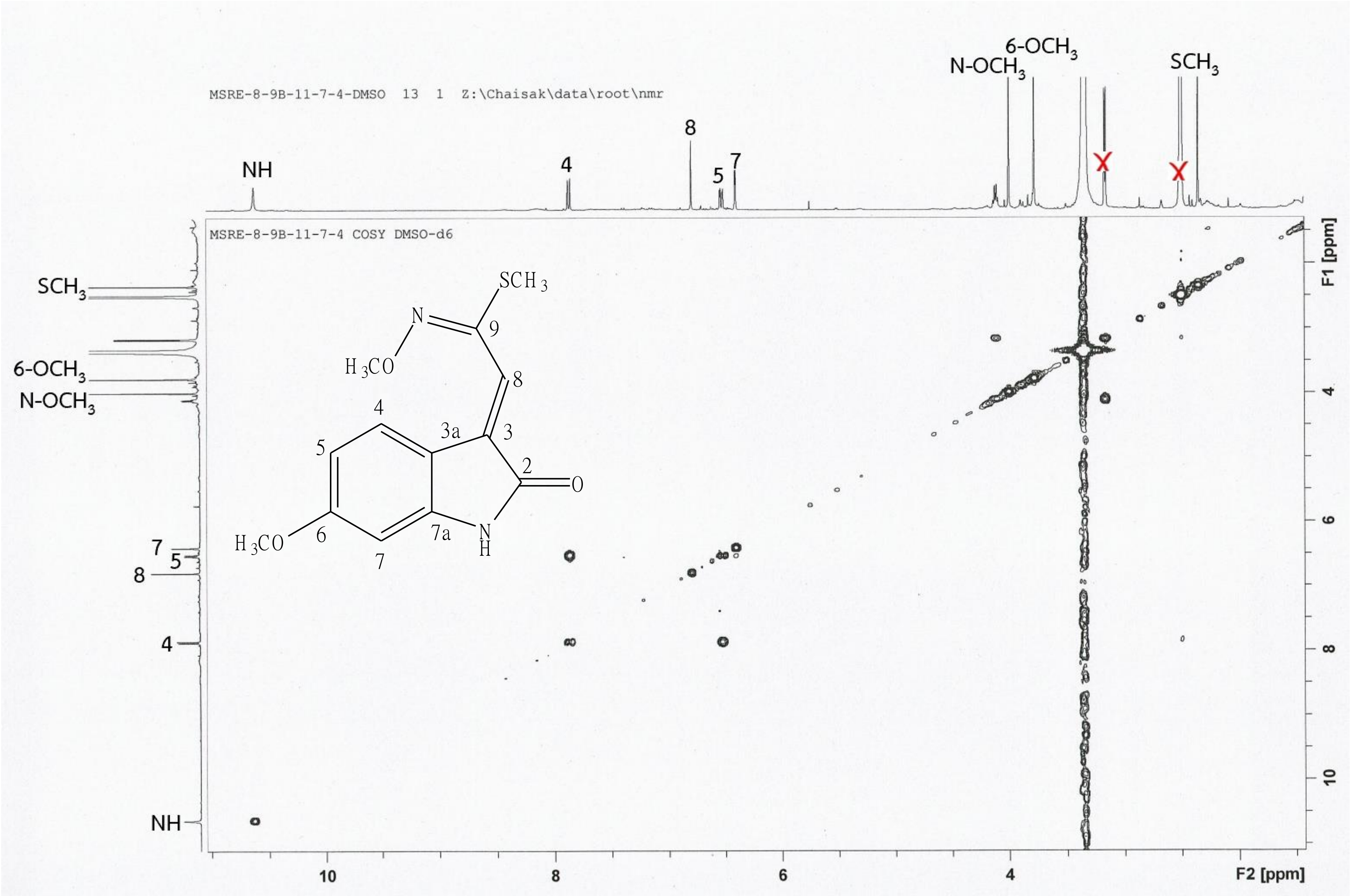

Figure S54. The ^{1}H-^{1}H COSY spectrum of compound **5**

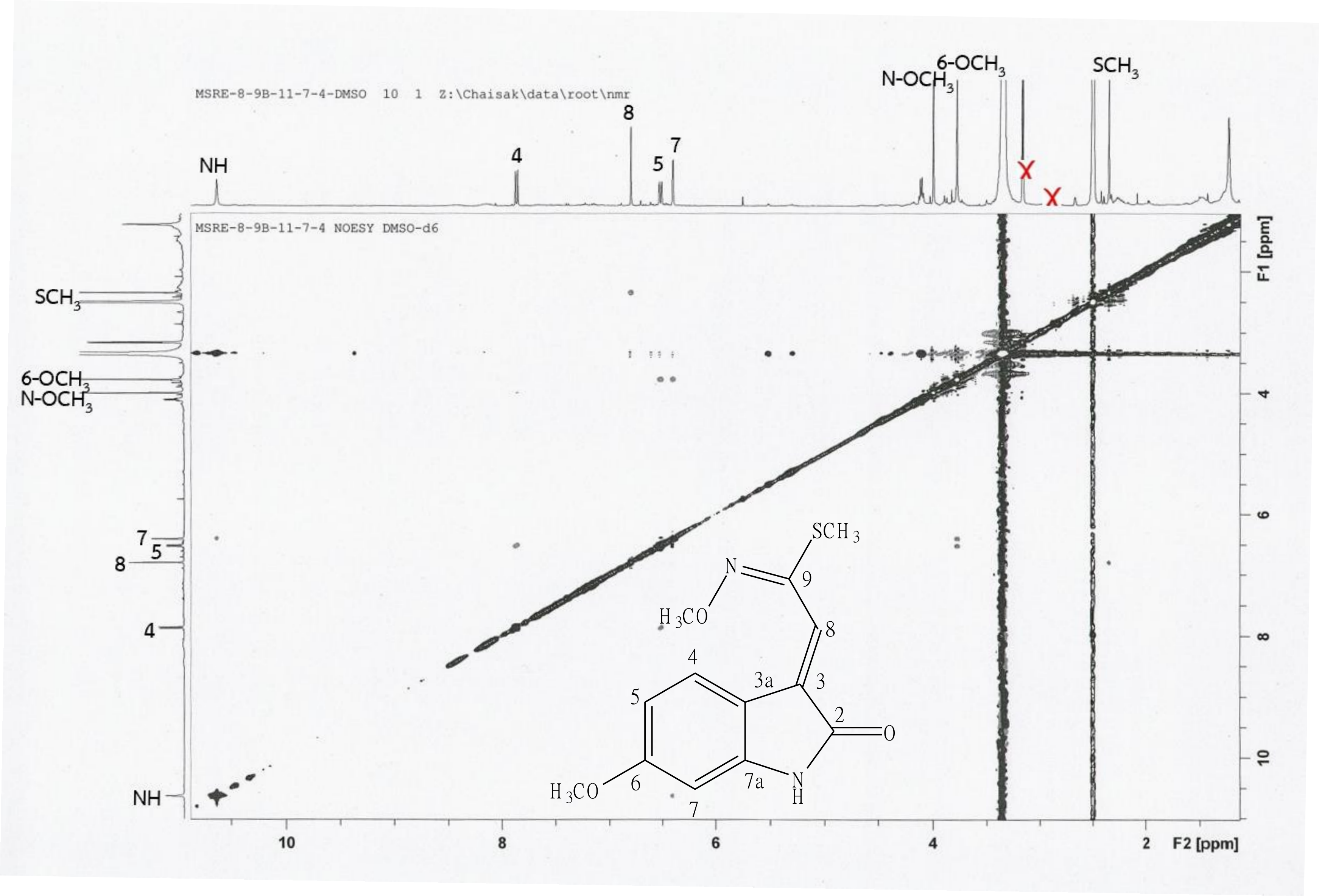

Figure S55. The NOESY spectrum of compound **5**

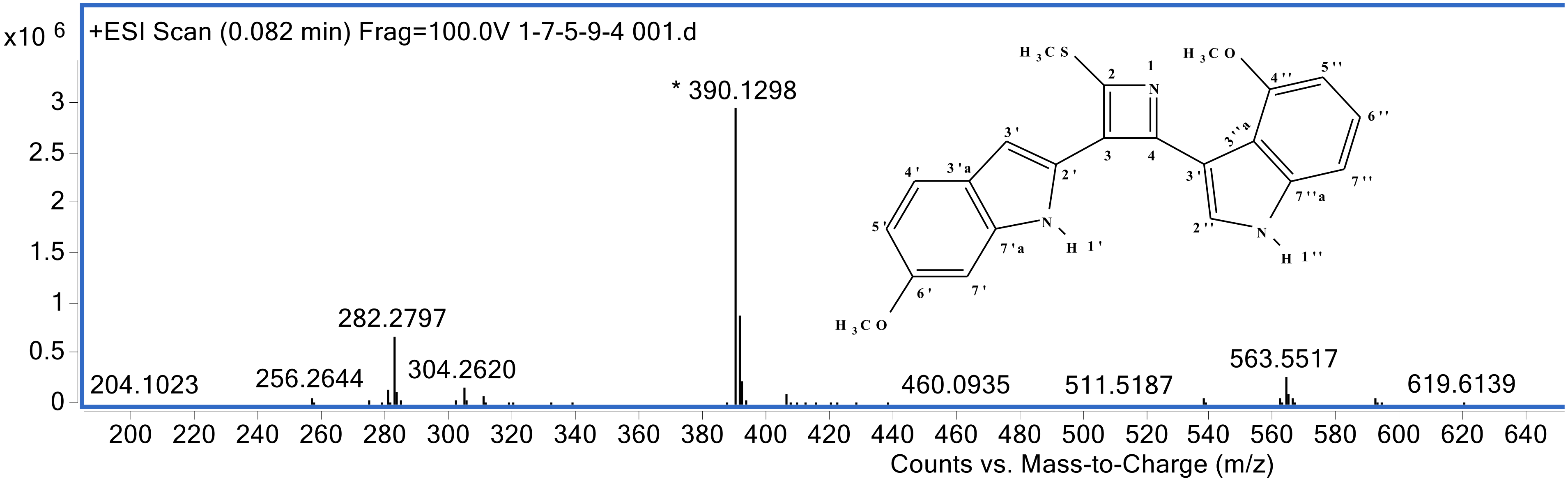

Figure S56. The HR-ESI-MS spectrum of compound **6**

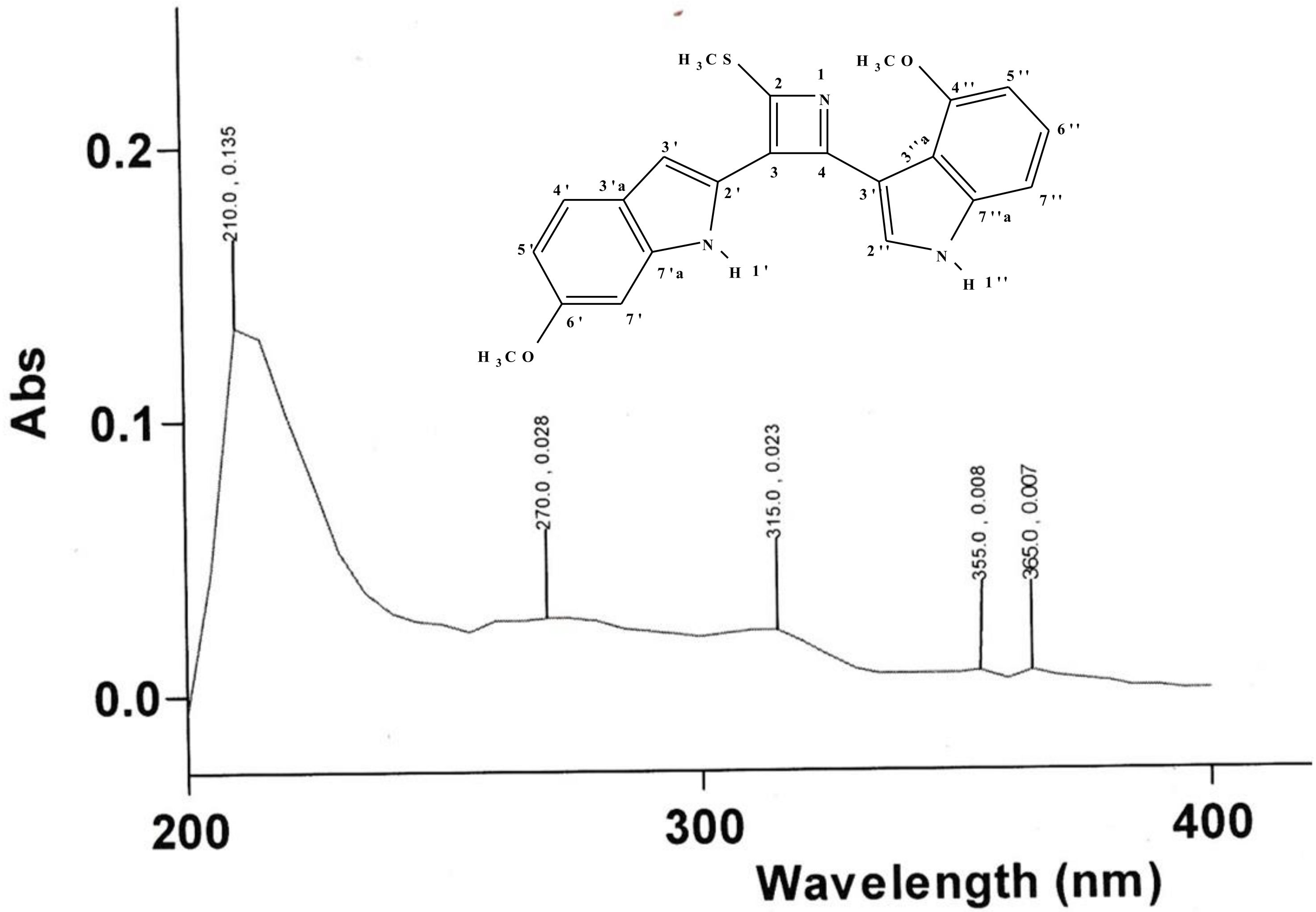

Figure S57. The UV spectrum of compound **6**

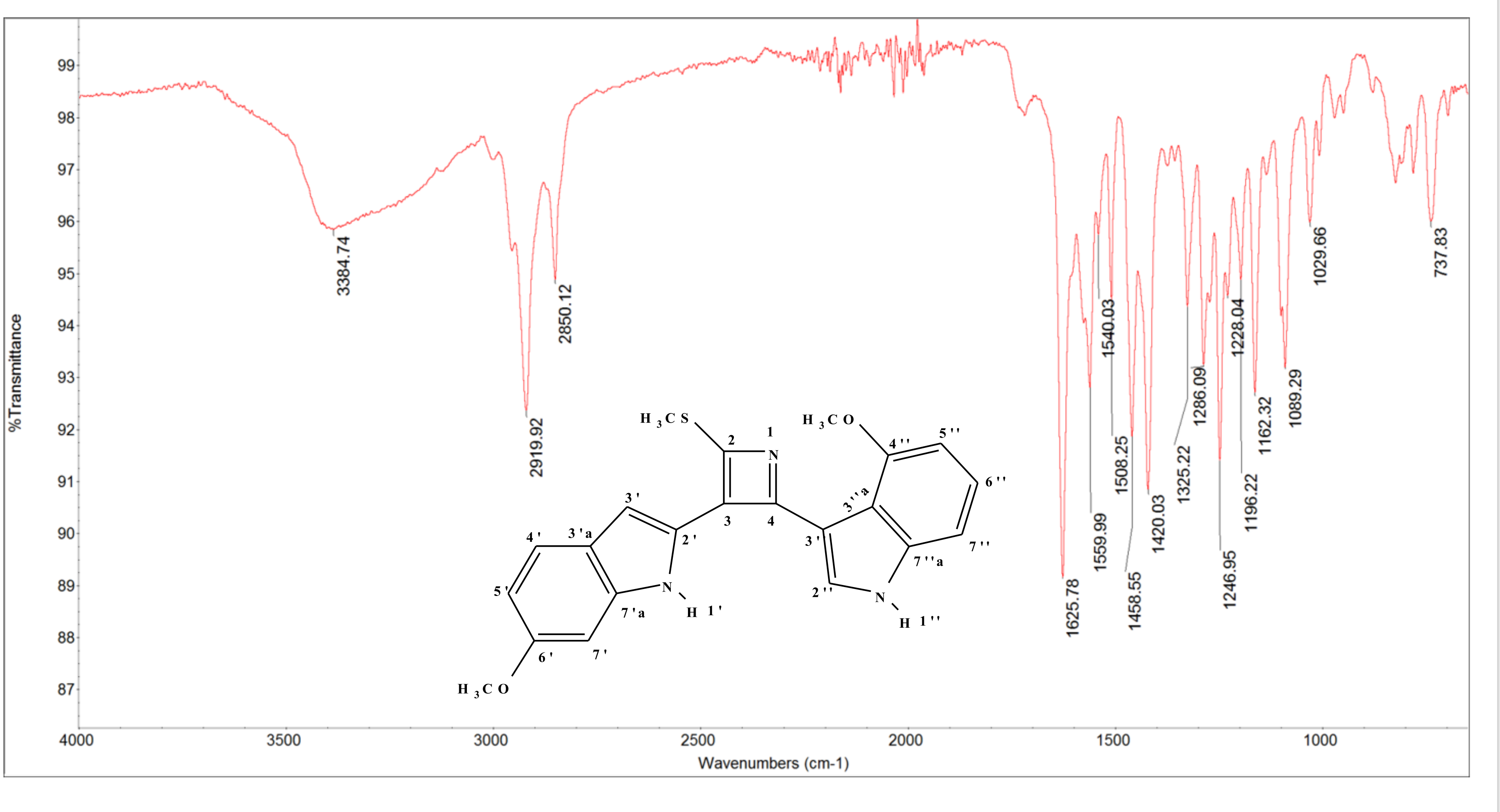

Figure S58. The IR spectrum of compound **6** (ATR)

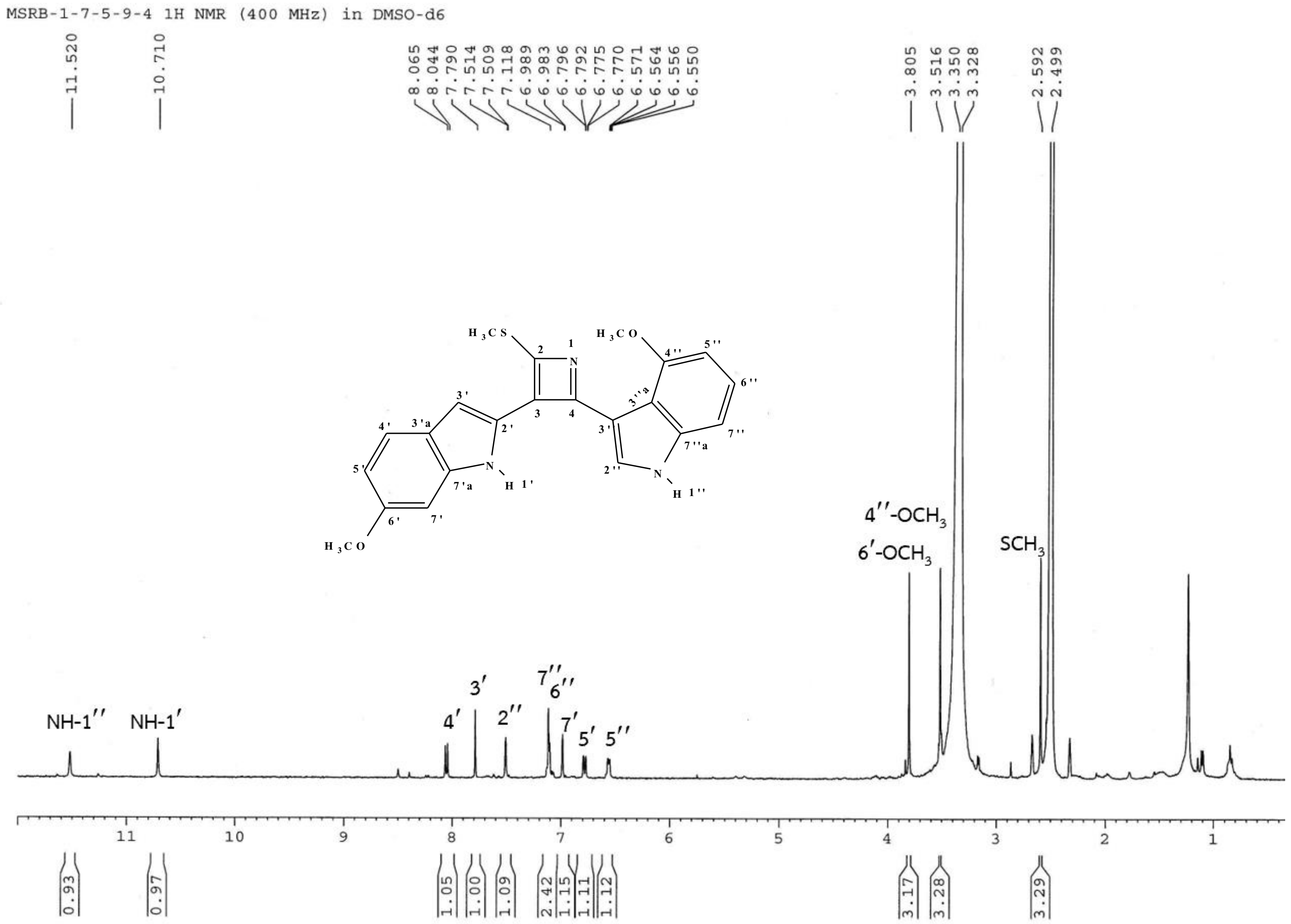

Figure S59. The ^{1}H NMR (400 MHz, DMSO-d_6) spectrum of compound **6**

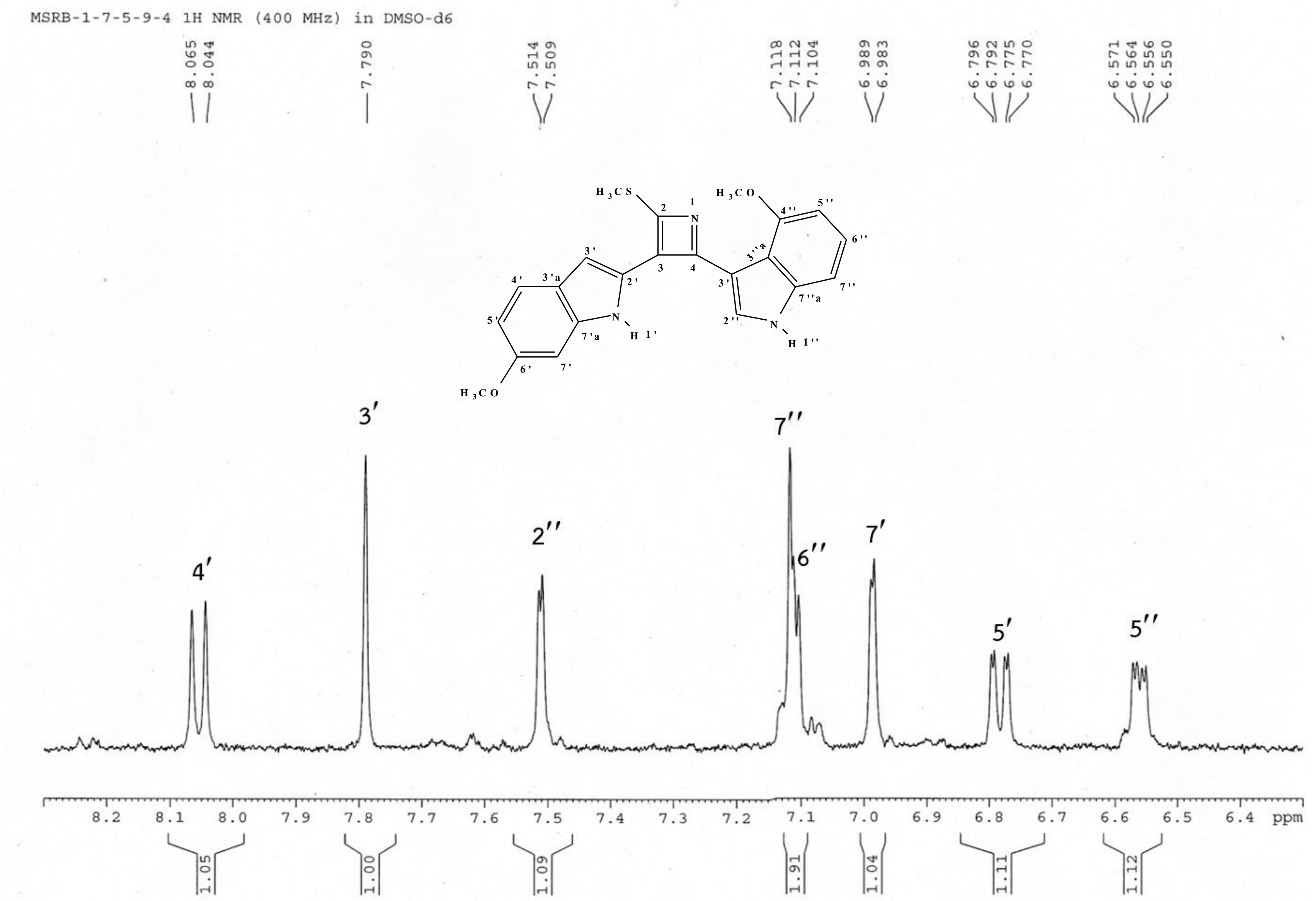

Figure S60. The ^{1}H NMR (400 MHz, DMSO-d_6) spectrum of compound **6 (continued)**

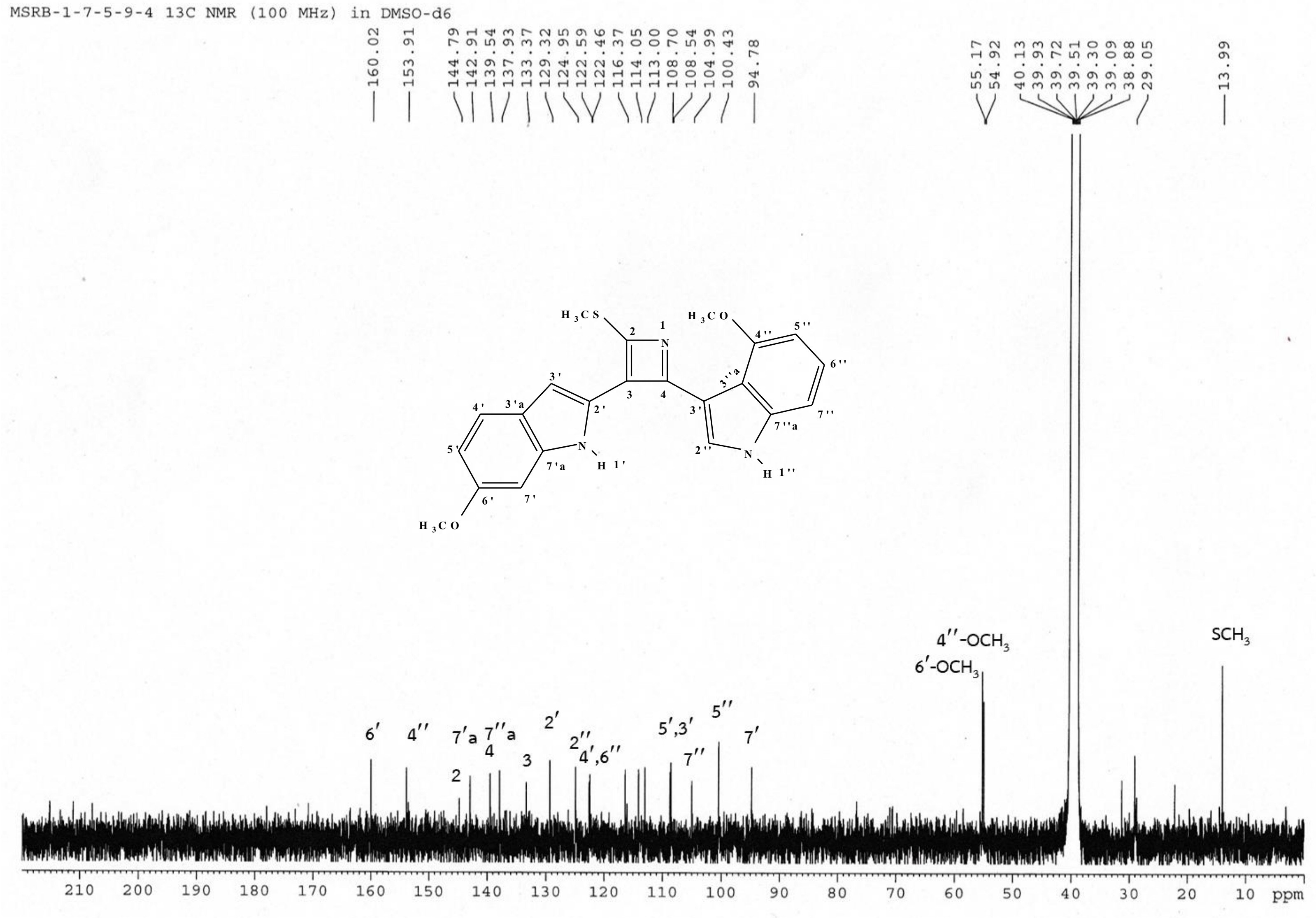

Figure S61. The ^{13}C NMR (100 MHz, DMSO-d_6) spectrum of compound **6**

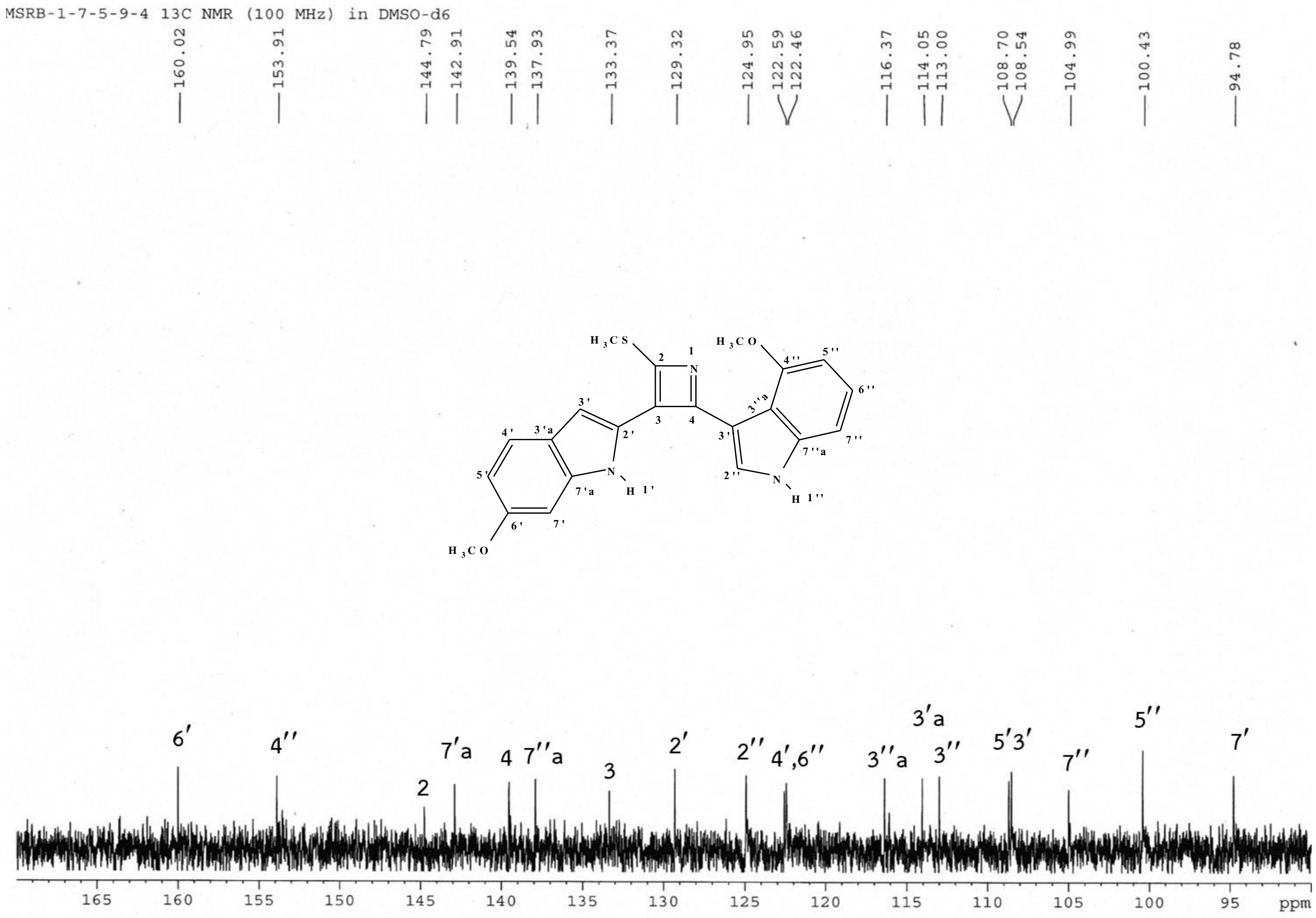

Figure S62. The ^{13}C NMR (100 MHz, DMSO-d_6) spectrum of compound **6 (continued)**

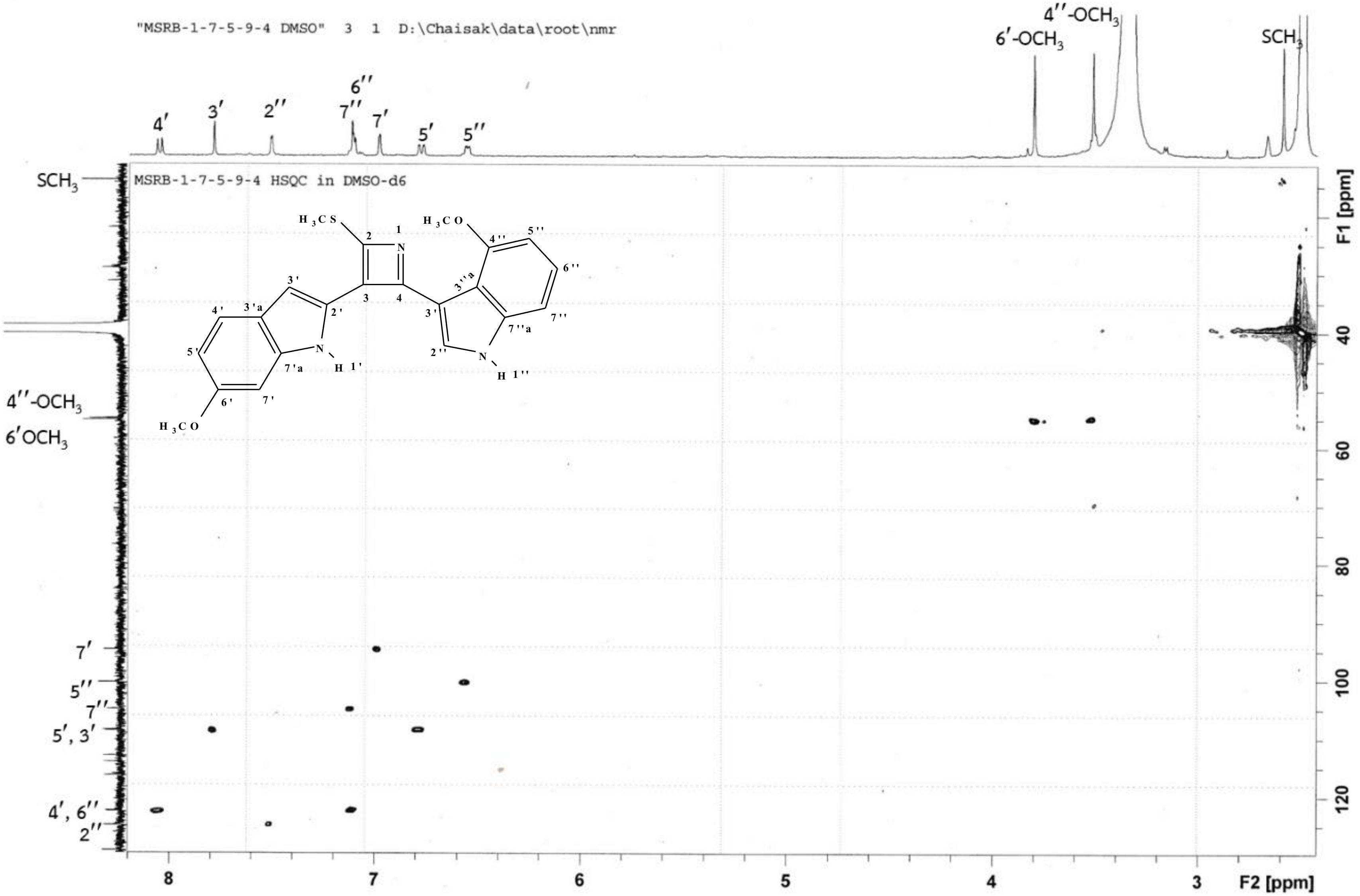

Figure S63. The HSQC spectrum of compound **6**

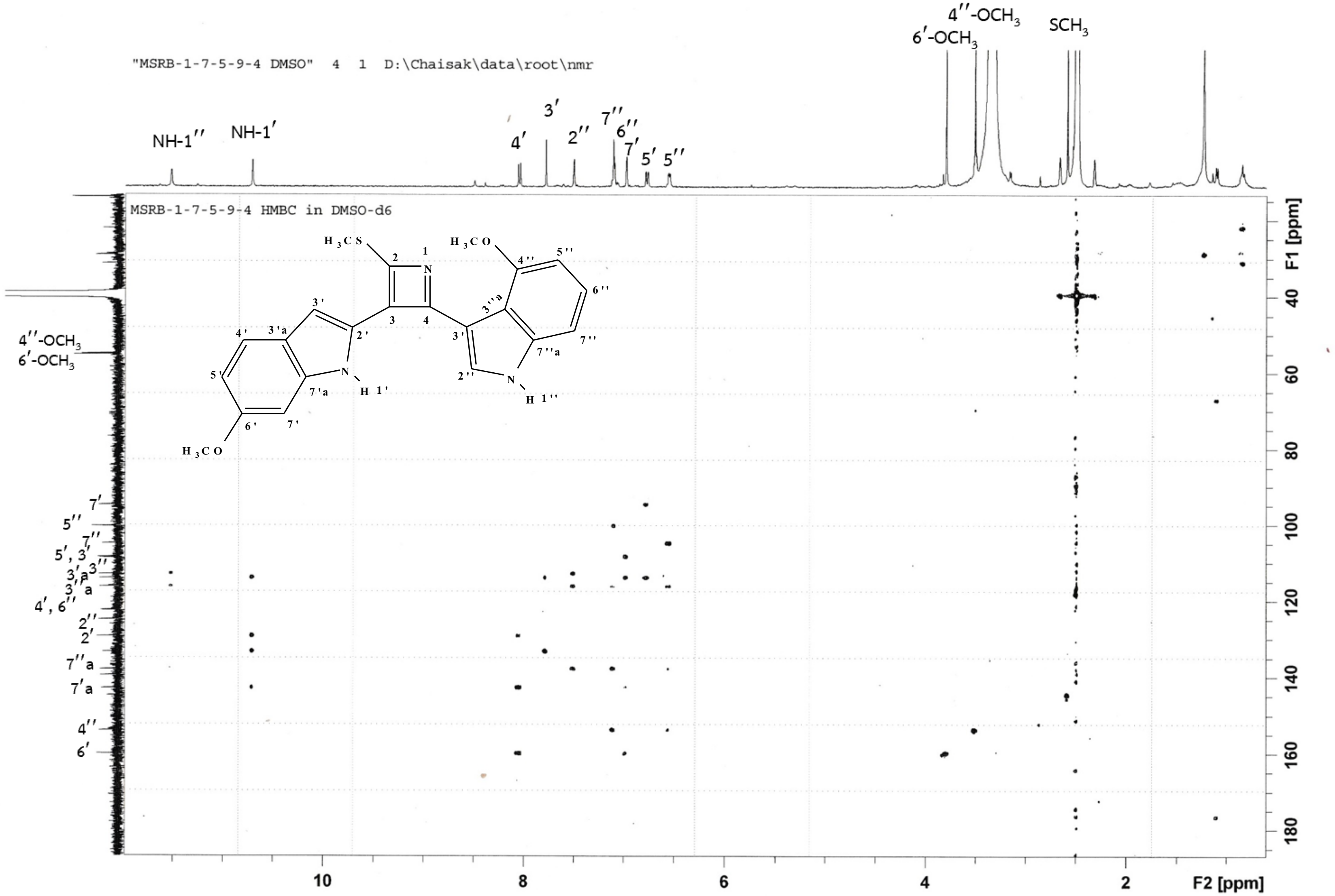

Figure S64. The HMBC spectrum of compound **6**

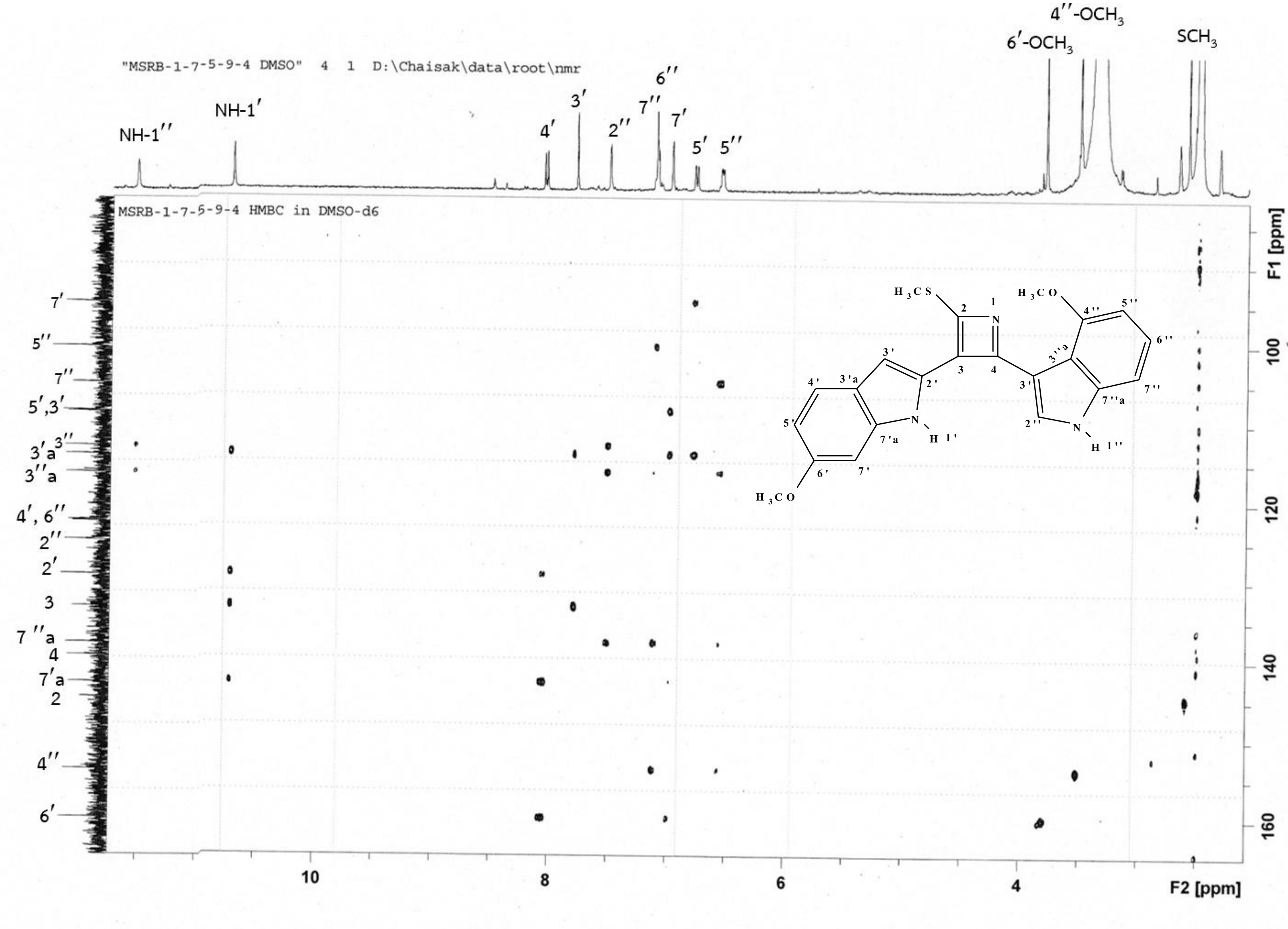

Figure S65. The HMBC spectrum of compound **6 (continued)**

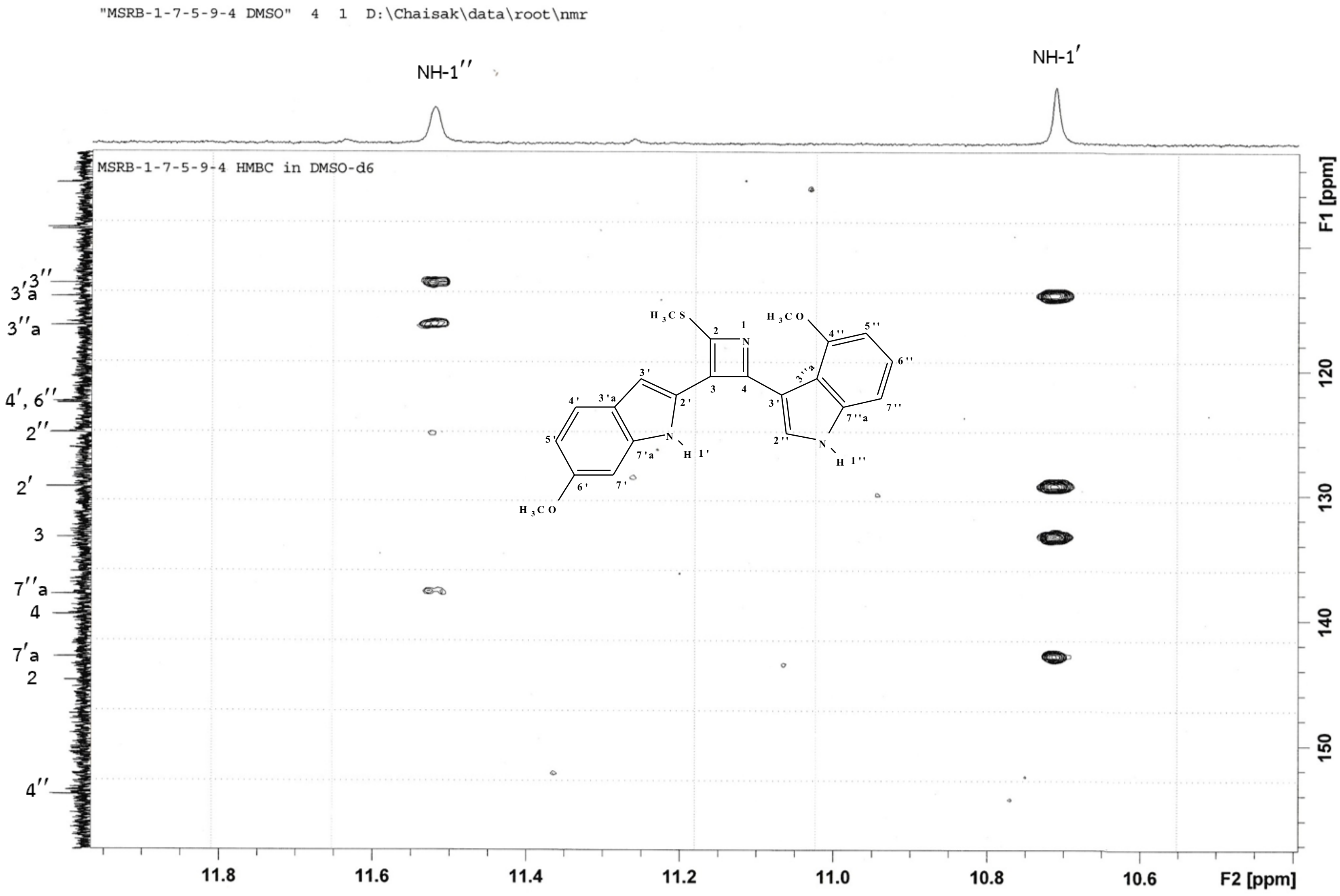

Figure S66. The HMBC spectrum of compound **6 (continued)**

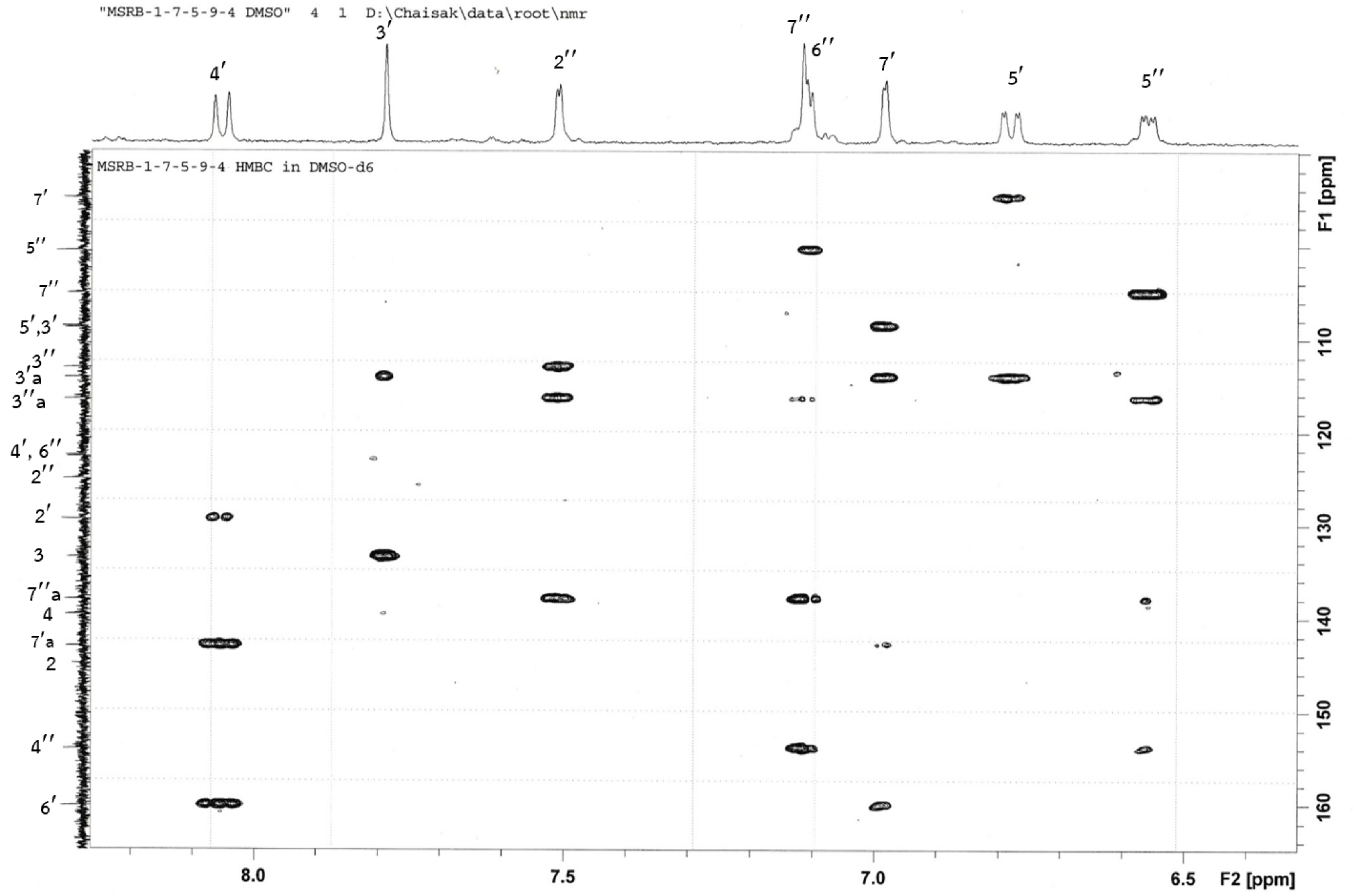

Figure S67. The HMBC spectrum of compound **6 (continued)**

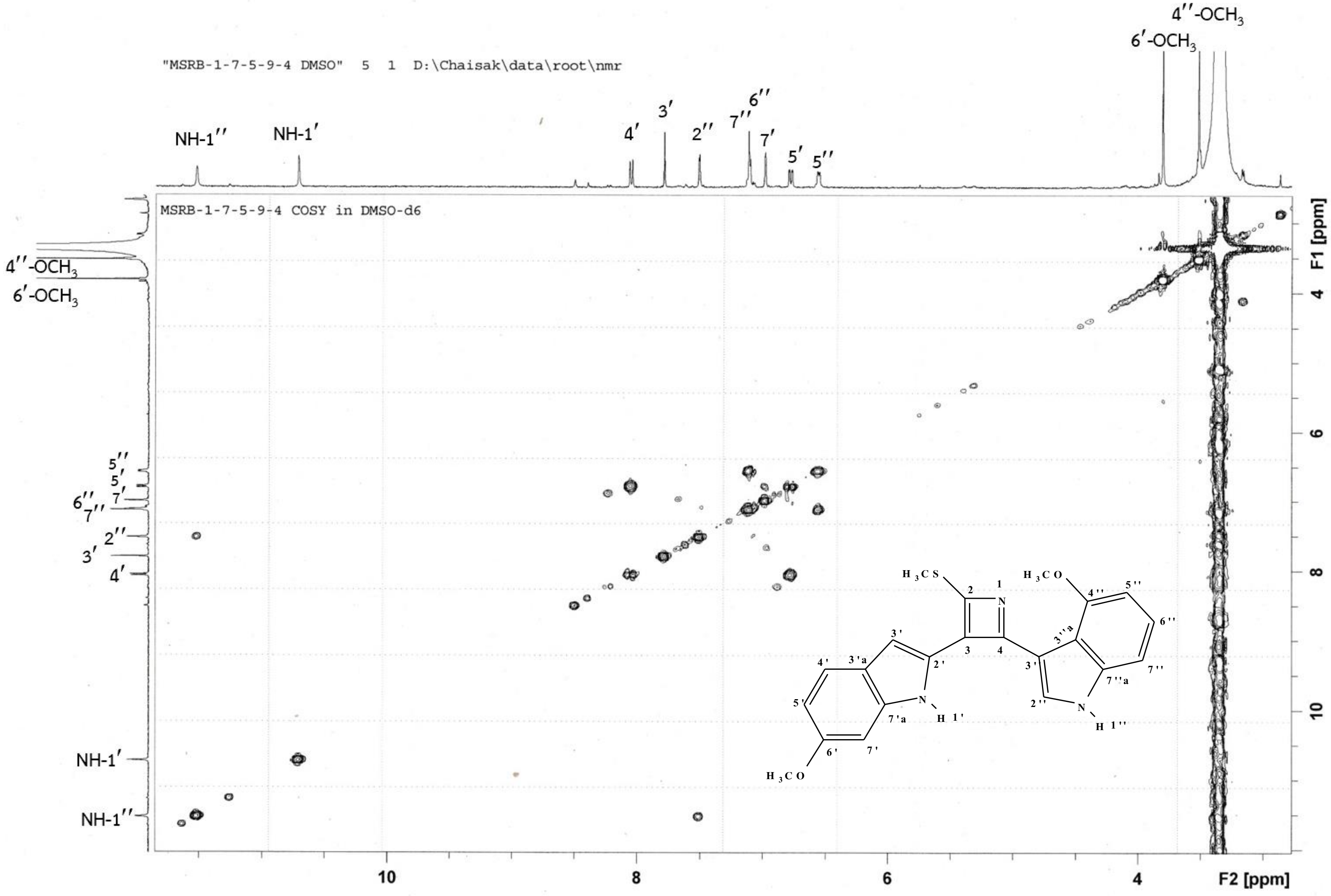

Figure S68. The ^{1}H-^{1}H COSY spectrum of compound **6**

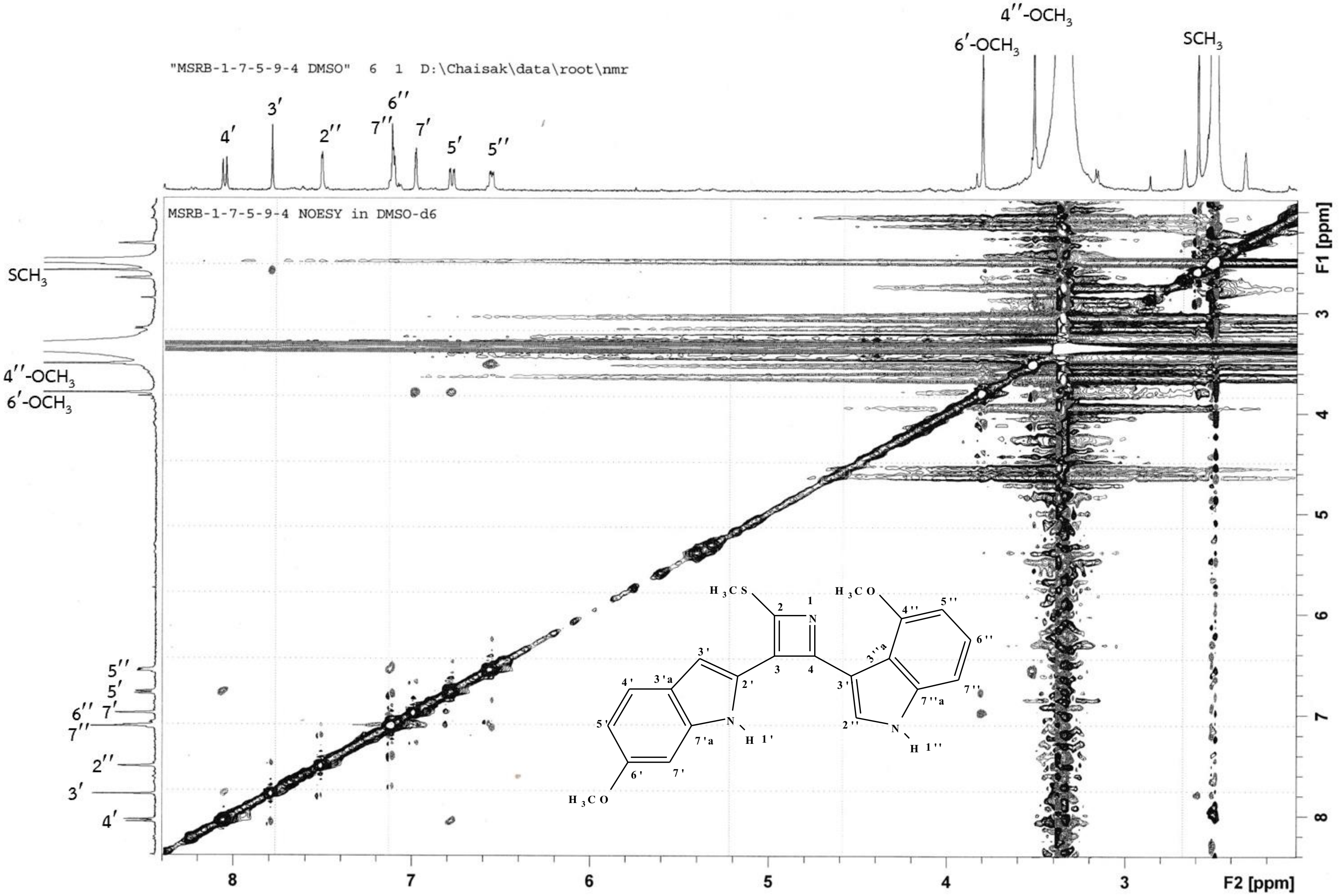

Figure S69. The NOESY spectrum of compound **6**

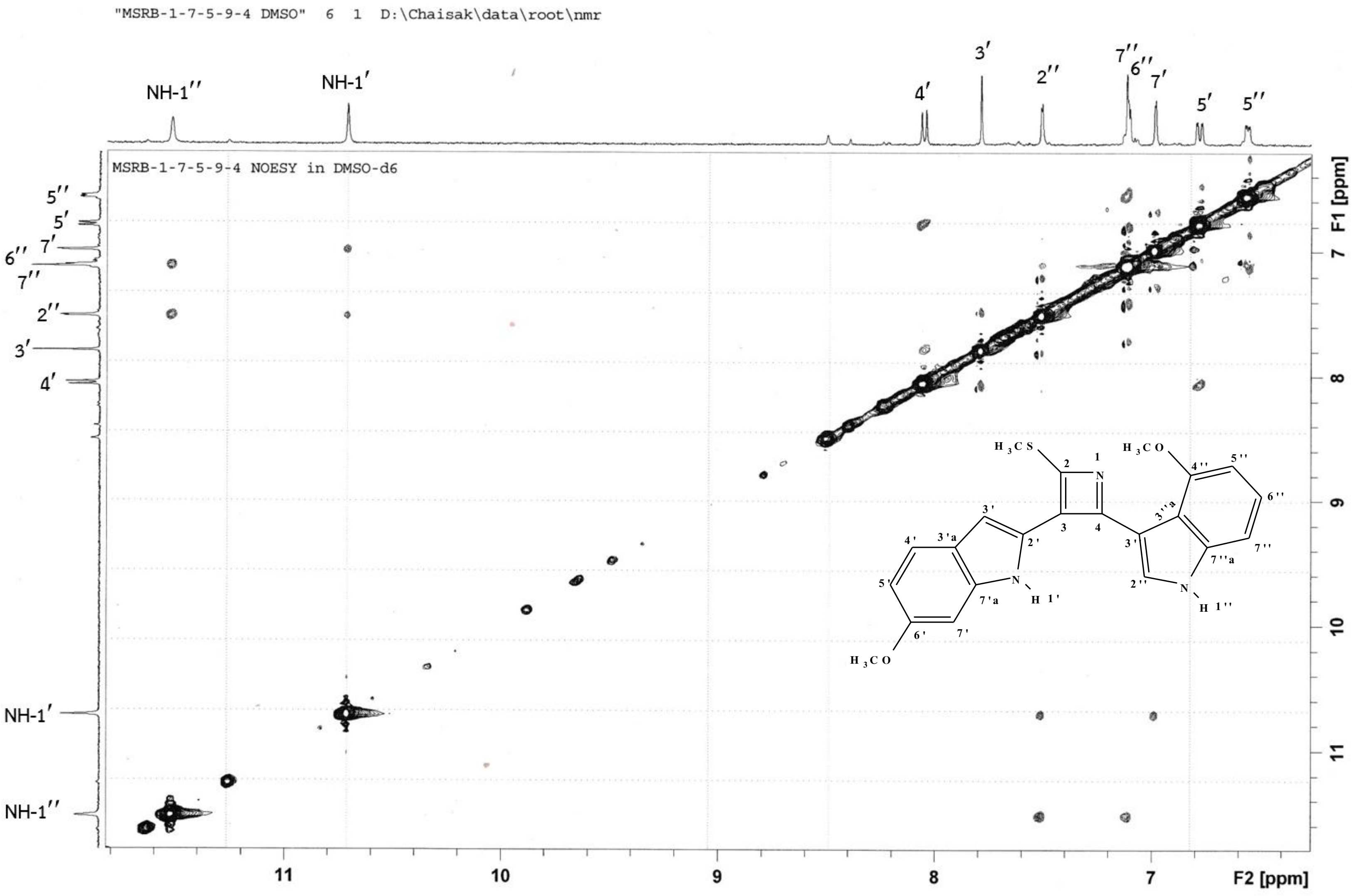

Figure S70. The NOESY spectrum of compound **6 (continued)**

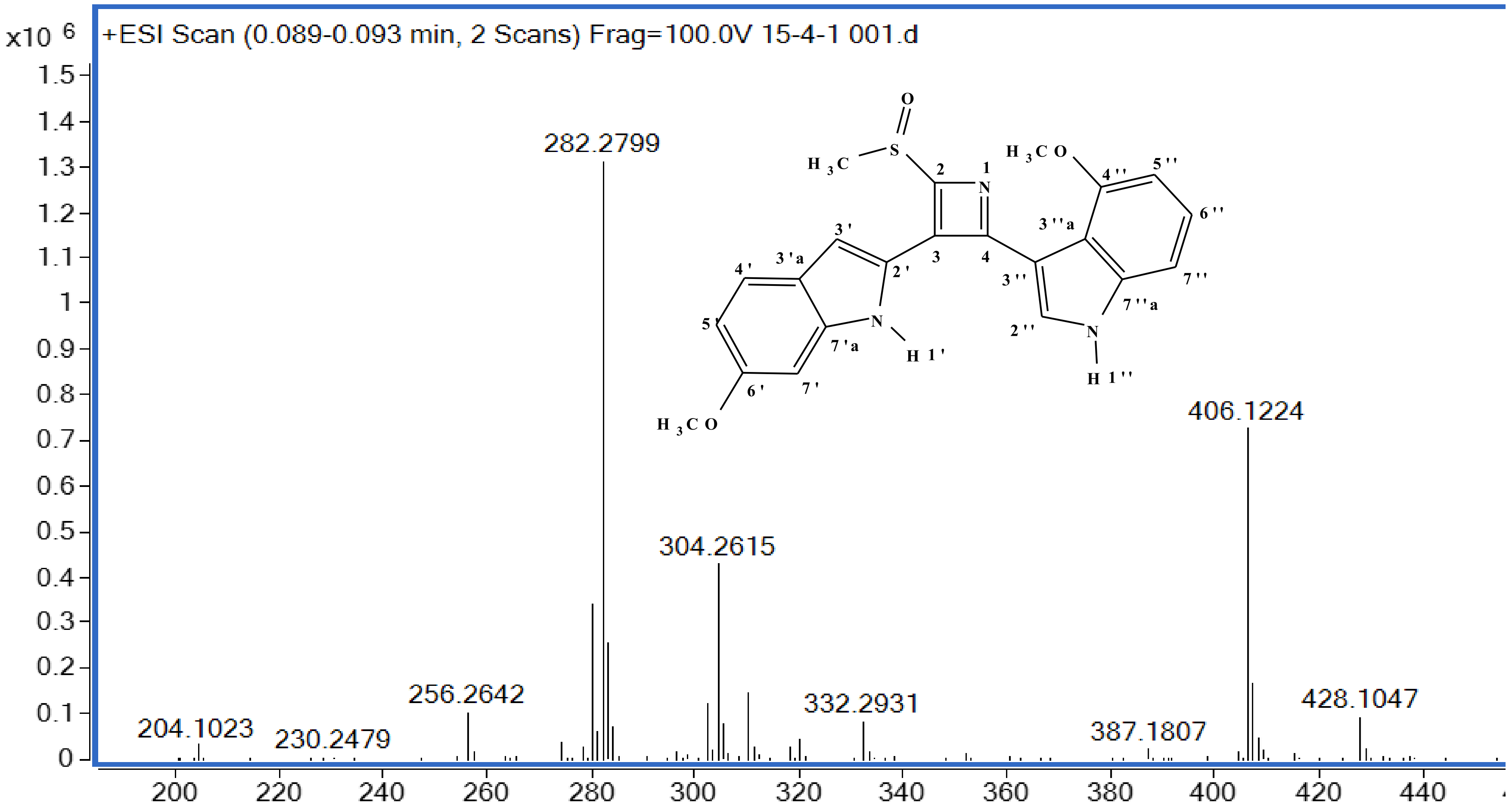

Figure S71. The HR-ESI-MS spectrum of compound **7**

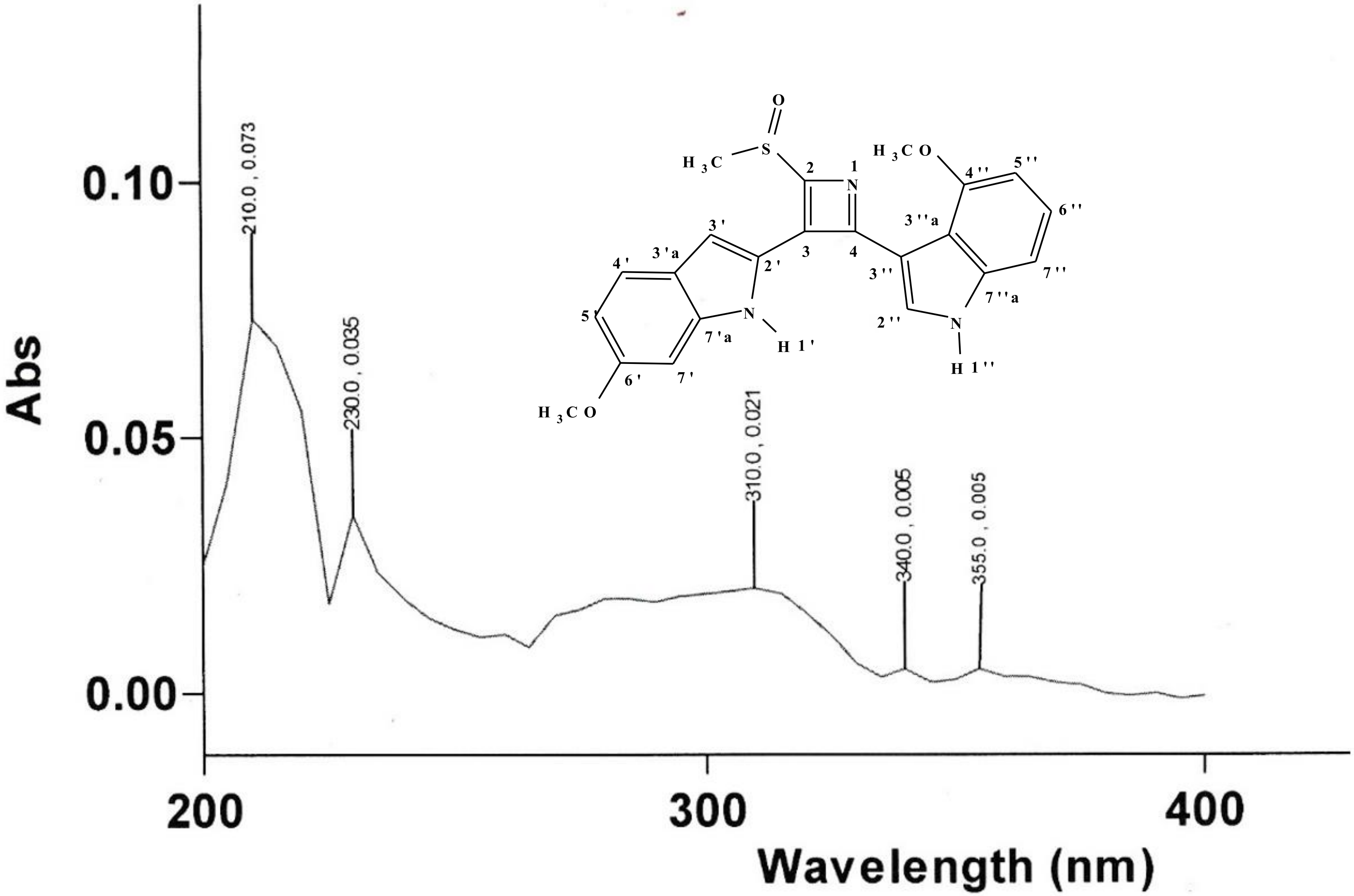

Figure S72. The UV spectrum of compound **7**

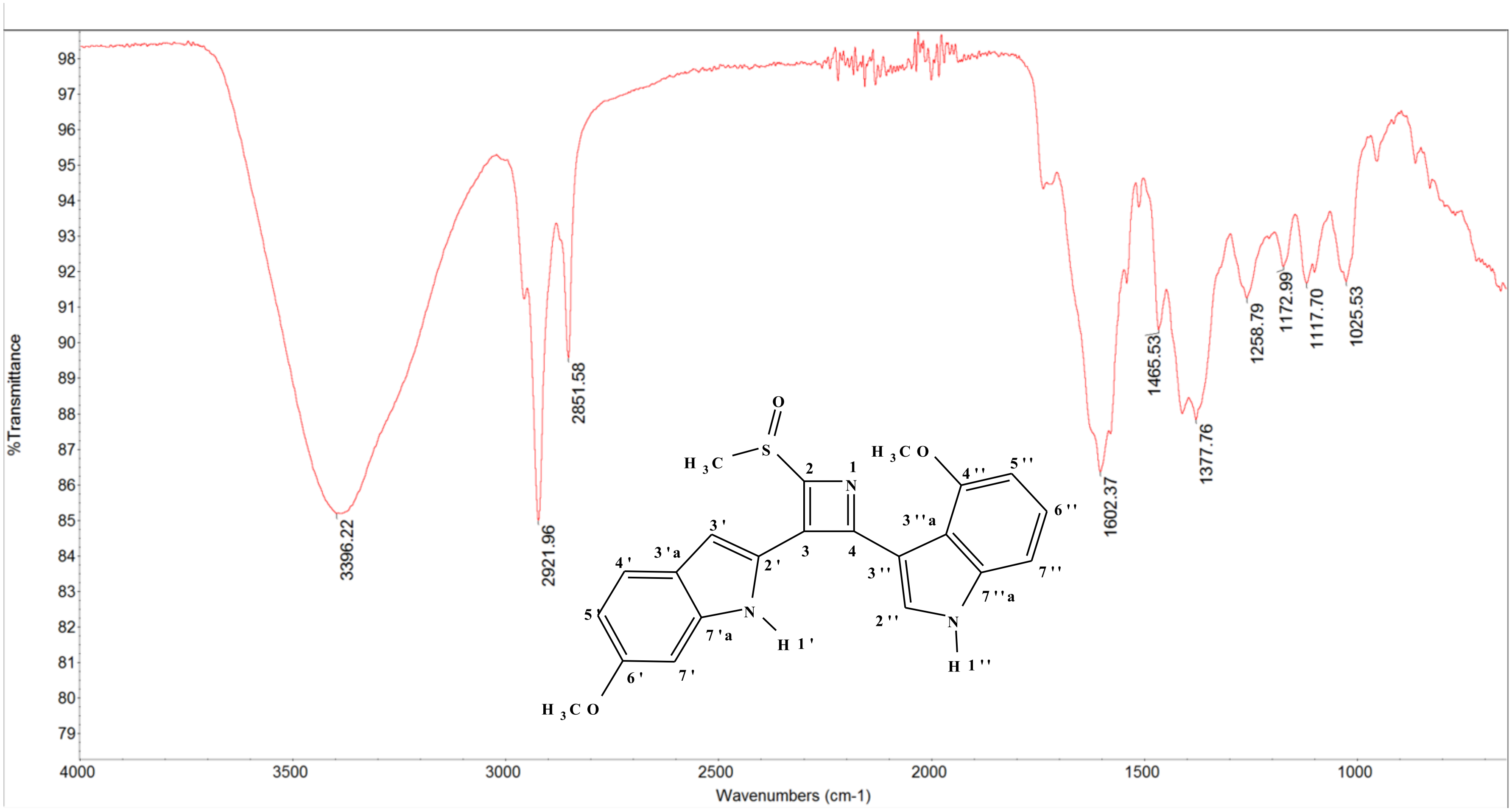

Figure S73. The IR spectrum of compound **7** (ATR)

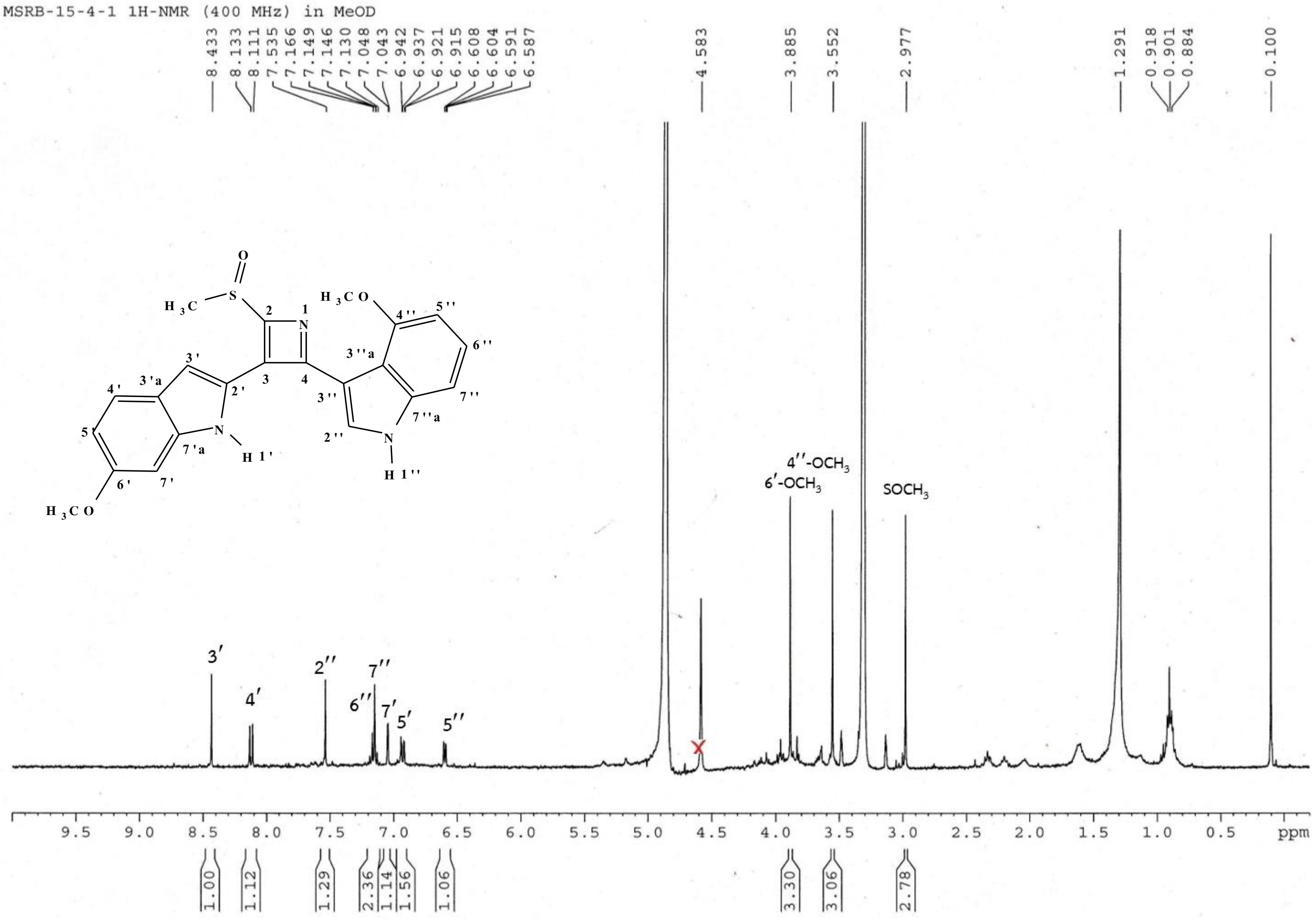

Figure S74. The ^{1}H NMR (400 MHz, CD3OD) spectrum of compound **7**

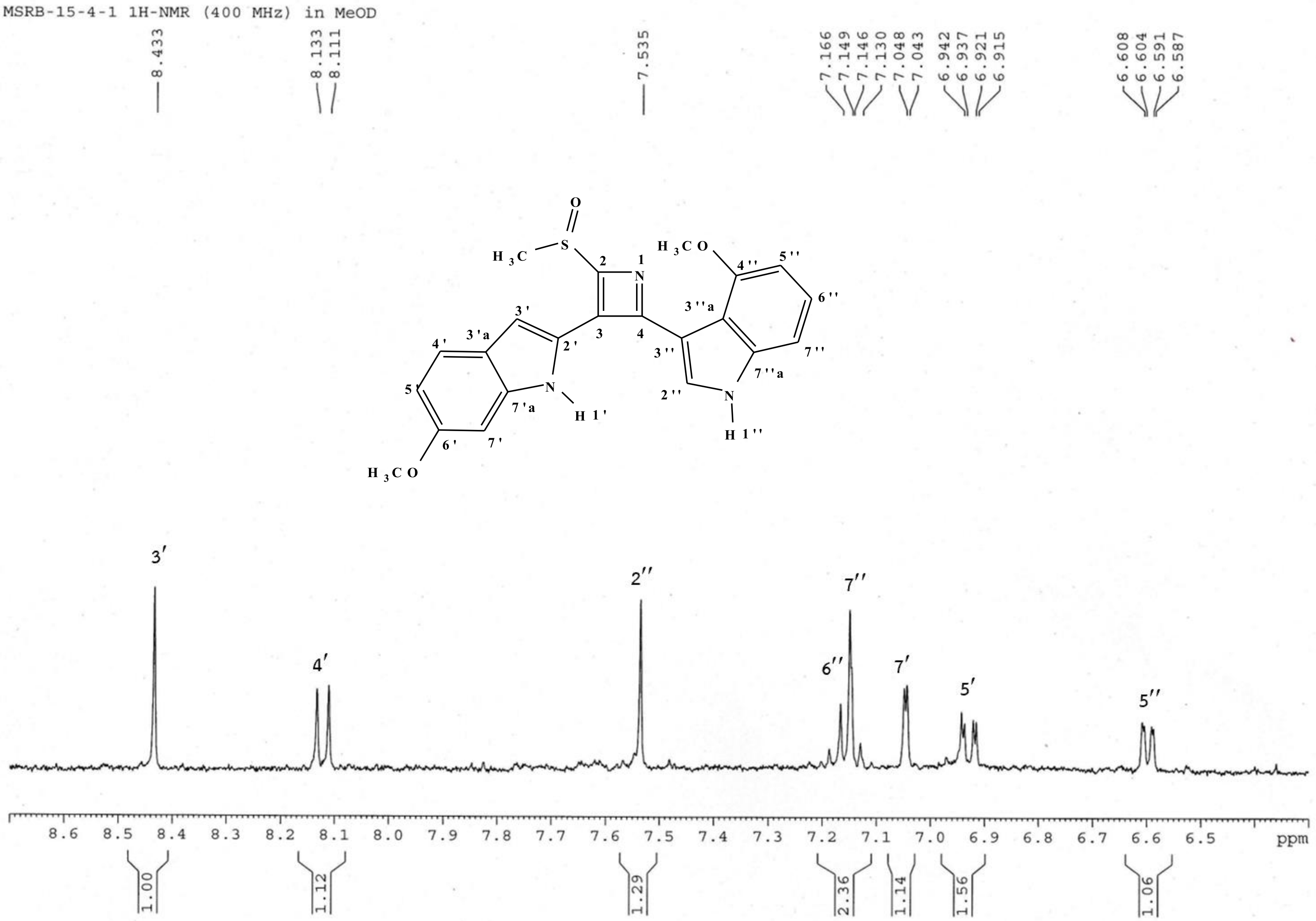

Figure S75. The ^{1}H NMR (400 MHz, CD_3OD) spectrum of compound **7 (continued)**

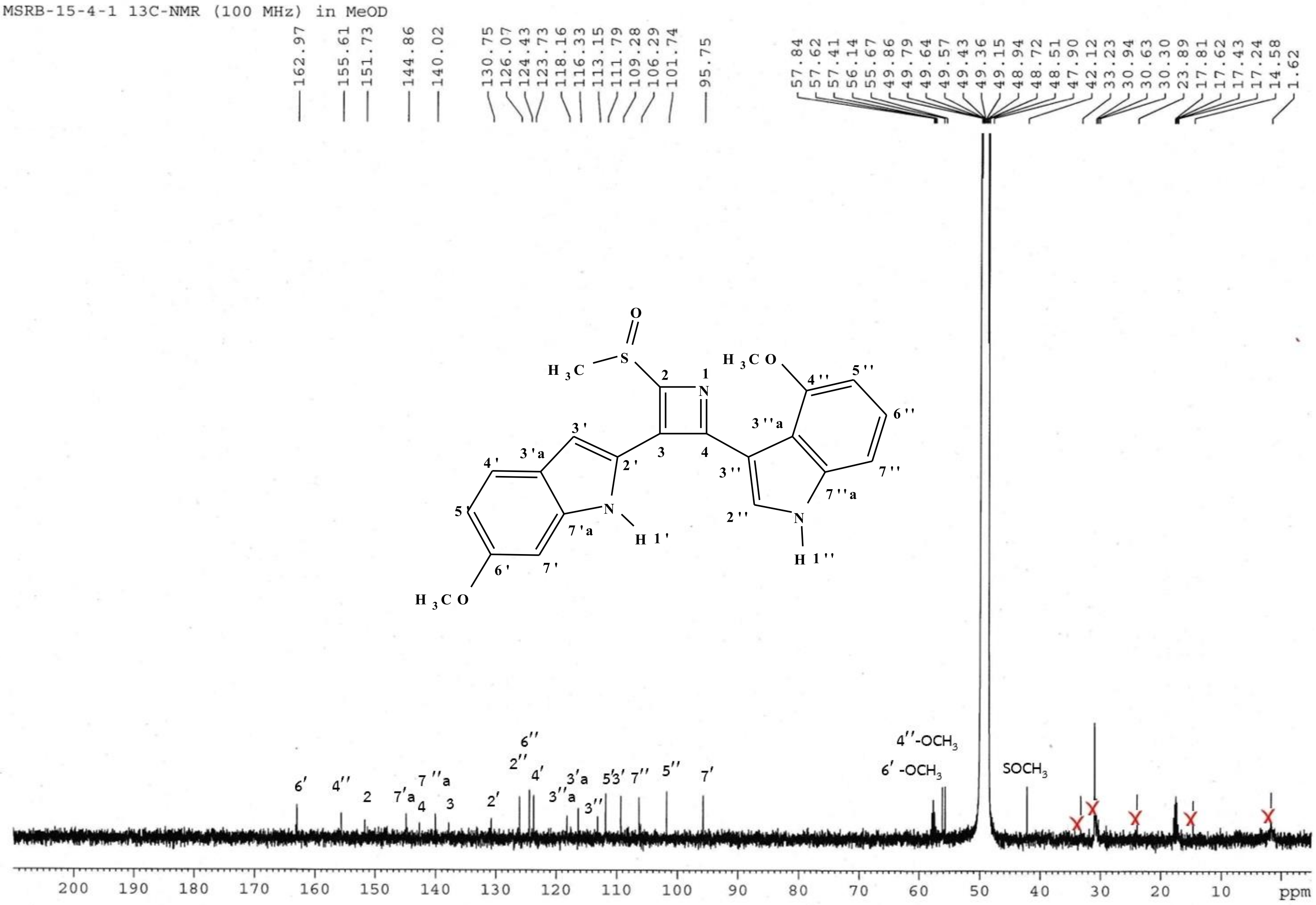

Figure S76. The ^{13}C NMR (100 MHz, CD3OD) spectrum of compound **7**

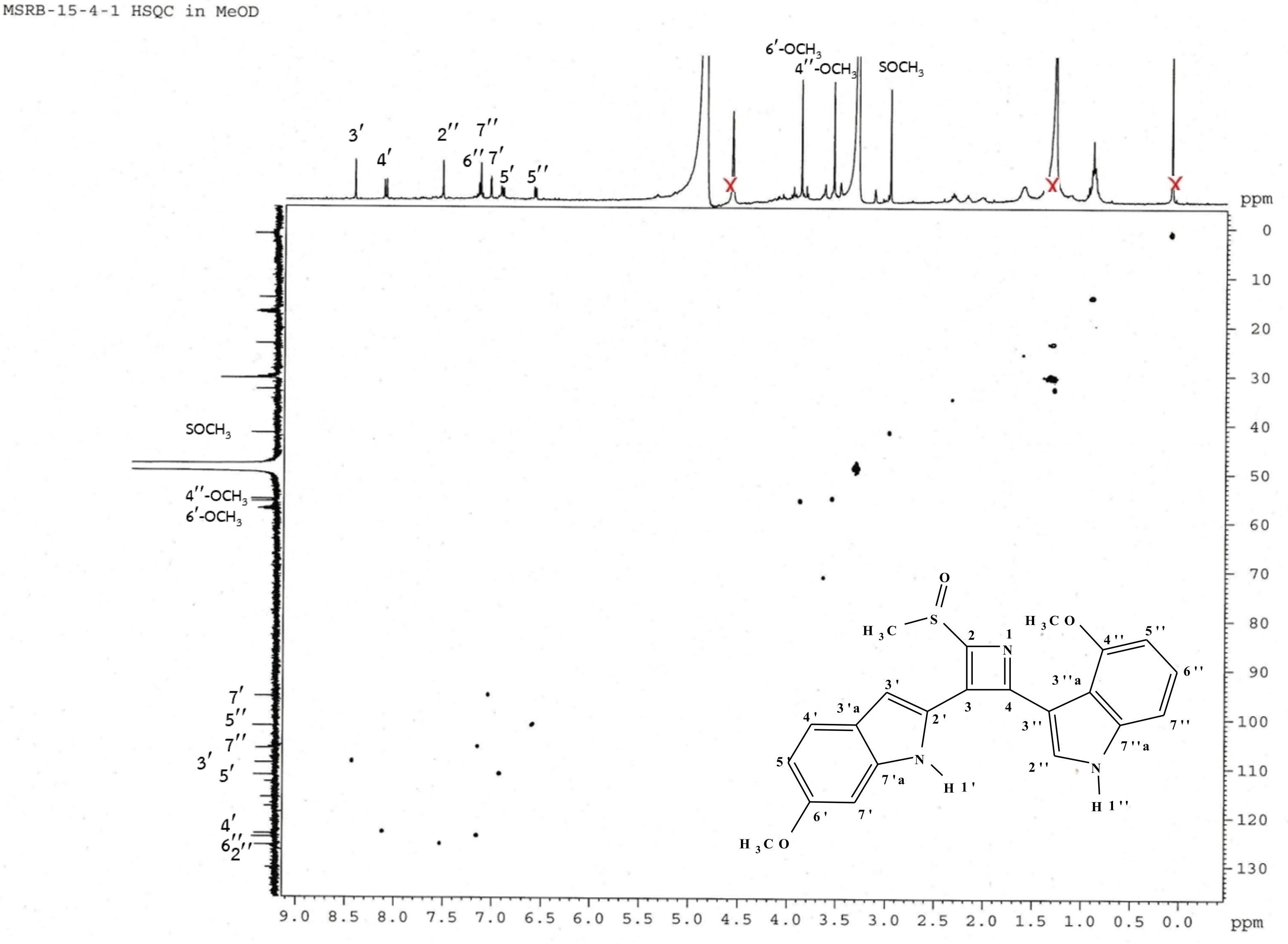

Figure S77. The HSQC spectrum of compound **7**

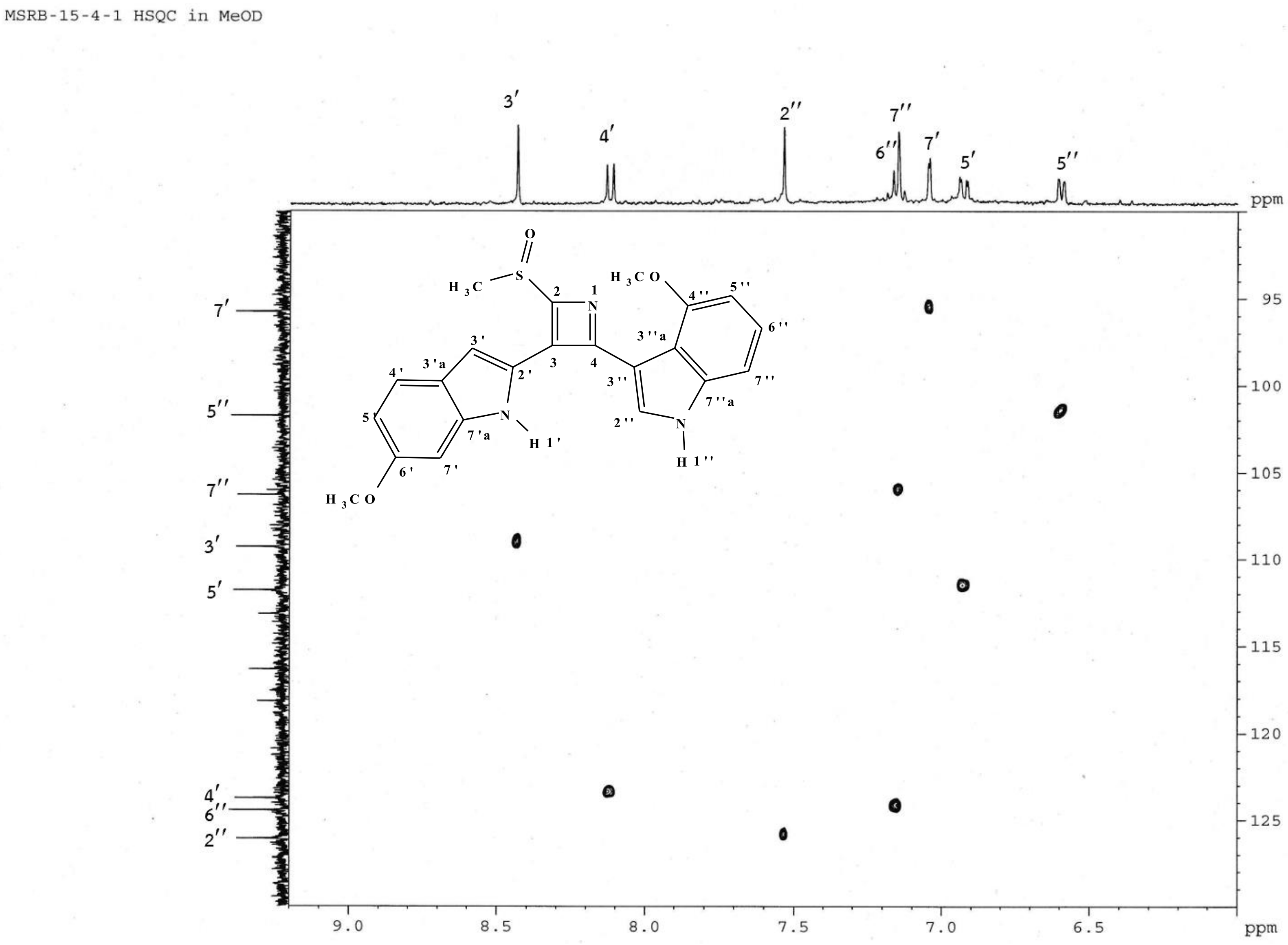

Figure S78. The HSQC spectrum of compound **7 (continued)**

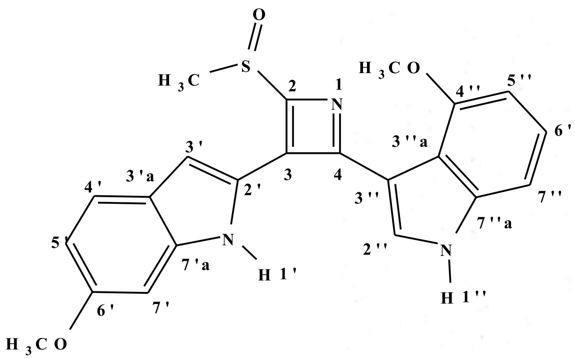

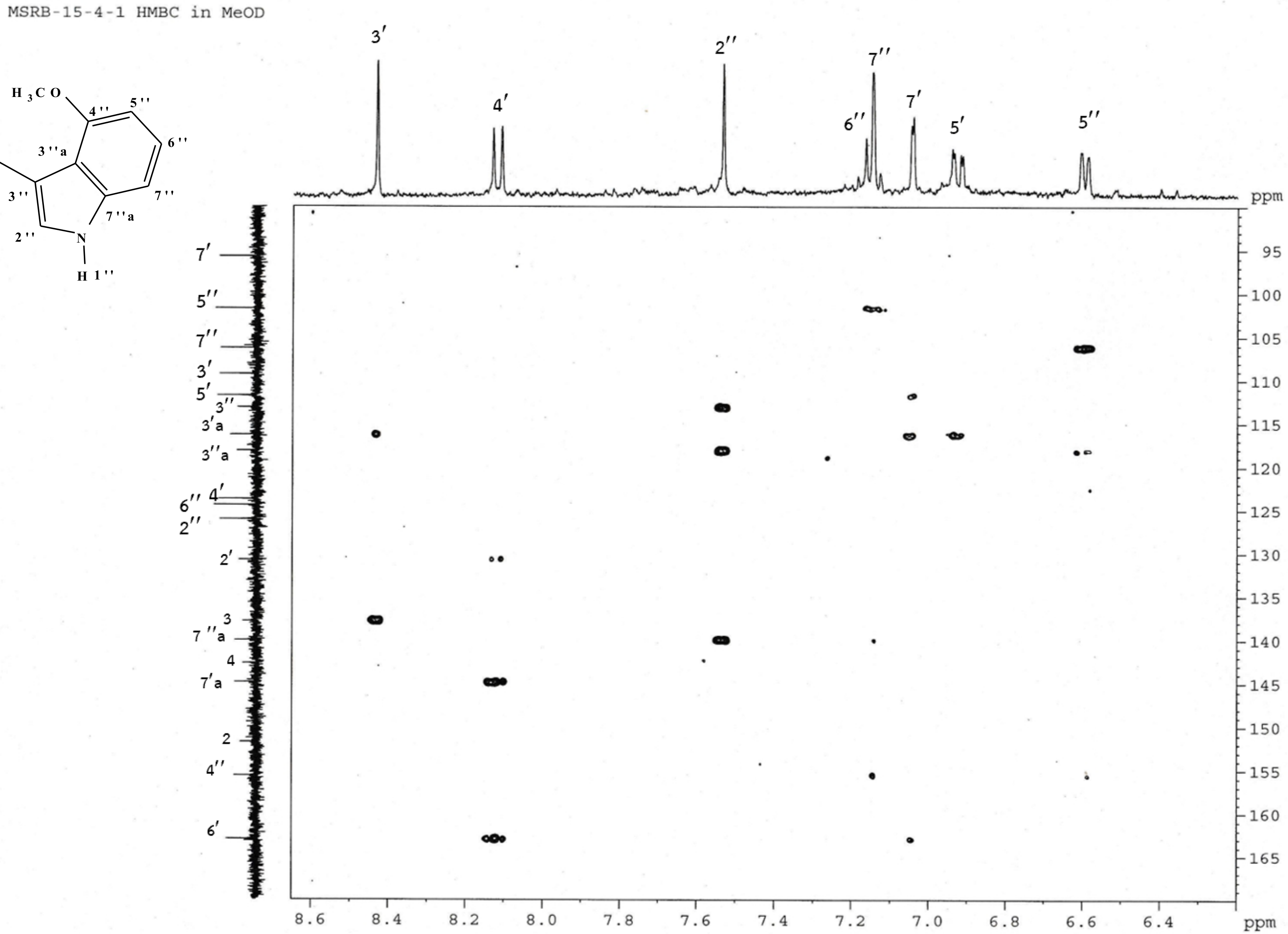

Figure S79. The HMBC spectrum of compound **7**

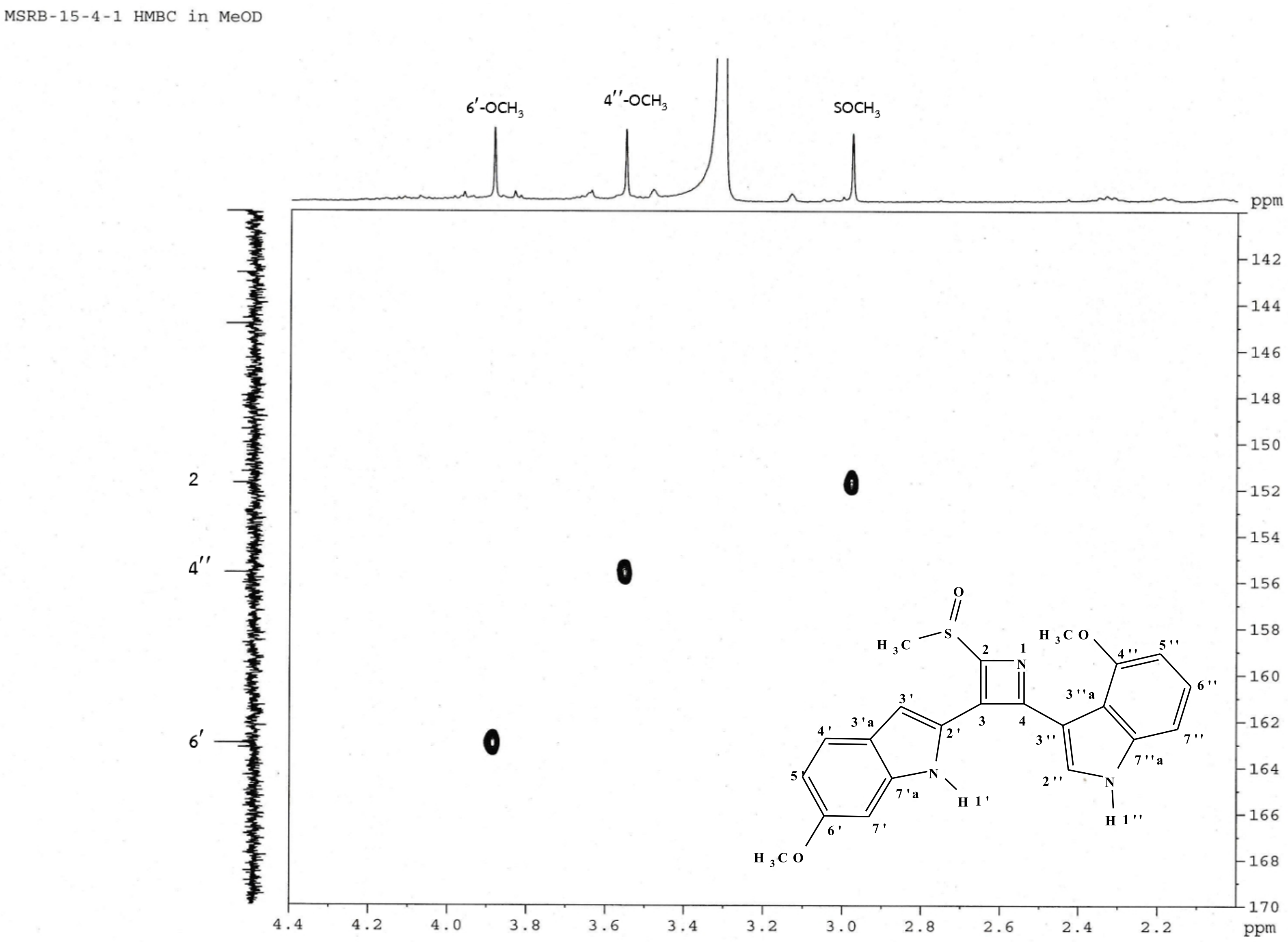

Figure S80. The HMBC spectrum of compound **7 (continued)**

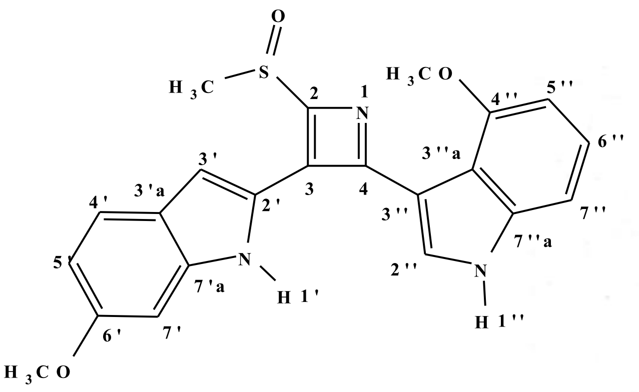

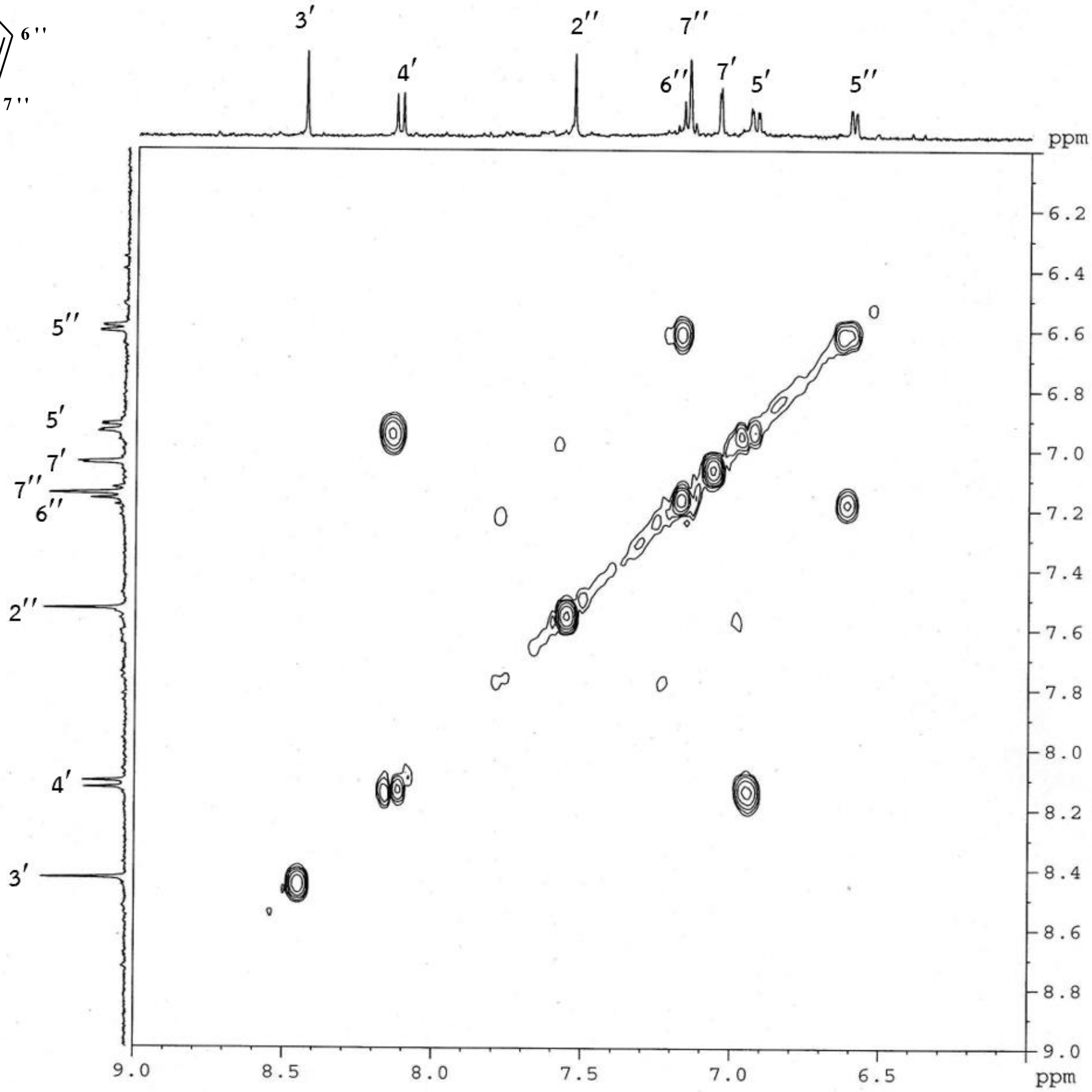

Figure S81. The ^1H-^1H COSY spectrum of compound **7**

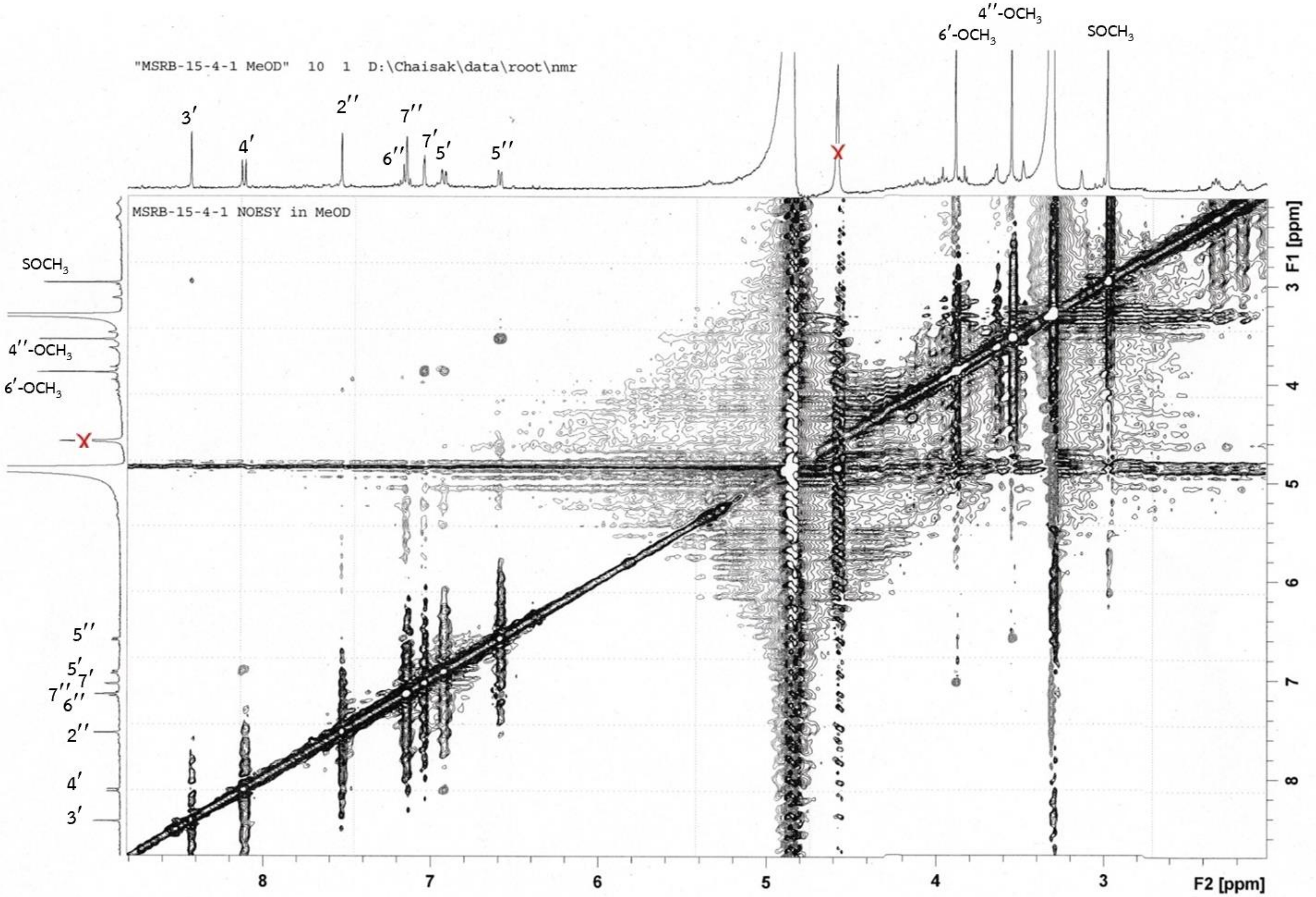

Figure S82. The NOESY spectrum of compound **7**

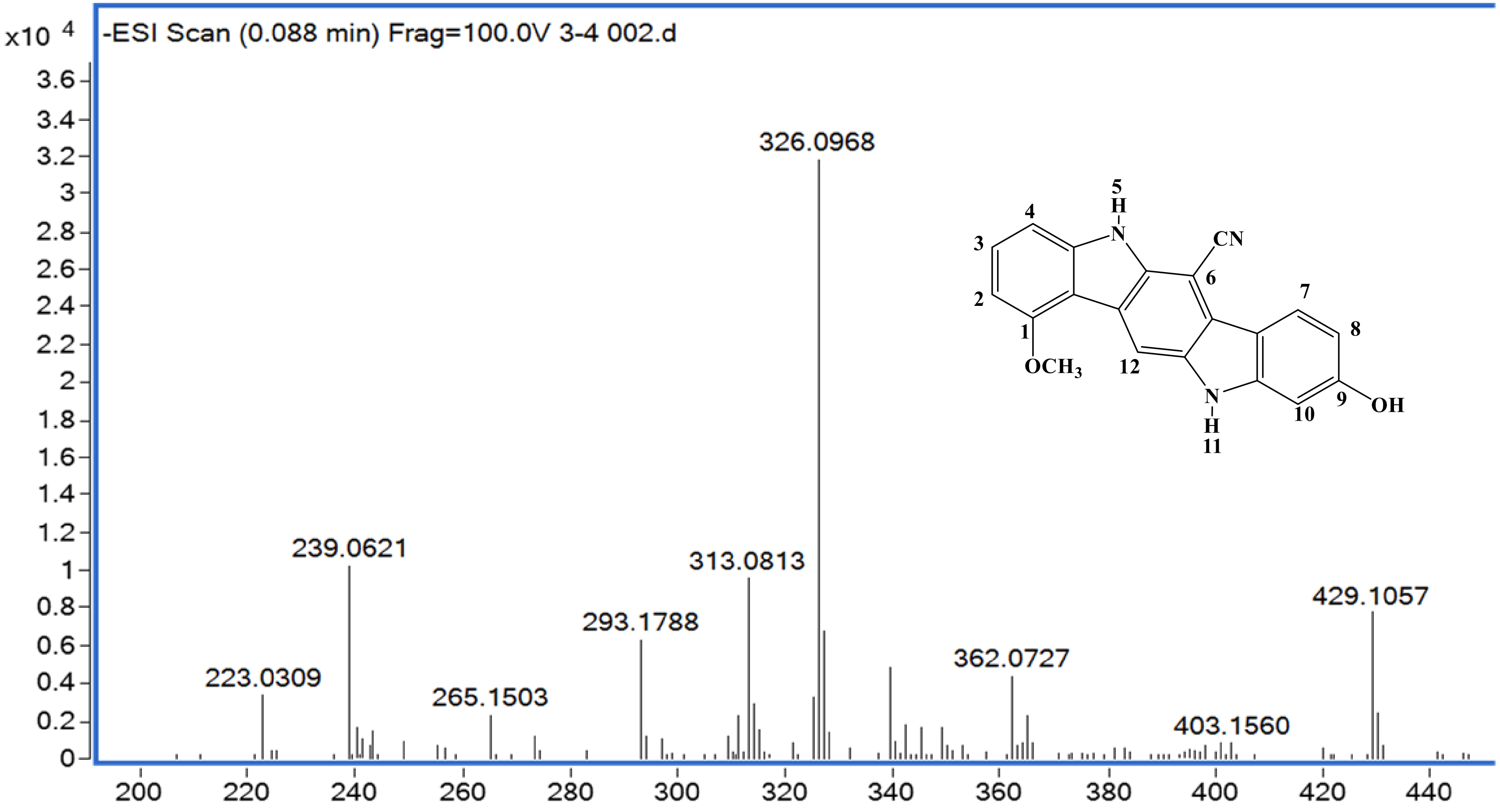

Figure S83. The HR-ESI-MS spectrum of compound **8**

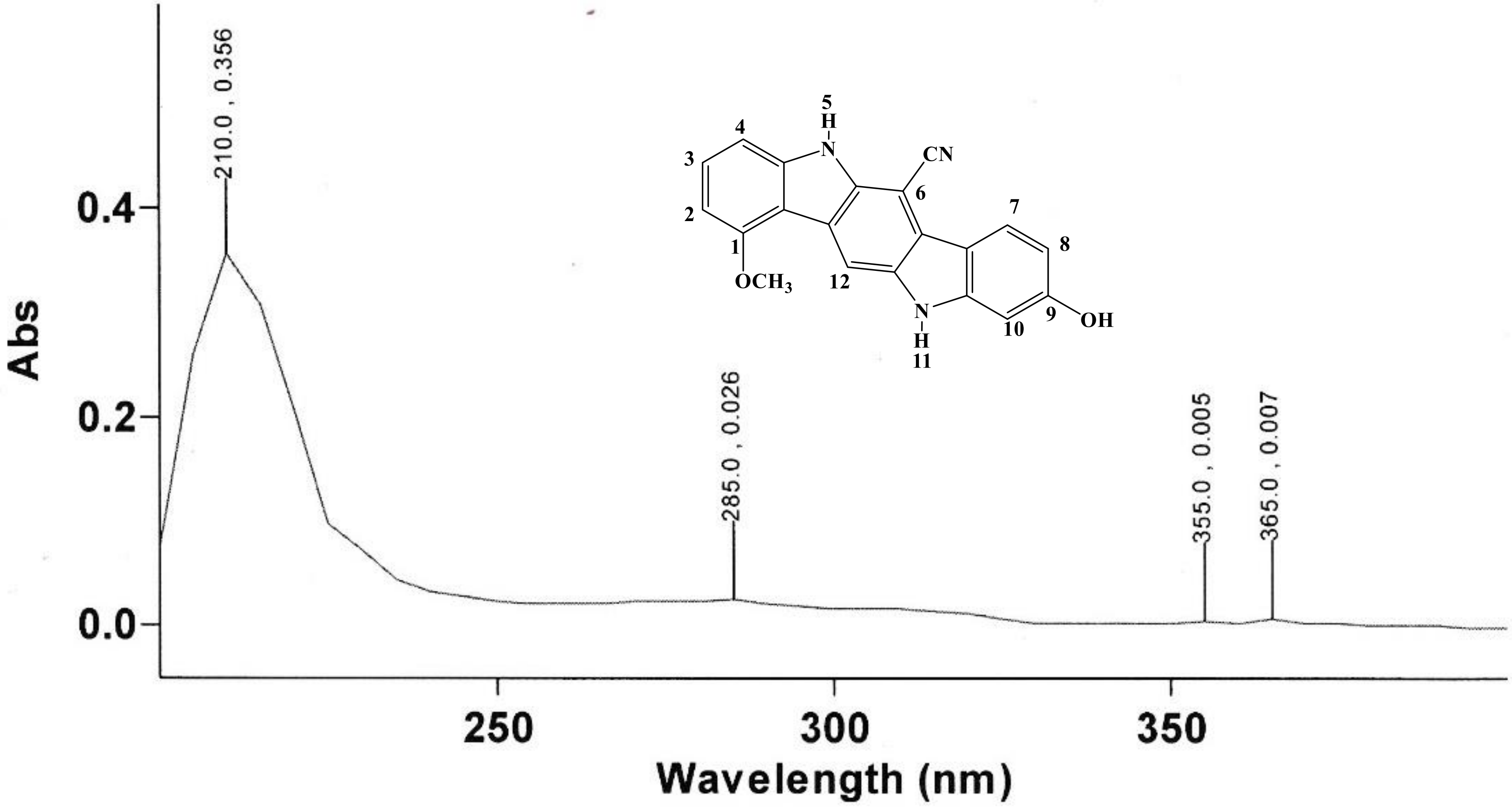

Figure S84. The UV spectrum of compound **8**

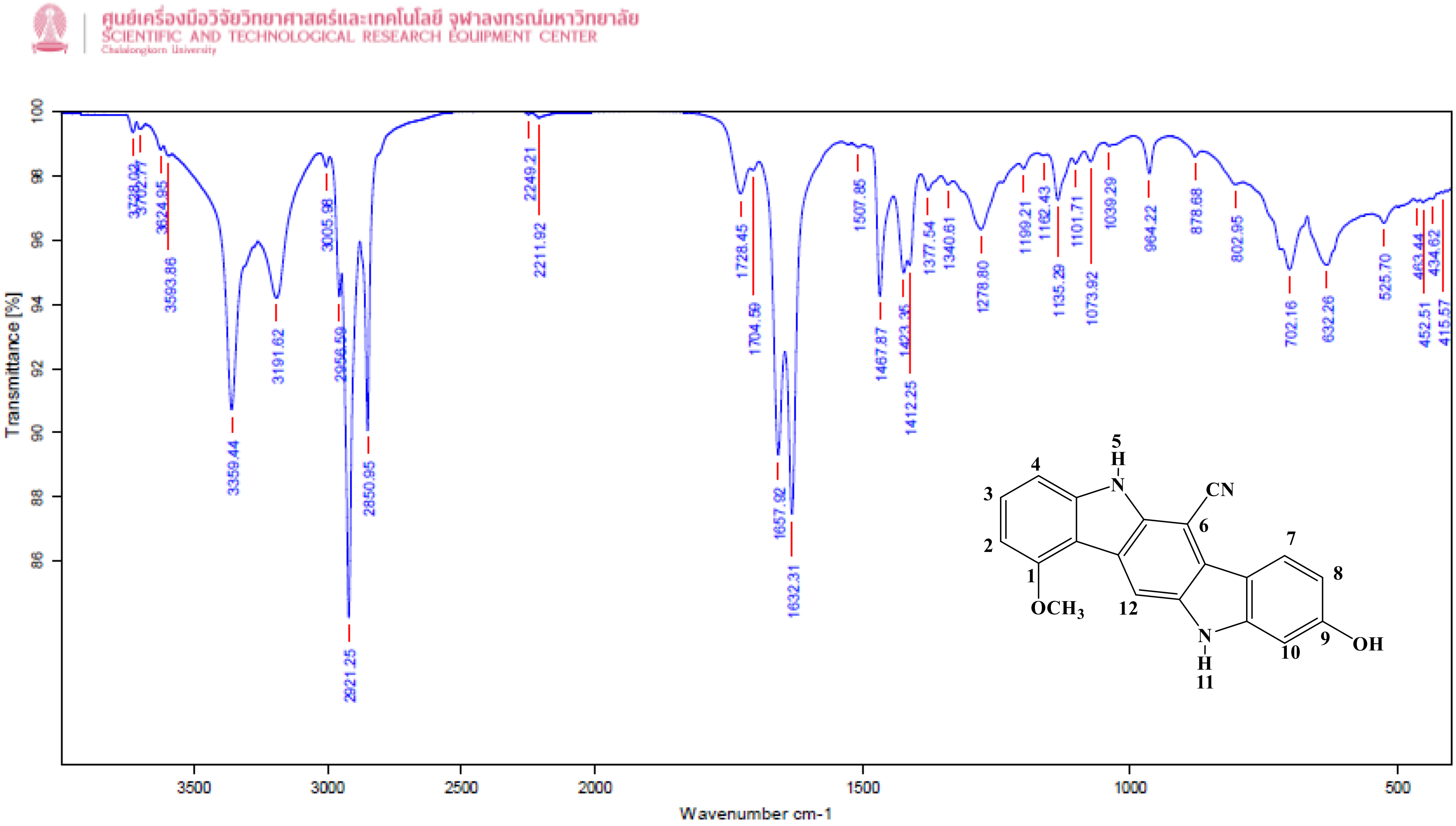

Figure S85. The IR spectrum of compound **8** (ATR)

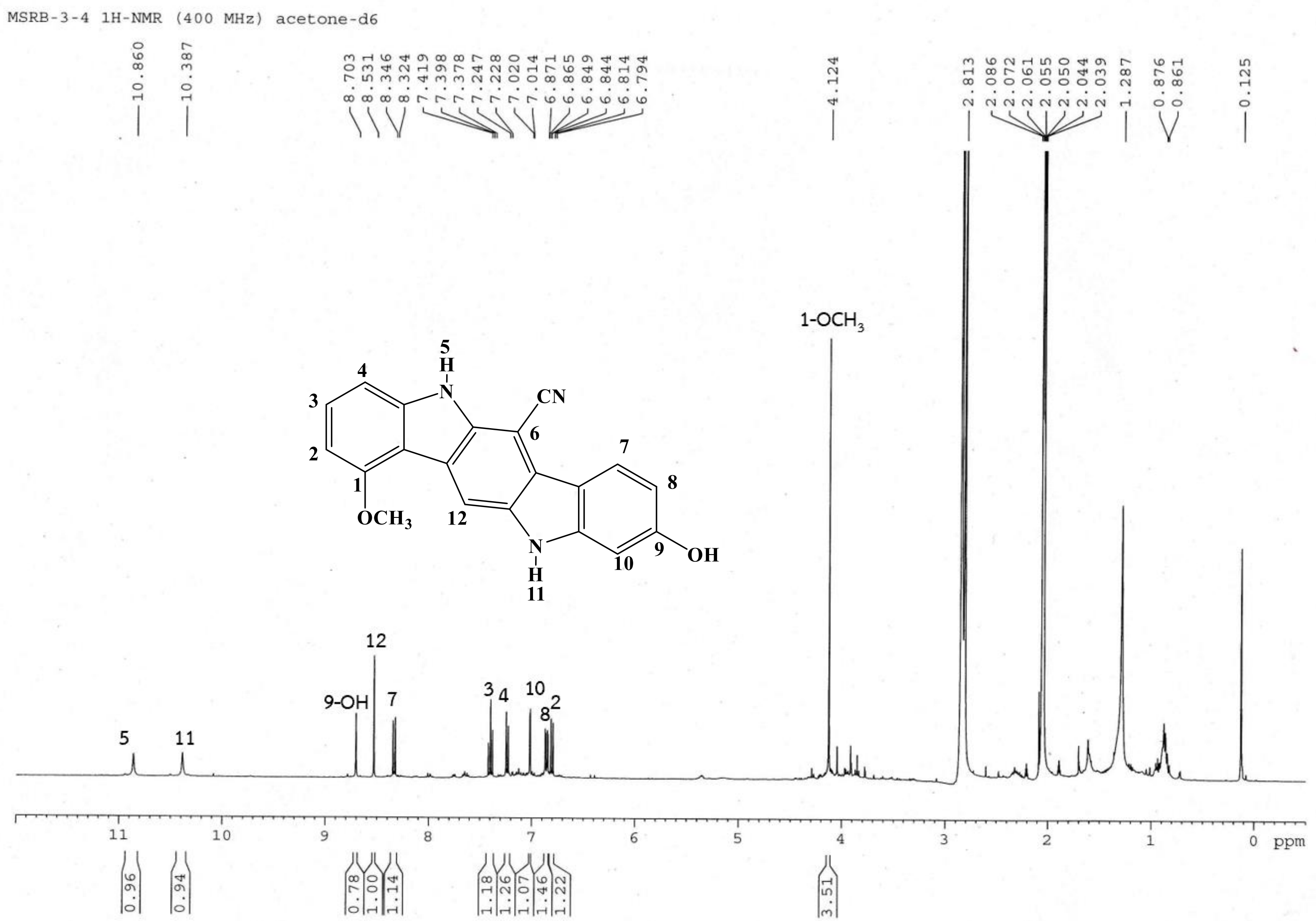

Figure S86. The ^{1}H NMR (400 MHz, acetone-d_6) spectrum of compound **8**

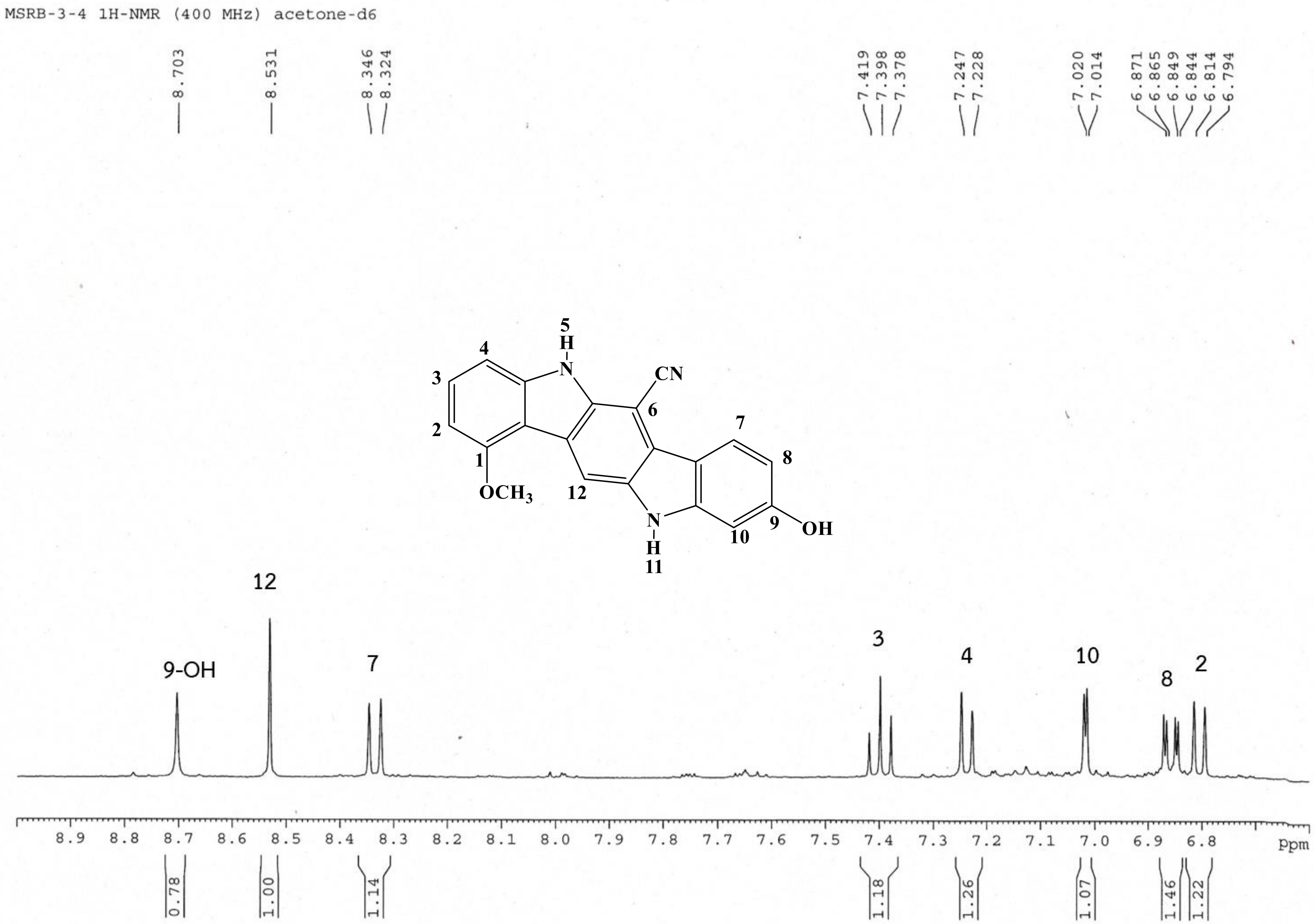

Figure S87. The ^{1}H NMR (400 MHz, acetone-d_6) spectrum of compound **8 (continued)**

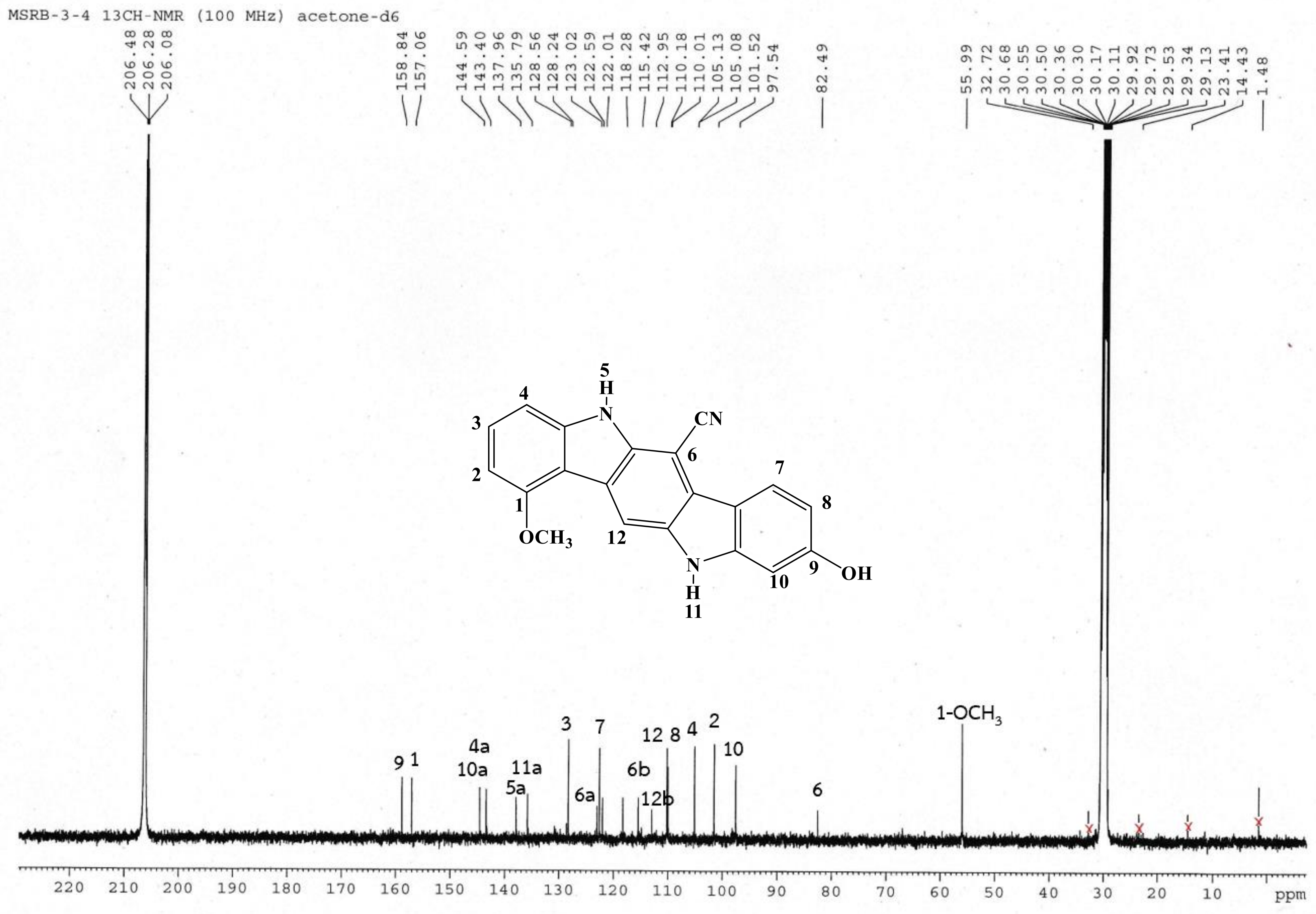

Figure S88. The ^{13}C NMR (100 MHz, acetone-*d*$_6$) spectrum of compound **8**

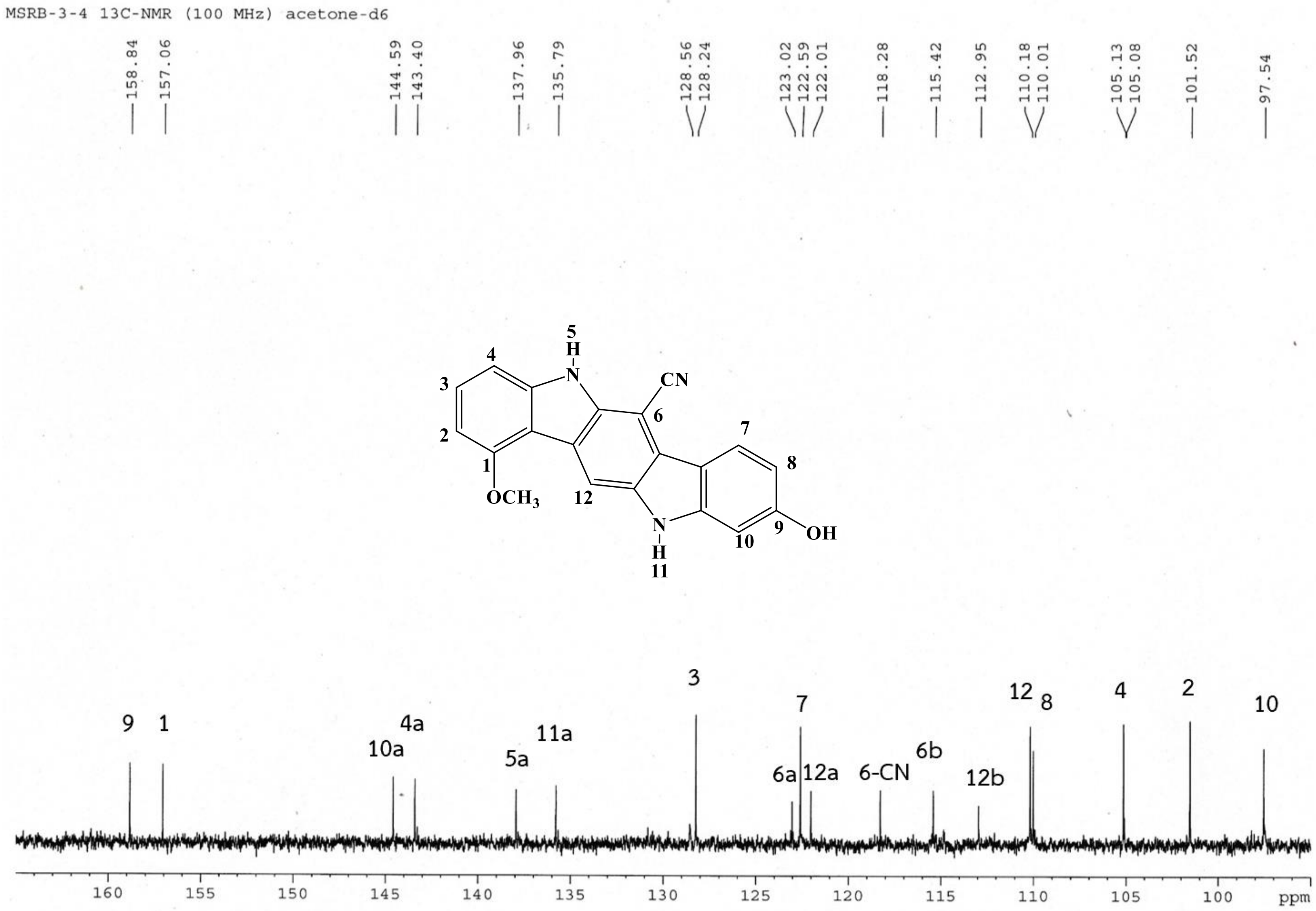

Figure S89. The ^{13}C NMR (100 MHz, acetone-d_6) spectrum of compound **8 (continued)**

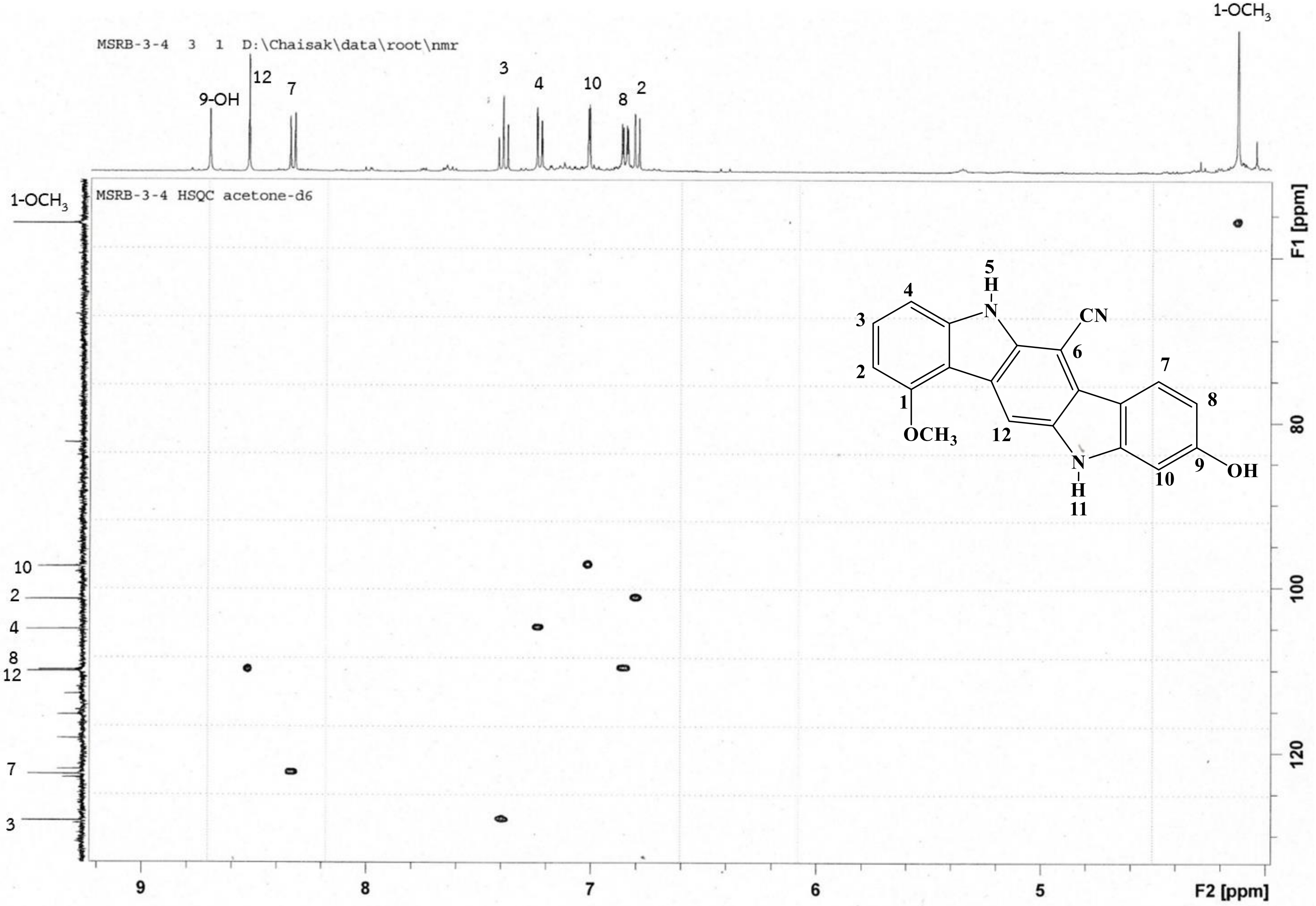

Figure S90. The HSQC spectrum of compound **8**

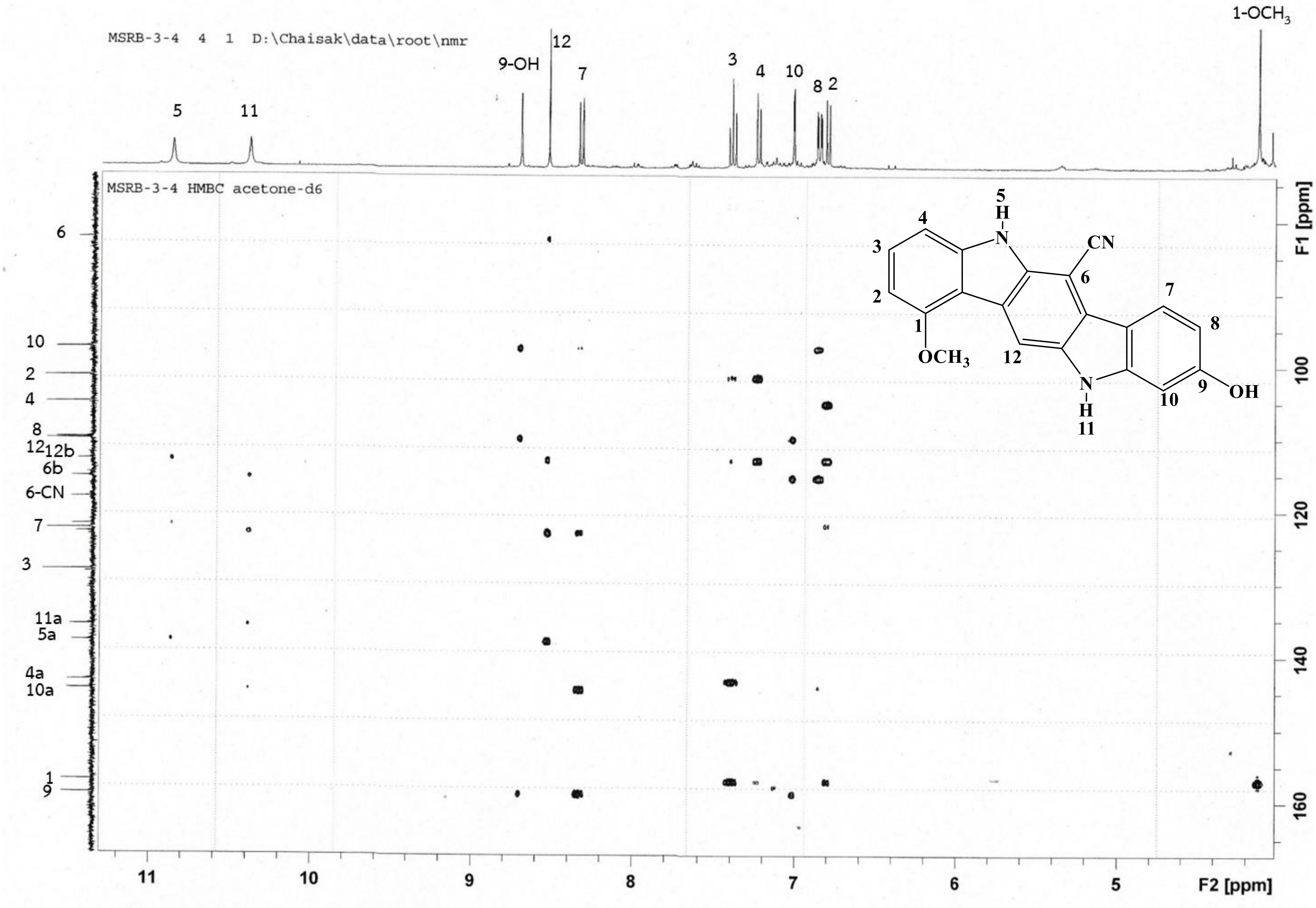

Figure S91. The HMBC spectrum of compound **8**

MSRB-3-4 HMBC acetone-d6

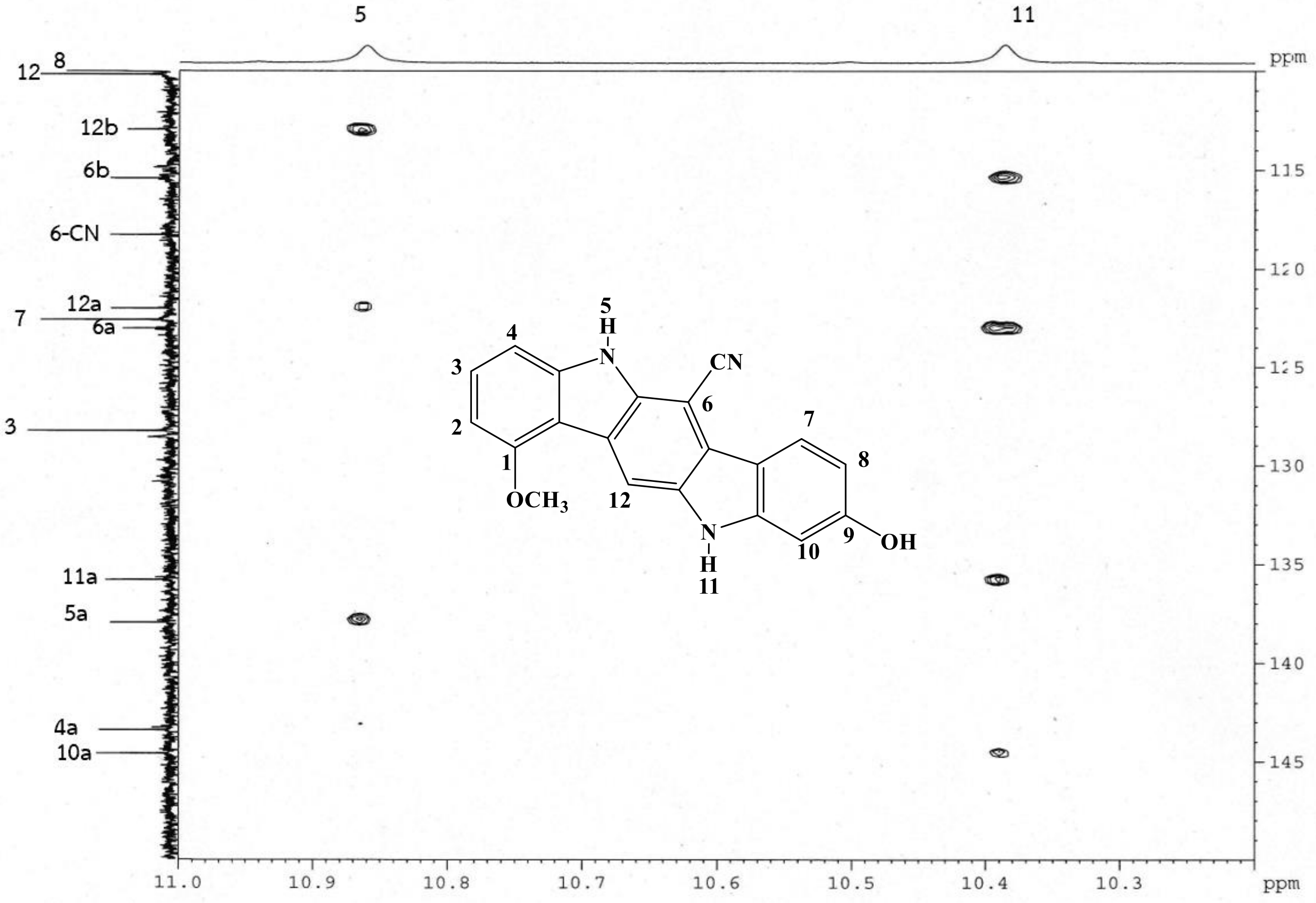

Figure S92. The HMBC spectrum of compound **8 (continued)**

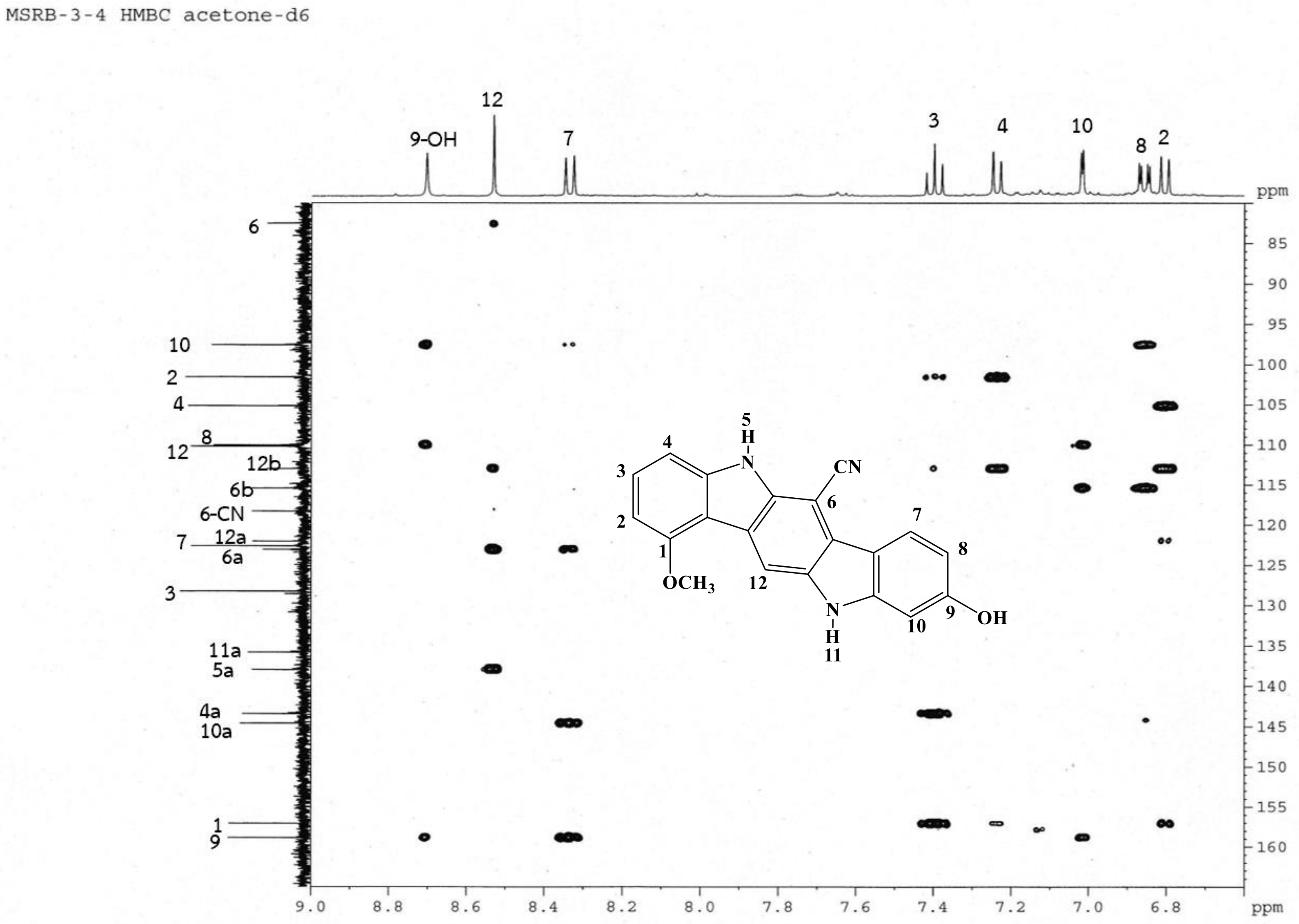

Figure S93. The HMBC spectrum of compound **8 (continued)**

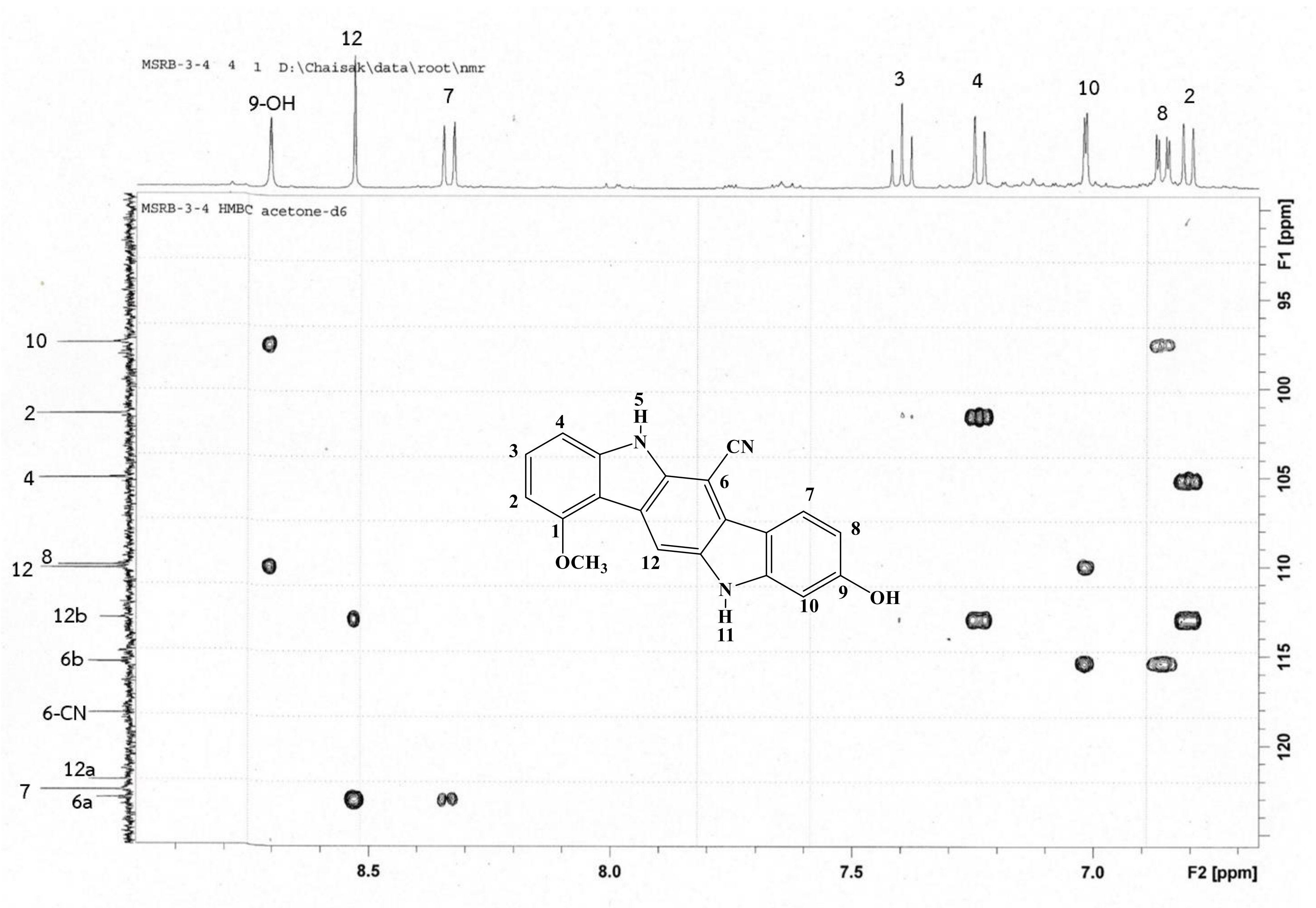

Figure S94. The HMBC spectrum of compound **8 (continued)**

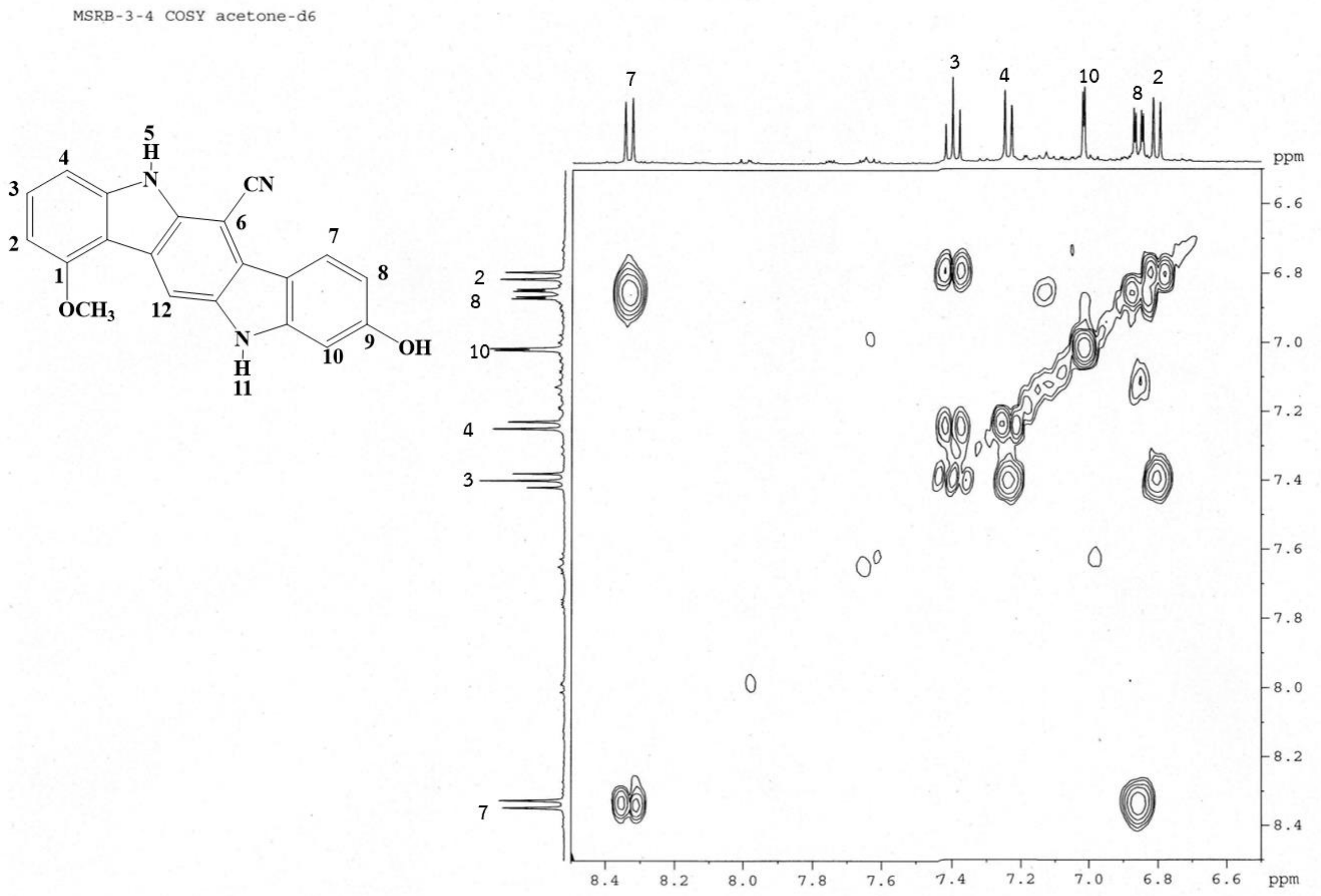

Figure S95. The ^{1}H-^{1}H COSY spectrum of compound **8**

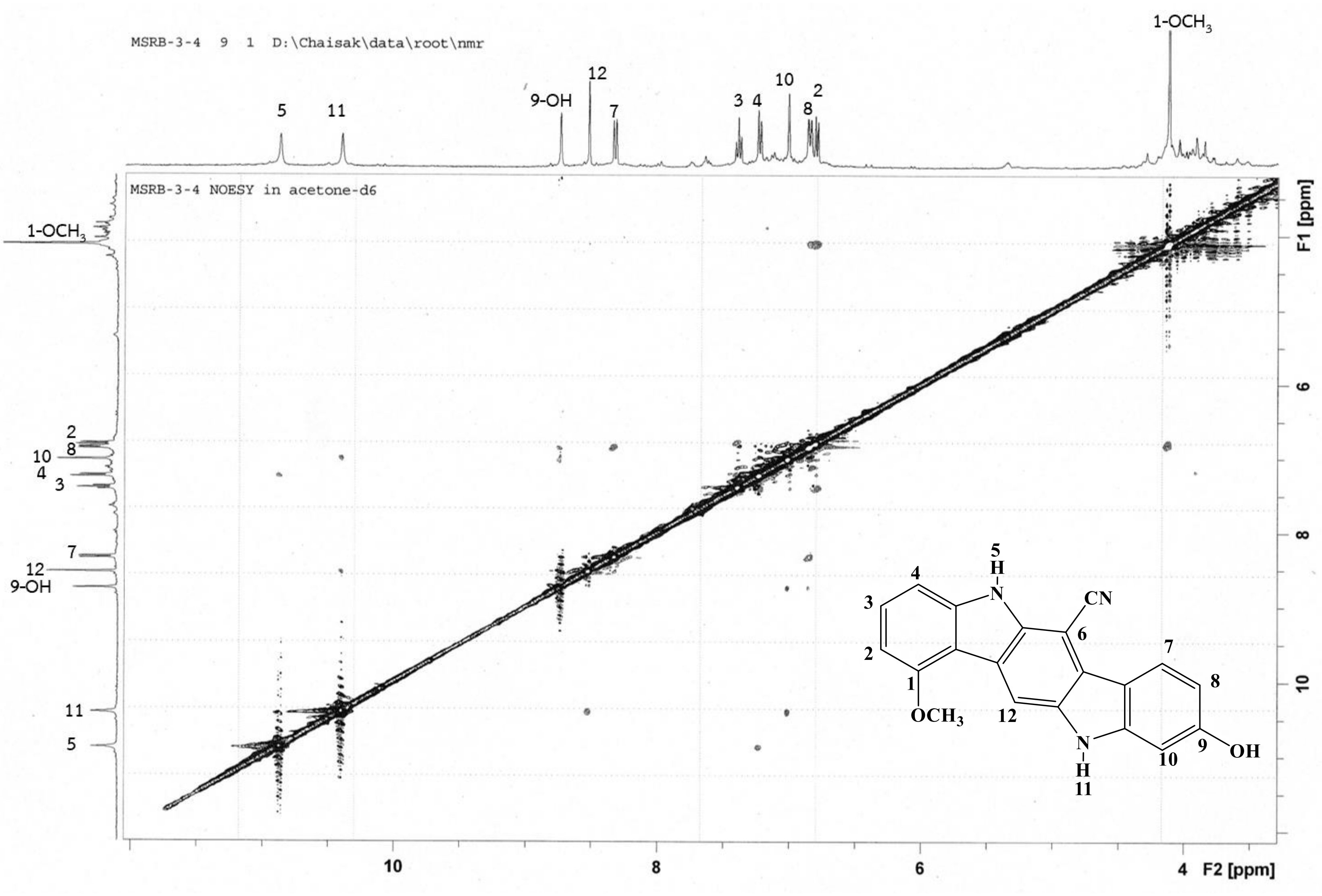

Figure S96. The NOESY spectrum of compound **8**

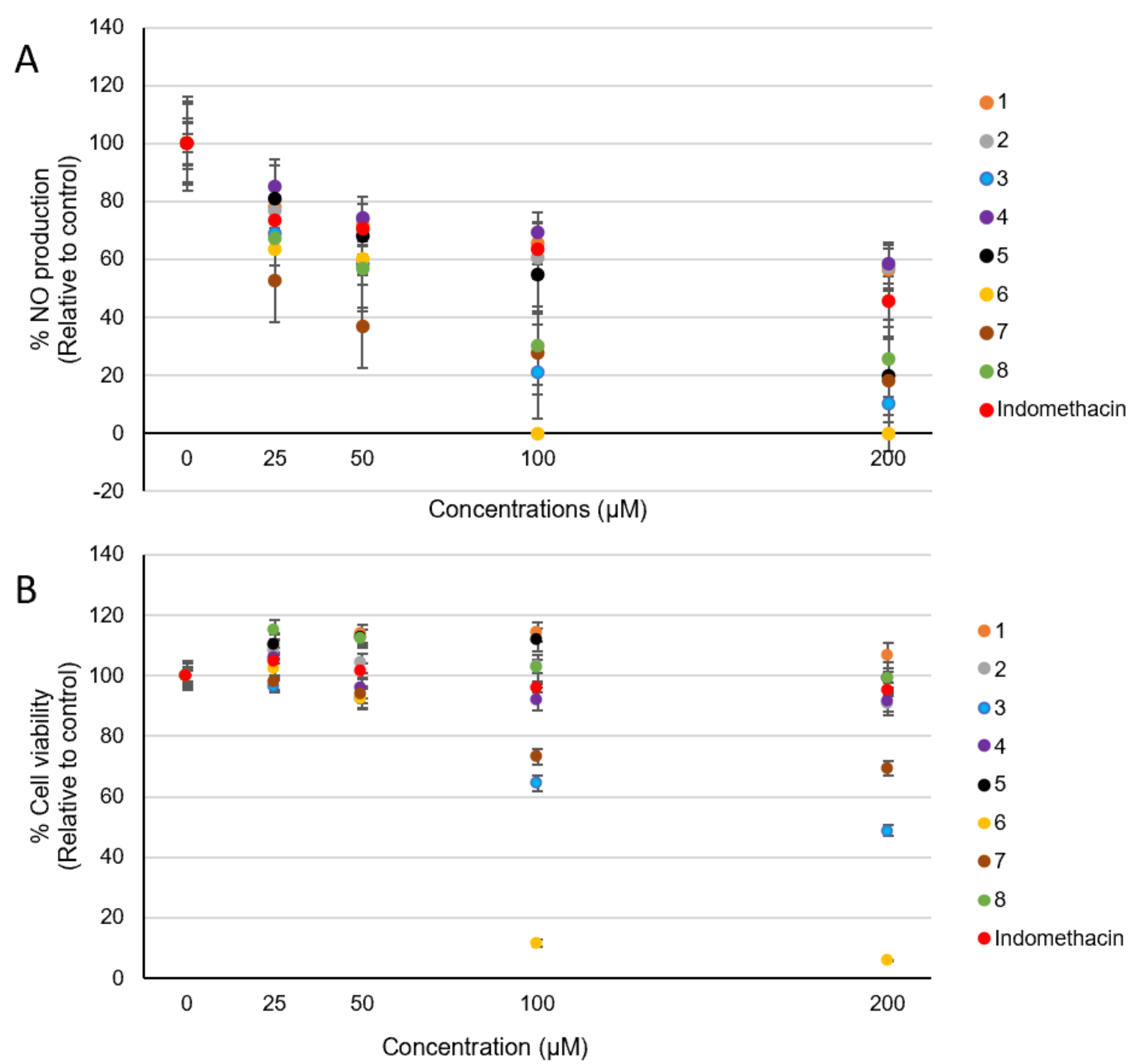

Figure S97. Effects of compounds isolated from *M. siamensis* roots against LPS-induced nitric oxide production in RAW 264.7 cells. (A) Nitric oxide production, and (B) cell viability were measured using Griess reagent kit and MTT assay (n =3), respectively. Cells were treated with compounds at the indicated concentration for 24 h before exposure to 100 ng/mL LPS. Indomethacin was used as a positive control.

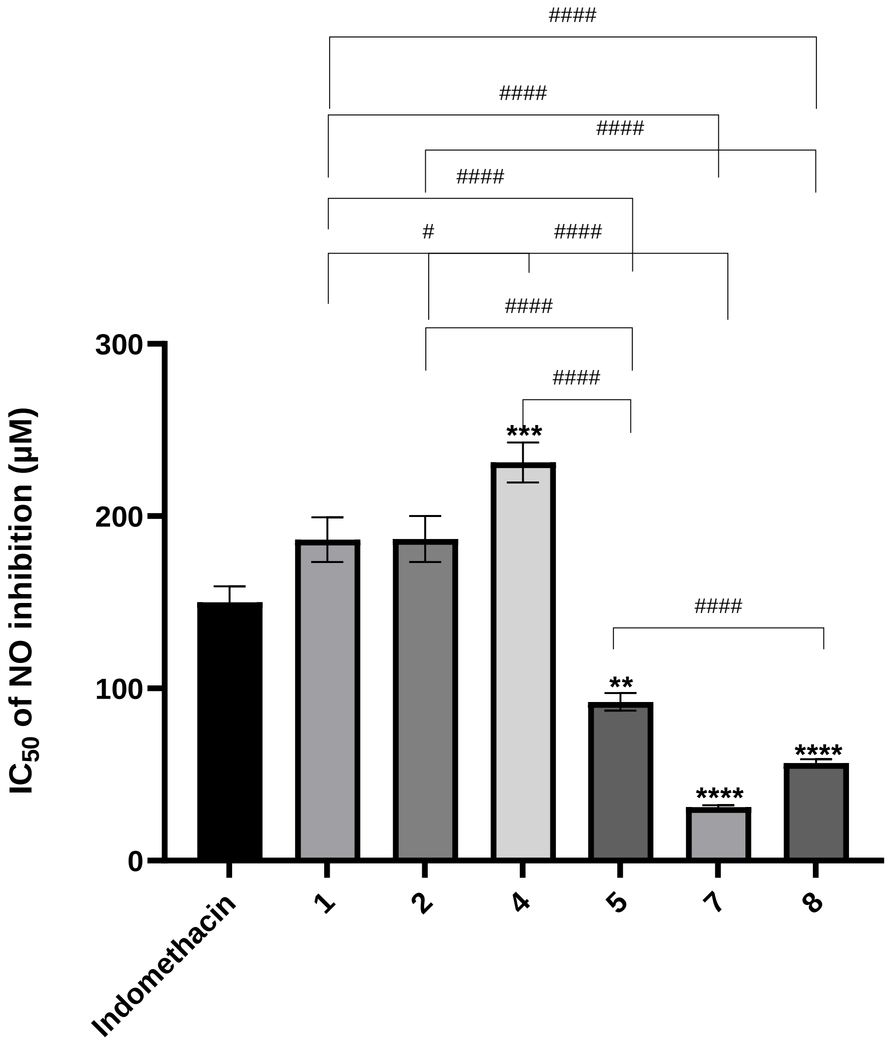

Figure S98. The IC_{50} values of compound isolated from *M. siamensis* roots against LPS-induced nitric oxide production in RAW 264.7 cells. The differences between means of the individual groups were analyzed using one-way analysis of variance (ANOVA) via GraphPad Prism 9.3.1 software (San Diego, CA, USA). **P< 0.005, ***p<0.001, ****p<0.0001 versus Indomethacin. $^{\#}$P<0.05, $^{\#\#\#\#}$p<0.0001 between group
